# Supplementary material for: Hepatitis C Virus Infection and Intrinsic Disorder in the Signaling Pathways Induced by Toll-Like Receptors
Source: Biology (Basel). 2022 Jul 21;11(7):1091. doi: 10.3390/biology11071091 (PMC9312352; doi:10.3390/biology11071091)
Supplement: Supplementary file 1 [file biology-11-01091-s001.zip › biology-1761975-supplementary.pdf]

Supplementary Materials

Hepatitis C virus infection and intrinsic disorder in the signaling pathways induced by toll-like receptors

Elrashdy M. Redwan [1,2], Abdullah A. Aljadawi [1], and Vladimir N. Uversky [1,3,*]

[1] Biological Science Department, Faculty of Science, King Abdulaziz University, P.O. Box 80203, Jeddah, 21589, Saudi Arabia; lradwan@kau.edu.sa (E.M.R.); aaljaddawi@kau.edu.sa (A.A.A.)
[2] Therapeutic and Protective Proteins Laboratory, Protein Research Department, Genetic Engineering and Biotechnology Research Institute, City for Scientific Research and Technology Applications, New Borg EL-Arab, Alexandria, 21934, Egypt; rredwan@gmail.com
[3] Department of Molecular Medicine and USF Health Byrd Alzheimer's Research Institute, Morsani College of Medicine, University of South Florida, Tampa, FL 33612, USA; vuversky@usf.edu
***** Correspondence: vuversky@usf.edu

Supplementary Figure S1. Amino acid sequences, structural and intrinsic disorder-based features of HCV proteins, human TLRs, and major players of the TLR-regulated downstream signaling pathways. With exception for HCV proteins, information for each protein includes amino acid sequence in FASTA format, disorder profile generated based on the outputs of RIDAO, functional disorder profile generated by D^2P^2, protein-protein interaction network generated by STRING, and a model of 3D structure generated by AlphaFold. For HCV polyprotein and mature individual proteins, amino acid sequences and disorder profile generated based on the outputs of RIDAO are shown.

HCV proteins

```
>sp|P27958|POLG_HCV77 Genome polyprotein OS=Hepatitis C virus genotype 1a (isolate
H77) OX=63746 PE=1 SV=3
MSTNPKPQRKTKRNTNRRPQDVKFPGGGQIVGGVYLLPRRGPRLGVRATRKTSERSQPRGRRQPIPKARRPEGRTWAQPGYPWPL
YGNEGCGWAGWLLSPRGSRPSWGPTDPRRRSRNLGKVIDTLTCGFADLMGYIPLVGAPLGGAARALAHGVRVLEDGVNYATGNLP
GCSFSIFLLALLSCLTVPASAYQVRNSSGLYHVTNDCPNSSVVYEAADAILHTPGCVPCVREGNASRCWVAVTPTVATRDGKLPT
TQLRRHIDLLVGSATLCSALYVGDLCGSVFLVGQLFTFSPRHHWTTQDCNCSIYPGHITGHRMAWNMMMNWSPTAALVVAQLLRI
PQAIMDMIAGAHWGVLAGIKYFSMVGNWAKVLVVLLLFAGVDAETHVTGGNAGRTTAGLVGLLTPGAKQNIQLINTNGSWHINST
ALNCNESLNTGWLAGLFYQHKFNSSGCPERLASCRRLTDFAQGWGPISYANGSGLDERPYCWHYPPRPCGIVPAKSVCGPVYCFT
PSPVVVGTTDRSGAPTYSWGANDTDVFVLNNTRPPLGNWFGCTWMNSTGFTKVCGAPPCVIGGVGNNTLLCPTDCFRKYPEATYS
RCGSGPRITPRCMVDYPYRLWHYPCTINYTIFKVRMYVGGVEHRLEAACNWTRGERCDLEDRDRSELSPLLLSTTQWQVLPCSFT
TLPALSTGLIHLHQNIVDVQYLYGVGSSIASWAIKWEYVVLLFLLLADARVCSCLWMMLLISQAEAALENLVILNAASLAGTHGL
VSFLVFFCFAWYLKGRWVPGAVYALYGMWPLLLLLLALPQRAYALDTEVAASCGGVVLVGLMALTLSPYYKRYISWCMWWLQYFL
TRVEAQLHVWVPPLNVRGGRDAVILLTCVVHPALVFDITKLLLAIFGPLWILQASLLKVPYFVRVQGLLRICALARKIAGGHYVQ
MAIIKLGALTGTCVYNHLAPLRDWAHNGLRDLAVAVEPVVFSRMETKLITWGADTAACGDIINGLPVSARRGQEILLGPADGMVS
KGWRLLAPITAYAQQTRGLLGCIITSLTGRDKNQVEGEVQIVSTATQTFLATCINGVCWTVYHGAGTRTIASPKGPVIQTYTNVD
QDLVGWPAPQGSRSLTPCTCGSSDLYLVTRHADVIPVRRRGDSRGSLLSPRPISYLKGSSGGPLLCPTGHAVGLFRAAVCTRGVA
KAVDFIPVENLETTMRSPVFTDNSSPPAVPQSFQVAHLHAPTGSGKSTKVPAAYAAKGYKVLVLNPSVAATLGFGAYMSKAHGVD
PNIRTGVRTITTGSPITYSTYGKFLADAGCSGGAYDIIICDECHSTDATSISGIGTVLDQAETAGARLVVLATATPPGSVTVSHP
NIEEVALSTTGEIPFYGKAIPLEVIKGGRHLIFCHSKKKCDELAAKLVALGINAVAYYRGLDVSVIPTSGDVVVVSTDALMTGFT
GDFDSVIDCNTCVTQTVDFSLDPTFTIETTTLPQDAVSRTQRRGRTGRGKPGIYRFVAPGERPSGMFDSSVLCECYDAGCAWYEL
TPAETTVRLRAYMNTPGLPVCQDHLGFWEGVFTGLTHIDAHFLSQTKQSGENFPYLVAYQATVCARAQAPPPSWDQMRKCLIRLK
PTLHGPTPLLYRLGAVQNEVTLTHPITKYIMTCMSADLEVVTSTWVLVGGVLAALAAYCLSTGCVVIVGRIVLSGKPAIIPDREV
LYQEFDEMEECSQHLPYIEQGMMLAEQFKQKALGLLQTASRHAEVITPAVQTNWQKLEVFWAKHMWNFISGIQYLAGLSTLPGNP
AIASLMAFTAAVTSPLTTGQTLLFNILGGWVAAQLAAPGAATAFVGAGLAGAALDSVGLGKVLVDILAGYGAGVAGALVAFKIMS
GEVPSTEDLVNLLPAILSPGALAVGVVFASILRRRVGPGEGAVQWMNRLIAFASRGNHVSPTHYVPESDAAARVTAILSSLTVTQ
LLRRLHQWISSECTTPCSGSWLRDIWDWICEVLSDFKTWLKAKLMPQLPGIPFVSCQRGYRGVWRGDGIMHTRCHCGAEITGHVK
NGTMRIVGPRTCKNMWSGTFFINAYTTGPCTPLPAPNYKFALWRVSAEEYVEIRRVGDFHYVSGMTTDNLKCPCQIPSPEFFTEL
DGVRLHRFAPPCKPLLREEVSFRVGLHEYPVGSQLPCEPEPDVAVLTSMLTDPSHITAEAAGRRLARGSPPSMASSSASQLSAPS
LKATCTANHDSPDAELIEANLLWRQEMGGNITRVESENKVVILDSFDPLVAEEDEREVSVPAEILRKSRRFAPALPVWARPDYNP
LLVETWKKPDYEPPVVHGCPLPPPRSPPVPPPRKKRTVVLTESTLPTALAELATKSFGSSSTSGITGDNTTTSSEPAPSGCPPDS
DVESYSSMPPLEGEPGDPDLSDGSWSTVSSGADTEDVVCCSMSYSWTGALVTPCAAEEQKLPINALSNSLLRHHNLVYSTTSRSA
CQRKKKVTFDRLQVLDSHYQDVLKEVKAAASKVKANLLSVEEACSLAPPHSAKSKFGYGAKDVRCHARKAVAHINSVWKDLLEDS
VTPIDTTIMAKNEVFCVQPEKGGRKPARLIVFPDLGVRVCEKMALYDVVSKLPLAVMGSSYGFQYSPGQRVEFLVQAWKSKKTPM
GLSYDTRCFDSTVTESDIRTEEAIYQCCDLDPQARVAIKSLTERLYVGGPLTNSRGENCGYRRCRASRVLTTSCGNTLTRYIKAR
AACRAAGLQDCTMLVCGDDLVVICESAGVQEDAASLRAFTEAMTRYSAPPGDPPQPEYDLELITSCSSNVSVAHDGAGKRVYYLT
RDPTTPLARAAWETARHTPVNSWLGNIIMFAPTLWARMILMTHFFSVLIARDQLEQALNCEIYGACYSIEPLDLPPIIQRLHGLS
AFSLHSYSPGEINRVAACLRKLGVPPLRAWRHRAWSVRARLLARGGKAAICGKYLFNWAVRTKLKLTPITAAGRLDLSGWFTAGY
SGGDIYHSVSHARPRWFWFCLLLLAAGVGIYLLPNR
```

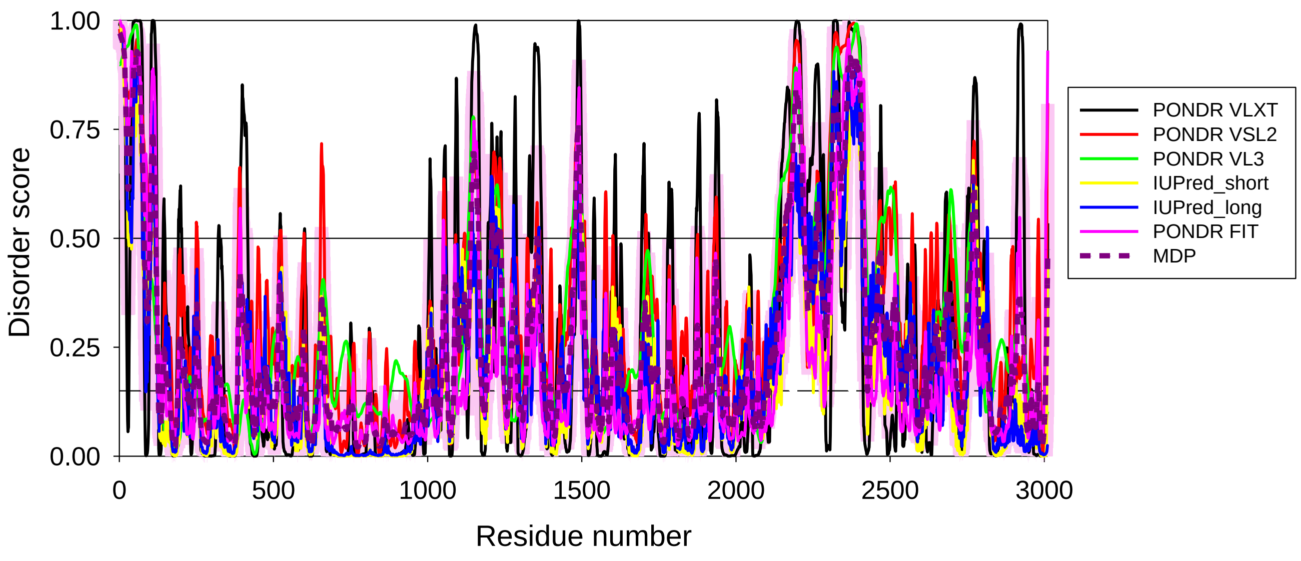

>sp|P27958|2-177 Core (2-177)

STNPKPQRKTKRNTNRRPQDVKFPGGGQIVGGVYLLPRRGPRLGVRATRKTSERSQPRGRRQPIPKARRPEGRTWAQPGYPWPLY
GNEGCGWAGWLLSPRGSRPSWGPTDPRRRSRNLGKVIDTLTCGFADLMGYIPLVGAPLGGAARALAHGVRVLEDGVNYATGNLPG
CSFSIF

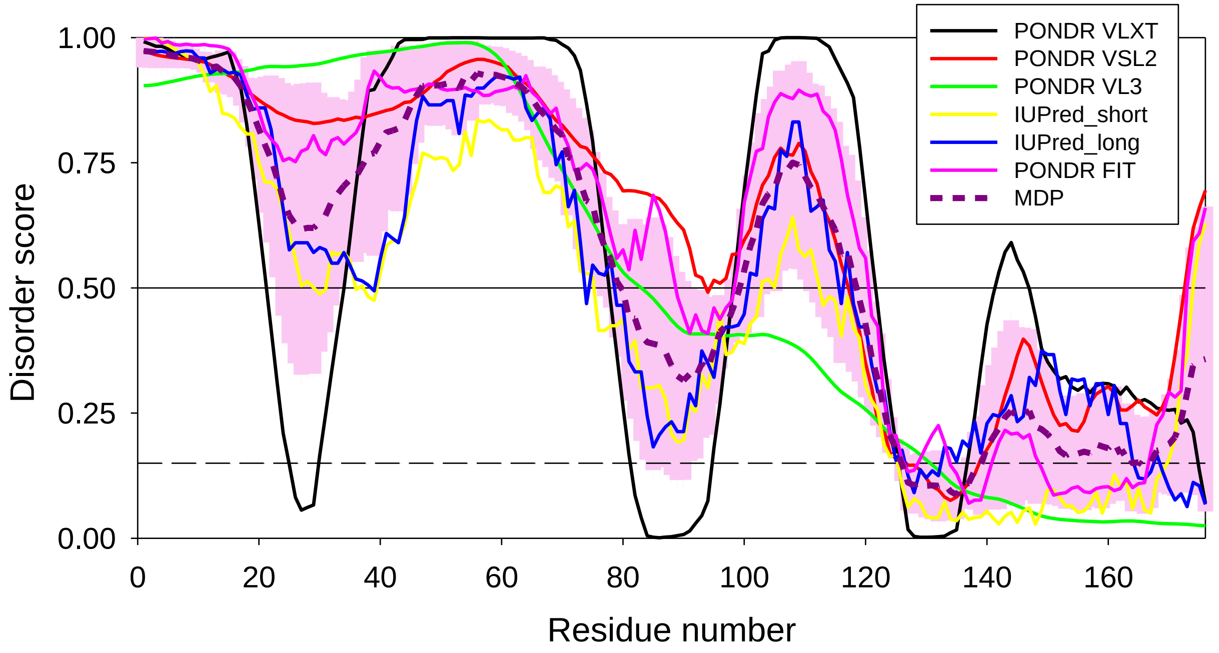

>sp|P27958|192-383 E1 (192-383)

YQVRNSSGLYHVTNDCPNSSVVYEAADAILHTPGCVPCVREGNASRCWVAVTPTVATRDGKLPTTQLRRHIDLLVGSATLCSALY
VGDLCGSVFLVGQLFTFSPRHHWTTQDCNCSIYPGHITGHRMAWNMMMNWSPTAALVVAQLLRIPQAIMDMIAGAHWGVLAGIKY
FSMVGNWAKVLVVLLLFAGVDA

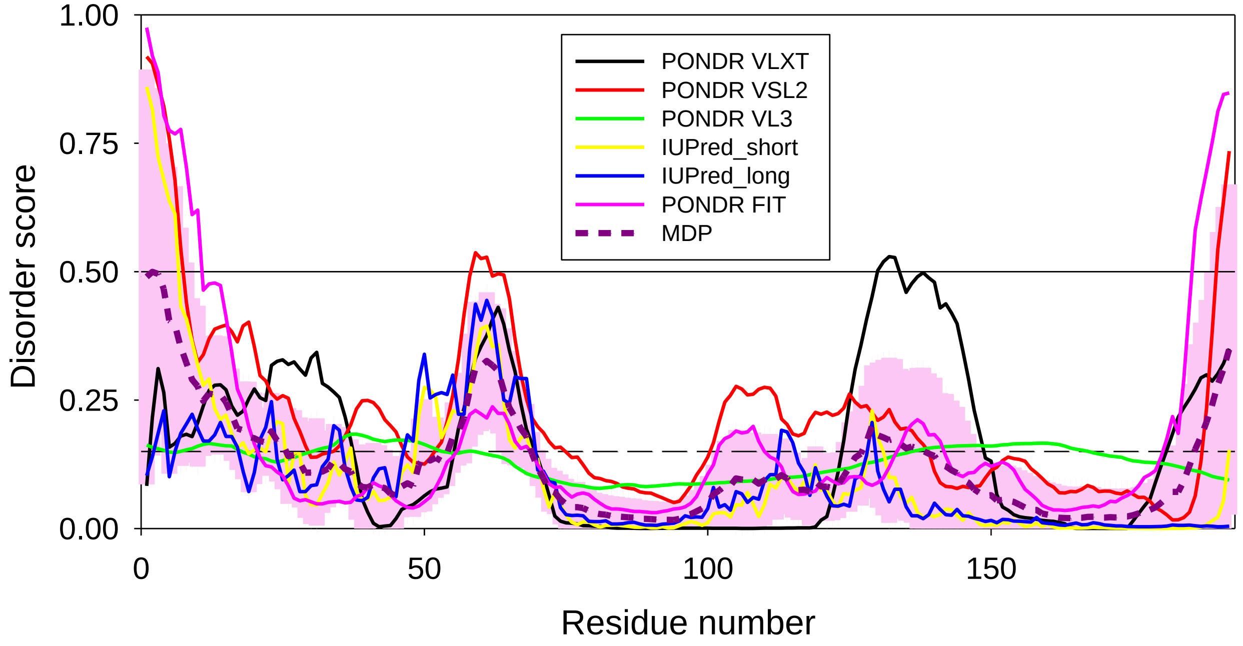

>sp|P27958|384-746 E2 (384-746)

ETHVTGGNAGRTTAGLVGLLTPGAKQNIQLINTNGSWHINSTALNCNESLNTGWLAGLFYQHKFNSSGCPERLASCRRLTDFAQG
WGPISYANGSGLDERPYCWHYPPRPCGIVPAKSVCGPVYCFTPSPVVVGTTDRSGAPTYSWGANDTDVFVLNNTRPPLGNWFGCT
WMNSTGFTKVCGAPPCVIGGVGNNTLLCPTDCFRKYPEATYSRCGSGPRITPRCMVDYPYRLWHYPCTINYTIFKVRMYVGGVEH
RLEAACNWTRGERCDLEDRDRSELSPLLLSTTQWQVLPCSFTTLPALSTGLIHLHQNIVDVQYLYGVGSSIASWAIKWEYVVLLF
LLLADARVCSCLWMMLLISQAEA

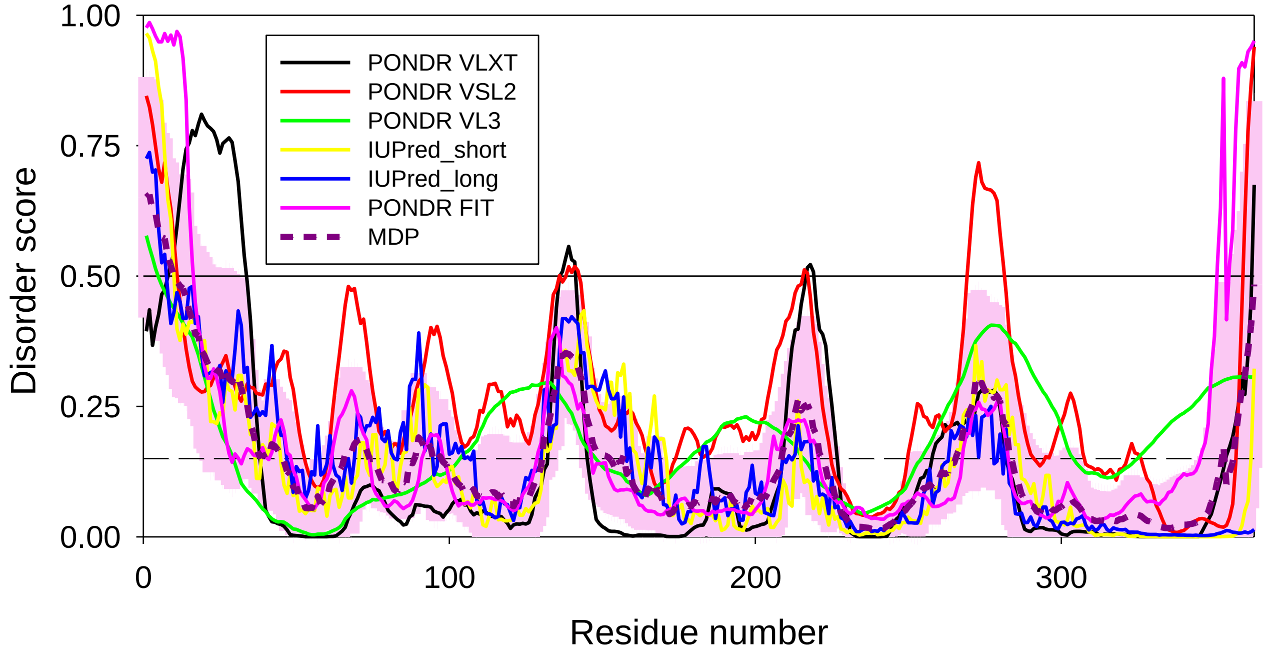

>sp|P27958|747-809 p7 (747-809)

ALENLVILNAASLAGTHGLVSFLVFFCFAWYLKGRWVPGAVYALYGMWPLLLLLLALPQRAYA

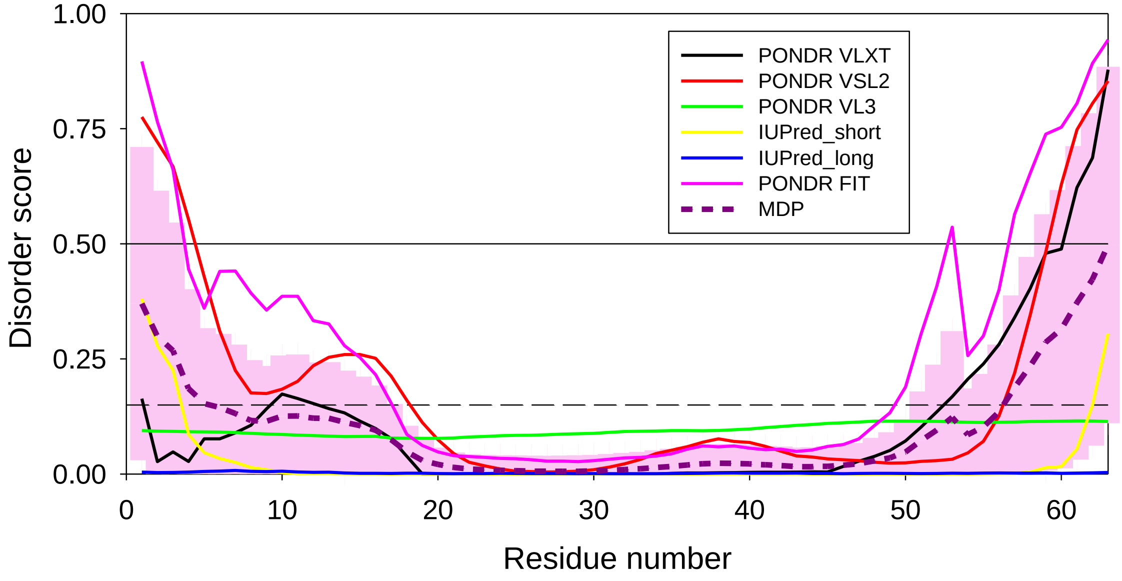

>sp|P27958|810-1026 NS2 (810-1026)

LDTEVAASCGGVVLVGLMALTLSPYYKRYISWCMWWLQYFLTRVEAQLHVWVPPLNVRGGRDAVILLTCVVHPALVFDITKLLLA
IFGPLWILQASLLKVPYFVRVQGLLRICALARKIAGGHYVQMAIIKLGALTGTCVYNHLAPLRDWAHNGLRDLAVAVEPVVFSRM
ETKLITWGADTAACGDIINGLPVSARRGQEILLGPADGMVSKGWRLL

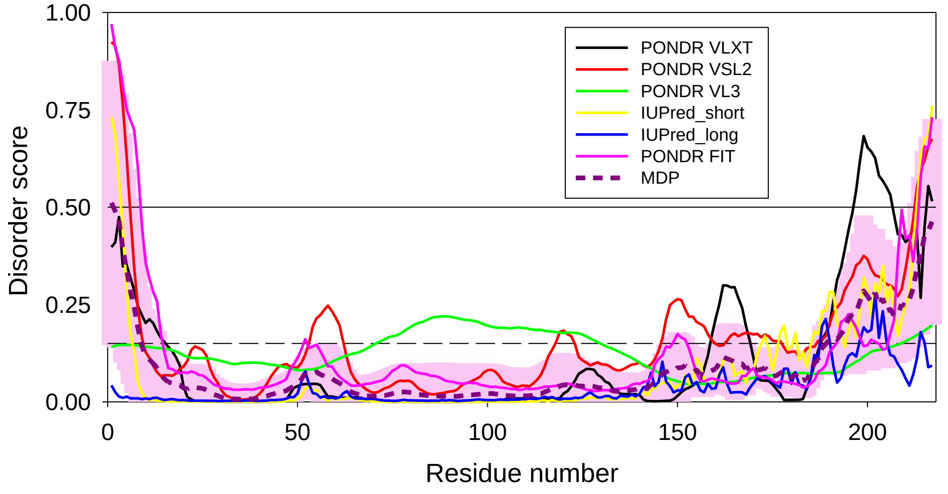

>sp|P27958|1027-1657 NS3 (1027-1657)

APITAYAQQTRGLLGCIITSLTGRDKNQVEGEVQIVSTATQTFLATCINGVCWTVYHGAGTRTIASPKGPVIQTYTNVDQDLVGW
PAPQGSRSLTPCTCGSSDLYLVTRHADVIPVRRRGDSRGSLLSPRPISYLKGSSGGPLLCPTGHAVGLFRAAVCTRGVAKAVDFI
PVENLETTMRSPVFTDNSSPPAVPQSFQVAHLHAPTGSGKSTKVPAAYAAKGYKVLVLNPSVAATLGFGAYMSKAHGVDPNIRTG
VRTITTGSPITYSTYGKFLADAGCSGGAYDIIICDECHSTDATSISGIGTVLDQAETAGARLVVLATATPPGSVTVSHPNIEEVA
LSTTGEIPFYGKAIPLEVIKGGRHLIFCHSKKKCDELAAKLVALGINAVAYYRGLDVSVIPTSGDVVVVSTDALMTGFTGDFDSV
IDCNTCVTQTVDFSLDPTFTIETTTLPQDAVSRTQRRGRTGRGKPGIYRFVAPGERPSGMFDSSVLCECYDAGCAWYELTPAETT
VRLRAYMNTPGLPVCQDHLGFWEGVFTGLTHIDAHFLSQTKQSGENFPYLVAYQATVCARAQAPPPSWDQMRKCLIRLKPTLHGP
TPLLYRLGAVQNEVTLTHPITKYIMTCMSADLEVVT

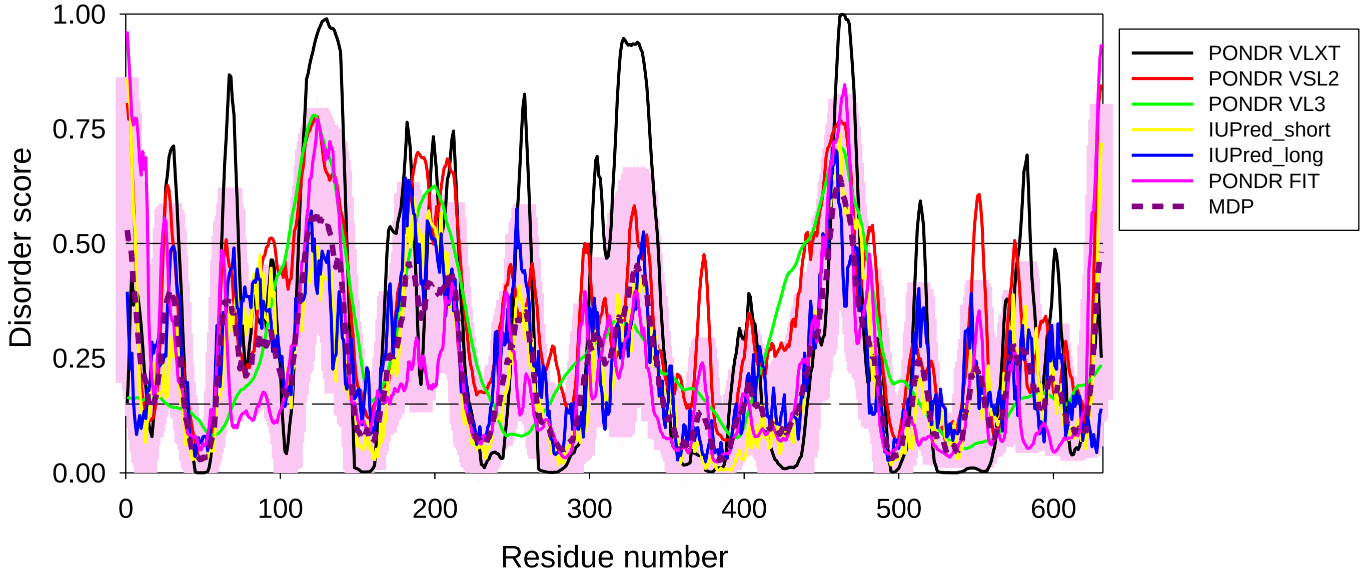

>sp|P27958|1658-1711 NS4A (1658-1711)

STWVLVGGVLAALAAYCLSTGCVVIVGRIVLSGKPAIIPDREVLYQEFDEMEEC

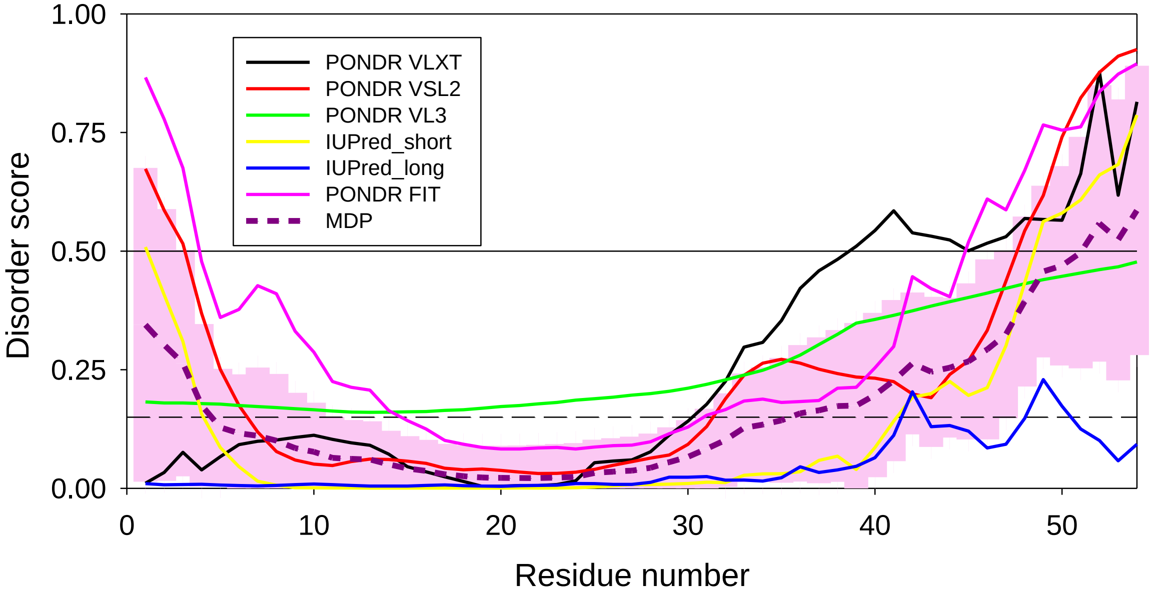

>sp|P27958|1712-1972 NS4B (1712-1972)

SQHLPYIEQGMMLAEQFKQKALGLLQTASRHAEVITPAVQTNWQKLEVFWAKHMWNFISGIQYLAGLSTLPGNPAIASLMAFTAA
VTSPLTTGQTLLFNILGGWVAAQLAAPGAATAFVGAGLAGAALDSVGLGKVLVDILAGYGAGVAGALVAFKIMSGEVPSTEDLVN
LLPAILSPGALAVGVVFASILRRRVGPGEGAVQWMNRLIAFASRGNHVSPTHYVPESDAAARVTAILSSLTVTQLLRRLHQWISS
ECTTPC

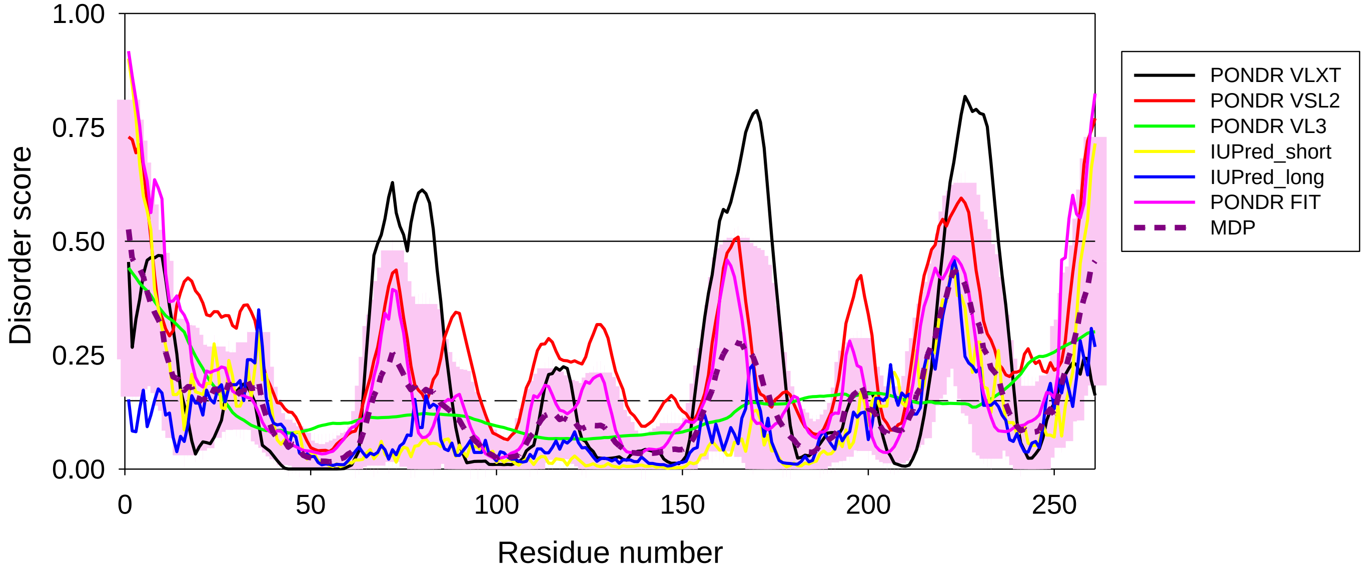

>sp|P27958|1973-2420 NS5A (1973-2420)

SGSWLRDIWDWICEVLSDFKTWLKAKLMPQLPGIPFVSCQRGYRGVWRGDGIMHTRCHCGAEITGHVKNGTMRIVGPRTCKNMWS
GTFFINAYTTGPCTPLPAPNYKFALWRVSAEEYVEIRRVGDFHYVSGMTTDNLKCPCQIPSPEFFTELDGVRLHRFAPPCKPLLR
EEVSFRVGLHEYPVGSQLPCEPEPDVAVLTSMLTDPSHITAEAAGRRLARGSPPSMASSSASQLSAPSLKATCTANHDSPDAELI
EANLLWRQEMGGNITRVESENKVVILDSFDPLVAEEDEREVSVPAEILRKSRRFAPALPVWARPDYNPLLVETWKKPDYEPPVVH
GCPLPPPRSPPVPPPRKKRTVVLTESTLPTALAELATKSFGSSSTSGITGDNTTTSSEPAPSGCPPDSDVESYSSMPPLEGEPGD
PDLSDGSWSTVSSGADTEDVVCC

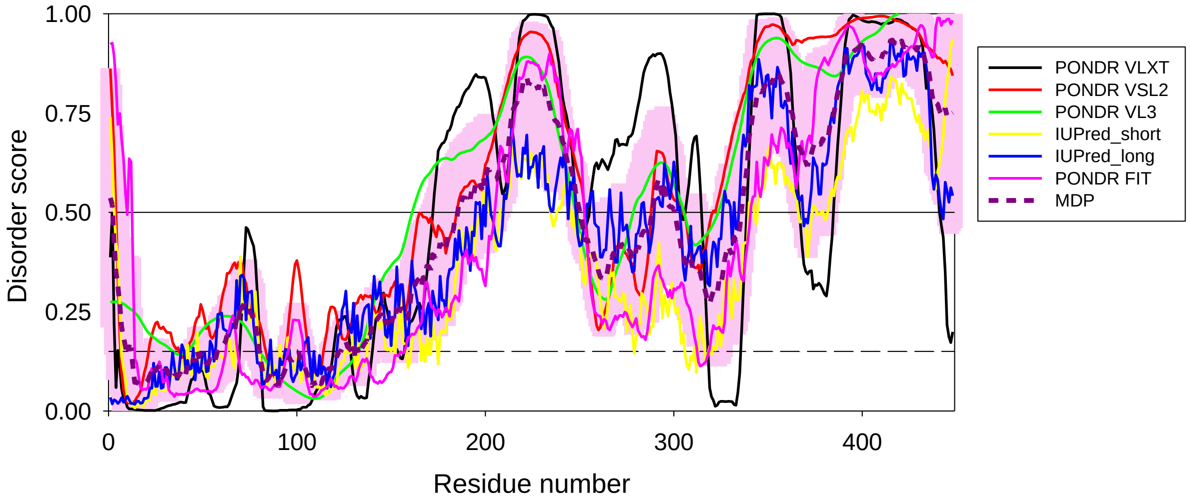

>sp|P27958|2421-3011 NS5B (2421-3011)

SMSYSWTGALVTPCAAEEQKLPINALSNSLLRHHNLVYSTTSRSACQRKKKVTFDRLQVLDSHYQDVLKEVKAAASKVKANLLSV
EEACSLAPPHSAKSKFGYGAKDVRCHARKAVAHINSVWKDLLEDSVTPIDTTIMAKNEVFCVQPEKGGRKPARLIVFPDLGVRVC
EKMALYDVVSKLPLAVMGSSYGFQYSPGQRVEFLVQAWKSKKTPMGLSYDTRCFDSTVTESDIRTEEAIYQCCDLDPQARVAIKS
LTERLYVGGPLTNSRGENCGYRRCRASRVLTTSCGNTLTRYIKARAACRAAGLQDCTMLVCGDDLVVICESAGVQEDAASLRAFT
EAMTRYSAPPGDPPQPEYDLELITSCSSNVSVAHDGAGKRVYYLTRDPTTPLARAAWETARHTPVNSWLGNIIMFAPTLWARMIL
MTHFFSVLIARDQLEQALNCEIYGACYSIEPLDLPPIIQRLHGLSAFSLHSYSPGEINRVAACLRKLGVPPLRAWRHRAWSVRAR
LLARGGKAAICGKYLFNWAVRTKLKLTPITAAGRLDLSGWFTAGYSGGDIYHSVSHARPRWFWFCLLLLAAGVGIYLLPNR

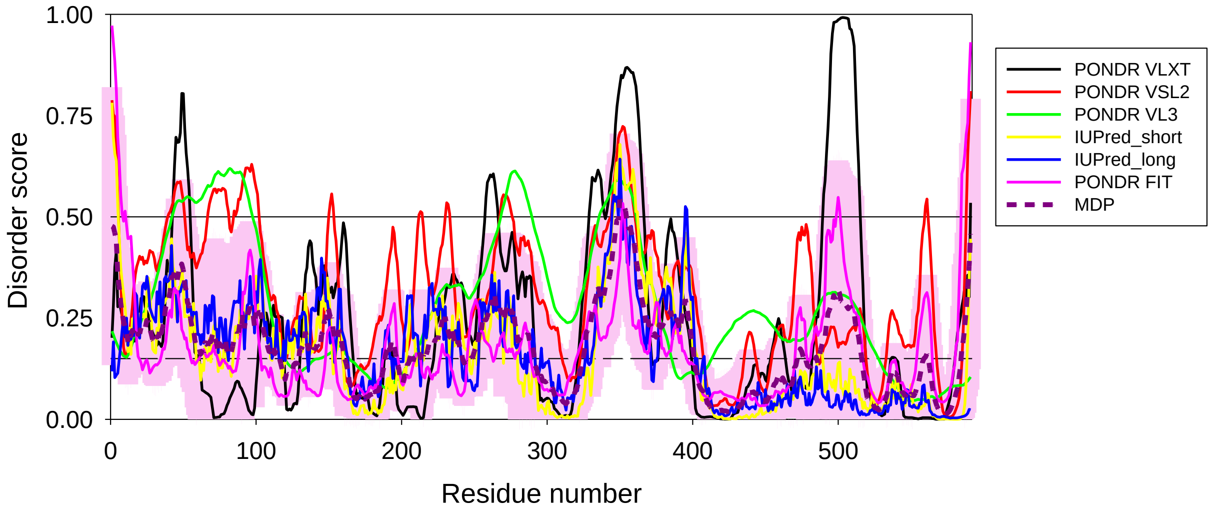

Human TLRs

>sp|Q15399|TLR1_HUMAN Toll-like receptor 1 OS=Homo sapiens OX=9606 GN=TLR1 PE=1 SV=3
MTSIFHFAIIFMLILQIRIQLSEESEFLVDRSKNGLIHVPKDLSQKTTILNISQNYISELWTSDILSLSKLRILIISHNRIQYLD
ISVFKFNQELEYLDLSHNKLVKISCHPTVNLKHLDLSFNAFDALPICKEFGNMSQLKFLGLSTTHLEKSSVLPIAHLNISKVLLV
LGETYGEKEDPEGLQDFNTESLHIVFPTNKEFHFILDVSVKTVANLELSNIKCVLEDNKCSYFLSILAKLQTNPKLSNLTLNNIE
TTWNSFIRILQLVWHTTVWYFSISNVKLQGQLDFRDFDYSGTSLKALSIHQVVSDVFGFPQSYIYEIFSNMNIKNFTVSGTRMVH
MLCPSKISPFLHLDFSNNLLTDTVFENCGHLTELETLILQMNQLKELSKIAEMTTQMKSLQQLDISQNSVSYDEKKGDCSWTKSL
LSLNMSSNILTDTIFRCLPPRIKVLDLHSNKIKSIPKQVVKLEALQELNVAFNSLTDLPGCGSFSSLSVLIIDHNSVSHPSADFF
QSCQKMRSIKAGDNPFQCTCELGEFVKNIDQVSSEVLEGWPDSYKCDYPESYRGTLLKDFHMSELSCNITLLIVTIVATMLVLAV
TVTSLCSYLDLPWYLRMVCQWTQTRRRARNIPLEELQRNLQFHAFISYSGHDSFWVKNELLPNLEKEGMQICLHERNFVPGKSIV
ENIITCIEKSYKSIFVLSPNFVQSEWCHYELYFAHHNLFHEGSNSLILILLEPIPQYSIPSSYHKLKSLMARRTYLEWPKEKSKR
GLFWANLRAAINIKLTEQAKK

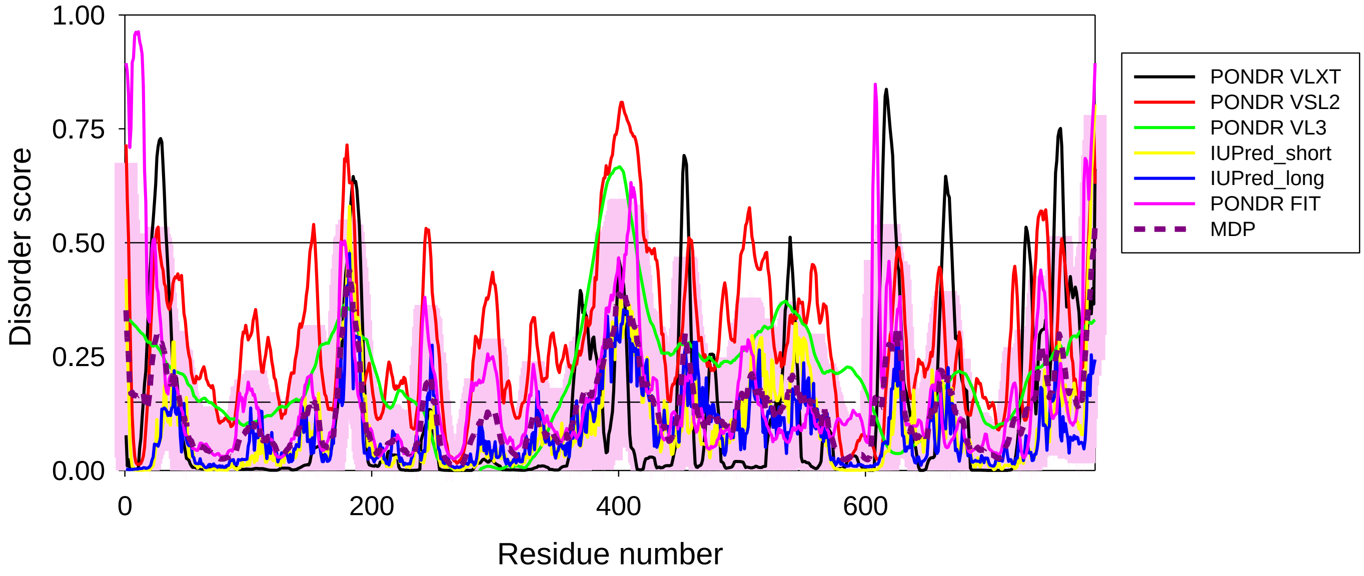

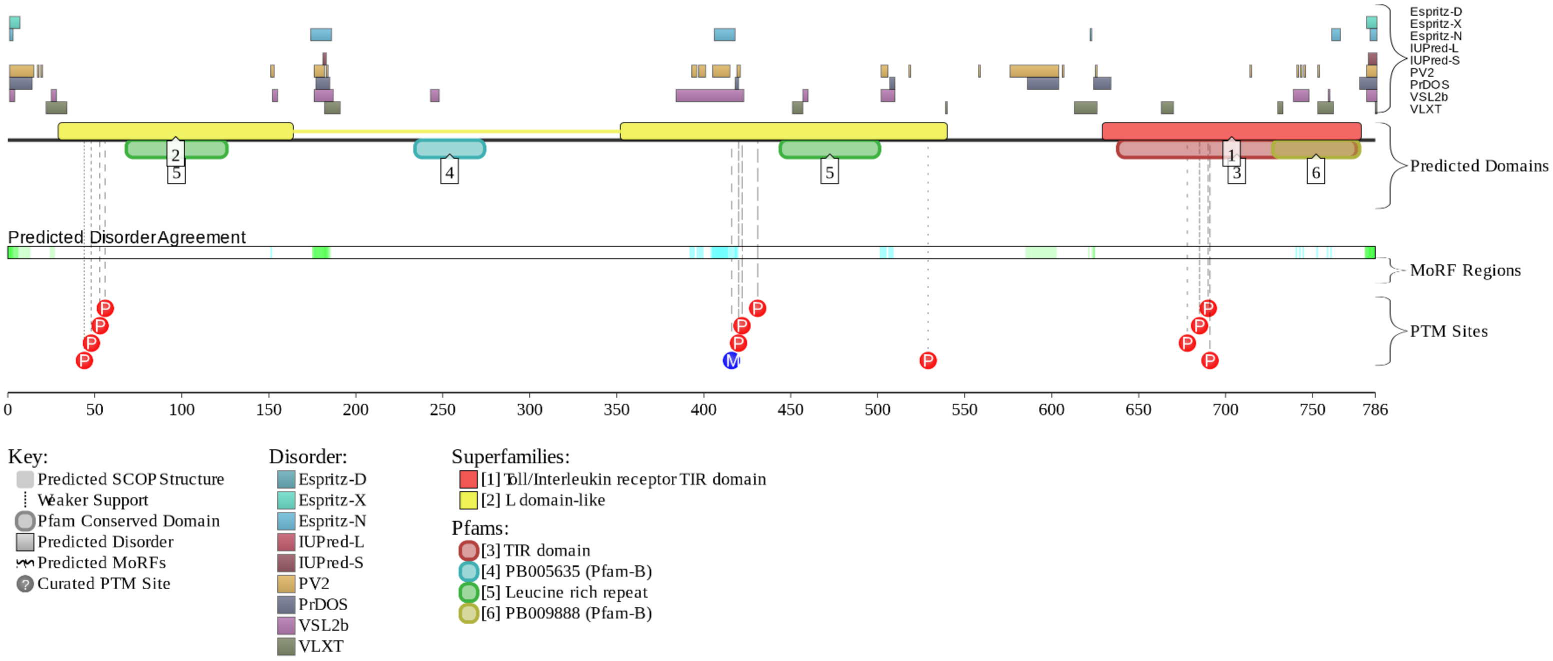

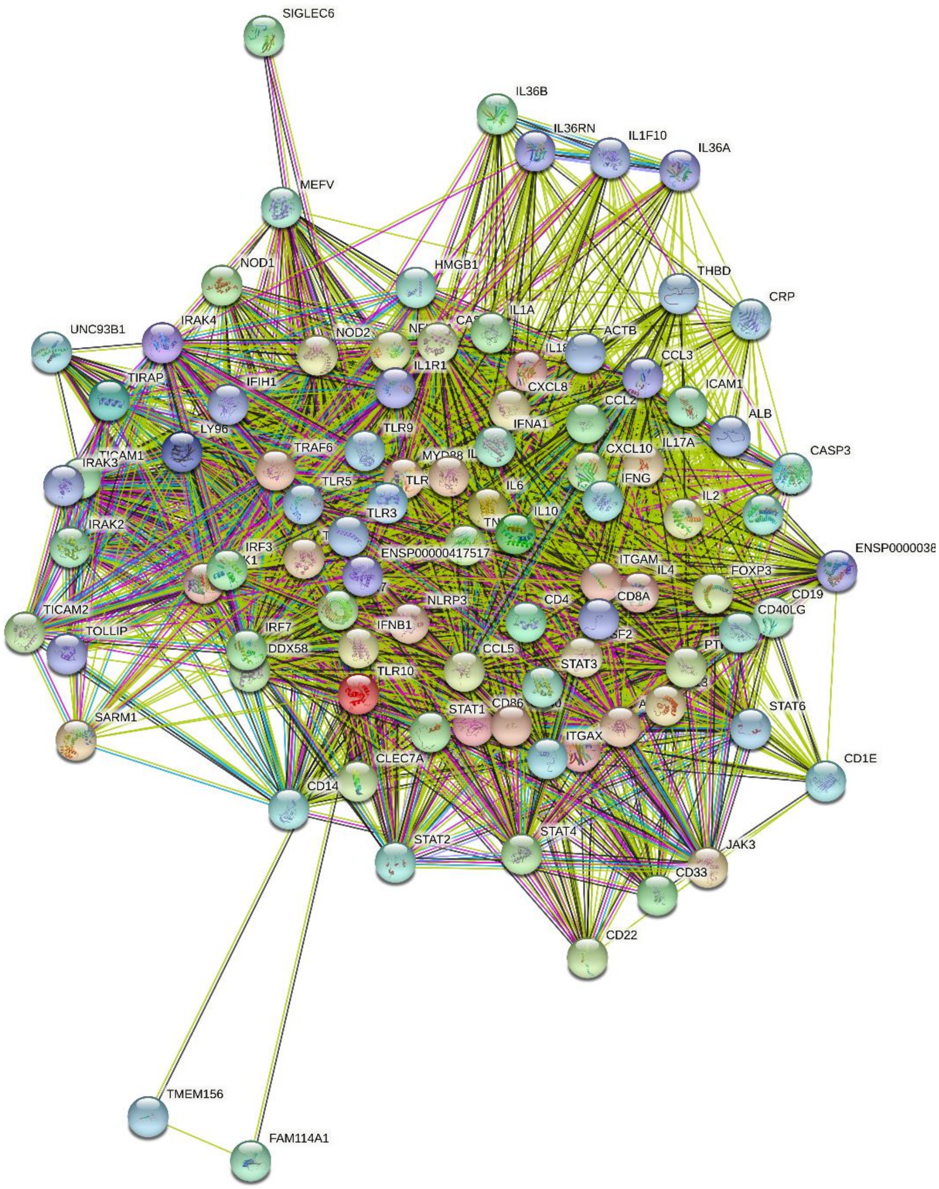
SIGLEC6
IL36B
IL36RN
IL1F10
IL36A
MEFV
NOD1
HMGB1
THBD
CRP
UNC93B1
IRAK4
NOD2
IL1A
ACTB
IL1R1
CCL3
TIRAP
IFIH1
CXCL8
ICAM1
CCL2
ALB
LY96
IFNA1
TICAM1
TRAF6
TLR9
MYD88
CXCL10
IL17A
CASP3
IRAK3
TLR5
IL6
IFNG
IL2
TLR3
IL10
IRAK2
IRF3
ENSP00000417517
ITGAM
IL4
FOXP3
ENSP0000038
CD8A
CD19
TICAM2
NLRP3
CD4
CD40LG
IRF7
IFNB1
TOLLIP
DDX58
CCL5
STAT3
TLR10
STAT6
CD86
STAT1
SARM1
ITGAX
CLEC7A
CD1E
CD14
STAT2
STAT4
JAK3
CD33
CD22
TMEM156
FAM114A1

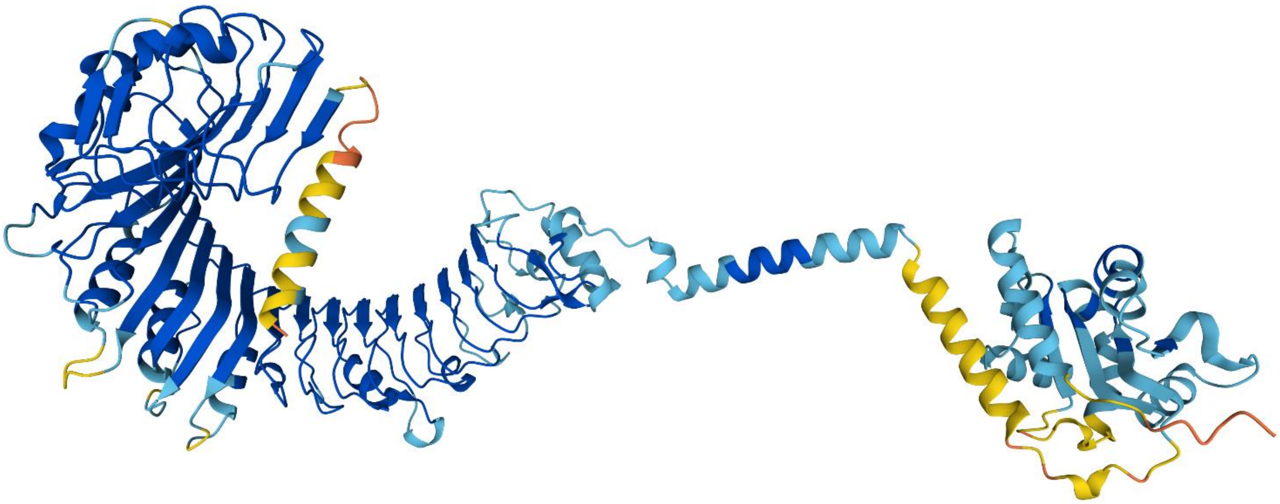

```
>sp|O60603|TLR2_HUMAN Toll-like receptor 2 OS=Homo sapiens OX=9606 GN=TLR2 PE=1 SV=1
MPHTLWMVWVLGVIISLSKEESSNQASLSCDRNGICKGSSGSLNSIPSGLTEAVKSLDLSNNRITYISNSDLQRCVNLQALVLTS
NGINTIEEDSFSSLGSLEHLDLSYNYLSNLSSSWFKPLSSLTFLNLLGNPYKTLGETSLFSHLTKLQILRVGNMDTFTKIQRKDF
AGLTFLEELEIDASDLQSYEPKSLKSIQNVSHLILHMKQHILLLEIFVDVTSSVECLELRDTDLDTFHFSELSTGETNSLIKKFT
FRNVKITDESLFQVMKLLNQISGLLELEFDDCTLNGVGNFRASDNDRVIDPGKVETLTIRRLHIPRFYLFYDLSTLYSLTERVKR
ITVENSKVFLVPCLLSQHLKSLEYLDLSENLMVEEYLKNSACEDAWPSLQTLILRQNHLASLEKTGETLLTLKNLTNIDISKNSF
HSMPETCQWPEKMKYLNLSSTRIHSVTGCIPKTLEILDVSNNNLNLFSLNLPQLKELYISRNKLMTLPDASLLPMLLVLKISRNA
ITTFSKEQLDSFHTLKTLEAGGNNFICSCEFLSFTQEQQALAKVLIDWPANYLCDSPSHVRGQQVQDVRLSVSECHRTALVSGMC
CALFLLILLTGVLCHRFHGLWYMKMMWAWLQAKRKPRKAPSRNICYDAFVSYSERDAYWVENLMVQELENFNPPFKLCLHKRDFI
PGKWIIDNIIDSIEKSHKTVFVLSENFVKSEWCKYELDFSHFRLFDENNDAAILILLEPIEKKAIPQRFCKLRKIMNTKTYLEWP
MDEAQREGFWVNLRAAIKS
```

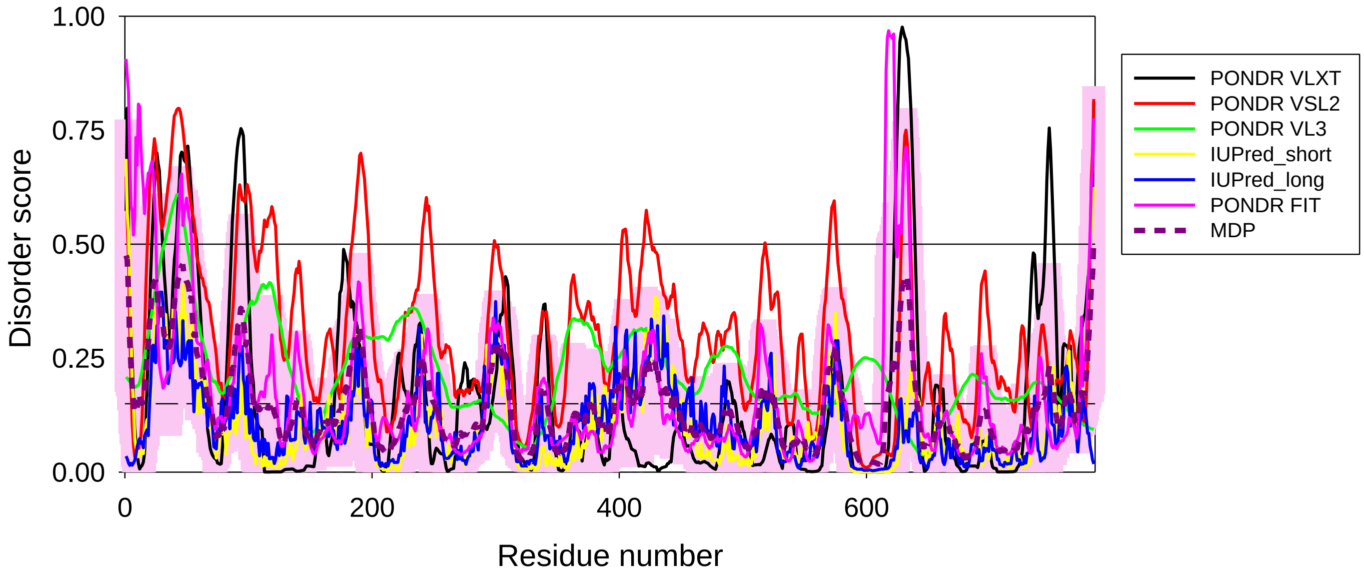

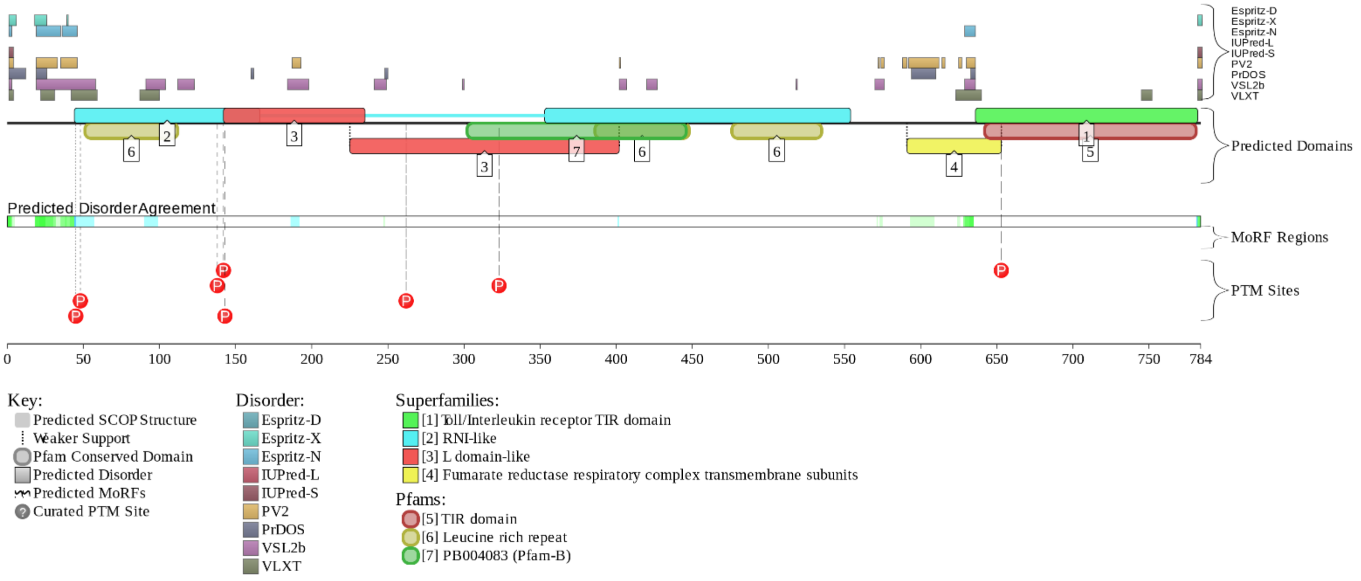

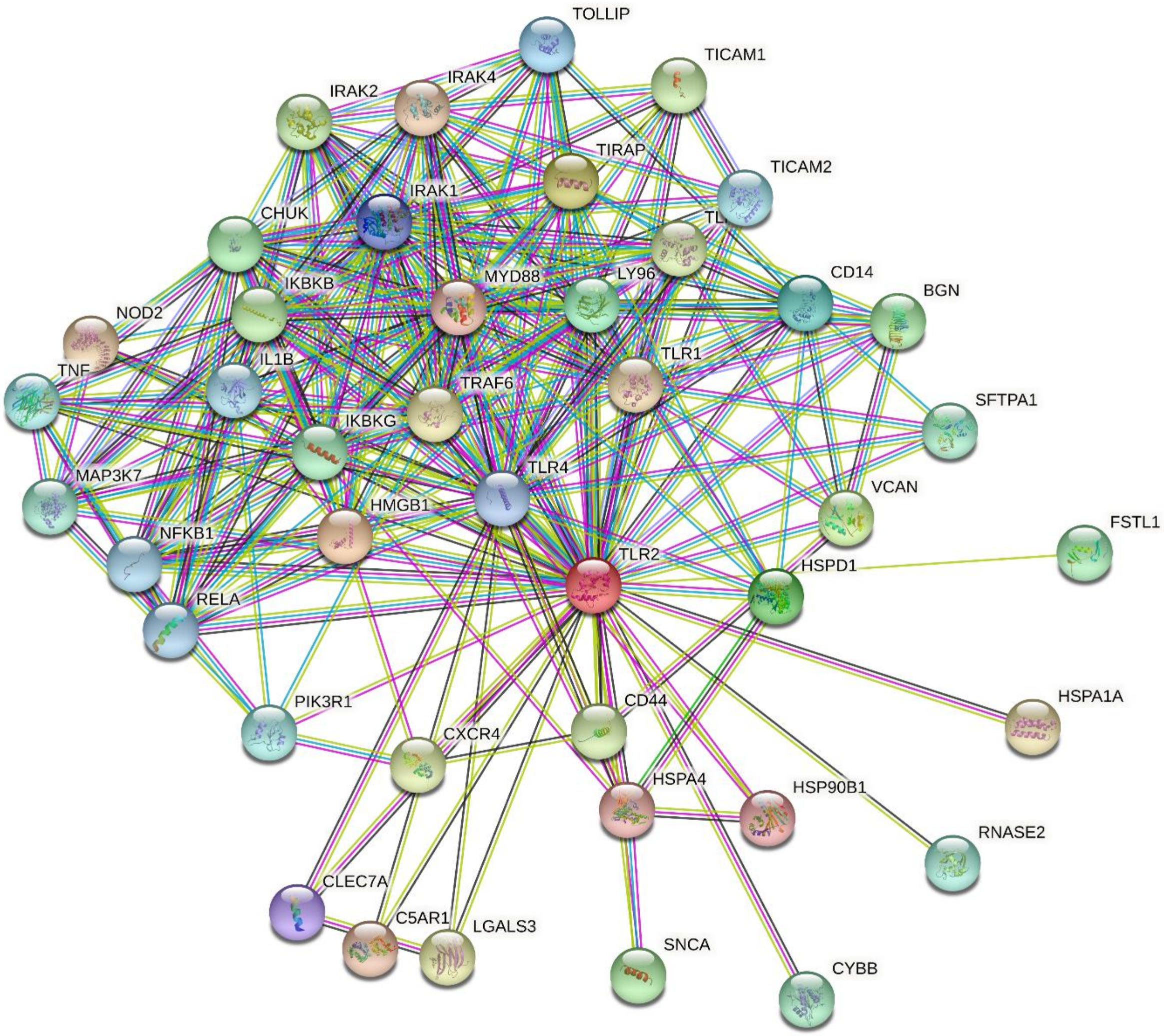
TOLLIP
TICAM1
IRAK2
IRAK4
TIRAP
TICAM2
IRAK1
CHUK
TL
CD14
IKBKB
MYD88
LY96
BGN
NOD2
IL1B
TLR1
TNF
TRAF6
SFTPA1
IKBKG
TLR4
MAP3K7
VCAN
HMGB1
FSTL1
NFKB1
TLR2
HSPD1
RELA
HSPA1A
PIK3R1
CD44
CXCR4
HSPA4
HSP90B1
RNASE2
CLEC7A
C5AR1
LGALS3
SNCA
CYBB

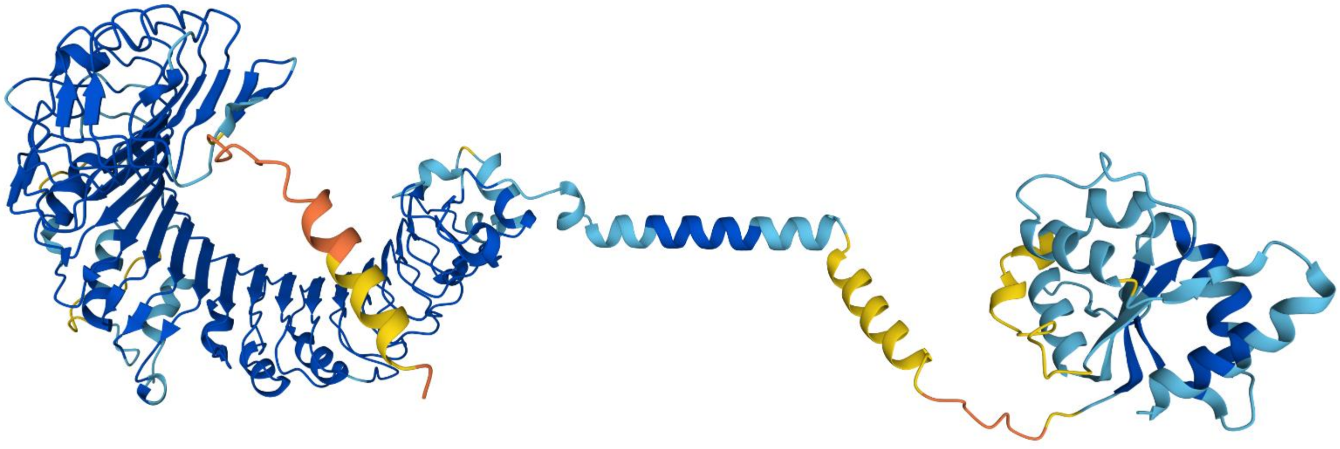

```
>sp|O15455|TLR3_HUMAN Toll-like receptor 3 OS=Homo sapiens OX=9606 GN=TLR3 PE=1 SV=1
MRQTLPCIYFWGGLLPFGMLCASSTTKCTVSHEVADCSHLKLTQVPDDLPTNITVLNLTHNQLRRLPAANFTRYSQLTSLDVGFN
TISKLEPELCQKLPMLKVLNLQHNELSQLSDKTFAFCTNLTELHLMSNSIQKIKNNPFVKQKNLITLDLSHNGLSSTKLGTQVQL
ENLQELLLSNNKIQALKSEELDIFANSSLKKLELSSNQIKEFSPGCFHAIGRLFGLFLNNVQLGPSLTEKLCLELANTSIRNLSL
SNSQLSTTSNTTFLGLKWTNLTMLDLSYNNLNVVGNDSFAWLPQLEYFFLEYNNIQHLFSHSLHGLFNVRYLNLKRSFTKQSISL
ASLPKIDDFSFQWLKCLEHLNMEDNDIPGIKSNMFTGLINLKYLSLSNSFTSLRTLTNETFVSLAHSPLHILNLTKNKISKIESD
AFSWLGHLEVLDLGLNEIGQELTGQEWRGLENIFEIYLSYNKYLQLTRNSFALVPSLQRLMLRRVALKNVDSSPSPFQPLRNLTI
LDLSNNNIANINDDMLEGLEKLEILDLQHNNLARLWKHANPGGPIYFLKGLSHLHILNLESNGFDEIPVEVFKDLFELKIIDLGL
NNLNTLPASVFNNQVSLKSLNLQKNLITSVEKKVFGPAFRNLTELDMRFNPFDCTCESIAWFVNWINETHTNIPELSSHYLCNTP
PHYHGFPVRLFDTSSCKDSAPFELFFMINTSILLIFIFIVLLIHFEGWRISFYWNVSVHRVLGFKEIDRQTEQFEYAAYIIHAYK
DKDWVWEHFSSMEKEDQSLKFCLEERDFEAGVFELEAIVNSIKRSRKIIFVITHHLLKDPLCKRFKVHHAVQQAIEQNLDSIILV
FLEEIPDYKLNHALCLRRGMFKSHCILNWPVQKERIGAFRHKLQVALGSKNSVH
```

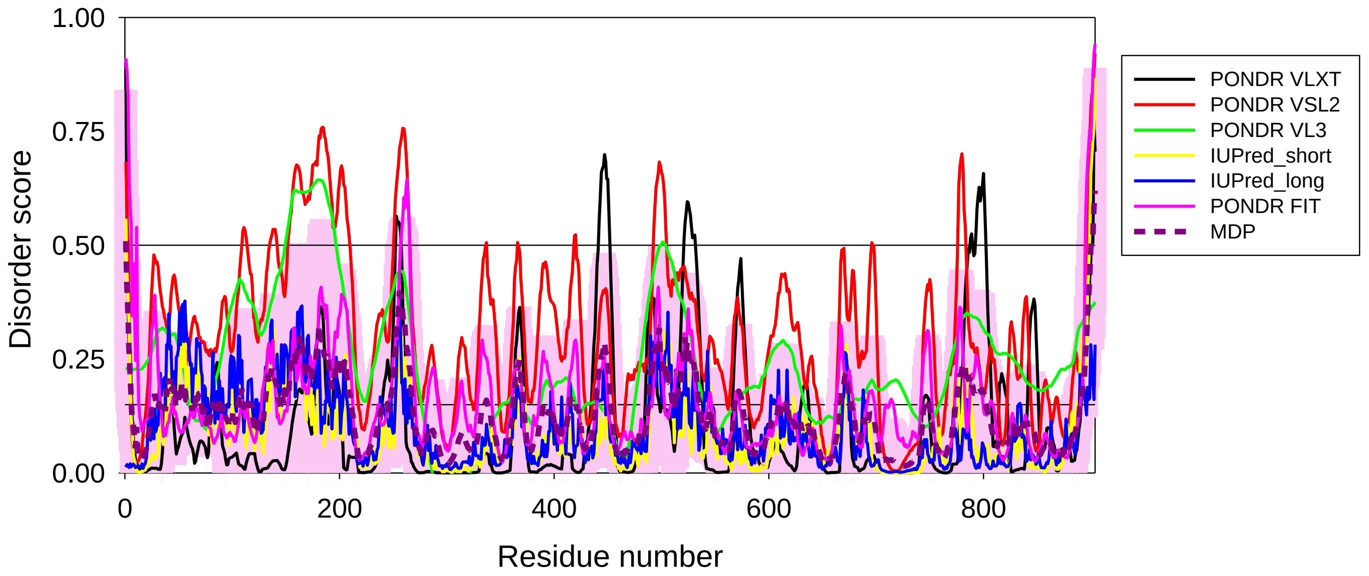

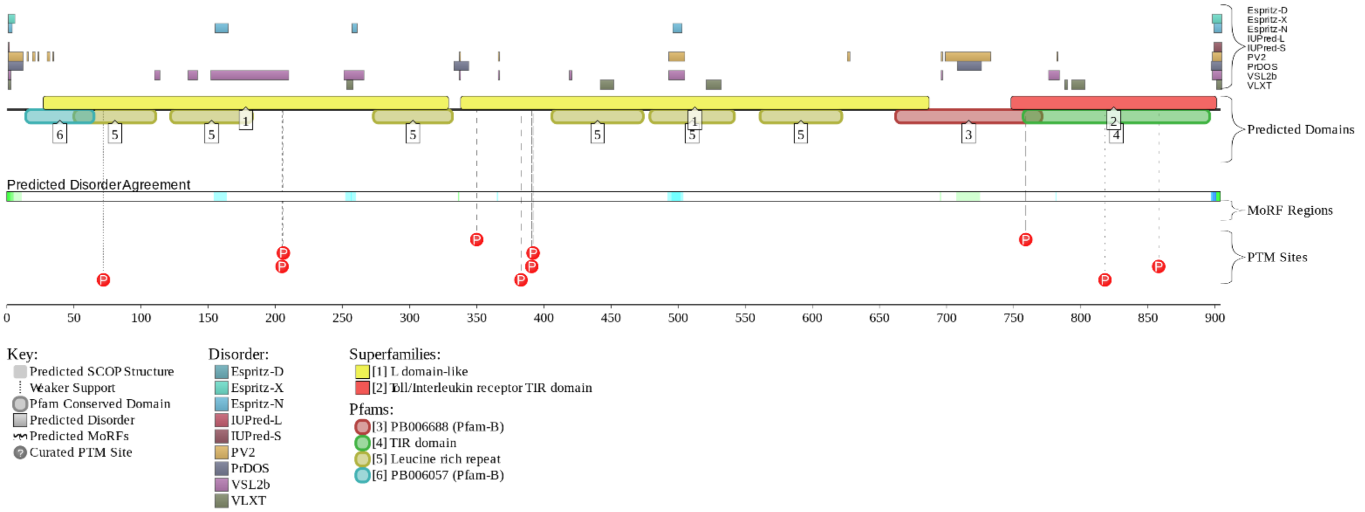

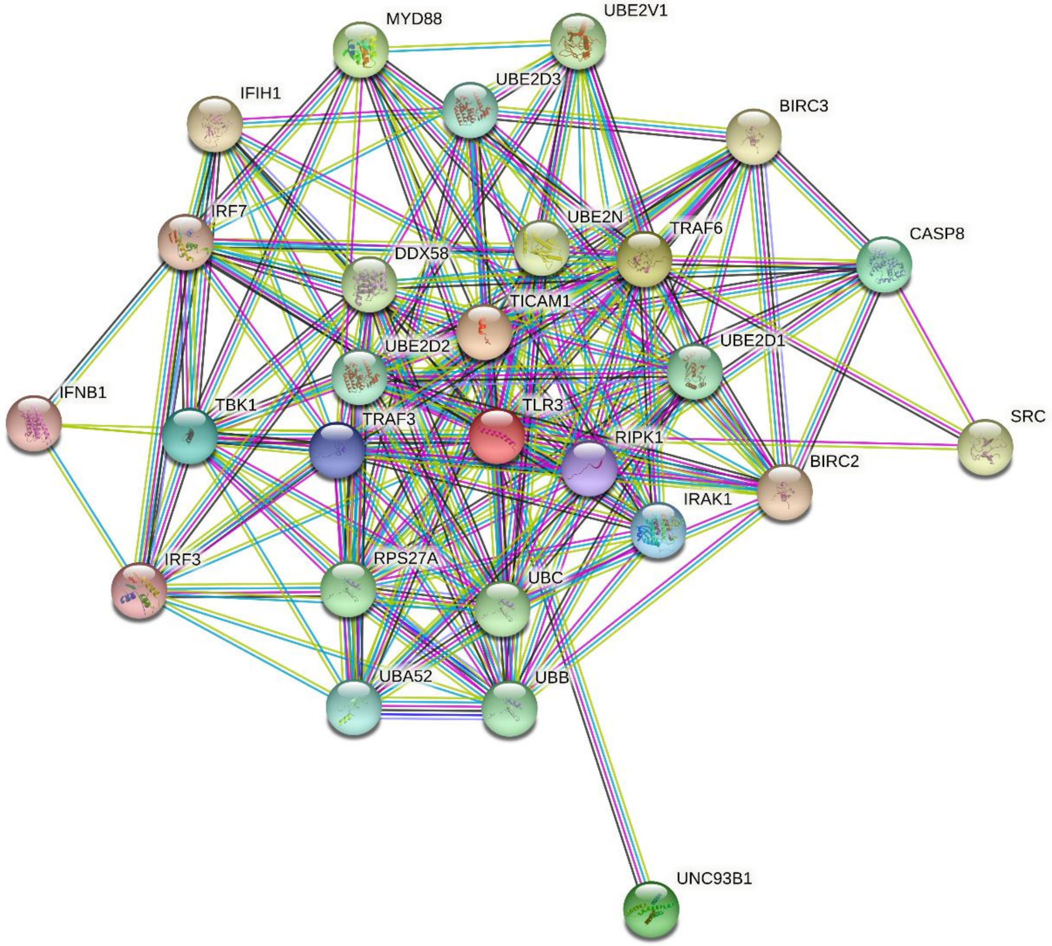
MYD88
UBE2V1
UBE2D3
IFIH1
BIRC3
IRF7
UBE2N
TRAF6
CASP8
DDX58
TICAM1
UBE2D2
UBE2D1
IFNB1
TBK1
TRAF3
TLR3
RIPK1
SRC
BIRC2
IRAK1
IRF3
RPS27A
UBC
UBA52
UBB
UNC93B1

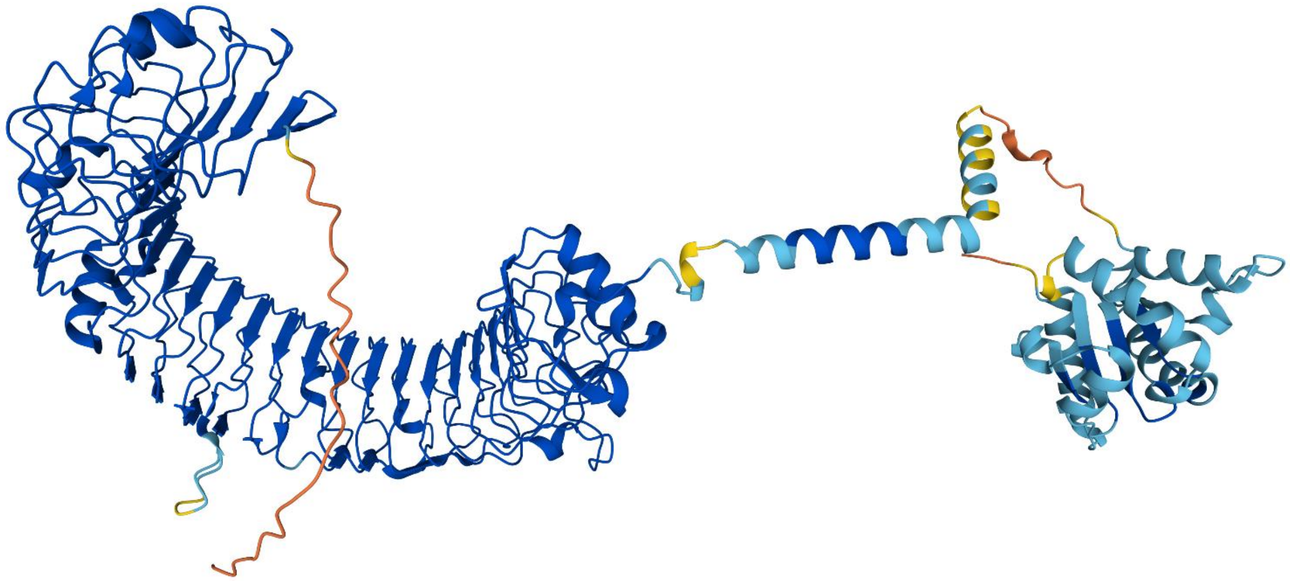

```
>sp|O00206|TLR4_HUMAN Toll-like receptor 4 OS=Homo sapiens OX=9606 GN=TLR4 PE=1 SV=2
MMSASRLAGTLIPAMAFLSCVRPESWEPCVEVVPNITYQCMELNFYKIPDNLPFSTKNLDLSFNPLRHLGSYSFFSFPELQVLDL
SRCEIQTIEDGAYQSLSHLSTLILTGNPIQSLALGAFSGLSSLQKLVAVETNLASLENFPIGHLKTLKELNVAHNLIQSFKLPEY
FSNLTNLEHLDLSSNKIQSIYCTDLRVLHQMPLLNLSLDLSLNPMNFIQPGAFKEIRLHKLTLRNNFDSLNVMKTCIQGLAGLEV
HRLVLGEFRNEGNLEKFDKSALEGLCNLTIEEFRLAYLDYYLDDIIDLFNCLTNVSSFSLVSVTIERVKDFSYNFGWQHLELVNC
KFGQFPTLKLKSLKRLTFTSNKGGNAFSEVDLPSLEFLDLSRNGLSFKGCCSQSDFGTTSLKYLDLSFNGVITMSSNFLGLEQLE
HLDFQHSNLKQMSEFSVFLSLRNLIYLDISHTHTRVAFNGIFNGLSSLEVLKMAGNSFQENFLPDIFTELRNLTFLDLSQCQLEQ
LSPTAFNSLSSLQVLNMSHNNFFSLDTFPYKCLNSLQVLDYSLNHIMTSKKQELQHFPSSLAFLNLTQNDFACTCEHQSFLQWIK
DQRQLLVEVERMECATPSDKQGMPVLSLNITCQMNKTIIGVSVLSVLVVSVVAVLVYKFYFHLMLLAGCIKYGRGENIYDAFVIY
SSQDEDWVRNELVKNLEEGVPPFQLCLHYRDFIPGVAIAANIIHEGFHKSRKVIVVVSQHFIQSRWCIFEYEIAQTWQFLSSRAG
IIFIVLQKVEKTLLRQQVELYRLLSRNTYLEWEDSVLGRHIFWRRLRKALLDGKSWNPEGTVGTGCNWQEATSI
```

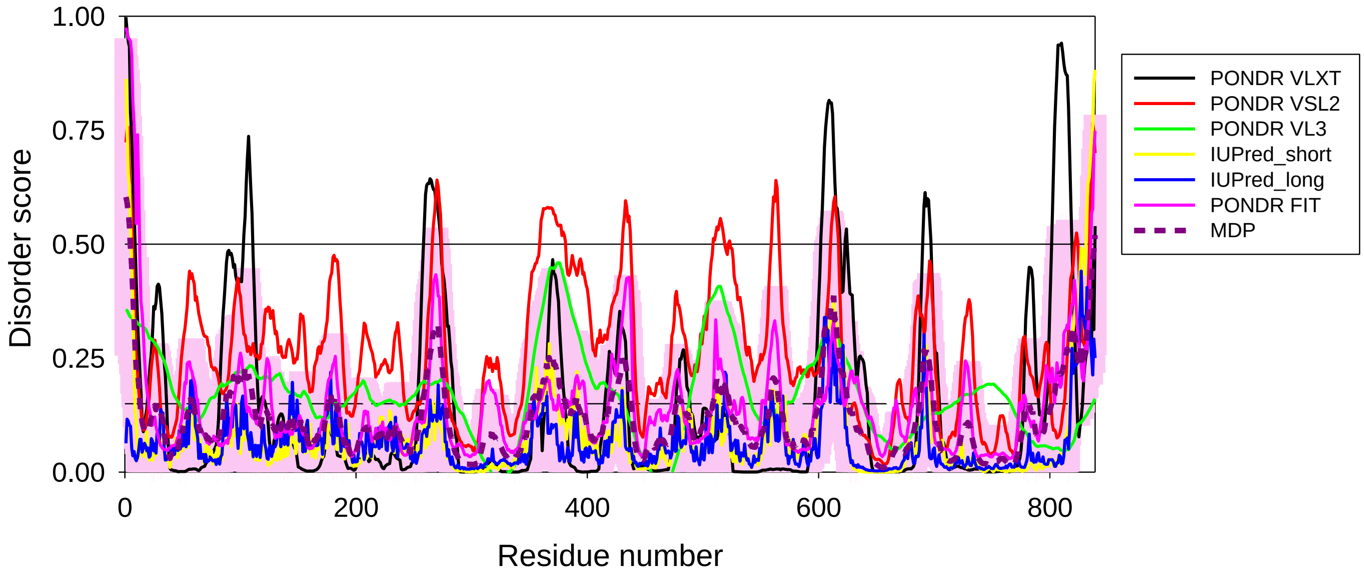

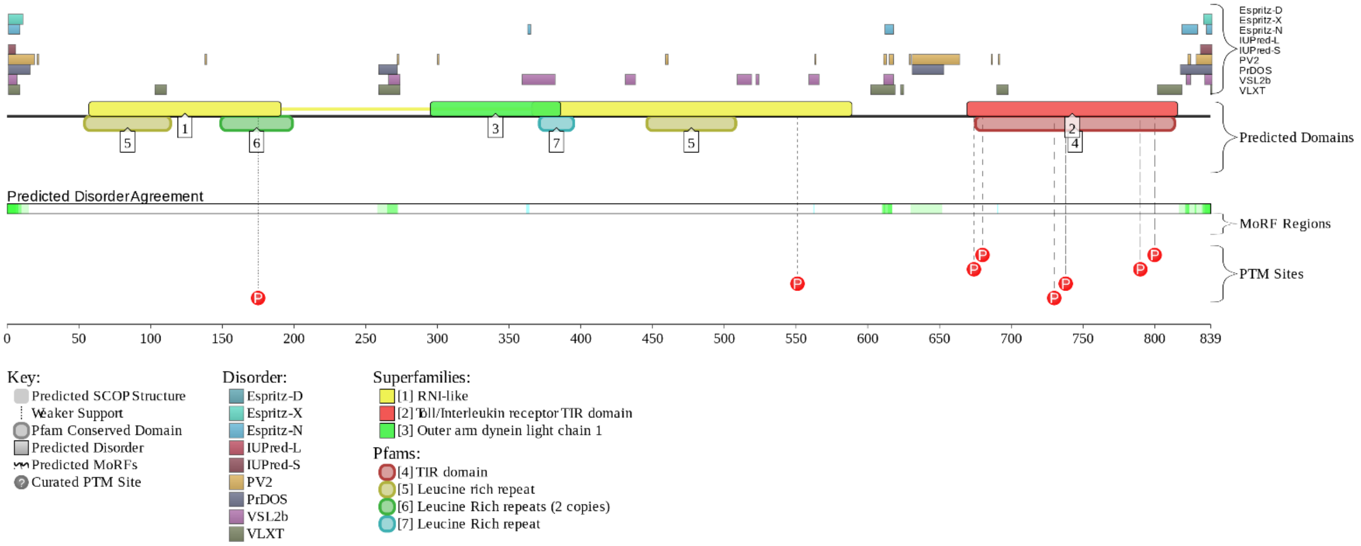

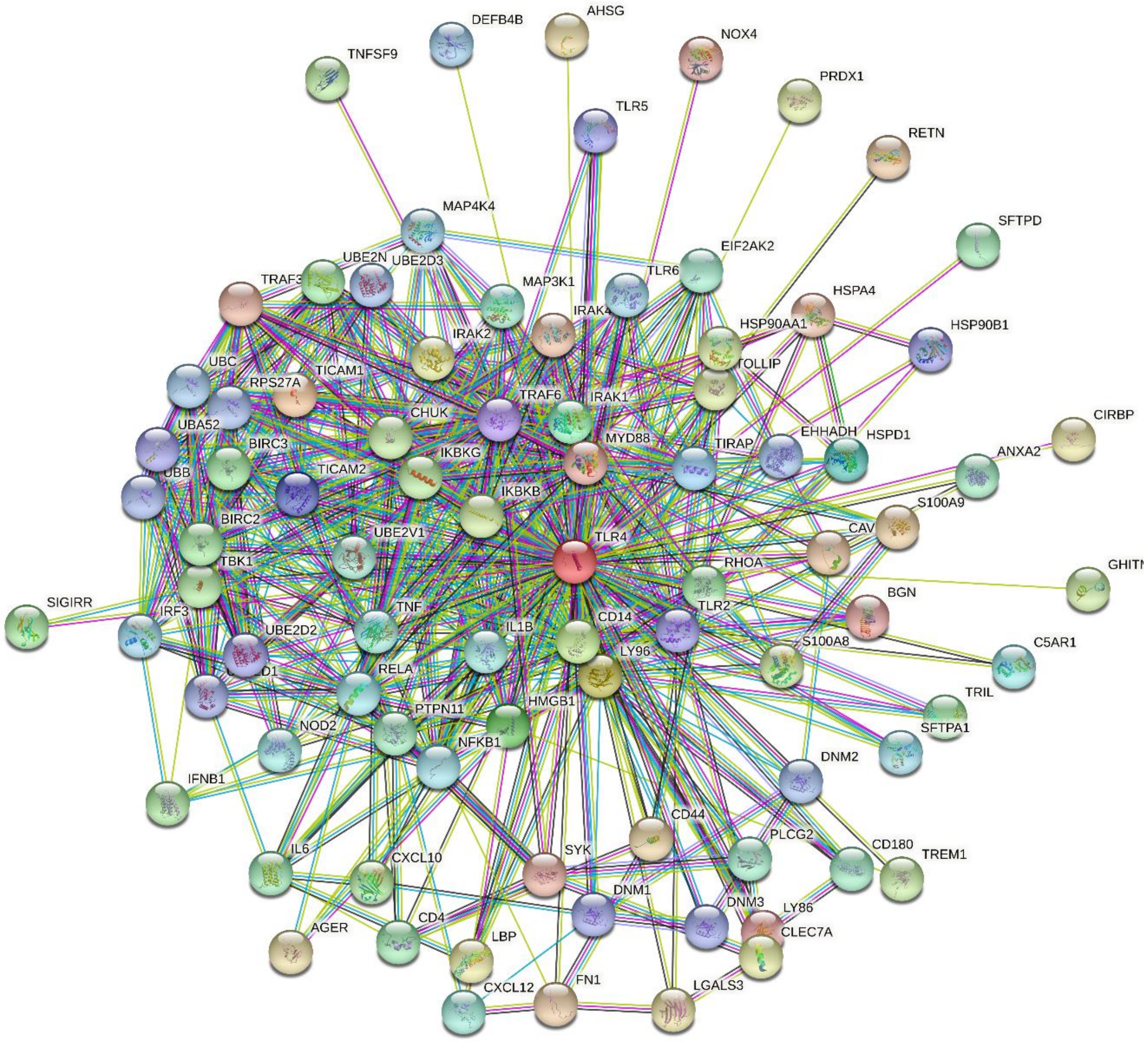
TNFSF9
DEFB4B
AHSG
NOX4
PRDX1
TLR5
RETN
SFTPD
MAP4K4
EIF2AK2
UBE2N
UBE2D3
TRAF3
MAP3K1
TLR6
HSPA4
IRAK4
HSP90AA1
HSP90B1
IRAK2
TOLLIP
UBC
TICAM1
RPS27A
TRAF6
IRAK1
CHUK
CIRBP
EHHADH
HSPD1
UBA52
BIRC3
MYD88
TIRAP
ANXA2
IKBKG
TICAM2
UBB
IKBKB
S100A9
BIRC2
UBE2V1
CAV
TLR4
TBK1
RHOA
BGN
SIGIRR
IRF3
TNF
TLR2
IL1B
CD14
UBE2D2
S100A8
C5AR1
LY96
RELA
TRIL
HMGB1
PTPN11
SFTPA1
NOD2
NFKB1
DNM2
IFNB1
CD44
PLCG2
CD180
TREM1
IL6
SYK
CXCL10
DNM1
DNM3
LY86
AGER
CD4
CLEC7A
LBP
CXCL12
FN1
LGALS3

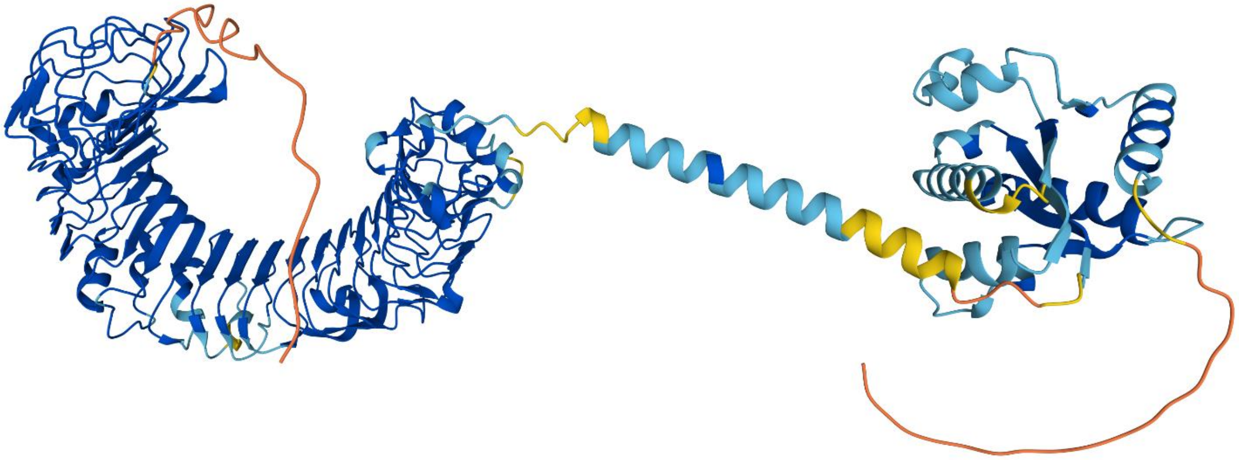

>sp|O60602|TLR5_HUMAN Toll-like receptor 5 OS=Homo sapiens OX=9606 GN=TLR5 PE=1 SV=4
MGDHLDLLLGVVLMAGPVFGIPSCSFDGRIAFYRFCNLTQVPQVLNTTERLLLSFNYIRTVTASSFPFLEQLQLLELGSQYTPLT
IDKEAFRNLPNLRILDLGSSKIYFLHPDAFQGLFHLFELRLYFCGLSDAVLKDGYFRNLKALTRLDLSKNQIRSLYLHPSFGKLN
SLKSIDFSSNQIFLVCEHELEPLQGKTLSFFSLAANSLYSRVSVDWGKCMNPFRNMVLEILDVSGNGWTVDITGNFSNAISKSQA
FSLILAHHIMGAGFGFHNIKDPDQNTFAGLARSSVRHLDLSHGFVFSLNSRVFETLKDLKVLNLAYNKINKIADEAFYGLDNLQV
LNLSYNLLGELYSSNFYGLPKVAYIDLQKNHIAIIQDQTFKFLEKLQTLDLRDNALTTIHFIPSIPDIFLSGNKLVTLPKINLTA
NLIHLSENRLENLDILYFLLRVPHLQILILNQNRFSSCSGDQTPSENPSLEQLFLGENMLQLAWETELCWDVFEGLSHLQVLYLN
HNYLNSLPPGVFSHLTALRGLSLNSNRLTVLSHNDLPANLEILDISRNQLLAPNPDVFVSLSVLDITHNKFICECELSTFINWLN
HTNVTIAGPPADIYCVYPDSFSGVSLFSLSTEGCDEEEVLKSLKFSLFIVCTVTLTLFLMTILTVTKFRGFCFICYKTAQRLVFK
DHPQGTEPDMYKYDAYLCFSSKDFTWVQNALLKHLDTQYSDQNRFNLCFEERDFVPGENRIANIQDAIWNSRKIVCLVSRHFLRD
GWCLEAFSYAQGRCLSDLNSALIMVVVGSLSQYQLMKHQSIRGFVQKQQYLRWPEDFQDVGWFLHKLSQQILKKEKEKKKDNNIP
LQTVATIS

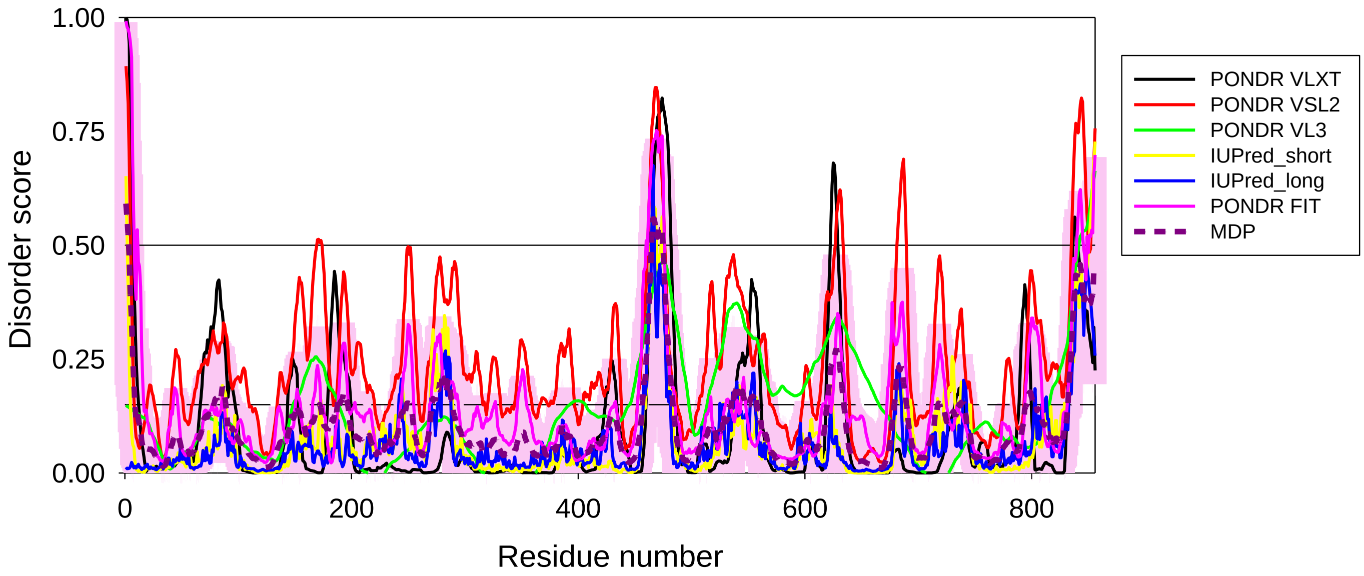

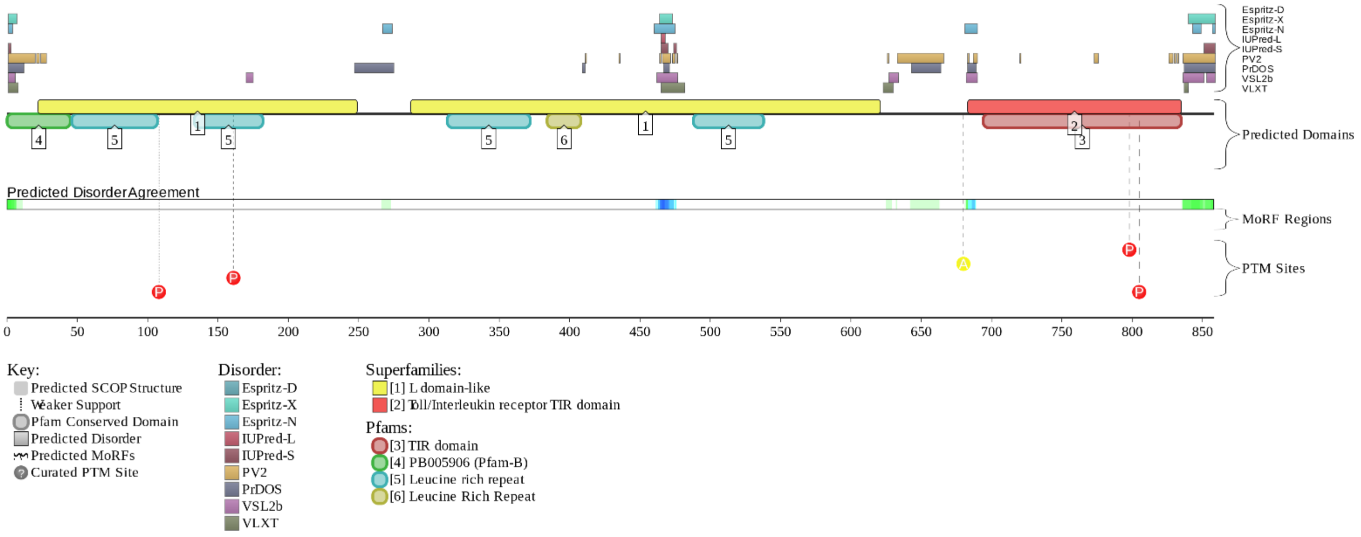

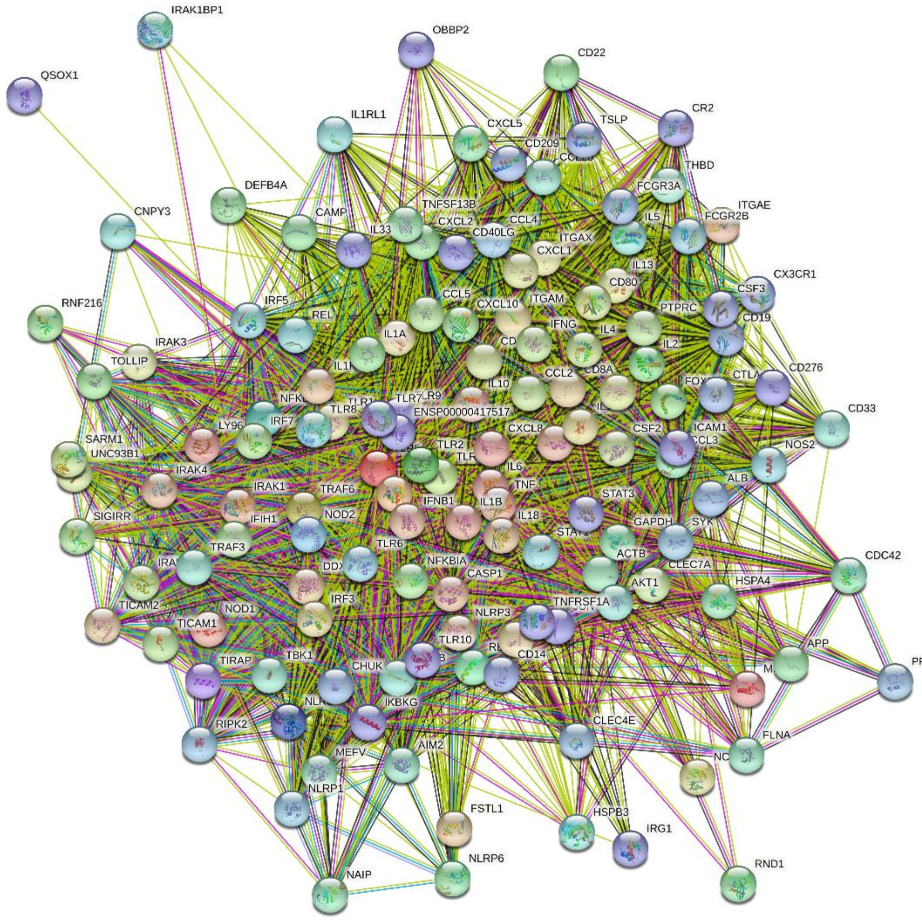
IRAK1BP1
OBBP2
QSOX1
CD22
CR2
TSLP
IL1RL1
CXCL5
CD209
THBD
DEFB4A
FCGR3A
ITGAE
FCGR2B
CNPY3
CAMP
TNFSF13B
IL33
CXCL2
CCL4
CD40LG
IL5
ITGAX
CXCL1
IL13
CX3CR1
CD80
CSF3
RNF216
IRF5
CCL5
CXCL10
ITGAM
PTPRC
REL
IFNG
CD19
IRAK3
IL1A
IL4
IL2
TOLLIP
CD276
CD8A
CCL2
IL10
TLR7
TLR8
IRF7
ENSP00000417517
CD33
LY96
ICAM1
CXCL8
CSF2
SARM1
CCL3
UNC93B1
TLR2
NOS2
IRAK4
ALB
IL6
IRAK1
TNF
TRAF6
STAT3
IFNB1
IL1B
SIGIRR
NOD2
IFIH1
IL18
GAPDH
SYK
TRAF3
ACTB
TLR6
CDC42
NFKBIA
CLEC7A
CASP1
AKT1
HSPA4
TICAM2
IRF3
NOD1
TNFRSF1A
TICAM1
NLRP3
APP
TLR10
PFN1
TIRAP
TBK1
CHUK
CD14
IKBKG
RIPK2
CLEC4E
FLNA
MEFV
AIM2
NLRP1
FSTL1
HSPB3
IRG1
NLRP6
NAIP
RND1

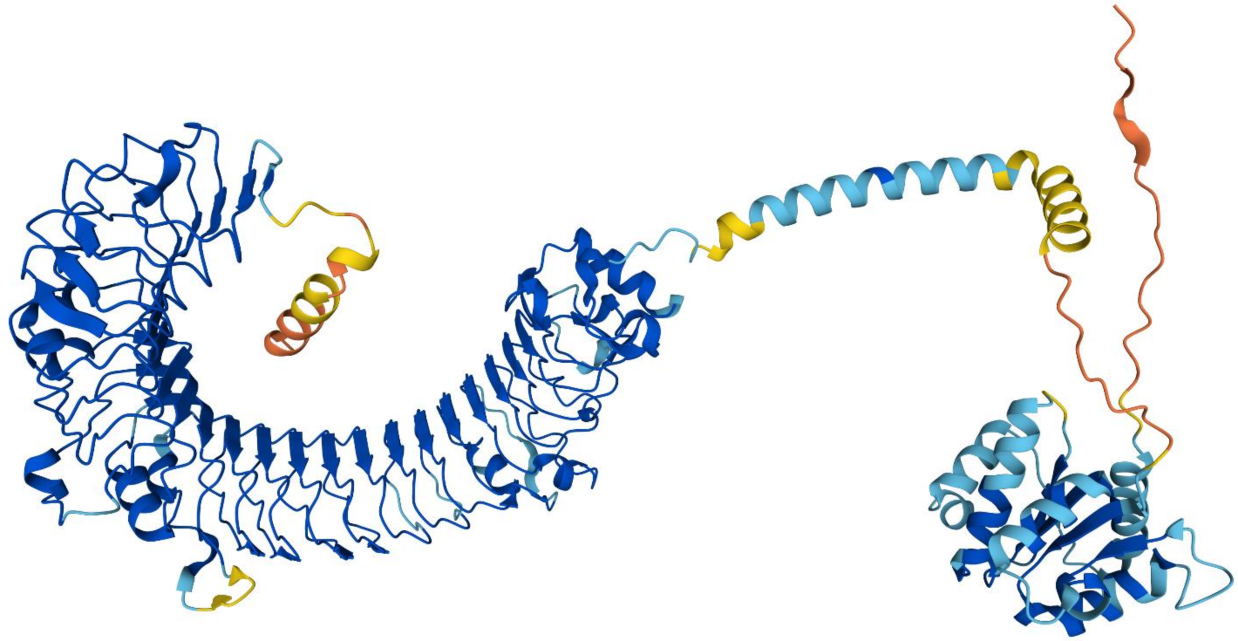

>sp|Q9Y2C9|TLR6_HUMAN Toll-like receptor 6 OS=Homo sapiens OX=9606 GN=TLR6 PE=1 SV=2
MTKDKEPIVKSFHFVCLMIIIVGTRIQFSDGNEFAVDKSKRGLIHVPKDLPLKTKVLDMSQNYIAELQVSDMSFLSELTVLRLSH
NRIQLLDLSVFKFNQDLEYLDLSHNQLQKISCHPIVSFRHLDLSFNDFKALPICKEFGNLSQLNFLGLSAMKLQKLDLLPIAHLH
LSYILLDLRNYYIKENETESLQILNAKTLHLVFHPTSLFAIQVNISVNTLGCLQLTNIKLNDDNCQVFIKFLSELTRGSTLLNFT
LNHIETTWKCLVRVFQFLWPKPVEYLNIYNLTIIESIREEDFTYSKTTLKALTIEHITNQVFLFSQTALYTVFSEMNIMMLTISD
TPFIHMLCPHAPSTFKFLNFTQNVFTDSIFEKCSTLVKLETLILQKNGLKDLFKVGLMTKDMPSLEILDVSWNSLESGRHKENCT
WVESIVVLNLSSNMLTDSVFRCLPPRIKVLDLHSNKIKSVPKQVVKLEALQELNVAFNSLTDLPGCGSFSSLSVLIIDHNSVSHP
SADFFQSCQKMRSIKAGDNPFQCTCELREFVKNIDQVSSEVLEGWPDSYKCDYPESYRGSPLKDFHMSELSCNITLLIVTIGATM
LVLAVTVTSLCIYLDLPWYLRMVCQWTQTRRRARNIPLEELQRNLQFHAFISYSEHDSAWVKSELVPYLEKEDIQICLHERNFVP
GKSIVENIINCIEKSYKSIFVLSPNFVQSEWCHYELYFAHHNLFHEGSNNLILILLEPIPQNSIPNKYHKLKALMTQRTYLQWPK
EKSKRGLFWANIRAAFNMKLTLVTENNDVKS

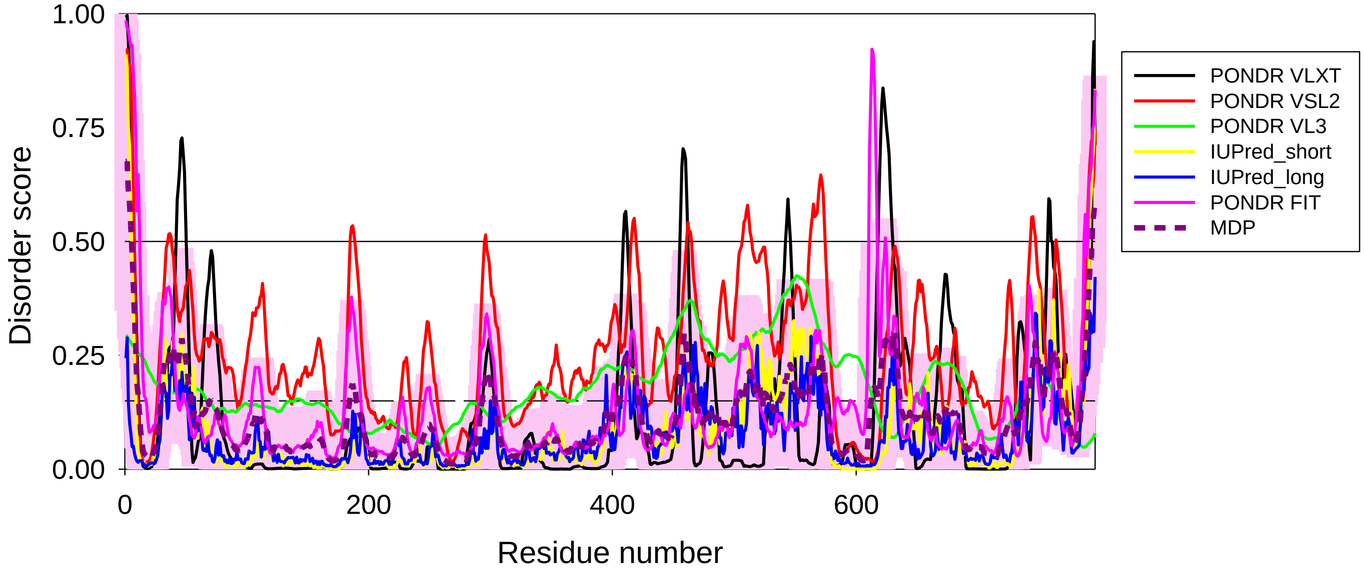

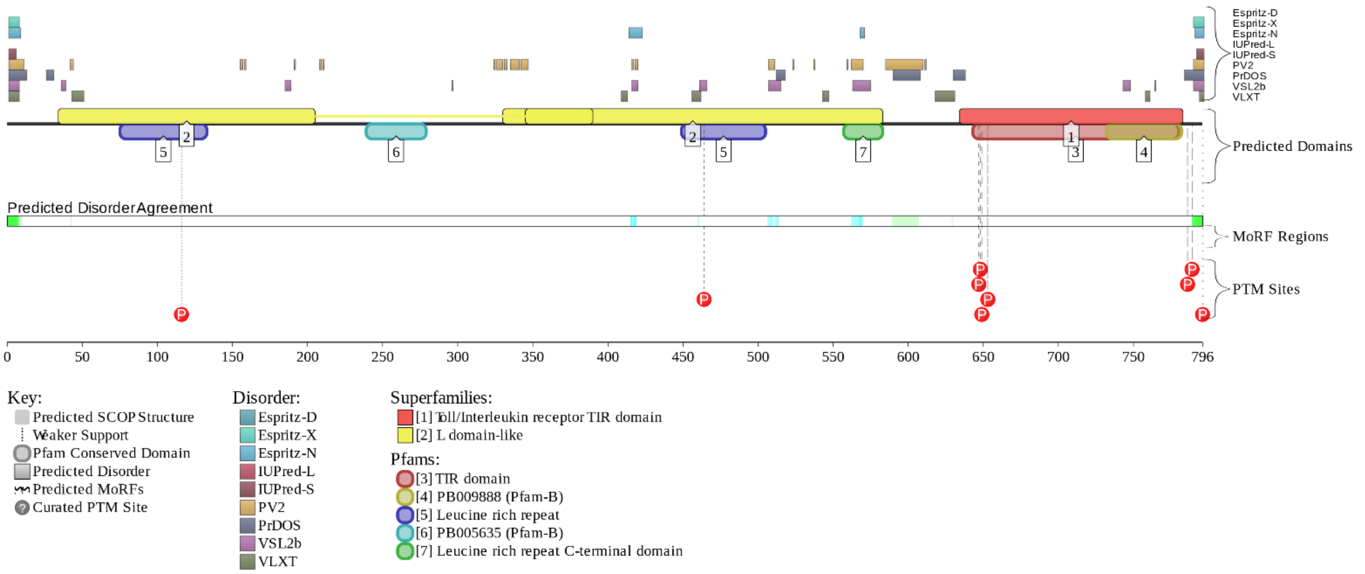

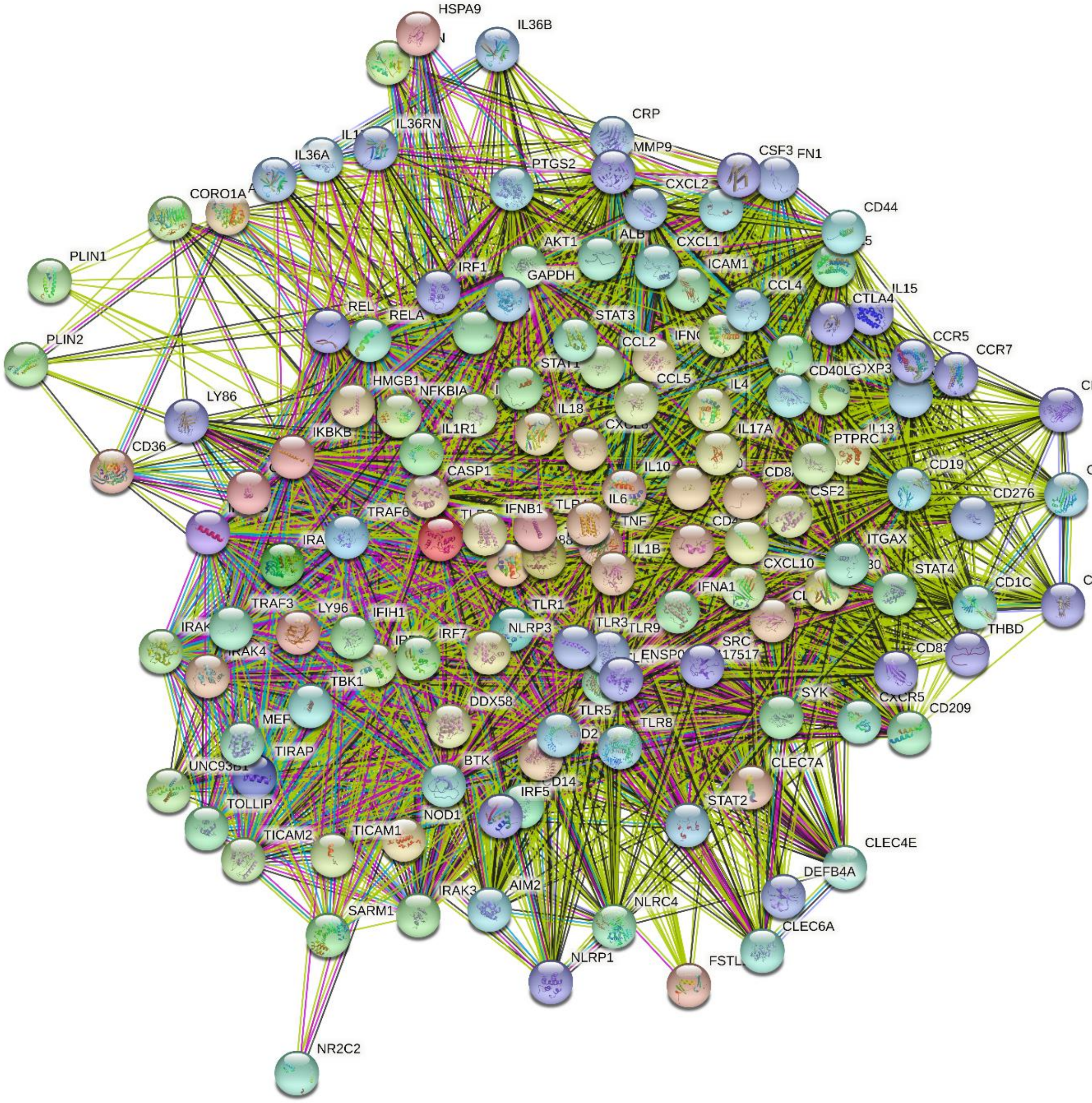
HSPA9
IL36B
CRP
IL36RN
MMP9
CSF3
FN1
IL36A
PTGS2
CXCL2
CORO1A
CD44
AKT1
ALB
CXCL1
PLIN1
IRF1
GAPDH
ICAM1
CCL4
IL15
CTLA4
REL
RELA
STAT3
CCL2
CCR5
CCR7
PLIN2
STAT1
CD40LG
HMGB1
NFKBIA
CCL5
IL4
CD1E
LY86
IL18
IKBKB
IL1R1
IL17A
IL13
PTPRC
CD36
CASP1
IL10
CD19
CD1B
CSF2
CD276
IL6
TRAF6
IFNB1
TNF
IL1B
ITGAX
STAT4
CXCL10
CD1C
CD1A
IFNA1
TRAF3
LY96
IFIH1
TLR1
THBD
NLRP3
TLR3
TLR9
IRF7
SRC
IRAK4
TBK1
SYK
CXCR5
CD209
DDX58
TLR5
TLR8
TIRAP
BTK
CLEC7A
IRF5
TOLLIP
STAT2
NOD1
TICAM2
TICAM1
CLEC4E
DEFB4A
IRAK3
AIM2
SARM1
NLRC4
CLEC6A
NLRP1
NR2C2

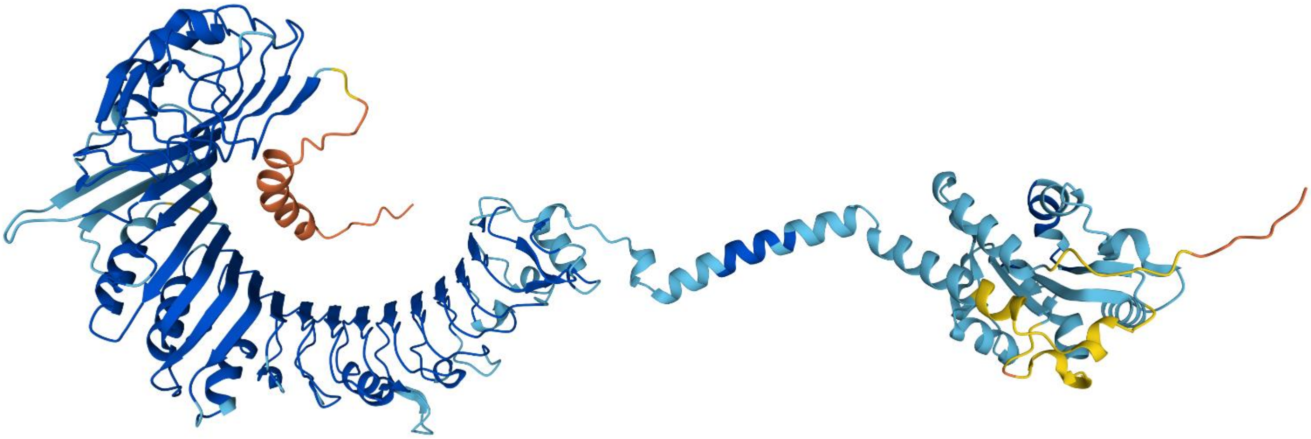

```
>sp|Q9NYK1|TLR7_HUMAN Toll-like receptor 7 OS=Homo sapiens OX=9606 GN=TLR7 PE=1 SV=1
MVFPMWTLKRQILILFNIILISKLLGARWFPKTLPCDVTLDVPKNHVIVDCTDKHLTEIPGGIPTNTTNLTLTINHIPDISPASF
HRLDHLVEIDFRCNCVPIPLGSKNNMCIKRLQIKPRSFSGLTYLKSLYLDGNQLLEIPQGLPPSLQLLSLEANNIFSIRKENLTE
LANIEILYLGQNCYYRNPCYVSYSIEKDAFLNLTKLKVLSLKDNNVTAVPTVLPSTLTELYLYNNMIAKIQEDDFNNLNQLQILD
LSGNCPRCYNAPFPCAPCKNNSPLQIPVNAFDALTELKVLRLHSNSLQHVPPRWFKNINKLQELDLSQNFLAKEIGDAKFLHFLP
SLIQLDLSFNFELQVYRASMNLSQAFSSLKSLKILRIRGYVFKELKSFNLSPLHNLQNLEVLDLGTNFIKIANLSMFKQFKRLKV
IDLSVNKISPSGDSSEVGFCSNARTSVESYEPQVLEQLHYFRYDKYARSCRFKNKEASFMSVNESCYKYGQTLDLSKNSIFFVKS
SDFQHLSFLKCLNLSGNLISQTLNGSEFQPLAELRYLDFSNNRLDLLHSTAFEELHKLEVLDISSNSHYFQSEGITHMLNFTKNL
KVLQKLMMNDNDISSSTSRTMESESLRTLEFRGNHLDVLWREGDNRYLQLFKNLLKLEELDISKNSLSFLPSGVFDGMPPNLKNL
SLAKNGLKSFSWKKLQCLKNLETLDLSHNQLTTVPERLSNCSRSLKNLILKNNQIRSLTKYFLQDAFQLRYLDLSSNKIQMIQKT
SFPENVLNNLKMLLLHHNRFLCTCDAVWFVWWVNHTEVTIPYLATDVTCVGPGAHKGQSVISLDLYTCELDLTNLILFSLSISVS
LFLMVMMTASHLYFWDVWYIYHFCKAKIKGYQRLISPDCCYDAFIVYDTKDPAVTEWVLAELVAKLEDPREKHFNLCLEERDWLP
GQPVLENLSQSIQLSKKTVFVMTDKYAKTENFKIAFYLSHQRLMDEKVDVIILIFLEKPFQKSKFLQLRKRLCGSSVLEWPTNPQ
AHPYFWQCLKNALATDNHVAYSQVFKETV
```

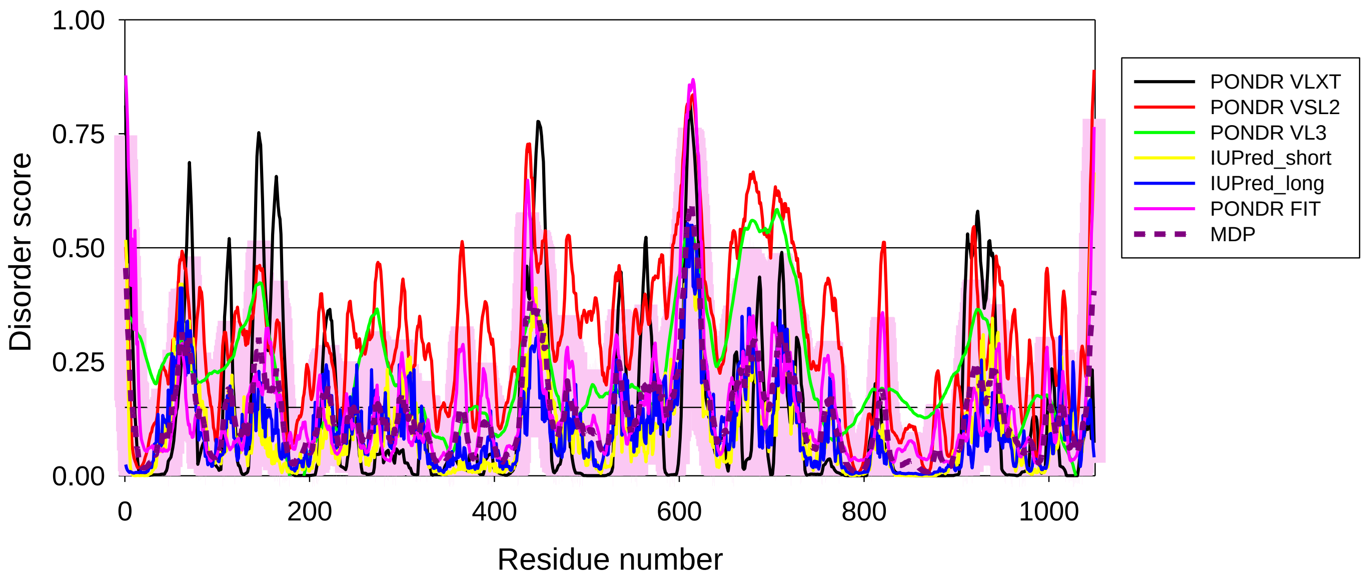

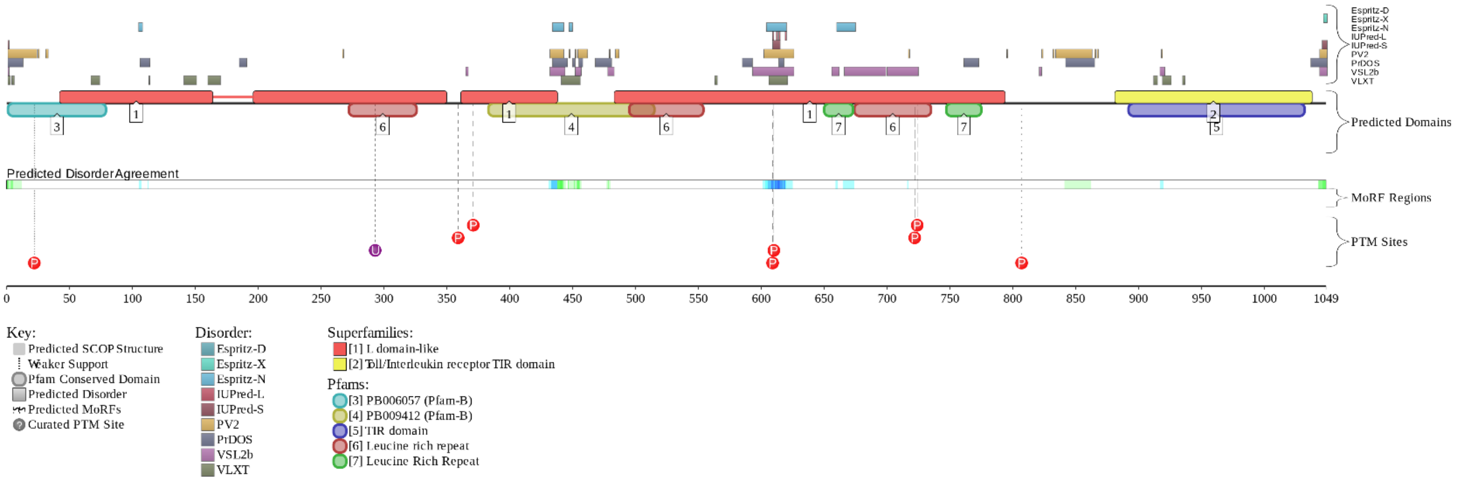

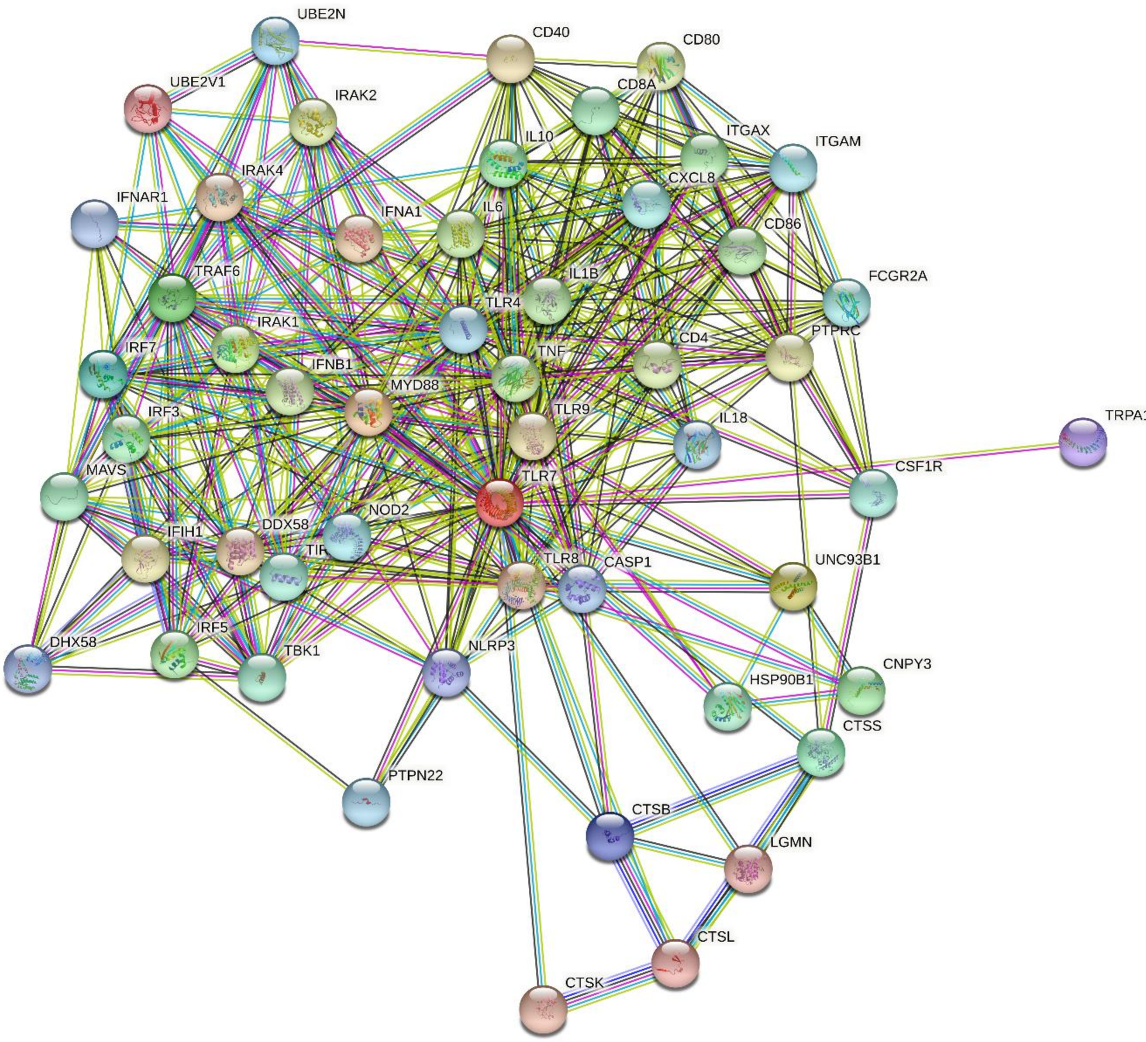
UBE2N
CD40
CD80
UBE2V1
IRAK2
CD8A
IL10
ITGAX
ITGAM
IRAK4
CXCL8
IFNAR1
IFNA1
IL6
CD86
TRAF6
IL1B
FCGR2A
TLR4
IRAK1
CD4
PTPRC
IRF7
TNF
IFNB1
MYD88
IRF3
TLR9
IL18
TRPA1
MAVS
TLR7
CSF1R
NOD2
IFIH1
DDX58
TLR8
CASP1
UNC93B1
DHX58
IRF5
TBK1
NLRP3
CNPY3
HSP90B1
CTSS
PTPN22
CTSB
LGMN
CTSL
CTSK

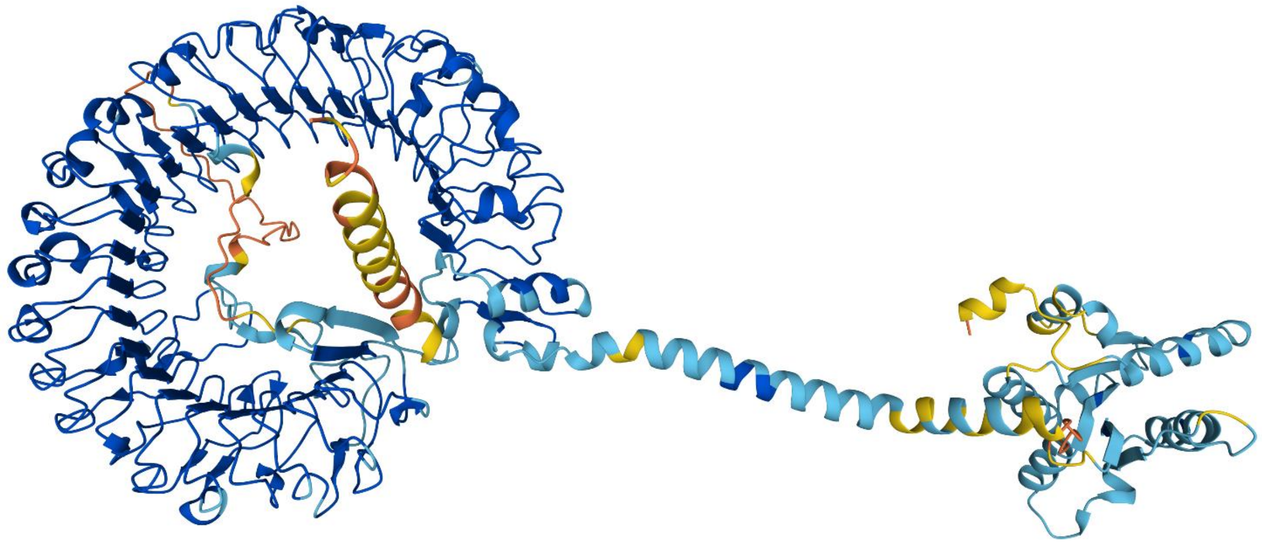

```
>sp|Q9NR97|TLR8_HUMAN Toll-like receptor 8 OS=Homo sapiens OX=9606 GN=TLR8 PE=1 SV=1
MENMFLQSSMLTCIFLLISGSCELCAEENFSRSYPCDEKKQNDSVIAECSNRRLQEVPQTVGKYVTELDLSDNFITHITNESFQG
LQNLTKINLNHNPNVQHQNGNPGIQSNGLNITDGAFLNLKNLRELLLEDNQLPQIPSGLPESLTELSLIQNNIYNITKEGISRLI
NLKNLYLAWNCYFNKVCEKTNIEDGVFETLTNLELLSLSFNSLSHVPPKLPSSLRKLFLSNTQIKYISEEDFKGLINLTLLDLSG
NCPRCFNAPFPCVPCDGGASINIDRFAFQNLTQLRYLNLSSTSLRKINAAWFKNMPHLKVLDLEFNYLVGEIASGAFLTMLPRLE
ILDLSFNYIKGSYPQHINISRNFSKLLSLRALHLRGYVFQELREDDFQPLMQLPNLSTINLGINFIKQIDFKLFQNFSNLEIIYL
SENRISPLVKDTRQSYANSSSFQRHIRKRRSTDFEFDPHSNFYHFTRPLIKPQCAAYGKALDLSLNSIFFIGPNQFENLPDIACL
NLSANSNAQVLSGTEFSAIPHVKYLDLTNNRLDFDNASALTELSDLEVLDLSYNSHYFRIAGVTHHLEFIQNFTNLKVLNLSHNN
IYTLTDKYNLESKSLVELVFSGNRLDILWNDDDNRYISIFKGLKNLTRLDLSLNRLKHIPNEAFLNLPASLTELHINDNMLKFFN
WTLLQQFPRLELLDLRGNKLLFLTDSLSDFTSSLRTLLLSHNRISHLPSGFLSEVSSLKHLDLSSNLLKTINKSALETKTTTKLS
MLELHGNPFECTCDIGDFRRWMDEHLNVKIPRLVDVICASPGDQRGKSIVSLELTTCVSDVTAVILFFFTFFITTMVMLAALAHH
LFYWDVWFIYNVCLAKVKGYRSLSTSQTFYDAYISYDTKDASVTDWVINELRYHLEESRDKNVLLCLEERDWDPGLAIIDNLMQS
INQSKKTVFVLTKKYAKSWNFKTAFYLALQRLMDENMDVIIFILLEPVLQHSQYLRLRQRICKSSILQWPDNPKAEGLFWQTLRN
VVLTENDSRYNNMYVDSIKQY
```

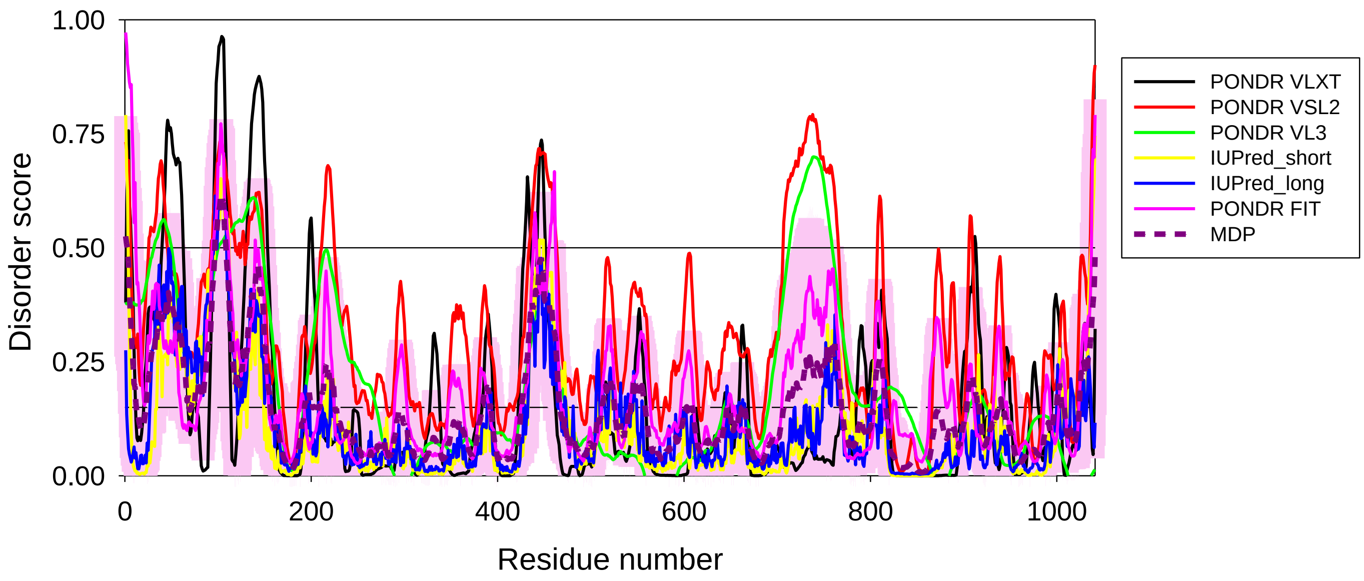

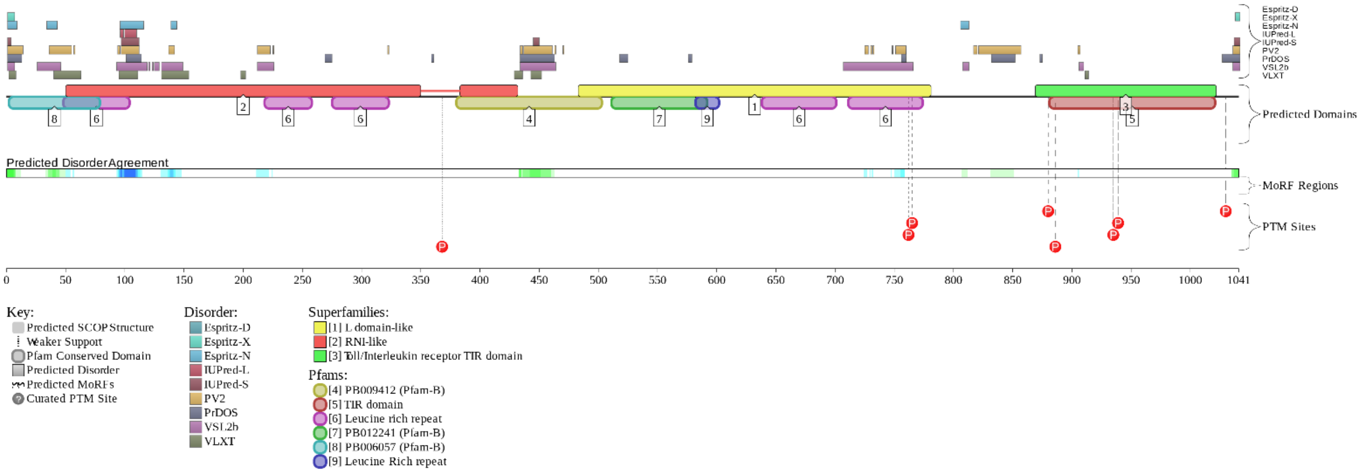

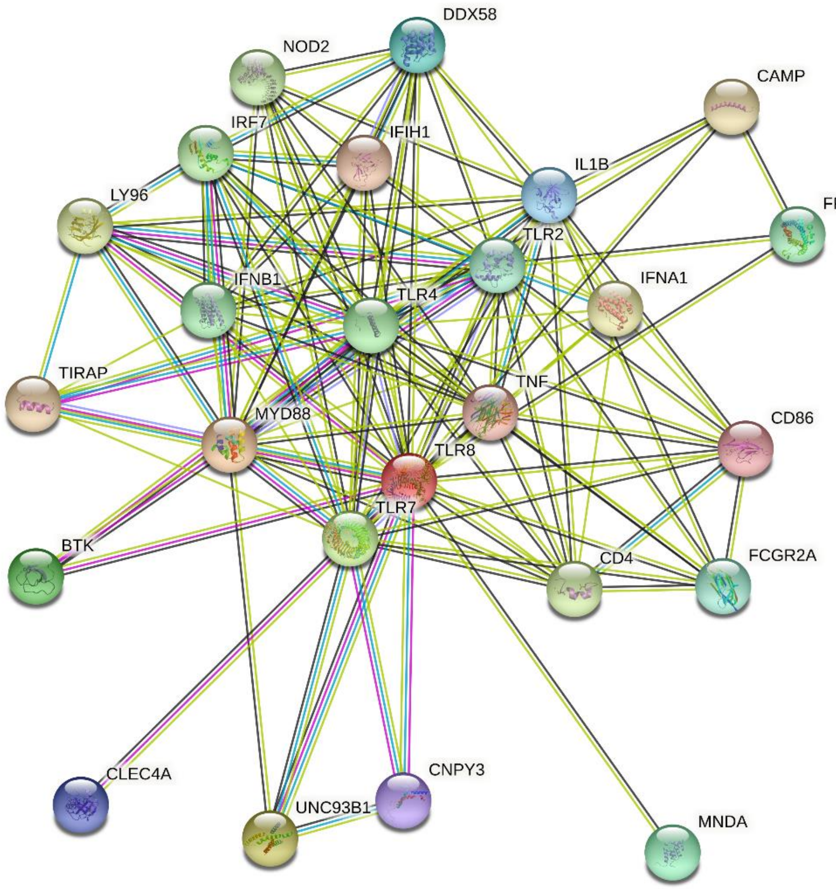
DDX58
NOD2
CAMP
IRF7
IFIH1
IL1B
LY96
FPR1
TLR2
IFNB1
IFNA1
TLR4
TIRAP
TNF
MYD88
CD86
TLR8
TLR7
BTK
CD4
FCGR2A
CLEC4A
CNPY3
UNC93B1
MNDA

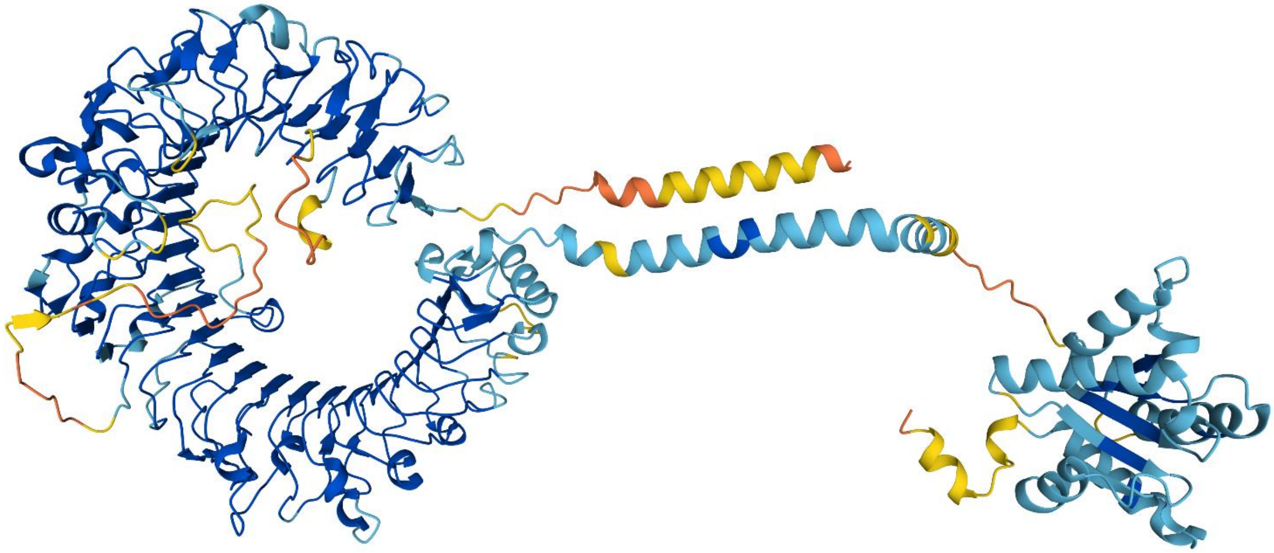

>sp|Q9NR96|TLR9_HUMAN Toll-like receptor 9 OS=Homo sapiens OX=9606 GN=TLR9 PE=1 SV=2
MGFCRSALHPLSLLVQAIMLAMTLALGTLPAFLPCELQPHGLVNCNWLFLKSVPHFSMAAPRGNVTSLSLSSNRIHHLHDSDFAH
LPSLRHLNLKWNCPPVGLSPMHFPCHMTIEPSTFLAVPTLEELNLSYNNIMTVPALPKSLISLSLSHTNILMLDSASLAGLHALR
FLFMDGNCYYKNPCRQALEVAPGALLGLGNLTHLSLKYNNLTVVPRNLPSSLEYLLLSYNRIVKLAPEDLANLTALRVLDVGGNC
RRCDHAPNPCMECPRHFPQLHPDTFSHLSRLEGLVLKDSSLSWLNASWFRGLGNLRVLDLSENFLYKCITKTKAFQGLTQLRKLN
LSFNYQKRVSFAHLSLAPSFGSLVALKELDMHGIFFRSLDETTLRPLARLPMLQTLRLQMNFINQAQLGIFRAFPGLRYVDLSDN
RISGASELTATMGEADGGEKVWLQPGDLAPAPVDTPSSEDFRPNCSTLNFTLDLSRNNLVTVQPEMFAQLSHLQCLRLSHNCISQ
AVNGSQFLPLTGLQVLDLSHNKLDLYHEHSFTELPRLEALDLSYNSQPFGMQGVGHNFSFVAHLRTLRHLSLAHNNIHSQVSQQL
CSTSLRALDFSGNALGHMWAEGDLYLHFFQGLSGLIWLDLSQNRLHTLLPQTLRNLPKSLQVLRLRDNYLAFFKWWSLHFLPKLE
VLDLAGNQLKALTNGSLPAGTRLRRLDVSCNSISFVAPGFFSKAKELRELNLSANALKTVDHSWFGPLASALQILDVSANPLHCA
CGAAFMDFLLEVQAAVPGLPSRVKCGSPGQLQGLSIFAQDLRLCLDEALSWDCFALSLLAVALGLGVPMLHHLCGWDLWYCFHLC
LAWLPWRGRQSGRDEDALPYDAFVVFDKTQSAVADWVYNELRGQLEECRGRWALRLCLEERDWLPGKTLFENLWASVYGSRKTLF
VLAHTDRVSGLLRASFLLAQQRLLEDRKDVVVLVILSPDGRRSRYVRLRQRLCRQSVLLWPHQPSGQRSFWAQLGMALTRDNHHF
YNRNFCQGPTAE

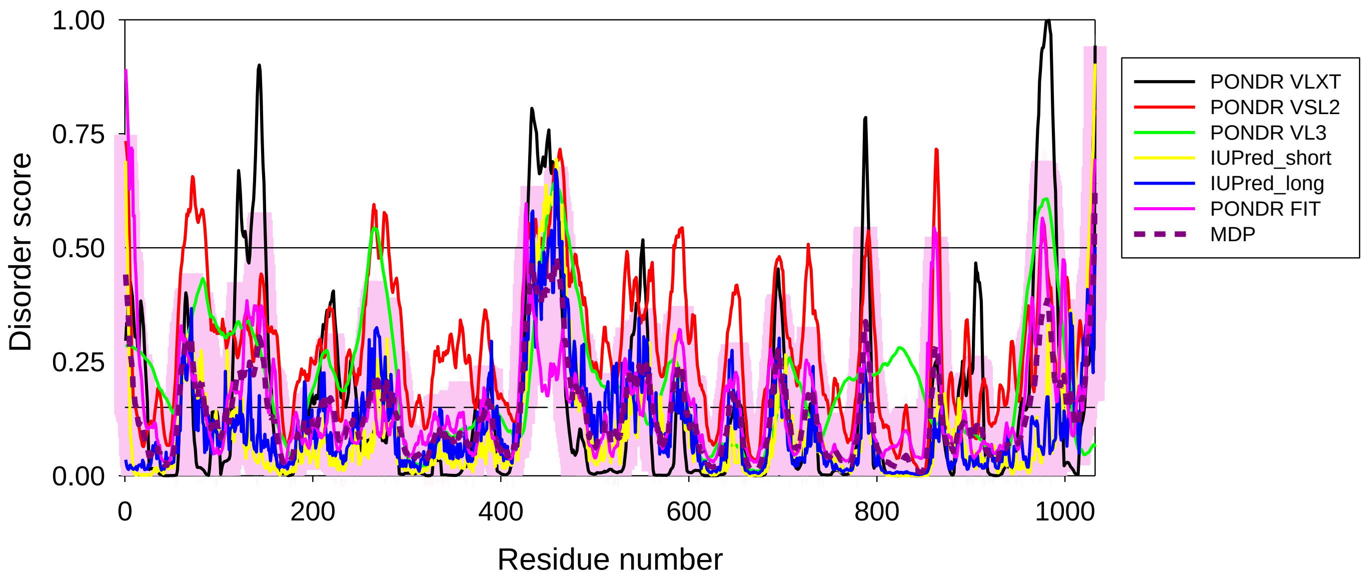

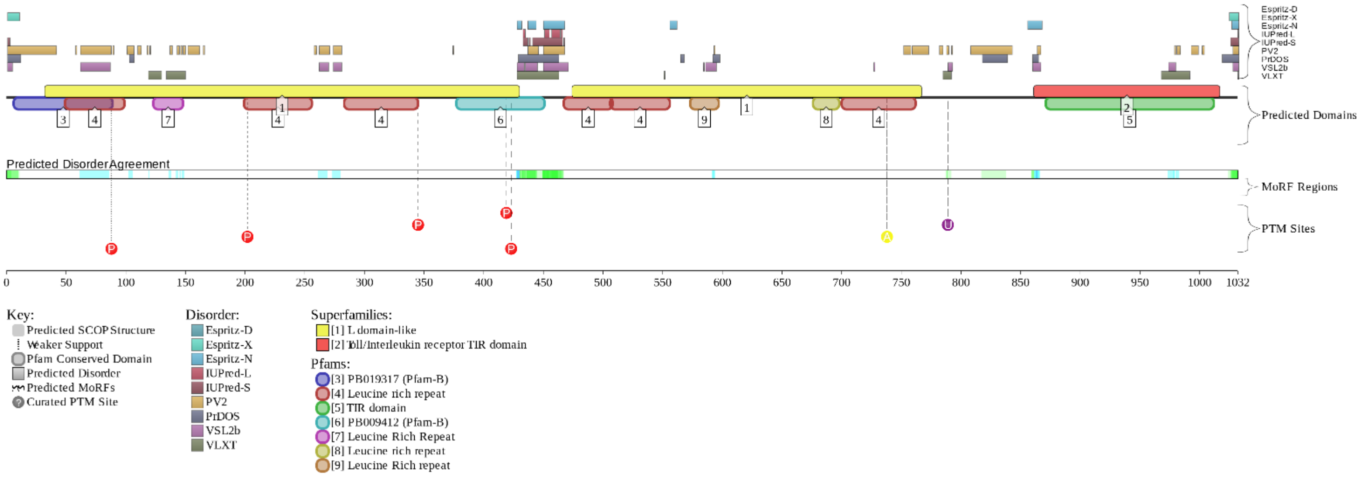

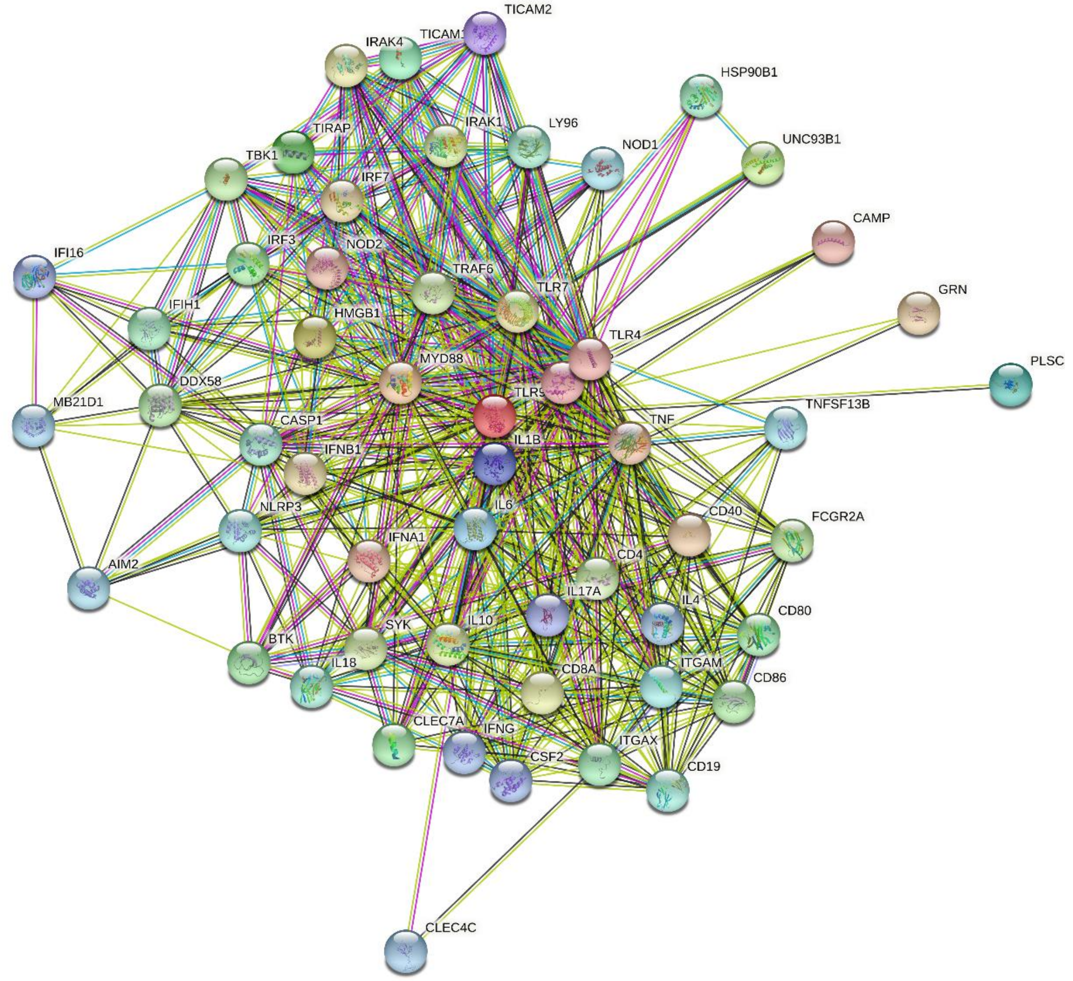
TICAM2
IRAK4
TICAM1
HSP90B1
TIRAP
IRAK1
LY96
NOD1
UNC93B1
TBK1
IRF7
CAMP
IFI16
IRF3
NOD2
TRAF6
TLR7
GRN
IFIH1
HMGB1
TLR4
MYD88
PLSC
DDX58
TLR9
MB21D1
TNF
TNFSF13B
CASP1
IL1B
IFNB1
IL6
NLRP3
CD40
FCGR2A
IFNA1
CD4
AIM2
IL17A
IL4
CD80
BTK
SYK
IL10
IL18
CD8A
ITGAM
CD86
CLEC7A
IFNG
ITGAX
CSF2
CD19
CLEC4C

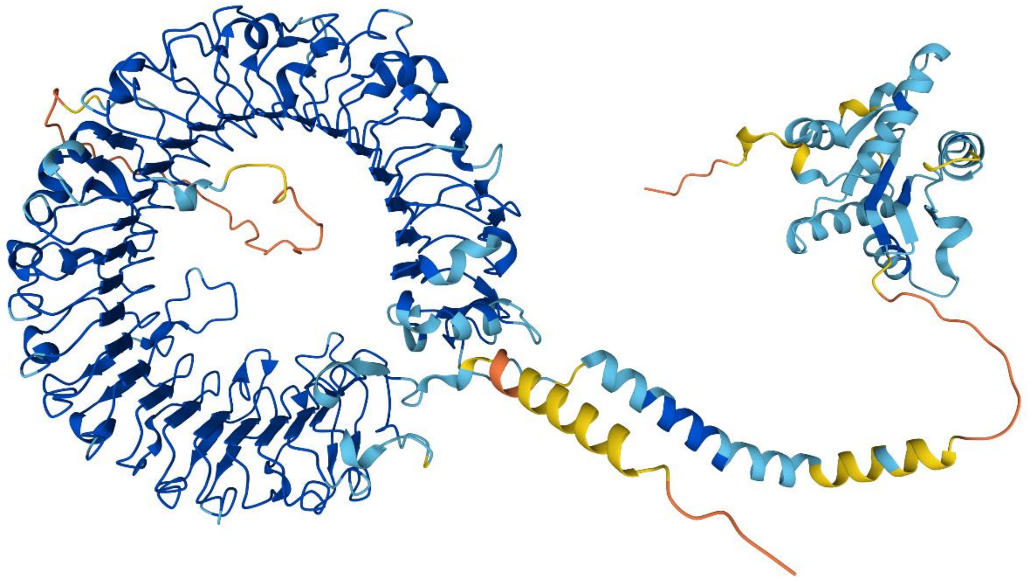

>sp|Q9BXR5|TLR10_HUMAN Toll-like receptor 10 OS=Homo sapiens OX=9606 GN=TLR10 PE=1 SV=2
MRLIRNIYIFCSIVMTAEGDAPELPEERELMTNCSNMSLRKVPADLTPATTTLDLSYNLLFQLQSSDFHSVSKLRVLILCHNRIQ
QLDLKTFEFNKELRYLDLSNNRLKSVTWYLLAGLRYLDLSFNDFDTMPICEEAGNMSHLEILGLSGAKIQKSDFQKIAHLHLNTV
FLGFRTLPHYEEGSLPILNTTKLHIVLPMDTNFWVLLRDGIKTSKILEMTNIDGKSQFVSYEMQRNLSLENAKTSVLLLNKVDLL
WDDLFLILQFVWHTSVEHFQIRNVTFGGKAYLDHNSFDYSNTVMRTIKLEHVHFRVFYIQQDKIYLLLTKMDIENLTISNAQMPH
MLFPNYPTKFQYLNFANNILTDELFKRTIQLPHLKTLILNGNKLETLSLVSCFANNTPLEHLDLSQNLLQHKNDENCSWPETVVN
MNLSYNKLSDSVFRCLPKSIQILDLNNNQIQTVPKETIHLMALRELNIAFNFLTDLPGCSHFSRLSVLNIEMNFILSPSLDFVQS
CQEVKTLNAGRNPFRCTCELKNFIQLETYSEVMMVGWSDSYTCEYPLNLRGTRLKDVHLHELSCNTALLIVTIVVIMLVLGLAVA
FCCLHFDLPWYLRMLGQCTQTWHRVRKTTQEQLKRNVRFHAFISYSEHDSLWVKNELIPNLEKEDGSILICLYESYFDPGKSISE
NIVSFIEKSYKSIFVLSPNFVQNEWCHYEFYFAHHNLFHENSDHIILILLEPIPFYCIPTRYHKLKALLEKKAYLEWPKDRRKCG
LFWANLRAAINVNVLATREMYELQTFTELNEESRGSTISLMRTDCL

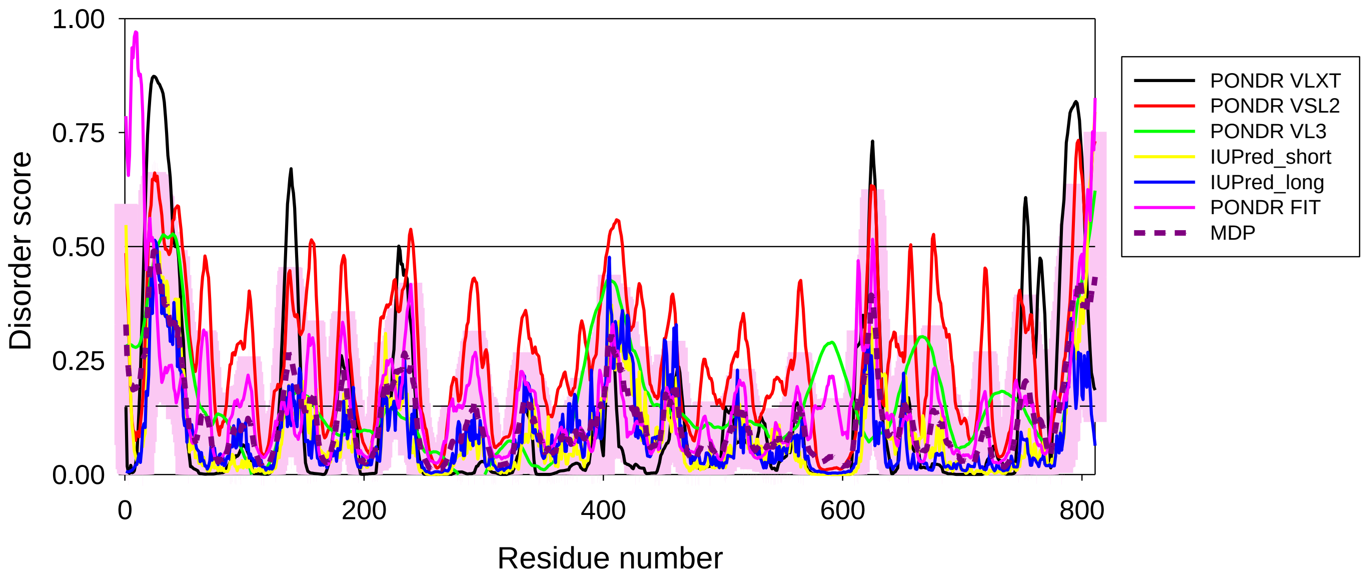

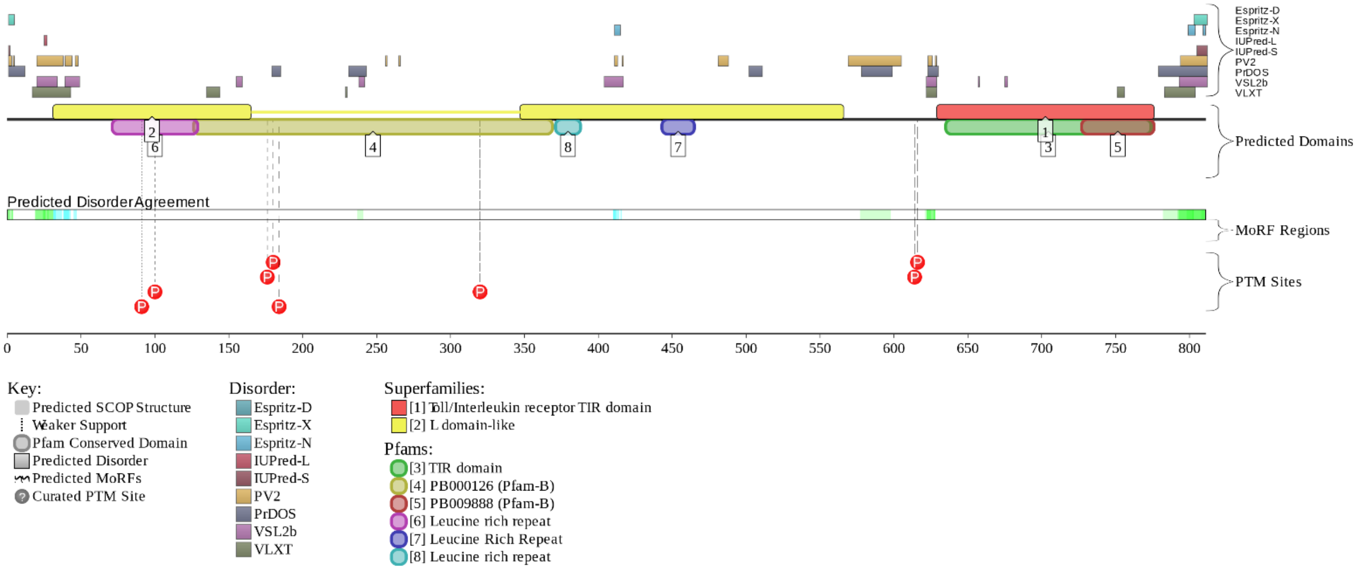

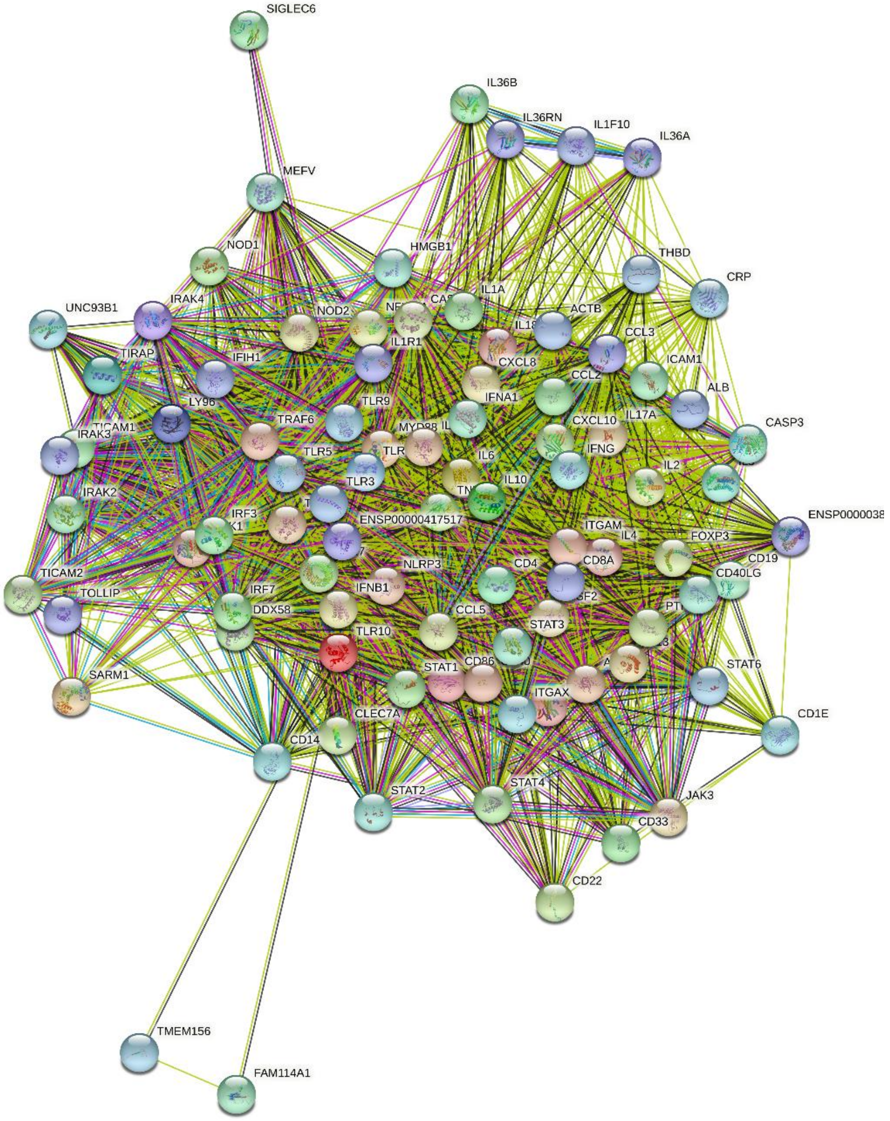
SIGLEC6
IL36B
IL36RN
IL1F10
IL36A
MEFV
HMGB1
THBD
CRP
NOD1
UNC93B1
IRAK4
IL1A
NOD2
ACTB
CCL3
TIRAP
IFIH1
IL1R1
CXCL8
ICAM1
ALB
LY96
TLR9
IFNA1
CCL2
IL17A
TICAM1
TRAF6
CXCL10
CASP3
IRAK3
TLR5
IFNG
IL6
IL2
TLR3
IL10
IRAK2
IRF3
ENSP00000417517
ITGAM
IL4
FOXP3
CD8A
CD19
NLRP3
CD4
CD40LG
TICAM2
IRF7
IFNB1
TOLLIP
DDX58
CCL5
STAT3
TLR10
STAT6
SARM1
STAT1
CD86
ITGAX
CLEC7A
CD1E
CD14
STAT4
JAK3
STAT2
CD33
CD22
TMEM156
FAM114A1

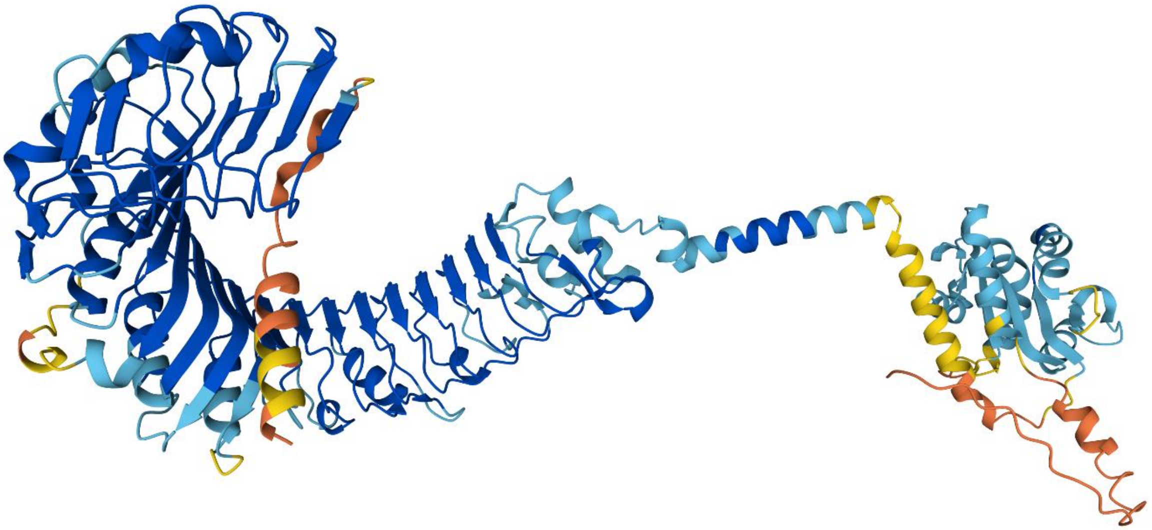

Proteins in TLR-related signaling pathways

>sp|P58753|TIRAP_HUMAN Toll/interleukin-1 receptor domain-containing adapter protein OS=Homo sapiens OX=9606 GN=TIRAP PE=1 SV=2
MASSTSLPAPGSRPKKPLGKMADWFRQTLLKKPKKRPNSPESTSSDASQPTSQDSPLPPSLSSVTSPSLPPTHASDSGSSRWSKD
YDVCVCHSEEDLVAAQDLVSYLEGSTASLRCFLQLRDATPGGAIVSELCQALSSSHCRVLLITPGFLQDPWCKYQMLQALTEAPG
AEGCTIPLLSGLSRAAYPPELRFMYYVDGRGPDGGFRQVKEAVMRYLQTLS

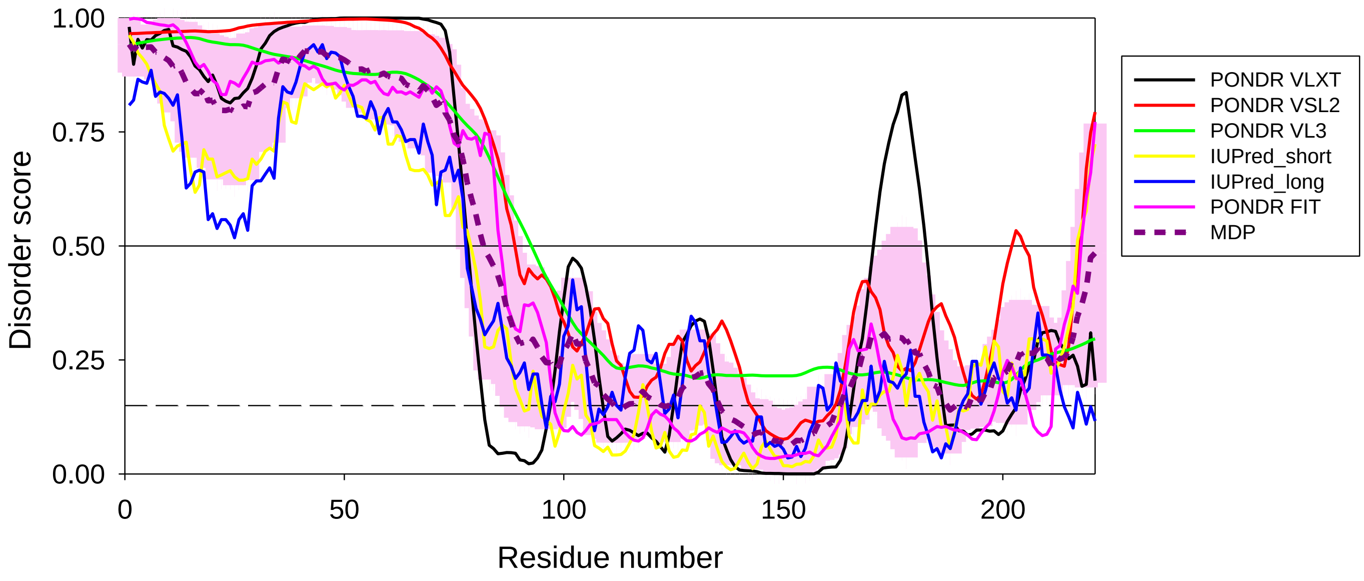

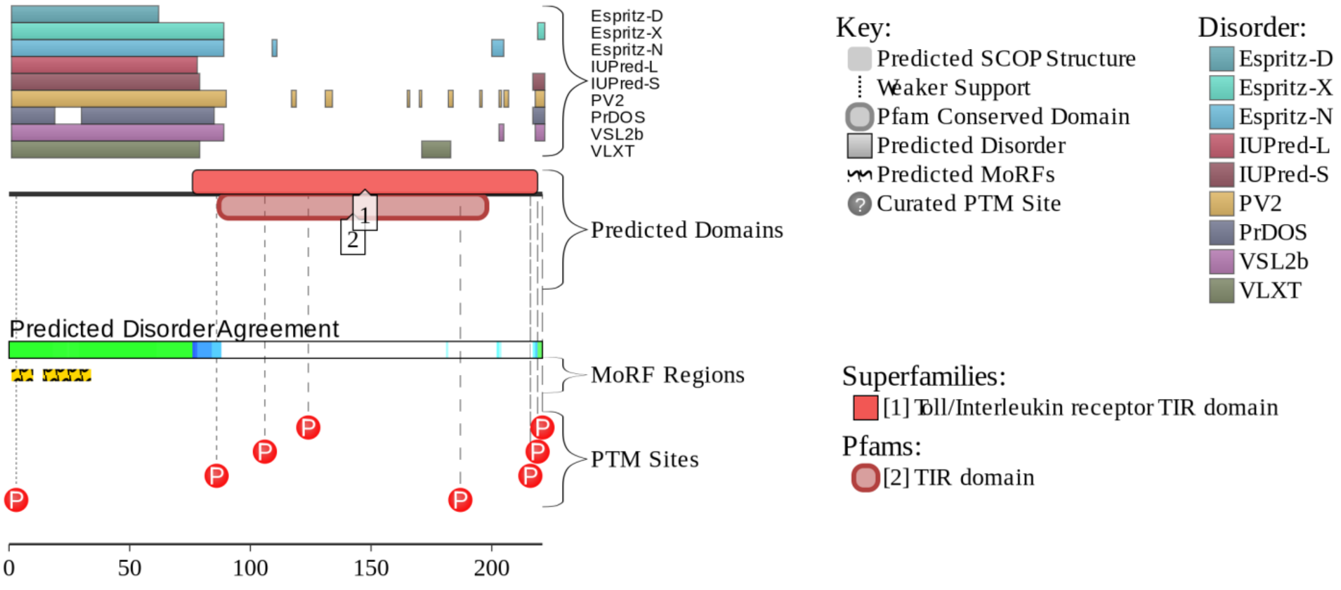

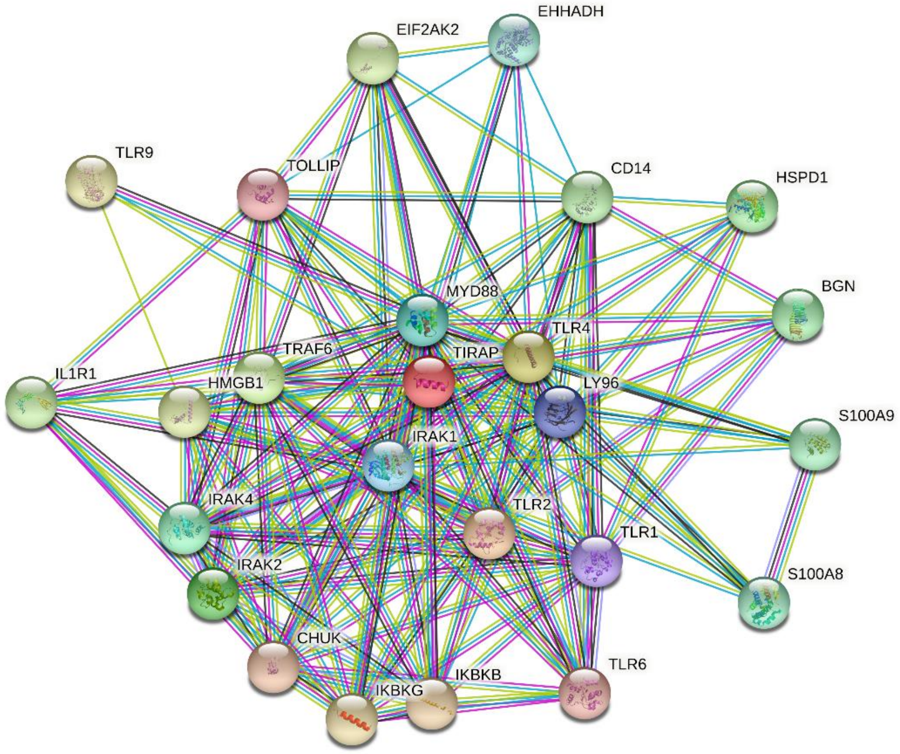
EHHADH
EIF2AK2
TLR9
TOLLIP
CD14
HSPD1
BGN
MYD88
TLR4
TRAF6
TIRAP
IL1R1
HMGB1
LY96
S100A9
IRAK1
IRAK4
TLR2
TLR1
IRAK2
S100A8
CHUK
TLR6
IKBKB
IKBKG

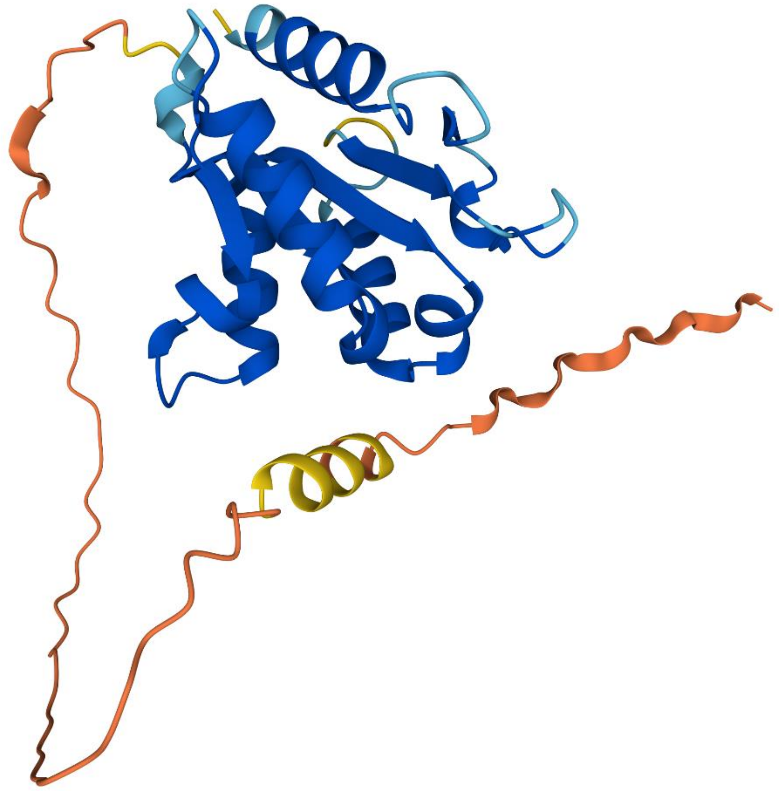

>sp|Q99836|MYD88_HUMAN Myeloid differentiation primary response protein MyD88 OS=Homo sapiens OX=9606 GN=MYD88 PE=1 SV=1
MAAGGPGAGSAAPVSSTSSLPLAALNMRVRRRLSLFLNVRTQVAADWTALAEEMDFEYLEIRQLETQADPTGRLLDAWQGRPGAS
VGRLLELLTKLGRDDVLLELGPSIEEDCQKYILKQQQEEAEKPLQVAAVDSSVPRTAELAGITTLDDPLGHMPERFDAFICYCPS
DIQFVQEMIRQLEQTNYRLKLCVSDRDVLPGTCVWSIASELIEKRCRRMVVVVSDDYLQSKECDFQTKFALSLSPGAHQKRLIPI
KYKAMKKEFPSILRFITVCDYTNPCTKSWFWTRLAKALSLP

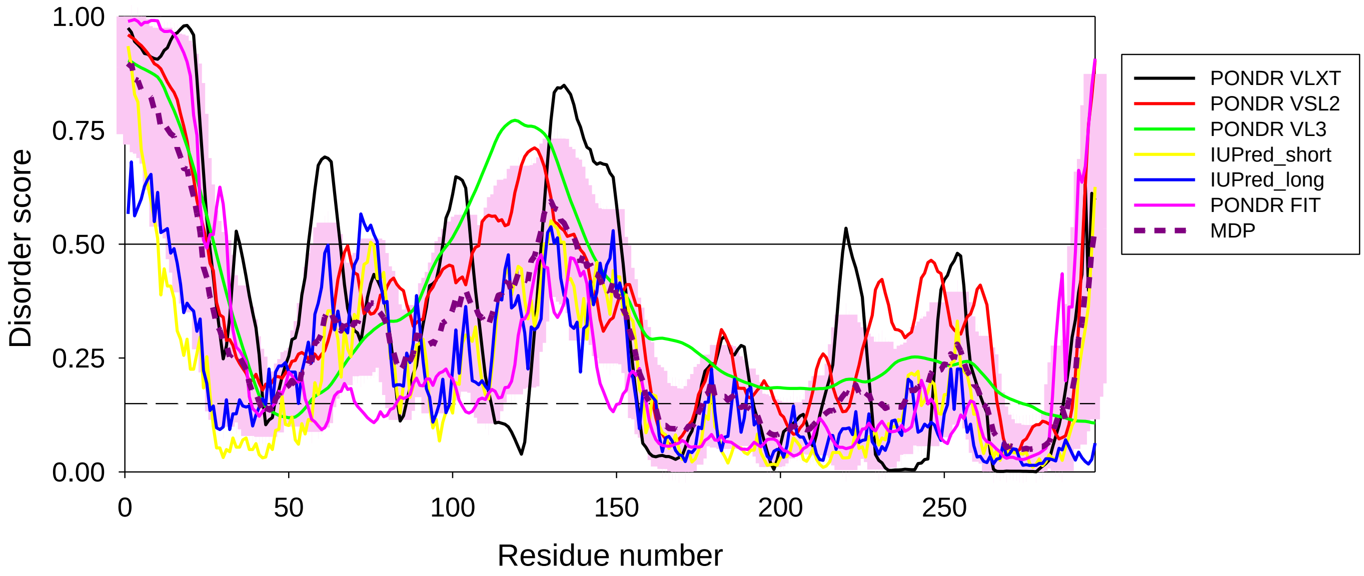

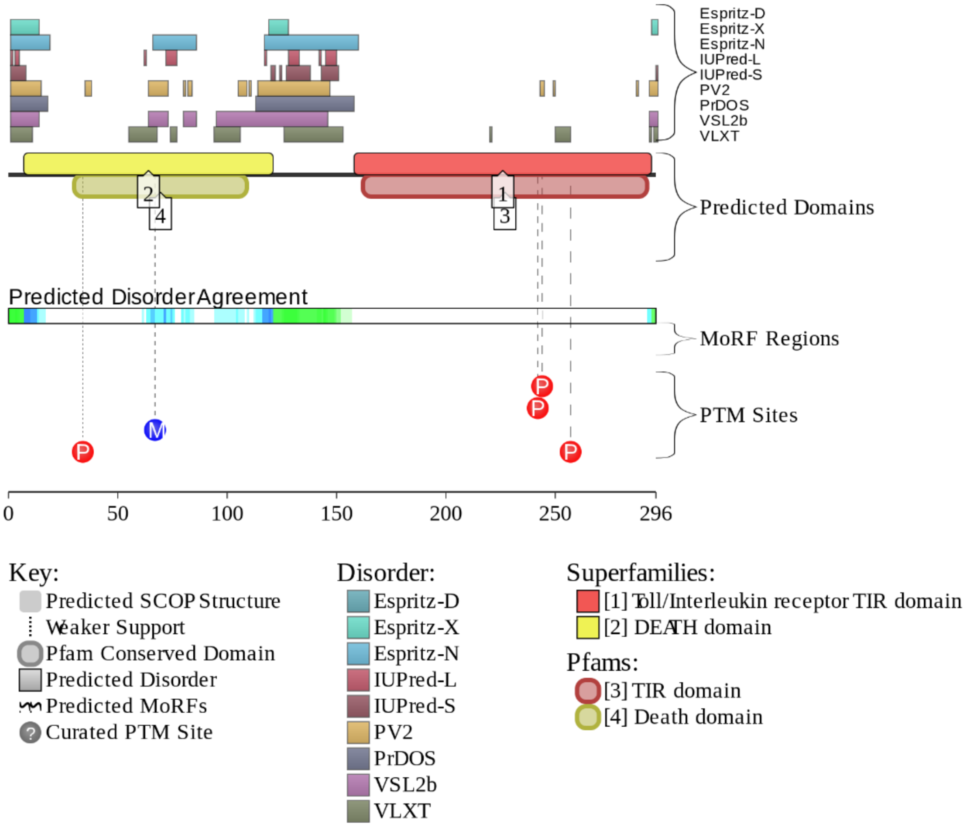

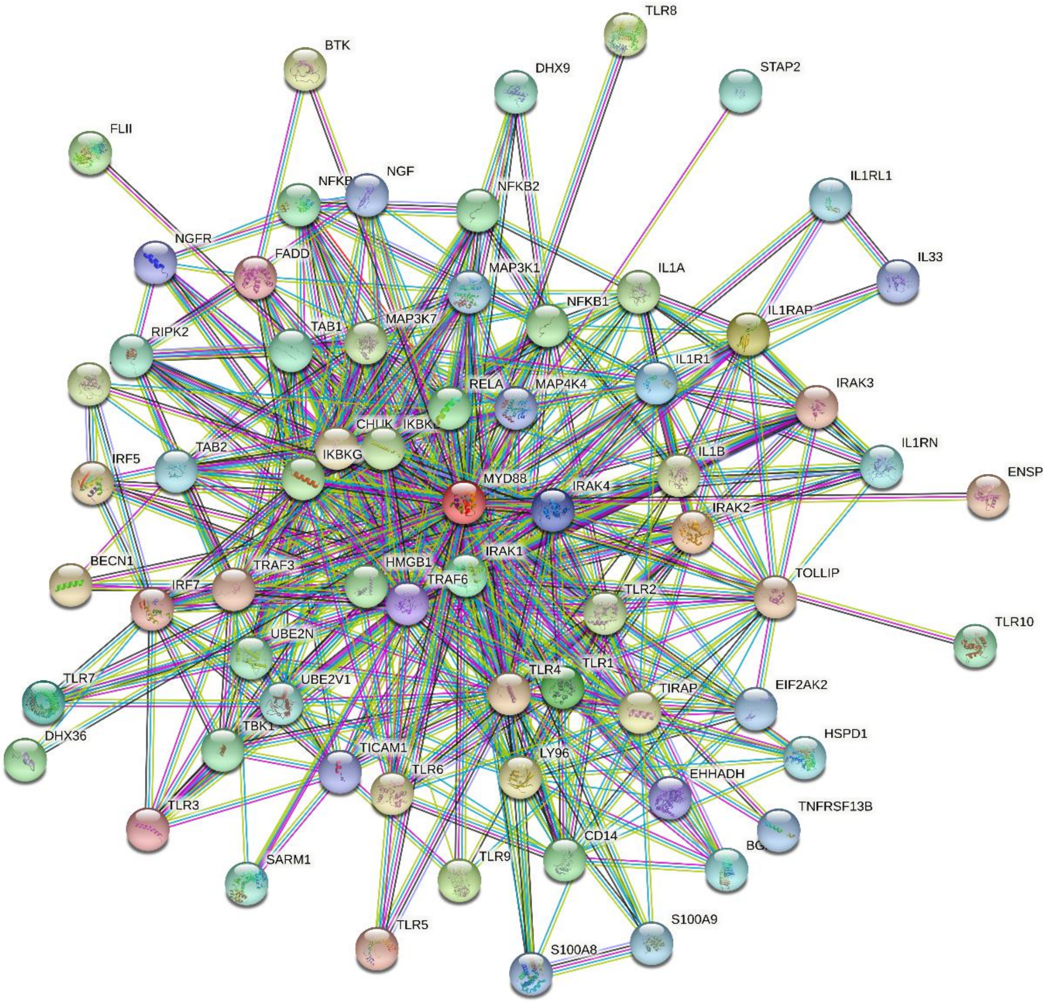
TLR8
BTK
DHX9
STAP2
FLII
NFKB
NGF
NFKB2
IL1RL1
NGFR
FADD
MAP3K1
IL33
IL1A
NFKB1
IL1RAP
TAB1
MAP3K7
RIPK2
IL1R1
IRAK3
RELA
MAP4K4
CHUK
IKBK
TAB2
IKBKG
IL1RN
IRF5
IL1B
ENSP
MYD88
IRAK4
IRAK2
BECN1
IRAK1
HMGB1
TRAF3
TOLLIP
IRF7
TRAF6
TLR2
TLR10
UBE2N
TLR7
TLR4
TLR1
UBE2V1
TIRAP
EIF2AK2
DHX36
TBK1
HSPD1
TICAM1
LY96
TLR6
EHHADH
TLR3
TNFRSF13B
CD14
BG
SARM1
TLR9
S100A9
TLR5
S100A8

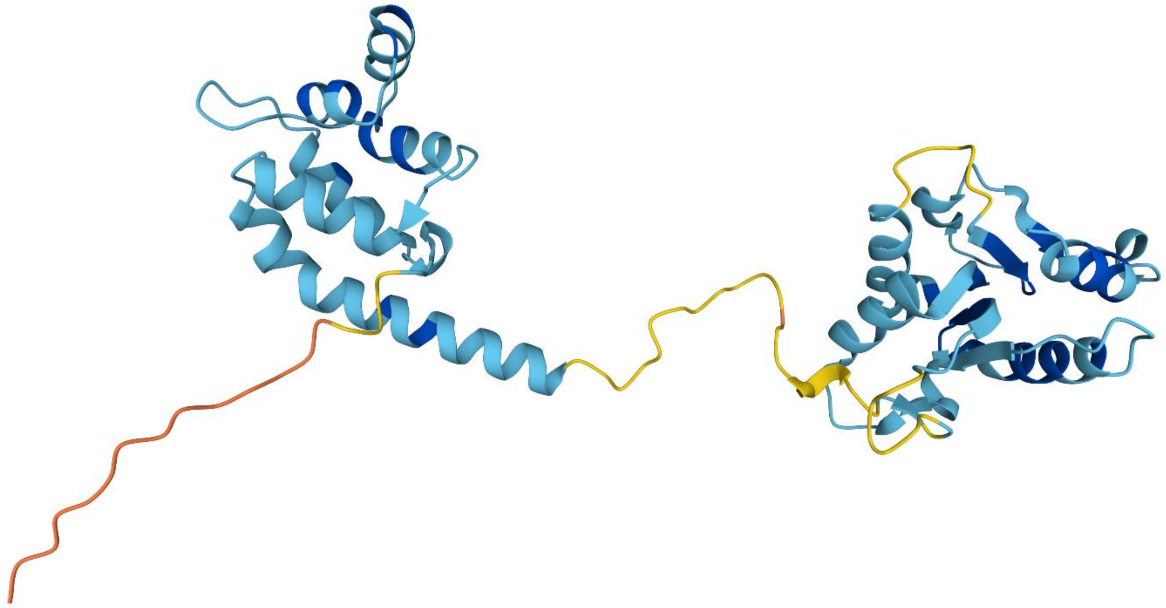

```
>sp|P51617|IRAK1_HUMAN Interleukin-1 receptor-associated kinase 1 OS=Homo sapiens
OX=9606 GN=IRAK1 PE=1 SV=2
MAGGPGPGEPAAPGAQHFLYEVPPWVMCRFYKVMDALEPADWCQFAALIVRDQTELRLCERSGQRTASVLWPWINRNARVADLVH
ILTHLQLLRARDIITAWHPPAPLPSPGTTAPRPSSIPAPAEAEAWSPRKLPSSASTFLSPAFPGSQTHSGPELGLVPSPASLWPP
PPSPAPSSTKPGPESSVSLLQGARPFPFCWPLCEISRGTHNFSEELKIGEGGFGCVYRAVMRNTVYAVKRLKENADLEWTAVKQS
FLTEVEQLSRFRHPNIVDFAGYCAQNGFYCLVYGFLPNGSLEDRLHCQTQACPPLSWPQRLDILLGTARAIQFLHQDSPSLIHGD
IKSSNVLLDERLTPKLGDFGLARFSRFAGSSPSQSSMVARTQTVRGTLAYLPEEYIKTGRLAVDTDTFSFGVVVLETLAGQRAVK
THGARTKYLKDLVEEEAEEAGVALRSTQSTLQAGLAADAWAAPIAMQIYKKHLDPRPGPCPPELGLGLGQLACCCLHRRAKRRPP
MTQVYERLEKLQAVVAGVPGHSEAASCIPPSPQENSYVSSTGRAHSGAAPWQPLAAPSGASAQAAEQLQRGPNQPVESDESLGGL
SAALRSWHLTPSCPLDPAPLREAGCPQGDTAGESSWGSGPGSRPTAVEGLALGSSASSSSEPPQIIINPARQKMVQKLALYEDGA
LDSLQLLSSSSLPGLGLEQDRQGPEESDEFQS
```

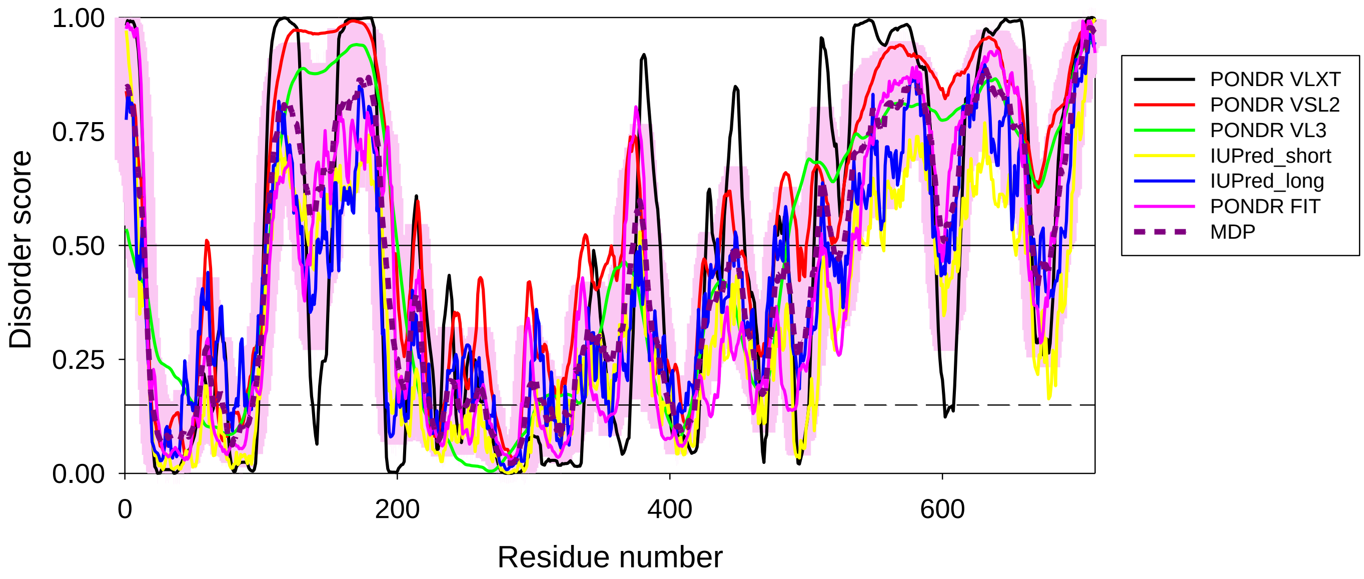

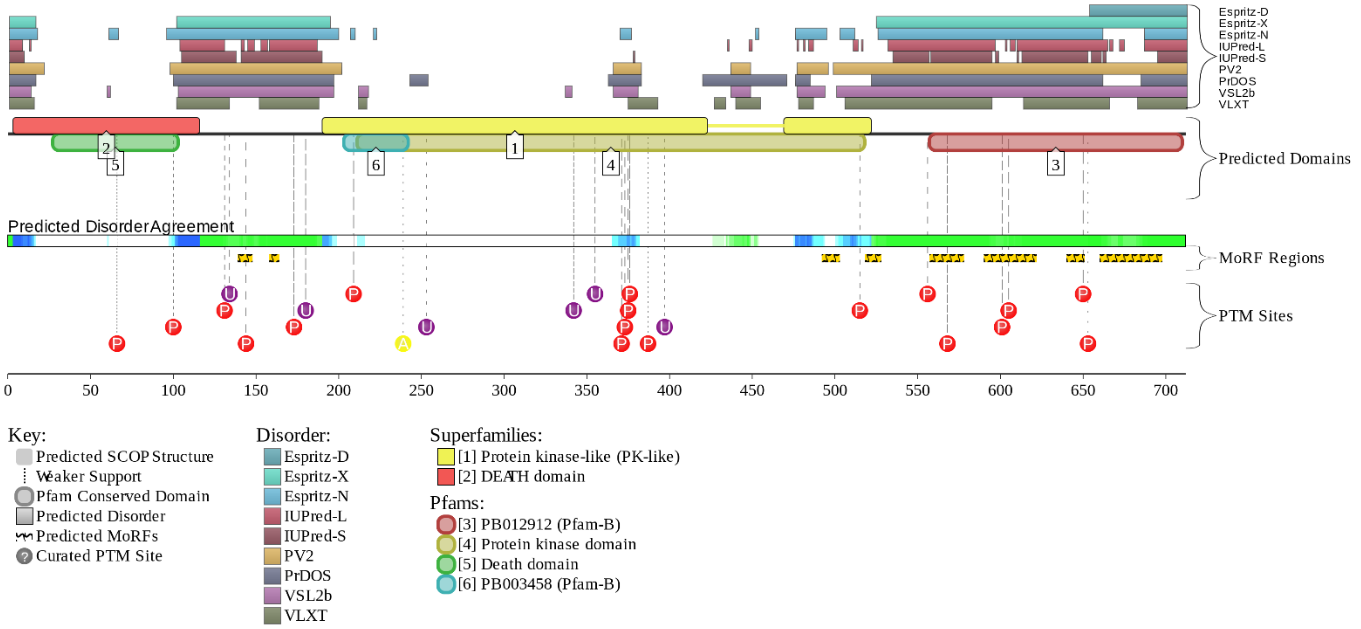

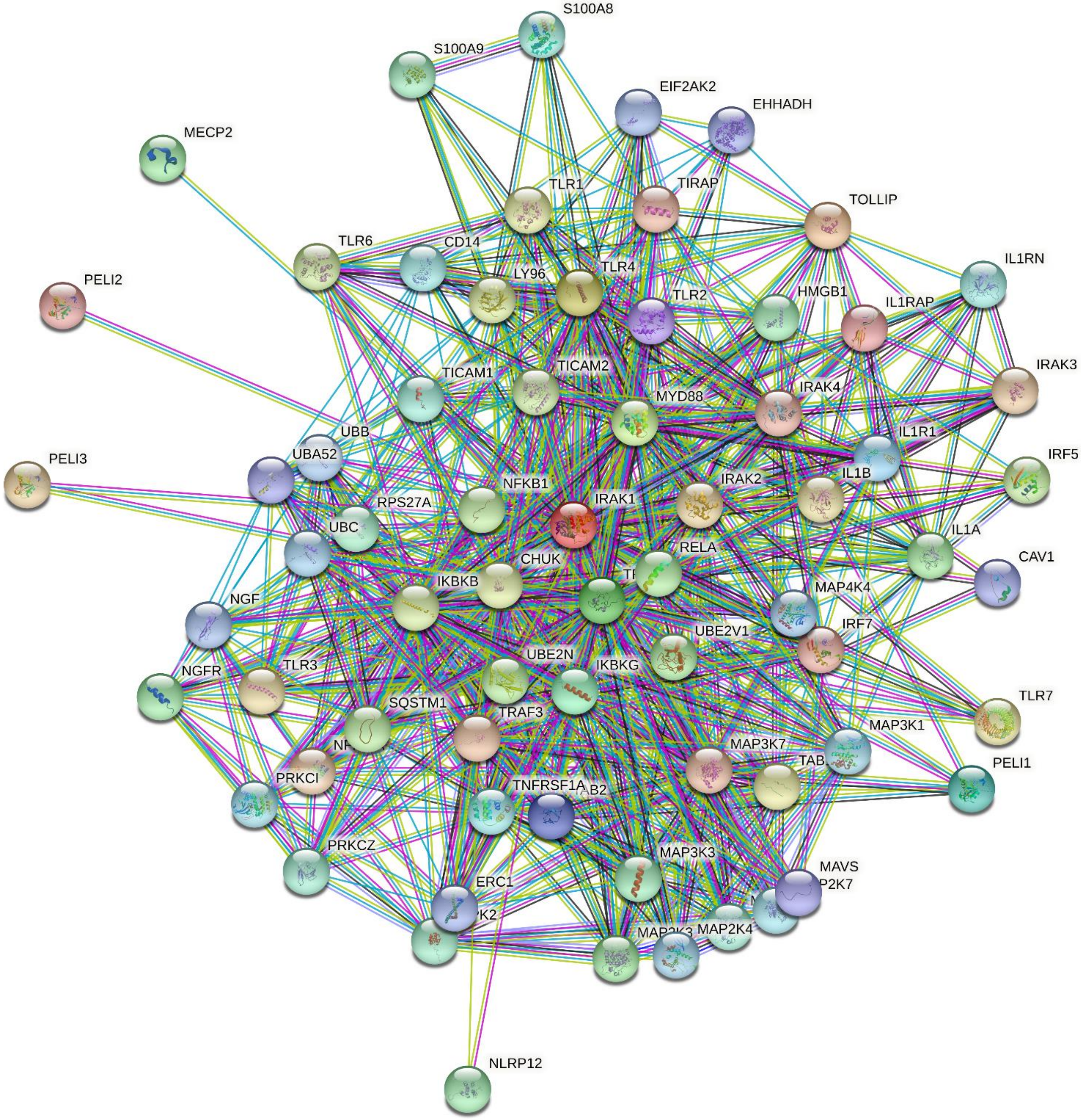
S100A8
S100A9
EIF2AK2
EHHADH
MECP2
TLR1
TIRAP
TOLLIP
TLR6
CD14
LY96
TLR4
IL1RN
PELI2
TLR2
HMGB1
IL1RAP
TICAM1
TICAM2
MYD88
IRAK4
IRAK3
UBB
IL1R1
PELI3
UBA52
IRF5
NFKB1
IRAK2
IL1B
RPS27A
IRAK1
UBC
IL1A
CHUK
RELA
CAV1
IKBKB
MAP4K4
NGF
UBE2V1
IRF7
UBE2N
IKBKG
NGFR
TLR3
TLR7
SQSTM1
MAP3K1
TRAF3
MAP3K7
PRKCI
PELI1
TNFRSF1A
PRKCZ
MAP3K3
MAVS
ERC1
MAP2K4
NLRP12

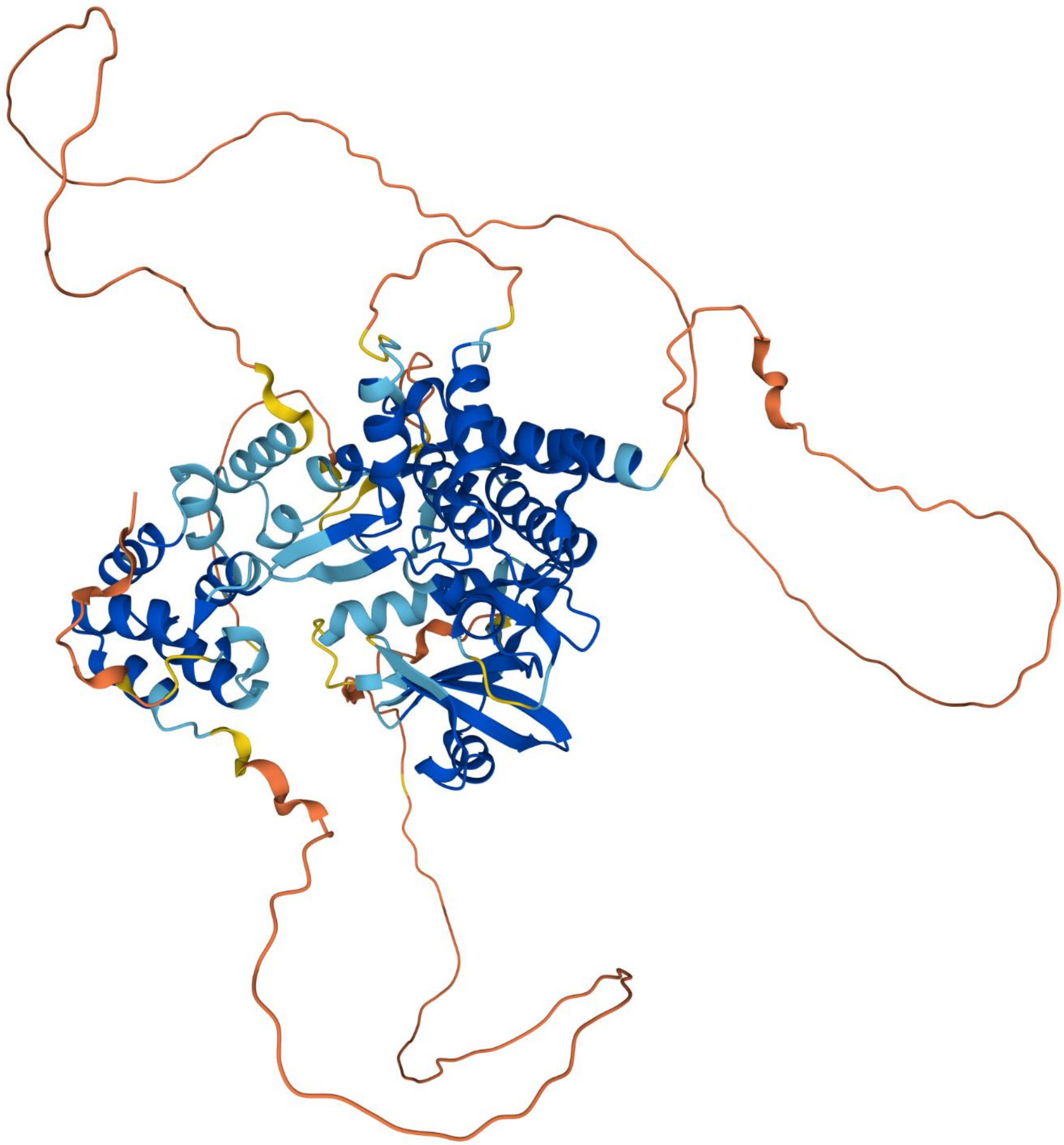

>sp|Q9NWZ3|IRAK4_HUMAN Interleukin-1 receptor-associated kinase 4 OS=Homo sapiens OX=9606 GN=IRAK4 PE=1 SV=1

MNKPITPSTYVRCLNVGLIRKLSDFIDPQEGWKKLAVAIKKPSGDDRYNQFHIRRFEALLQTGKSPTSELLFDWGTTNCTVGDLV
DLLIQNEFFAPASLLLPDAVPKTANTLPSKEAITVQQKQMPFCDKDRTLMTPVQNLEQSYMPPDSSSPENKSLEVSDTRFHSFSF
YELKNVTNNFDERPISVGGNKMGEGGFGVVYKGYVNNTTVAVKKLAAMVDITTEELKQQFDQEIKVMAKCQHENLVELLGFSSDG
DDLCLVYVYMPNGSLLDRLSCLDGTPPLSWHMRCKIAQGAANGINFLHENHHIHRDIKSANILLDEAFTAKISDFGLARASEKFA
QTVMTSRIVGTTAYMAPEALRGEITPKSDIYSFGVVLLEIITGLPAVDEHREPQLLLDIKEEIEDEEKTIEDYIDKKMNDADSTS
VEAMYSVASQCLHEKKNKRPDIKKVQQLLQEMTAS

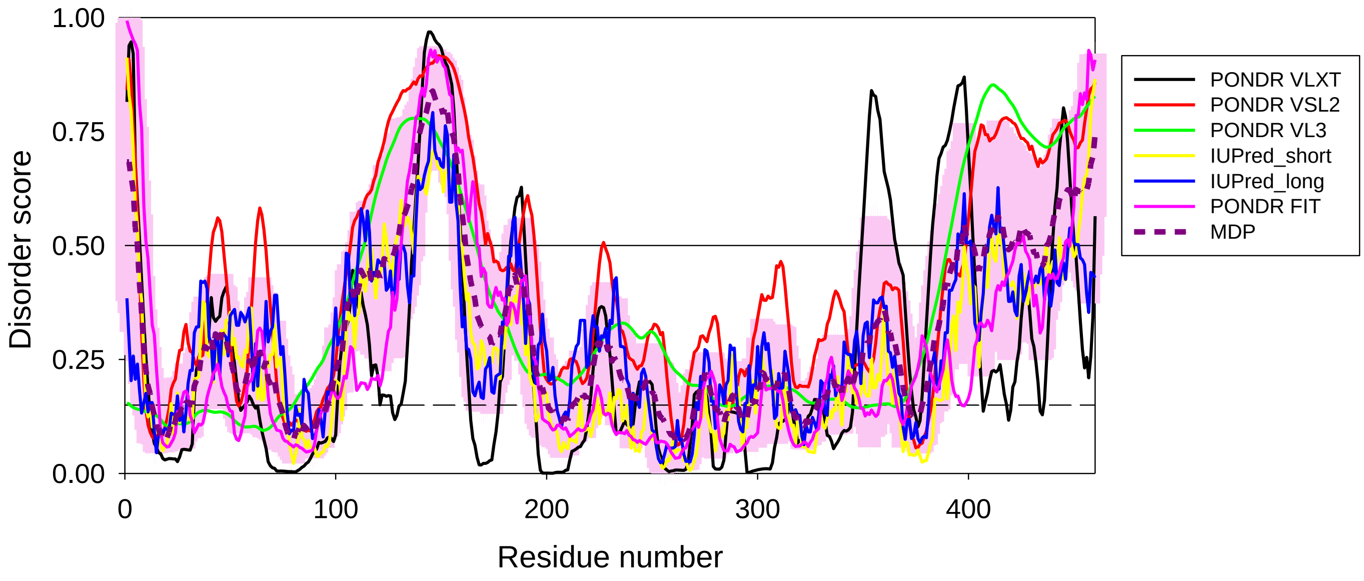

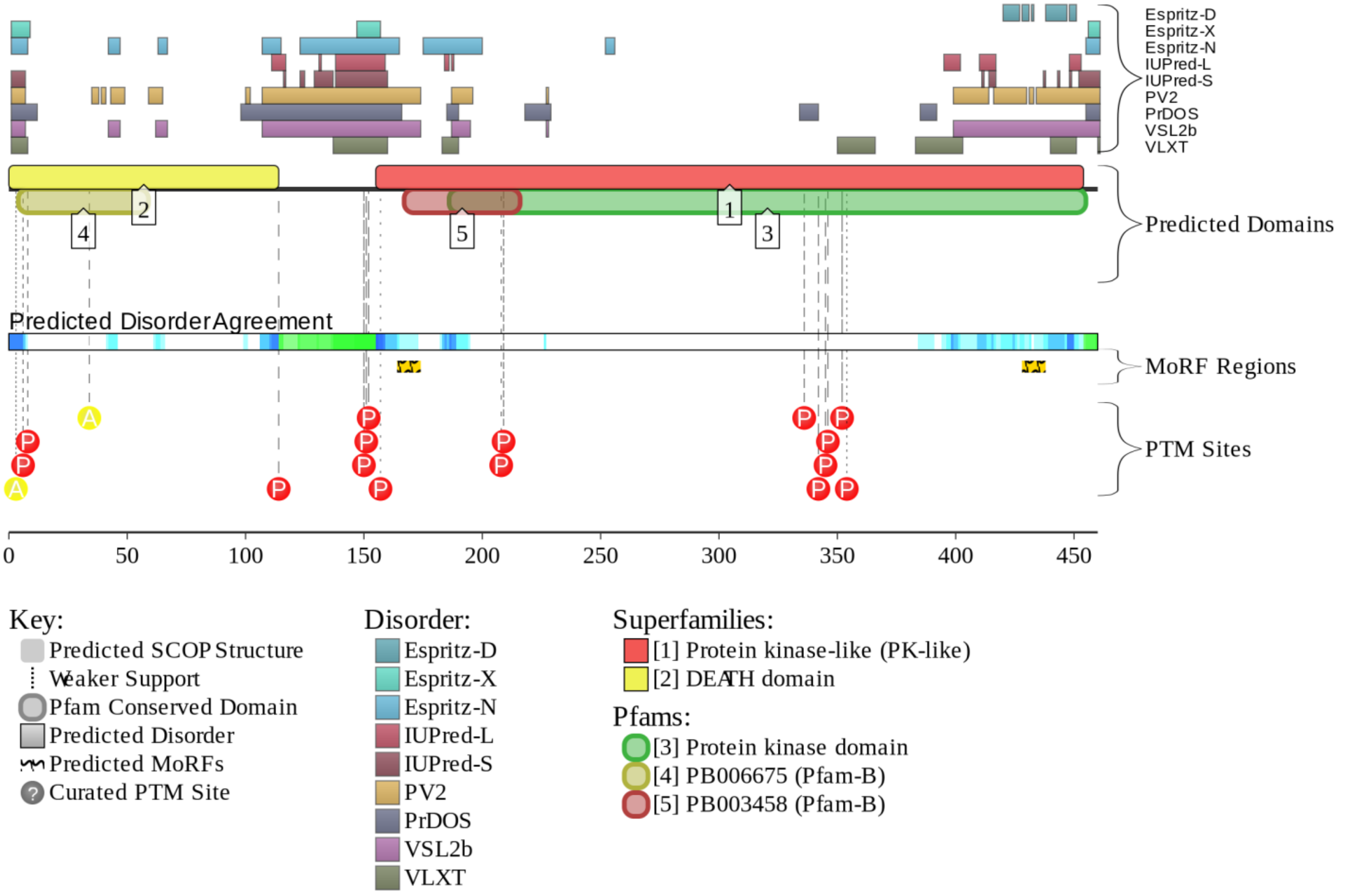

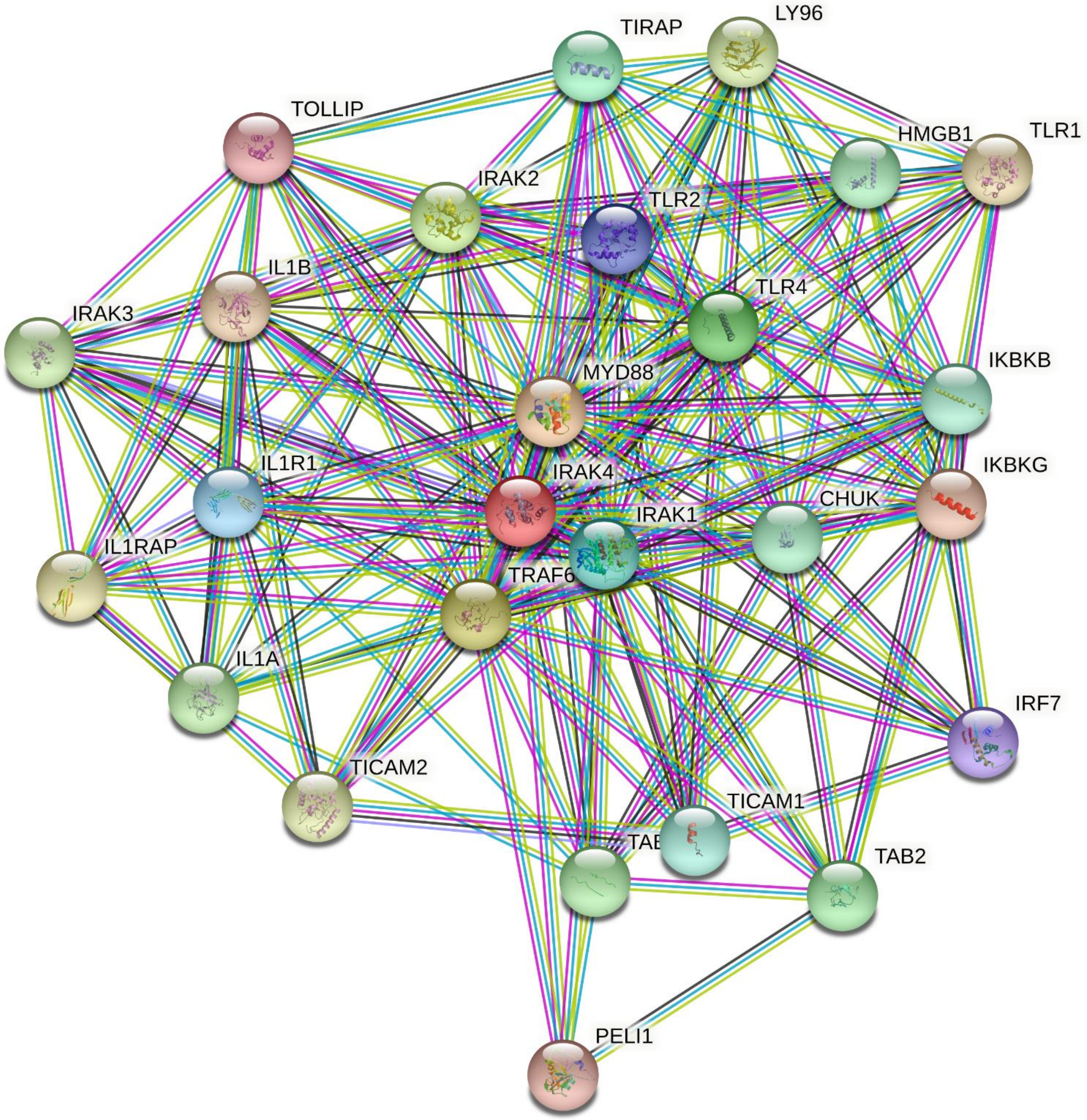
TIRAP
LY96
TOLLIP
HMGB1
TLR1
IRAK2
TLR2
IL1B
TLR4
IRAK3
IKBKB
MYD88
IL1R1
IRAK4
IKBKG
CHUK
IRAK1
IL1RAP
TRAF6
IL1A
IRF7
TICAM2
TICAM1
TAB2
PELI1

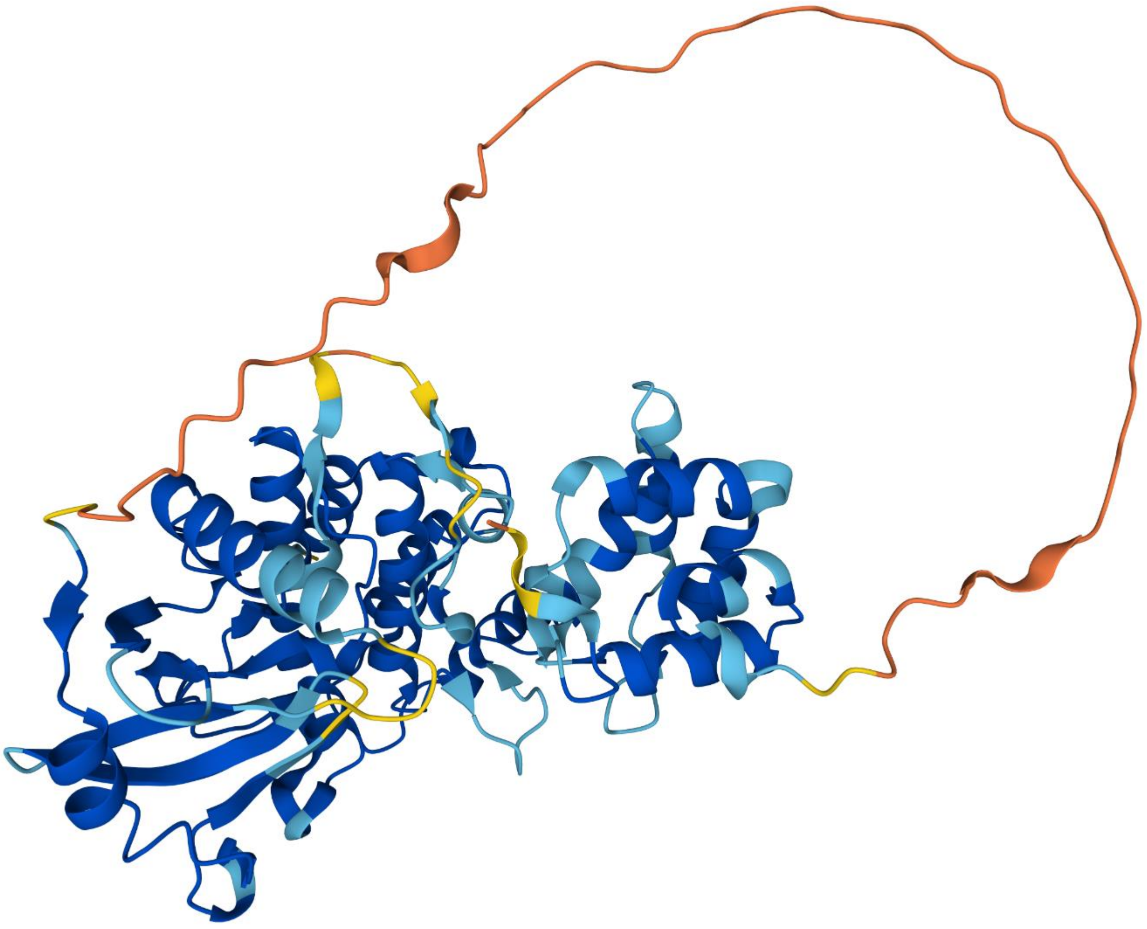

```
>sp|Q9Y4K3|TRAF6_HUMAN TNF receptor-associated factor 6 OS=Homo sapiens OX=9606
GN=TRAF6 PE=1 SV=1
MSLLNCENSCGSSQSESDCCVAMASSCSAVTKDDSVGGTASTGNLSSSFMEEIQGYDVEFDPPLESKYECPICLMALREAVQTPC
GHRFCKACIIKSIRDAGHKCPVDNEILLENQLFPDNFAKREILSLMVKCPNEGCLHKMELRHLEDHQAHCEFALMDCPQCQRPFQ
KFHINIHILKDCPRRQVSCDNCAASMAFEDKEIHDQNCPLANVICEYCNTILIREQMPNHYDLDCPTAPIPCTFSTFGCHEKMQR
NHLARHLQENTQSHMRMLAQAVHSLSVIPDSGYISEVRNFQETIHQLEGRLVRQDHQIRELTAKMETQSMYVSELKRTIRTLEDK
VAEIEAQQCNGIYIWKIGNFGMHLKCQEEEKPVVIHSPGFYTGKPGYKLCMRLHLQLPTAQRCANYISLFVHTMQGEYDSHLPWP
FQGTIRLTILDQSEAPVRQNHEEIMDAKPELLAFQRPTIPRNPKGFGYVTFMHLEALRQRTFIKDDTLLVRCEVSTRFDMGSLRR
EGFQPRSTDAGV
```

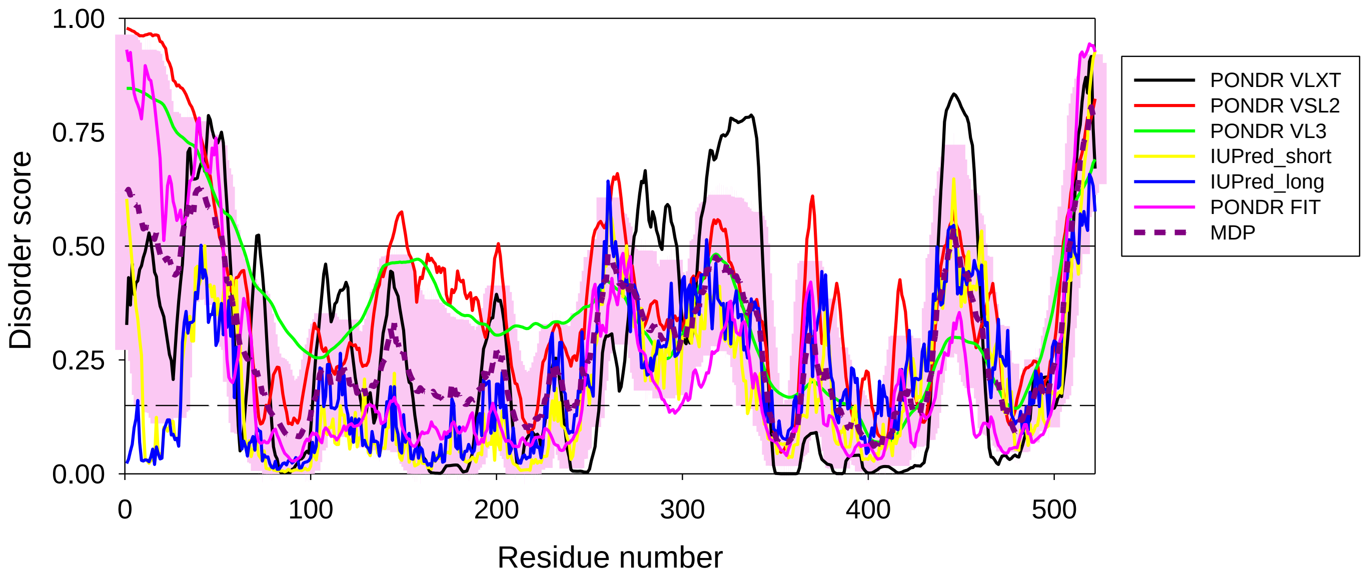

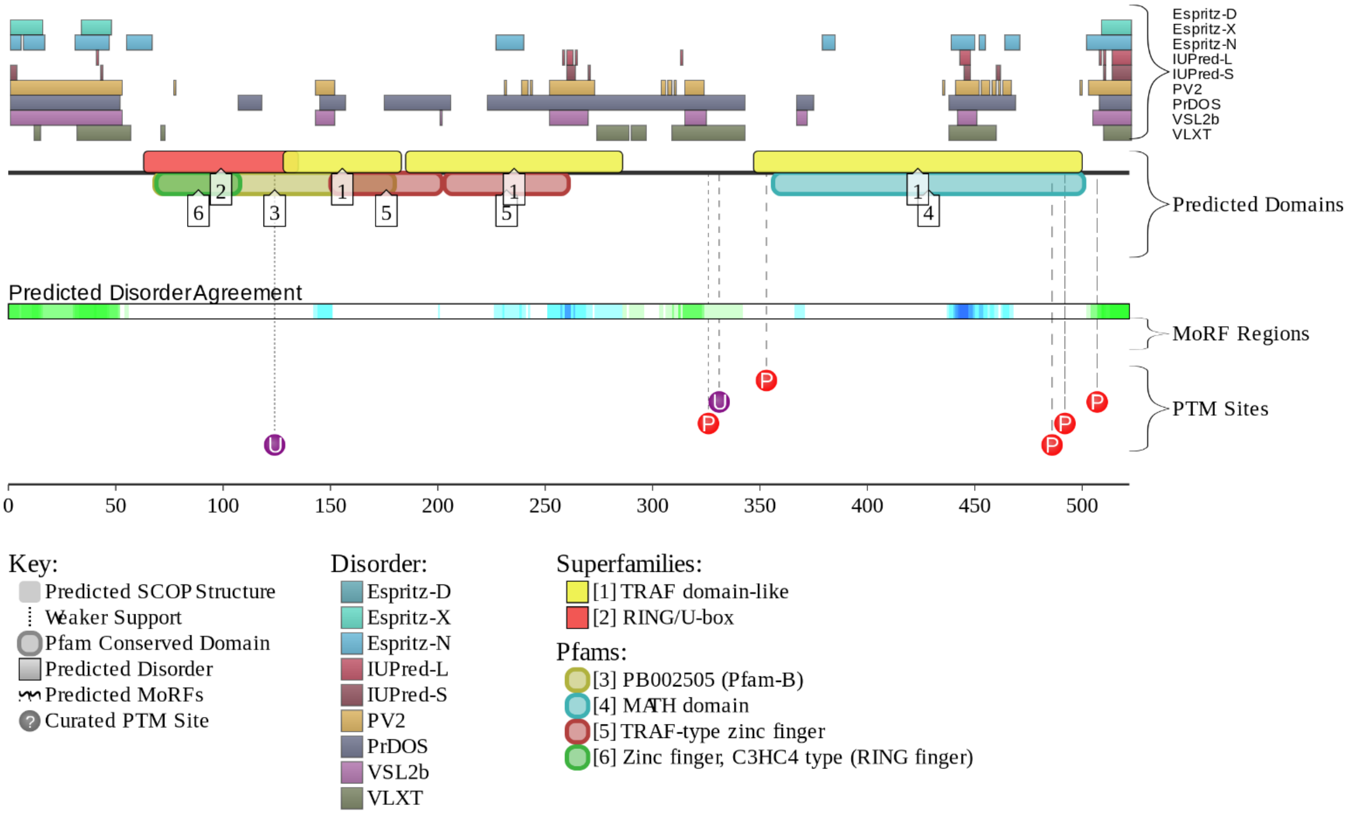

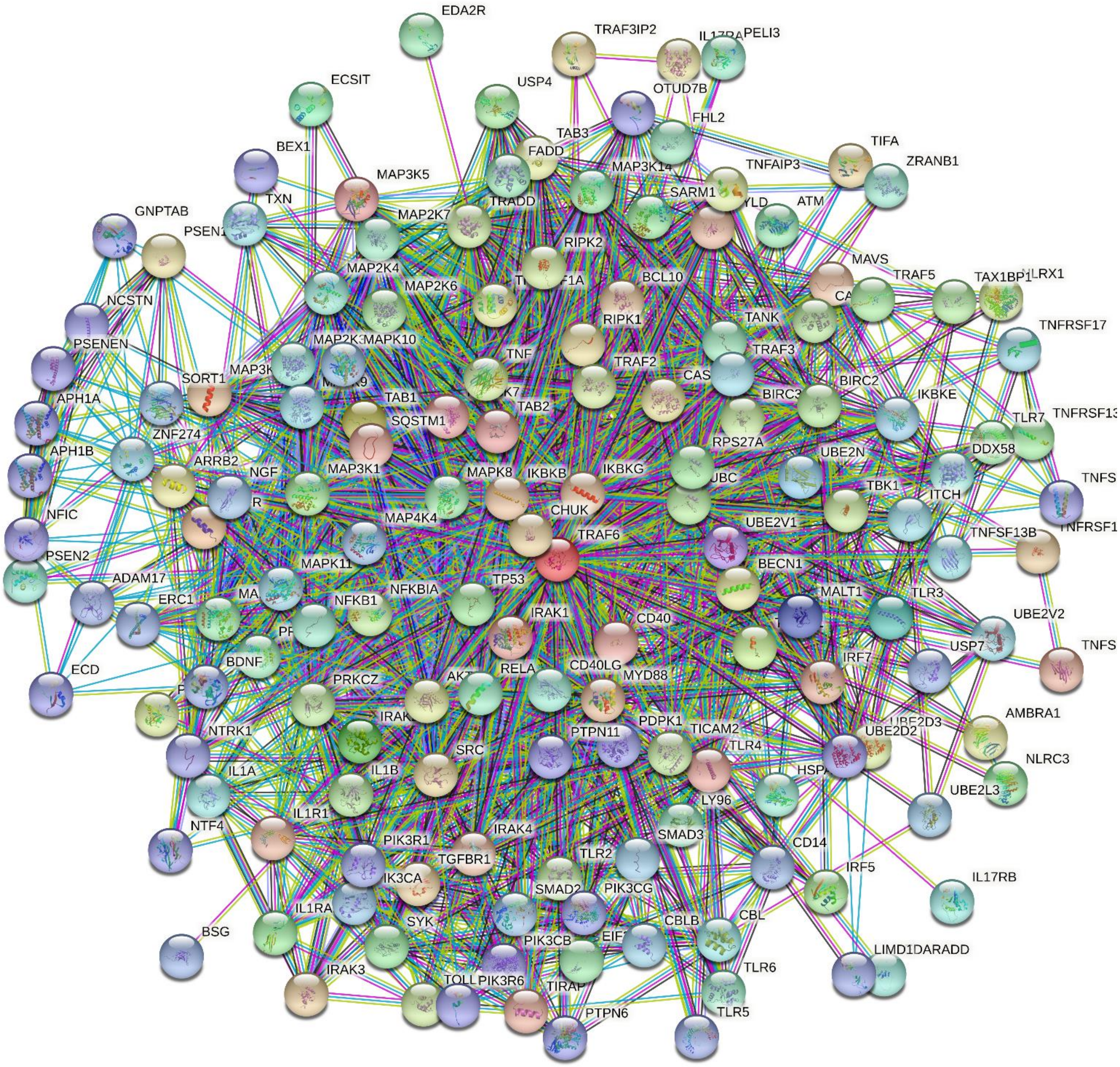

EDA2R
TRAF3IP2
IL17RA
PELI3
ECSIT
USP4
OTUD7B
FHL2
TAB3
BEX1
FADD
TIFA
MAP3K5
TNFAIP3
ZRANB1
MAP3K14
TXN
TRADD
SARM1
GNPTAB
MAP2K7
ATM
PSEN1
RIPK2
MAP2K4
MAVS
BCL10
TRAF5
TAX1BP1
NLRX1
MAP2K6
NCSTN
RIPK1
TANK
TNFRSF17
MAPK10
TNF
TRAF3
PSENEN
SORT1
TRAF2
APH1A
TAB1
BIRC3
BIRC2
IKBKE
SQSTM1
TAB2
TLR7
TNFRSF13
ZNF274
RPS27A
DDX58
APH1B
ARRB2
UBE2N
NGF
MAP3K1
IKBKG
MAPK8
IKBKB
UBC
TBK1
ITCH
NFIC
CHUK
UBE2V1
MAP4K4
TRAF6
TNFSF13B
PSEN2
MAPK11
BECN1
ADAM17
TP53
NFKBIA
ERC1
NFKB1
MALT1
TLR3
IRAK1
CD40
UBE2V2
USP7
BDNF
IRF7
ECD
RELA
CD40LG
MYD88
PRKCZ
AMBRA1
UBE2D3
NTRK1
PDPK1
TICAM2
UBE2D2
SRC
PTPN11
TLR4
NLRC3
IL1A
IL1B
LY96
UBE2L3
NTF4
IL1R1
IRAK4
SMAD3
CD14
PIK3R1
TGFBR1
TLR2
IRF5
IL17RB
SMAD2
PIK3CG
IL1RA
SYK
CBL
BSG
PIK3CB
CBLB
LIMD1DARADD
IRAK3
TLR6
PIK3R6
TIRAP
PTPN6
TLR5

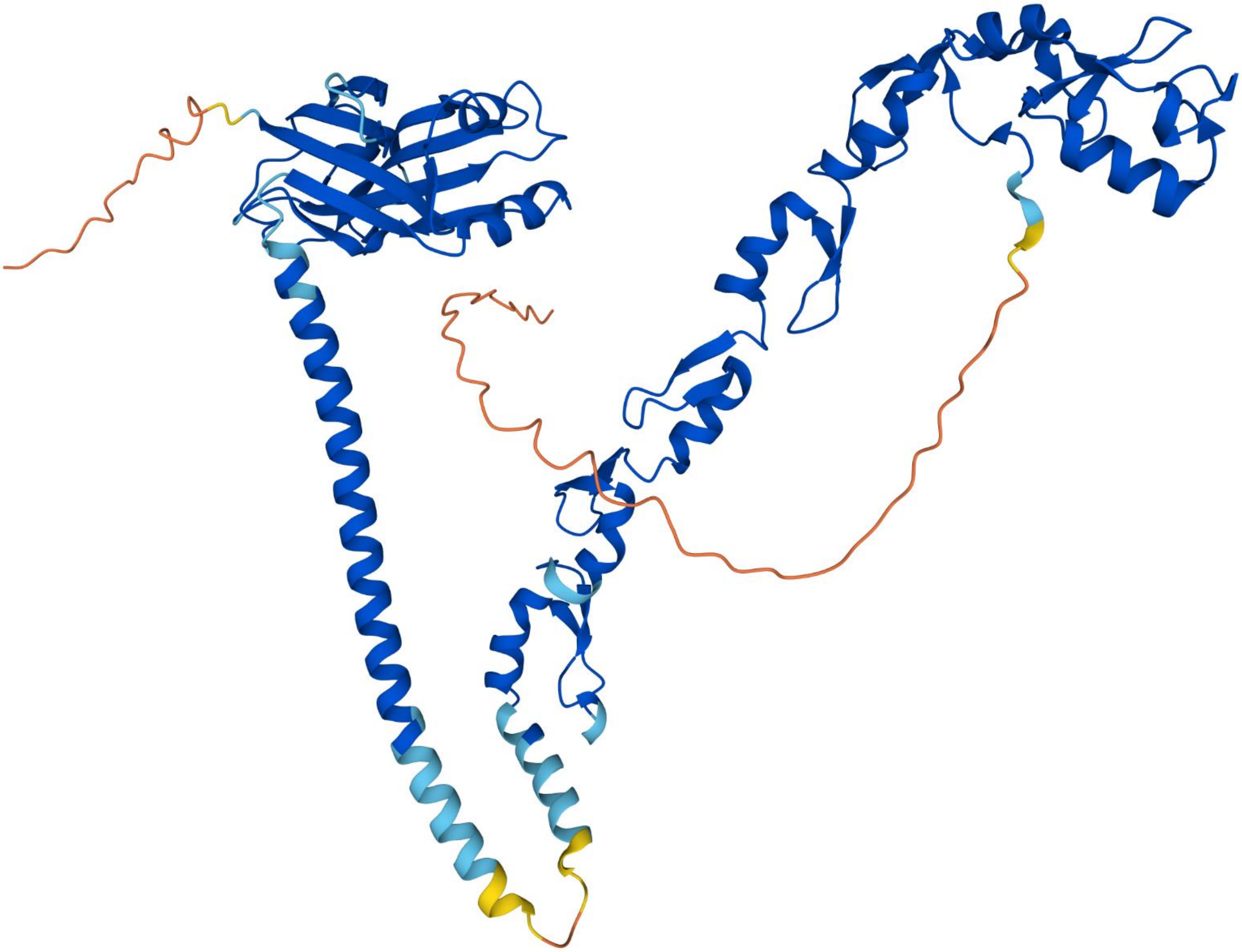

>sp|O43318|M3K7_HUMAN Mitogen-activated protein kinase kinase kinase 7 OS=Homo sapiens OX=9606 GN=MAP3K7 PE=1 SV=1
MSTASAASSSSSSSAGEMIEAPSQVLNFEEIDYKEIEVEEVVGRGAFGVVCKAKWRAKDVAIKQIESESERKAFIVELRQLSRVN
HPNIVKLYGACLNPVCLVMEYAEGGSLYNVLHGAEPLPYYTAAHAMSWCLQCSQGVAYLHSMQPKALIHRDLKPPNLLLVAGGTV
LKICDFGTACDIQTHMTNNKGSAAWMAPEVFEGSNYSEKCDVFSWGIILWEVITRRKPFDEIGGPAFRIMWAVHNGTRPPLIKNL
PKPIESLMTRCWSKDPSQRPSMEEIVKIMTHLMRYFPGADEPLQYPCQYSDEGQSNSATSTGSFMDIASTNTSNKSDTNMEQVPA
TNDTIKRLESKLLKNQAKQQSESGRLSLGASRGSSVESLPPTSEGKRMSADMSEIEARIAATTAYSKPKRGHRKTASFGNILDVP
EIVISGNGQPRRRSIQDLTVTGTEPGQVSSRSSSPSVRMITTSGPTSEKPTRSHPWTPDDSTDTNGSDNSIPMAYLTLDHQLQPL
APCPNSKESMAVFEQHCKMAQEYMKVQTEIALLLQRKQELVAELDQDEKDQQNTSRLVQEHKKLLDENKSLSTYYQQCKKQLEVI
RSQQQKRQGTS

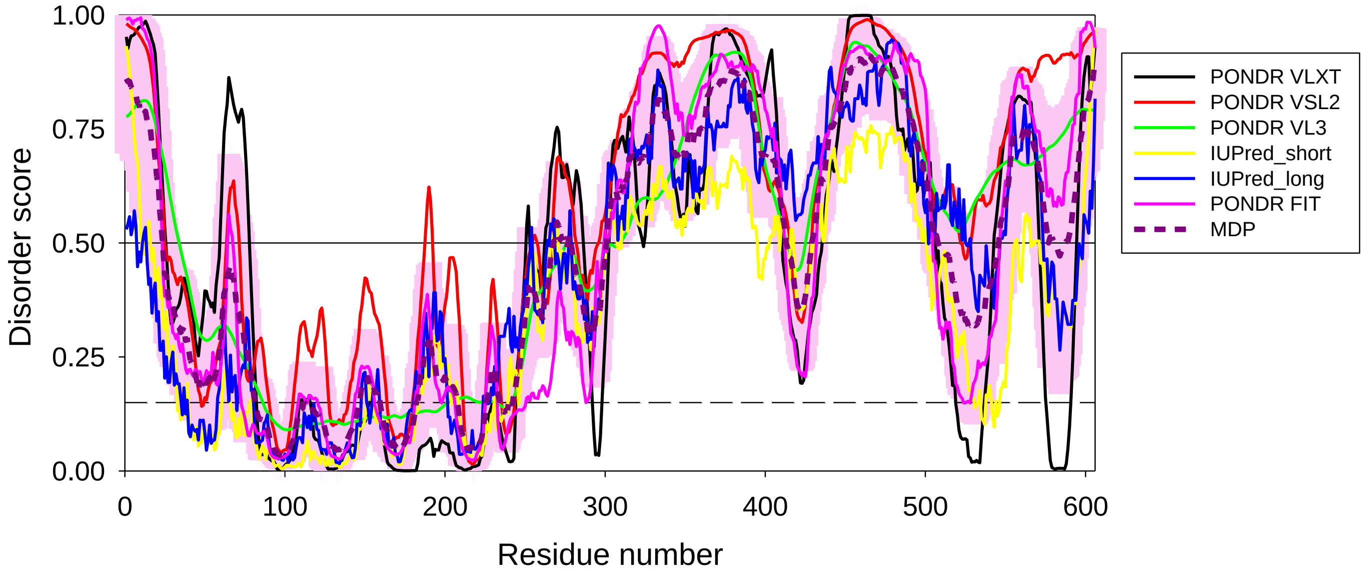

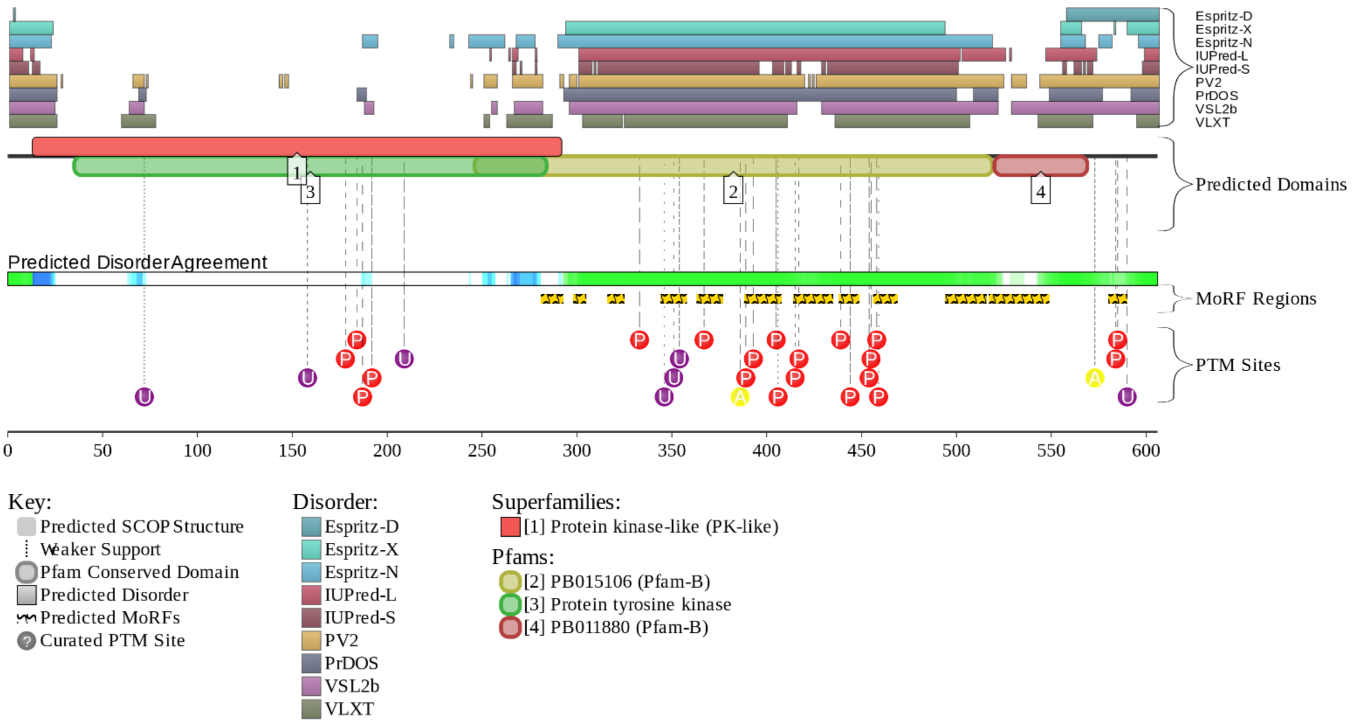

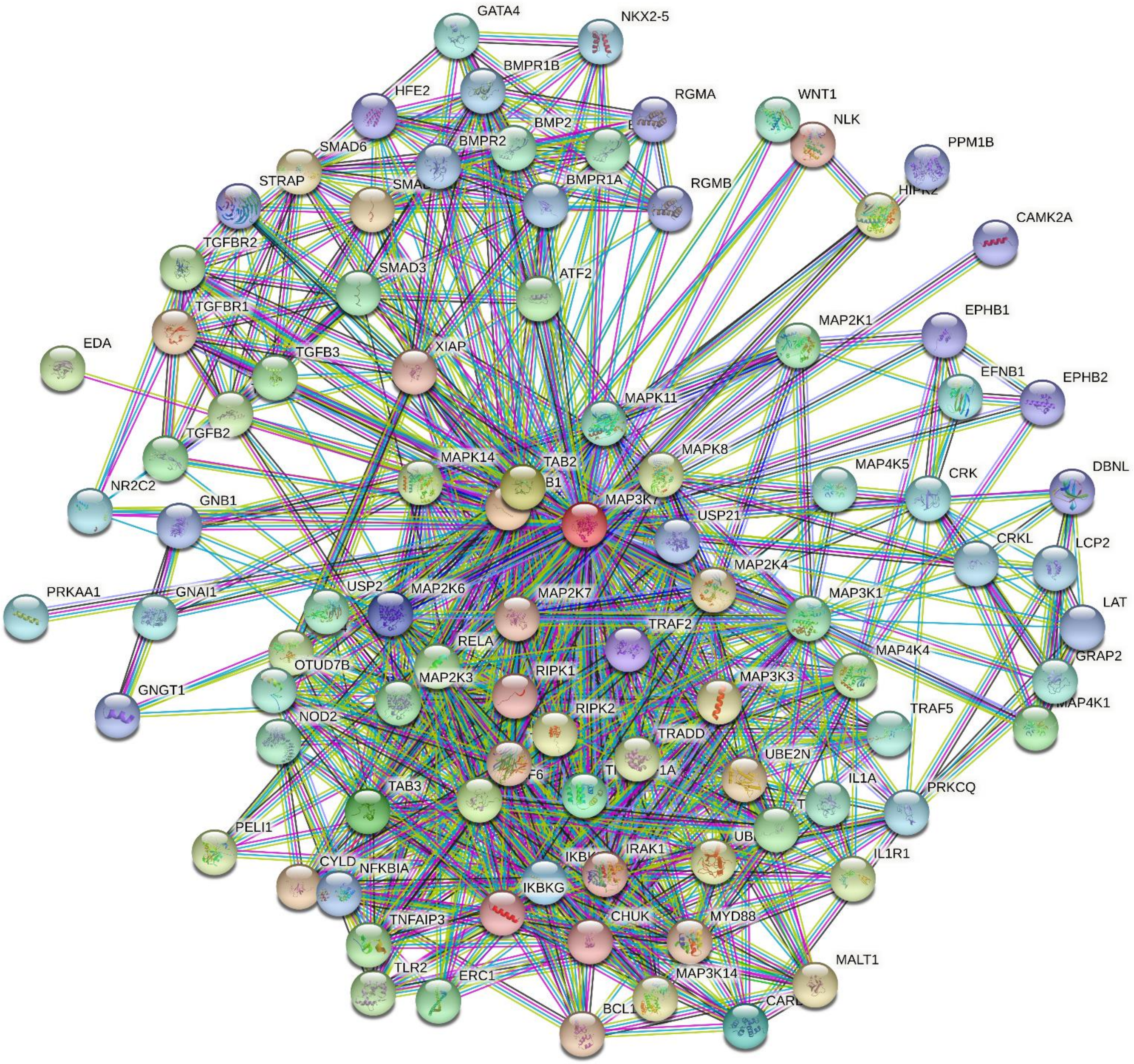
GATA4
NKX2-5
BMPR1B
HFE2
RGMA
WNT1
NLK
PPM1B
BMP2
BMPR2
SMAD6
STRAP
BMPR1A
RGMB
CAMK2A
TGFBR2
SMAD3
ATF2
TGFBR1
MAP2K1
EPHB1
EDA
TGFB3
XIAP
EFNB1
EPHB2
MAPK11
TGFB2
MAPK8
MAPK14
TAB2
MAP3K7
MAP4K5
CRK
DBNL
NR2C2
GNB1
USP21
CRKL
LCP2
MAP2K4
PRKAA1
GNAI1
USP2
MAP2K6
MAP2K7
MAP3K1
LAT
TRAF2
RELA
MAP4K4
GRAP2
OTUD7B
GNGT1
RIPK1
MAP2K3
MAP3K3
MAP4K1
TRAF5
NOD2
RIPK2
TRADD
UBE2N
IL1A
TAB3
PRKCQ
PELI1
IL1R1
CYLD
NFKBIA
IRAK1
IKBKG
CHUK
MYD88
TNFAIP3
MALT1
TLR2
ERC1
MAP3K14

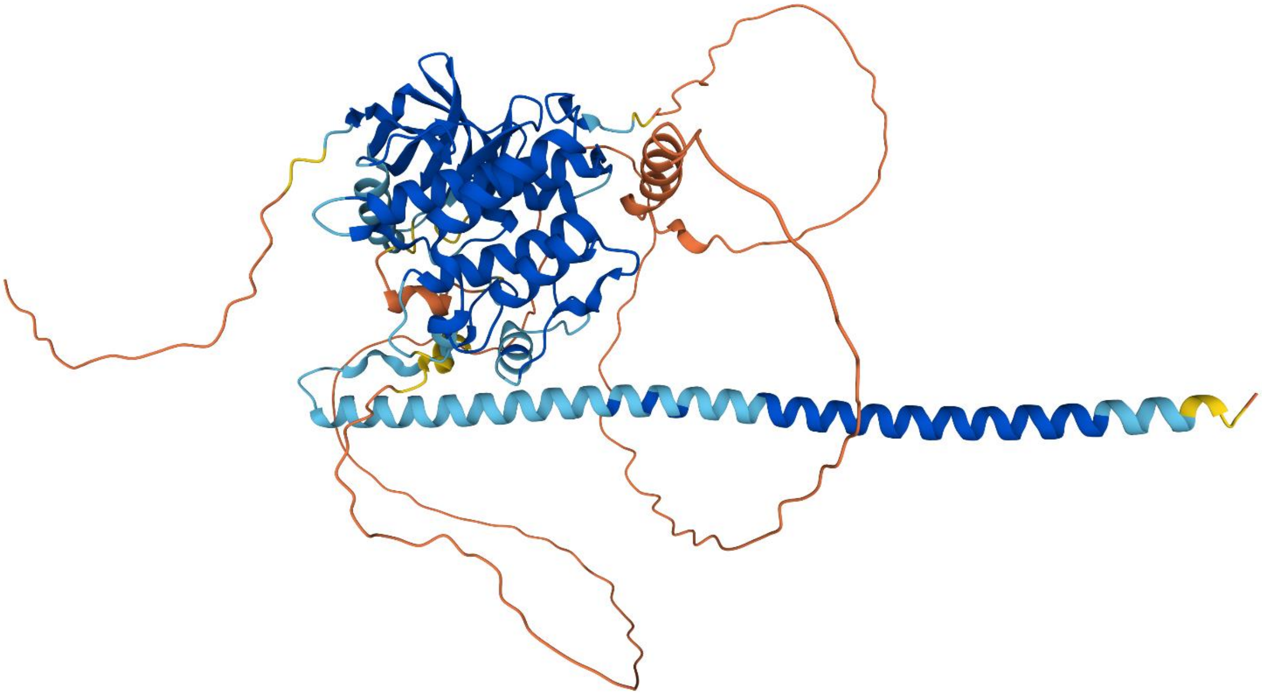

>sp|P28482|MK01_HUMAN Mitogen-activated protein kinase 1 OS=Homo sapiens OX=9606
GN=MAPK1 PE=1 SV=3
MAAAAAAGAGPEMVRGQVFDVGPRYTNLSYIGEGAYGMVCSAYDNVNKVRVAIKKISPFEHQTYCQRTLREIKILLRFRHENIIG
INDIIRAPTIEQMKDVYIVQDLMETDLYKLLKTQHLSNDHICYFLYQILRGLKYIHSANVLHRDLKPSNLLLNTTCDLKICDFGL
ARVADPDHDHTGFLTEYVATRWYRAPEIMLNSKGYTKSIDIWSVGCILAEMLSNRPIFPGKHYLDQLNHILGILGSPSQEDLNCI
INLKARNYLLSLPHKNKVPWNRLFPNADSKALDLLDKMLTFNPHKRIEVEQALAHPYLEQYYDPSDEPIAEAPFKFDMELDDLPK
EKLKELIFEETARFQPGYRS

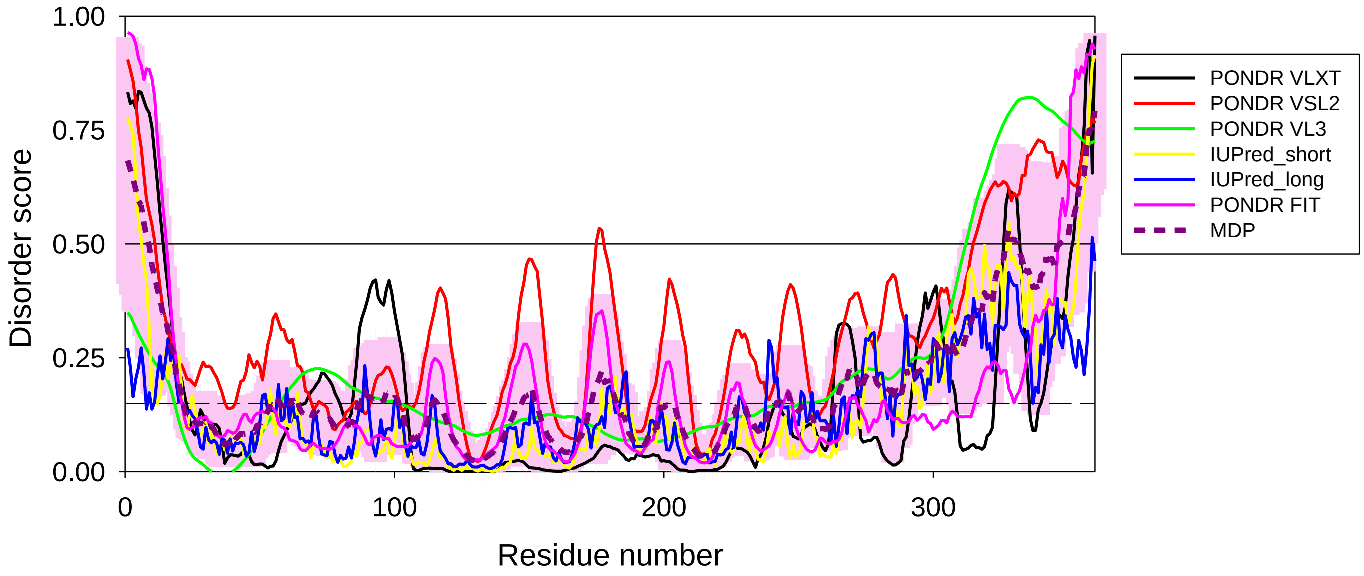

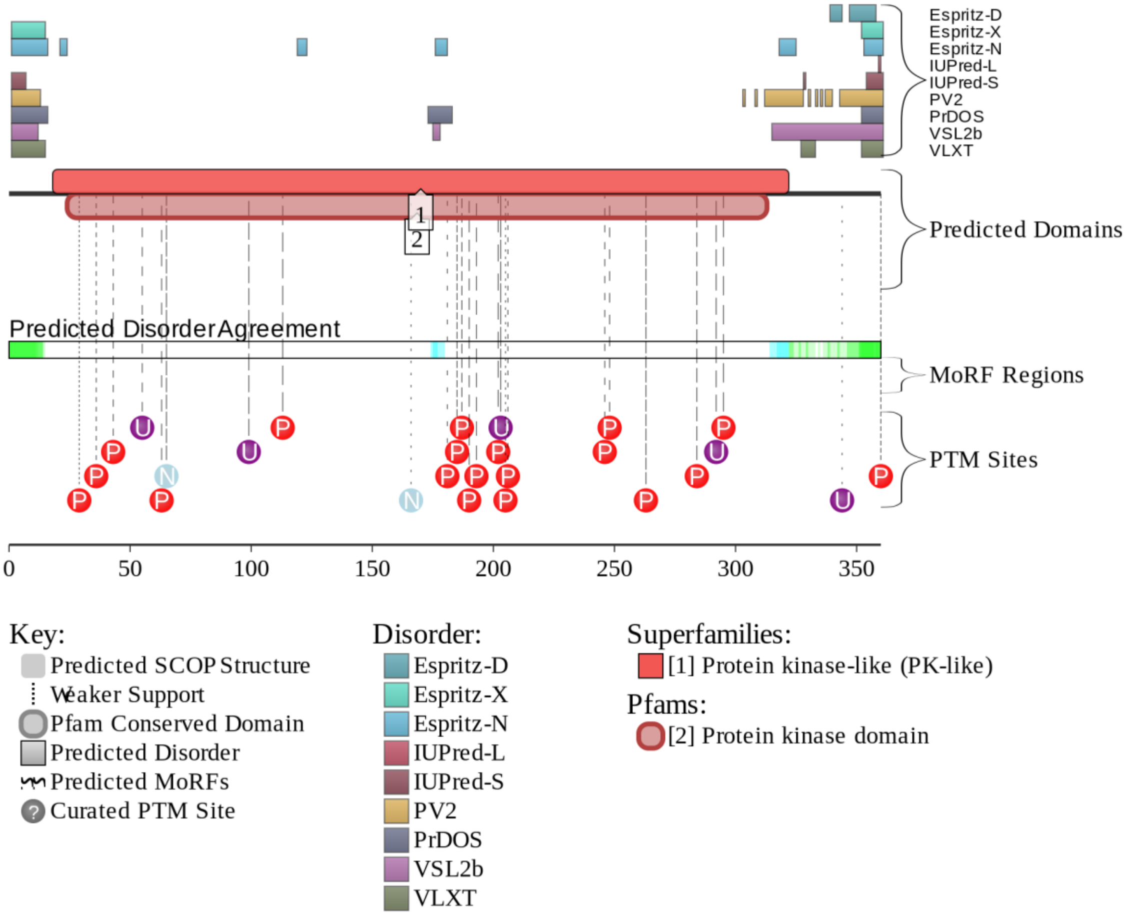

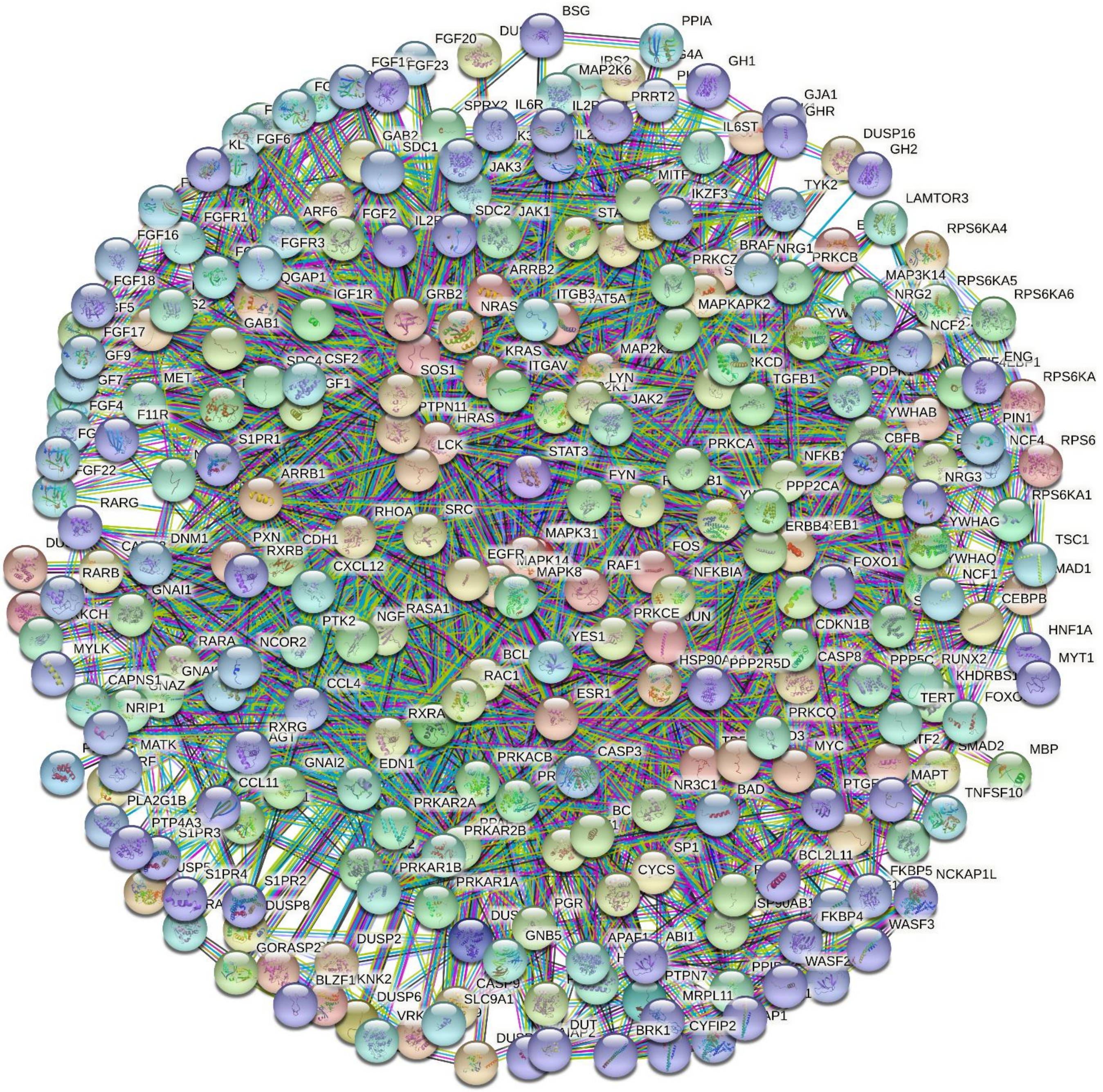
BSG
PPIA
FGF20
FGF23
MAP2K6
GH1
PRRT2
GJA1
GHR
SPRY2
IL6R
IL6ST
DUSP16
GH2
GAB2
SDC1
KL
FGF6
JAK3
MITF
IKZF3
TYK2
LAMTOR3
FGFR1
ARF6
FGF2
SDC2
JAK1
RPS6KA4
FGF16
FGFR3
BRAF
NRG1
PRKCB
PRKCZ
IQGAP1
ARRB2
MAP3K14
RPS6KA5
FGF18
IGF1R
GRB2
ITGB3
RPS6KA6
NRAS
MAPKAPK2
NRG2
GAB1
NCF2
FGF17
KRAS
IL2
ENG
CSF2
MAP2K2
MET
SOS1
ITGAV
LYN
RPS6KA
PRKCD
TGFB1
F11R
PTPN11
JAK2
FGF4
HRAS
YWHAB
PIN1
S1PR1
LCK
STAT3
CBFB
NCF4
RPS6
FGF22
PRKCA
NFKB1
ARRB1
FYN
NRG3
PPP2CA
RARG
RPS6KA1
RHOA
SRC
DNM1
PXN
CDH1
MAPK3
ERBB4
YWHAG
TSC1
RXRB
EGFR
MAPK14
FOS
YWHAQ
RARB
CXCL12
MAPK8
RAF1
NFKBIA
FOXO1
NCF1
GNAI1
CEBPB
RASA1
PRKCE
JUN
CDKN1B
HNF1A
MYLK
RARA
NCOR2
PTK2
YES1
MYT1
PPP2R5D
CASP8
RUNX2
CAPNS1
GNAZ
CCL4
RAC1
KHDRBS1
TERT
NRIP1
ESR1
PRKCQ
RXRG
RXRA
MYC
MATK
SMAD2
MBP
CASP3
PRKACB
GNAI2
EDN1
MAPT
CCL11
NR3C1
BAD
TNFSF10
PLA2G1B
PRKAR2A
PRKAR2B
PTP4A3
S1PR3
SP1
BCL2L11
CYCS
FKBP5
NCKAP1L
S1PR4
PRKAR1B
S1PR2
PRKAR1A
DUSP8
PGR
FKBP4
WASF3
GNB5
ABI1
DUSP2
APAF1
GORASP2
WASF2
BLZF1
PTPN7
DUSP6
CASP9
SLC9A1
MRPL11
CYFIP2
DUT
BRK1

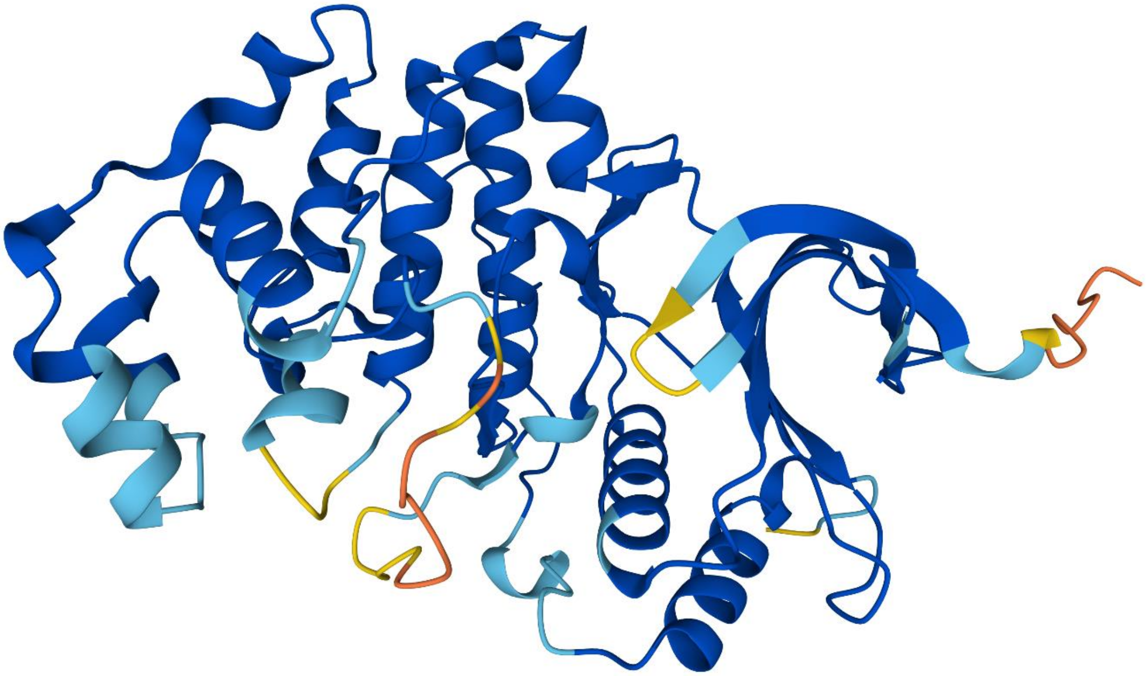

>sp|P27361|MK03_HUMAN Mitogen-activated protein kinase 3 OS=Homo sapiens OX=9606 GN=MAPK3 PE=1 SV=4
MAAAAAQGGGGGEPRRTEGVGPGVPGEVEMVKGQPFDVGPRYTQLQYIGEGAYGMVSSAYDHVRKTRVAIKKISPFEHQTYCQRT
LREIQILLRFRHENVIGIRDILRASTLEAMRDVYIVQDLMETDLYKLLKSQQLSNDHICYFLYQILRGLKYIHSANVLHRDLKPS
NLLINTTCDLKICDFGLARIADPEHDHTGFLTEYVATRWYRAPEIMLNSKGYTKSIDIWSVGCILAEMLSNRPIFPGKHYLDQLN
HILGILGSPSQEDLNCIINMKARNYLQSLPSKTKVAWAKLFPKSDSKALDLLDRMLTFNPNKRITVEEALAHPYLEQYYDPTDEP
VAEEPFTFAMELDDLPKERLKELIFQETARFQPGVLEAP

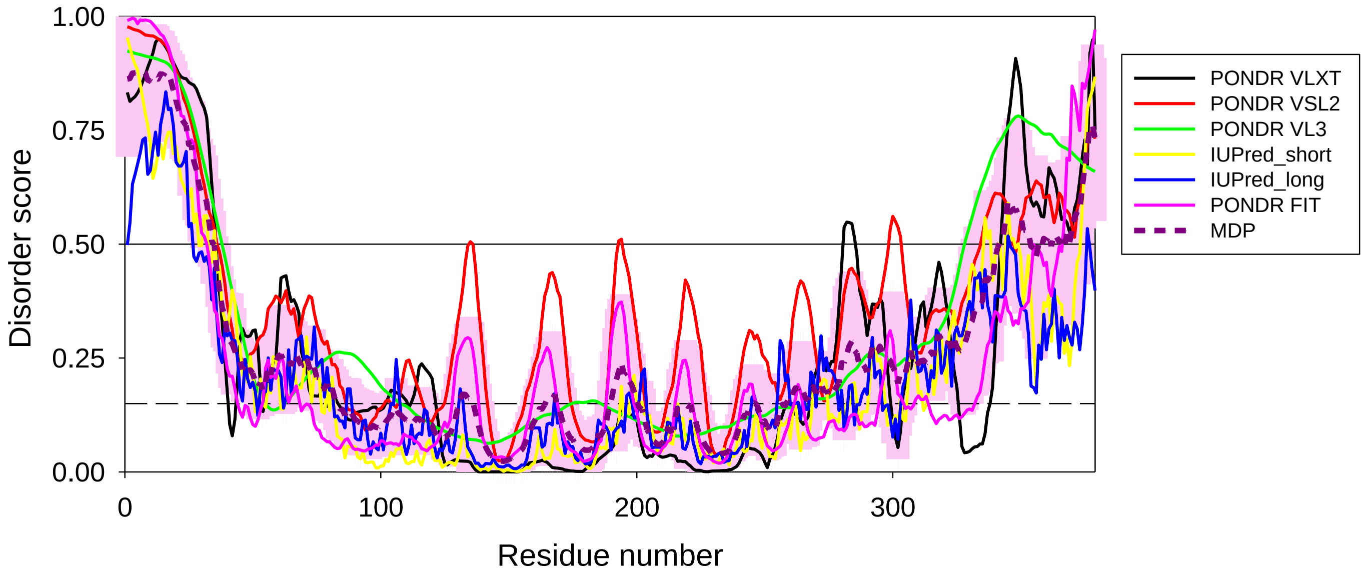

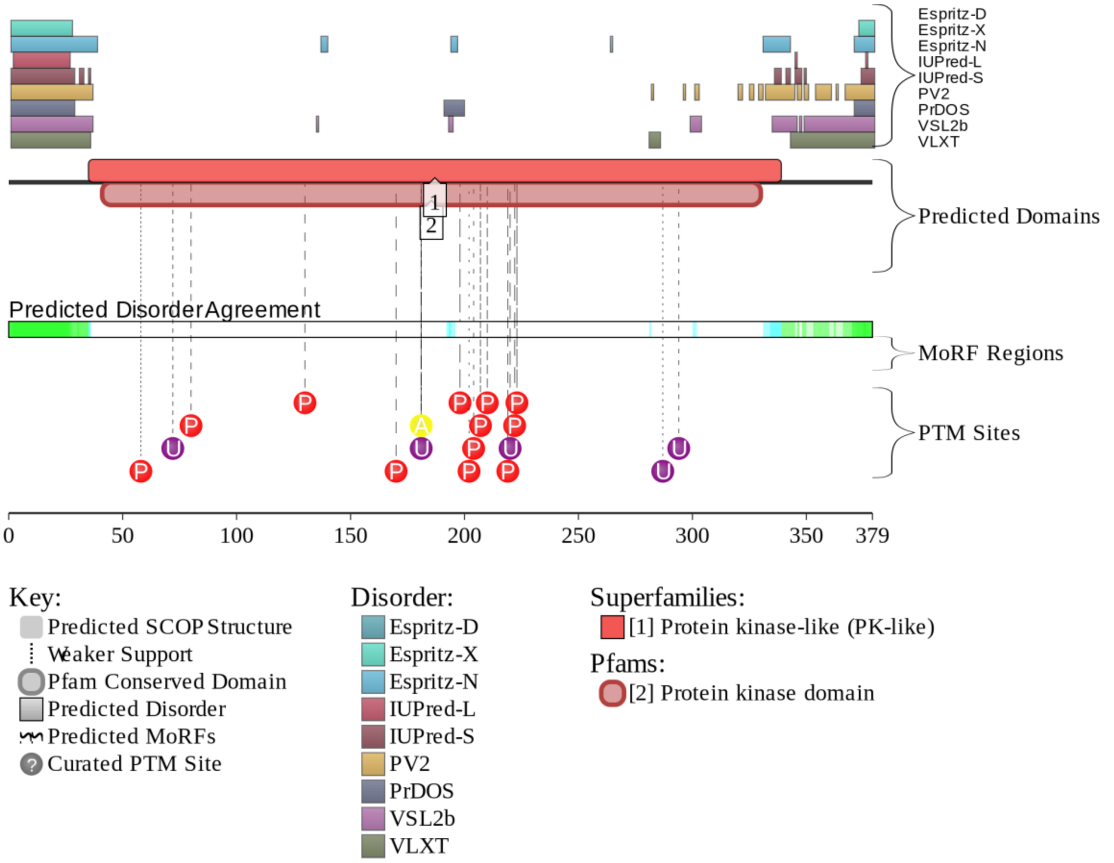

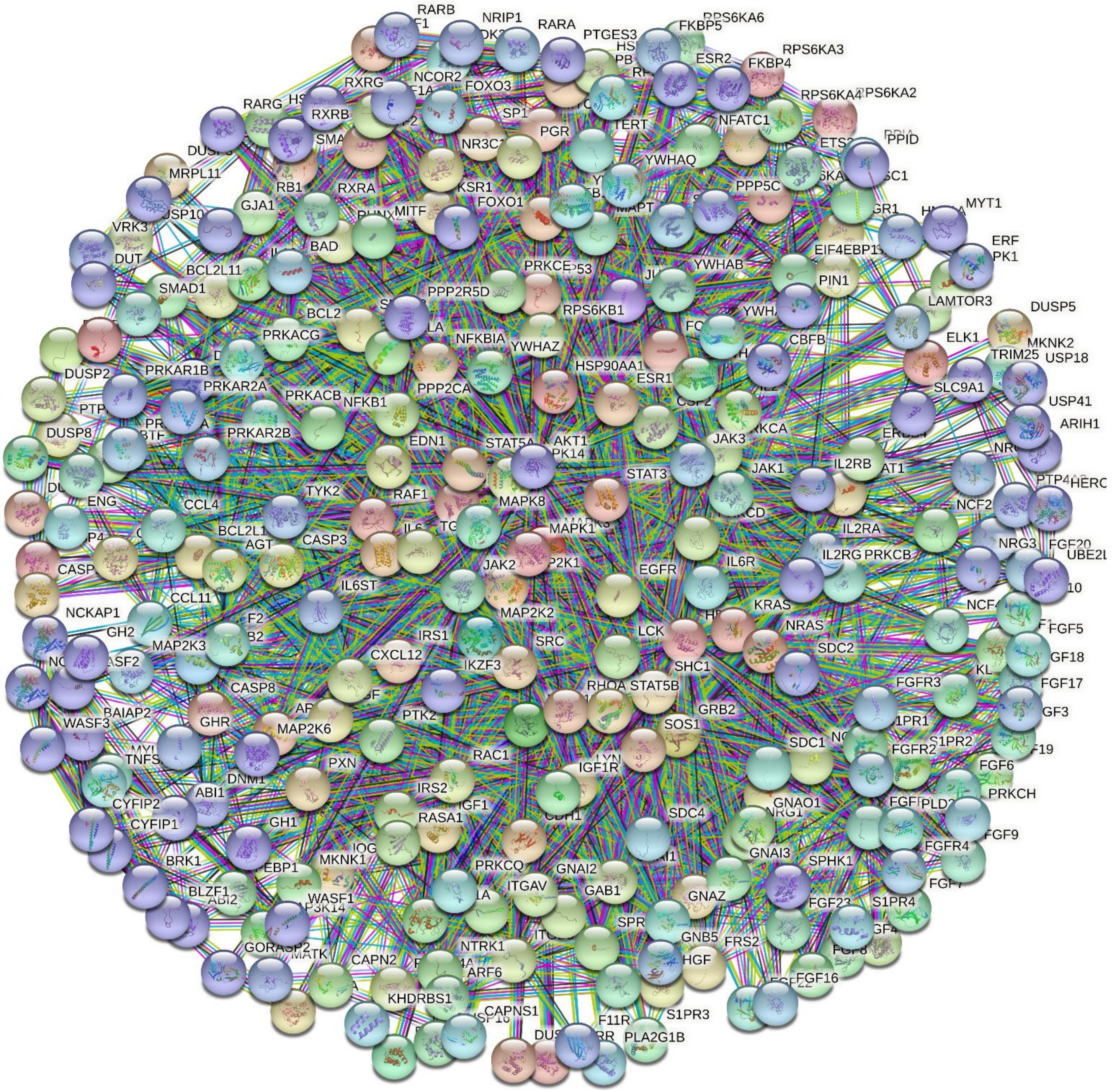
RARB
NRIP1
RARA
PTGES3
FKBP5
RPS6KA6
RPS6KA3
ESR2
FKBP4
RXRG
NCOR2
FOXO3
RPS6KA4
RPS6KA2
RARG
RXRB
SP1
PGR
TERT
NFATC1
PPID
NR3C1
YWHAQ
MRPL11
PPP5C
MYT1
RB1
RXRA
KSR1
FOXO1
GJA1
MITF
MAPT
ERF
VRK3
DUT
BAD
BCL2L11
PRKCE
YWHAB
PIN1
SMAD1
PPP2R5D
LAMTOR3
DUSP5
RPS6KB1
BCL2
PRKACG
NFKBIA
YWHAZ
CBFB
ELK1
MKNK2
TRIM25
USP18
HSP90AA1
DUSP2
PRKAR1B
ESR1
SLC9A1
PRKAR2A
PRKACB
NFKB1
PPP2CA
USP41
ARIH1
DUSP8
PRKAR2B
JAK3
EDN1
STAT5A
AKT1
ENG
TYK2
RAF1
MAPK8
STAT3
JAK1
IL2RB
CCL4
NCF2
BCL2L1
AGT
CASP3
MAPK1
IL2RA
NRG3
FGF20
IL2RG
PRKCB
JAK2
IL6R
EGFR
IL6ST
CCL11
NCKAP1
MAP2K2
KRAS
NRAS
FGF5
GH2
IRS1
SRC
LCK
SDC2
MAP2K3
CXCL12
IKZF3
FGF18
SHC1
FGF17
CASP8
RHOA
STAT5B
FGFR3
FGF3
GRB2
BAIAP2
GHR
MAP2K6
PTK2
S1PR1
WASF3
SOS1
SDC1
FGFR2
S1PR2
FGF19
PXN
RAC1
IGF1R
FGF6
DNM1
ABI1
IRS2
PRKCH
CYFIP2
GNAO1
IGF1
CDH1
CYFIP1
RASA1
SDC4
NRG1
GH1
FGF9
BRK1
MKNK1
SPHK1
GNAI3
FGFR4
PRKCQ
GNAI2
ITGAV
GAB1
BLZF1
WASF1
GNAZ
FGF23
S1PR4
GNB5
FRS2
HGF
GORASP2
MATK
NTRK1
CAPN2
ARF6
FGF16
KHDRBS1
CAPNS1
F11R
S1PR3
PLA2G1B

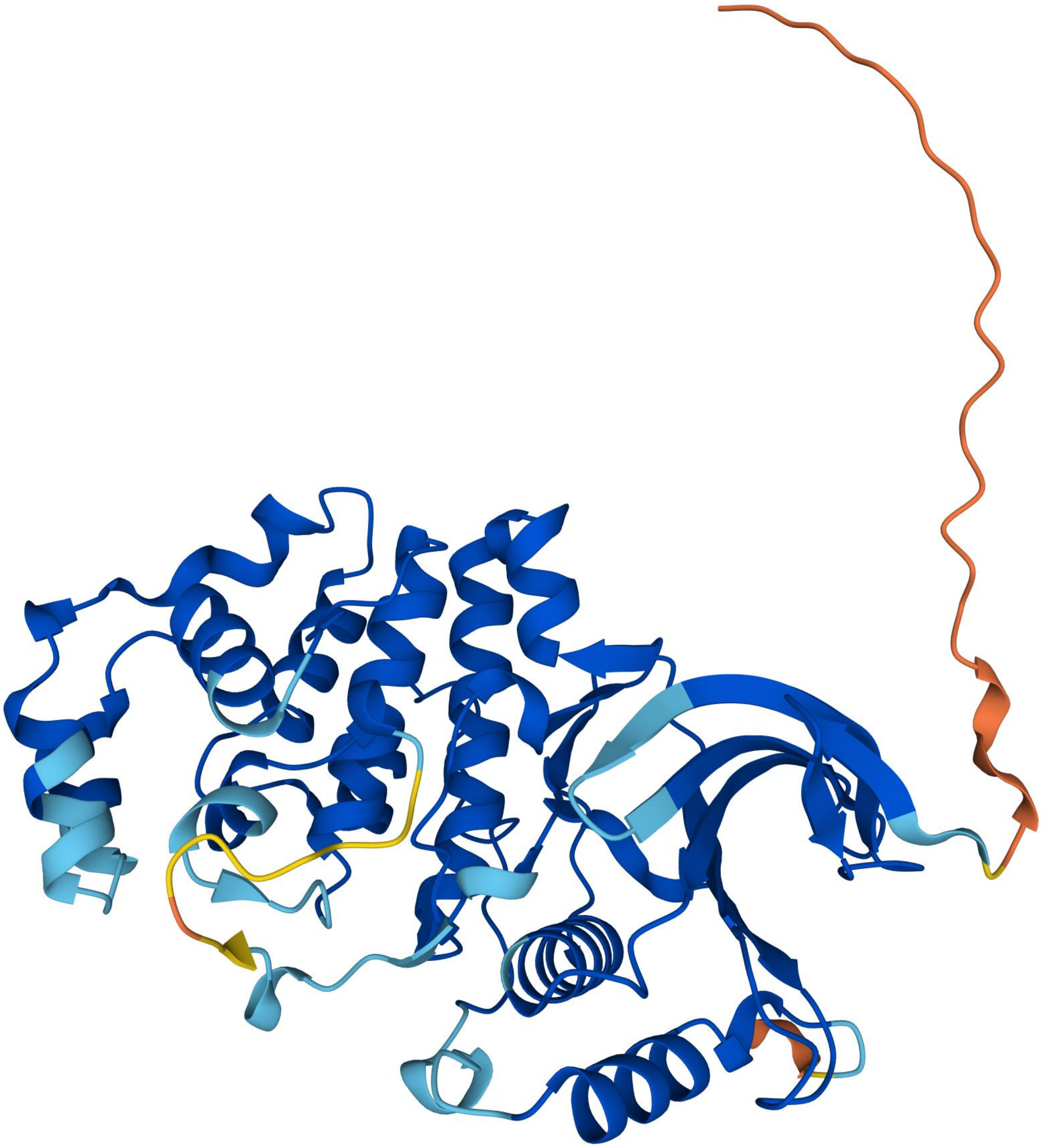

>sp|P31152|MK04_HUMAN Mitogen-activated protein kinase 4 OS=Homo sapiens OX=9606 GN=MAPK4 PE=1 SV=2
MAEKGDCIASVYGYDLGGRFVDFQPLGFGVNGLVLSAVDSRACRKVAVKKIALSDARSMKHALREIKIIRRLDHDNIVKVYEVLG
PKGTDLQGELFKFSVAYIVQEYMETDLARLLEQGTLAEEHAKLFMYQLLRGLKYIHSANVLHRDLKPANIFISTEDLVLKIGDFG
LARIVDQHYSHKGYLSEGLVTKWYRSPRLLLSPNNYTKAIDMWAAGCILAEMLTGRMLFAGAHELEQMQLILETIPVIREEDKDE
LLRVMPSFVSSTWEVKRPLRKLLPEVNSEAIDFLEKILTFNPMDRLTAEMGLQHPYMSPYSCPEDEPTSQHPFRIEDEIDDIVLM
AANQSQLSNWDTCSSRYPVSLSSDLEWRPDRCQDASEVQRDPRAGSAPLAEDVQVDPRKDSHSSSERFLEQSHSSMERAFEADYG
RSCDYKVGSPSYLDKLLWRDNKPHHYSEPKLILDLSHWKQAAGAPPTATGLADTGAREDEPASLFLEIAQWVKSTQGGPEHASPP
ADDPERRLSASPPGRPAPVDGGASPQFDLDVFISRALKLCTKPEDLPDNKLGDLNGACIPEHPGDLVQTEAFSKERW

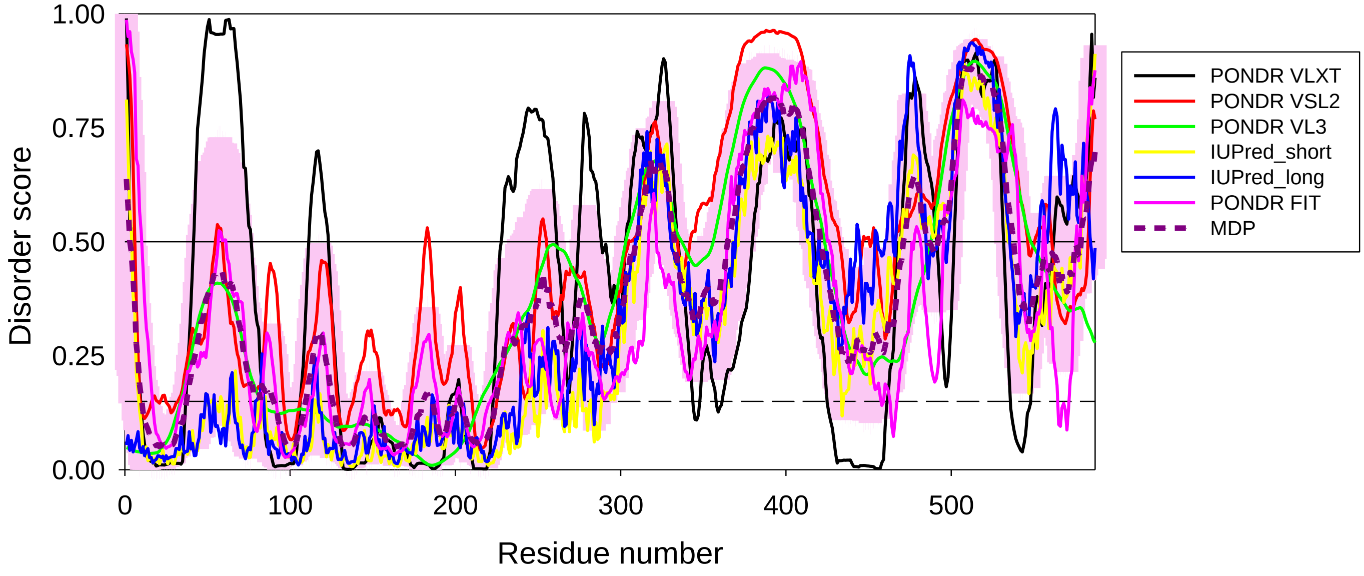

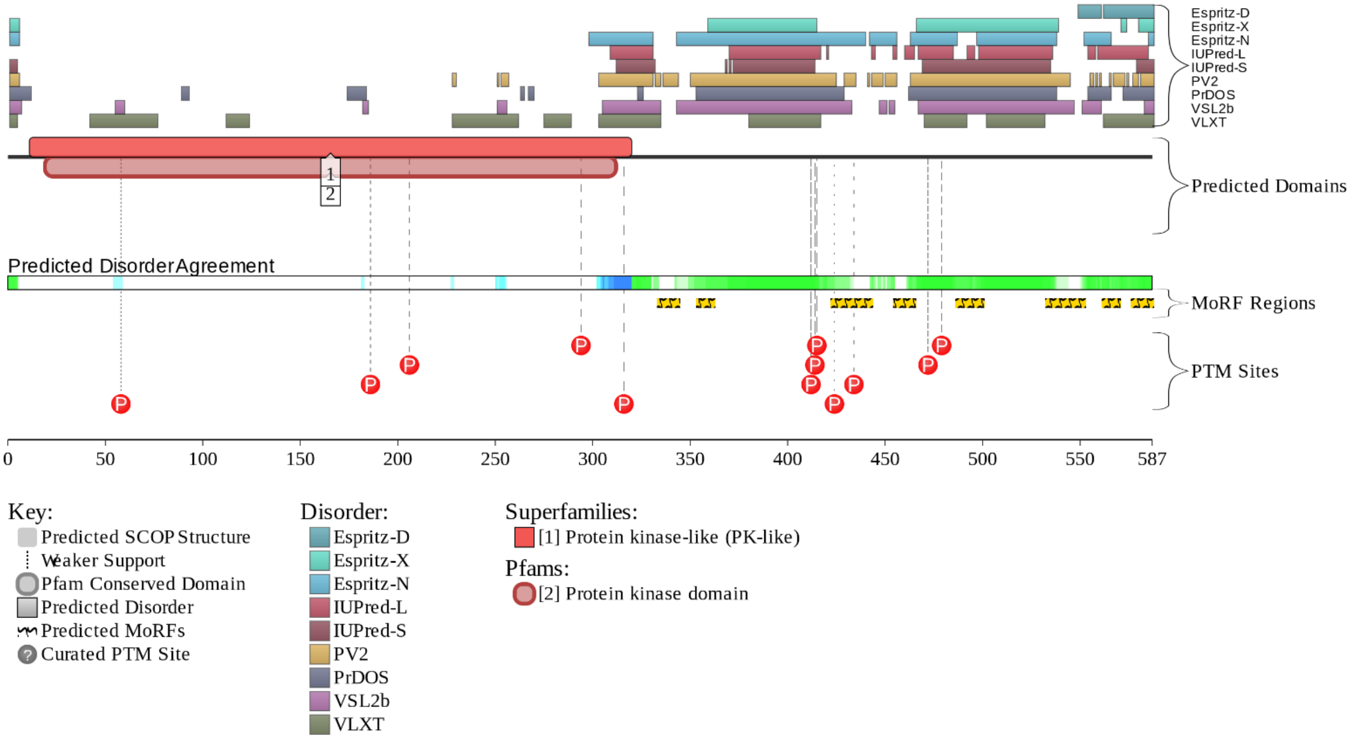

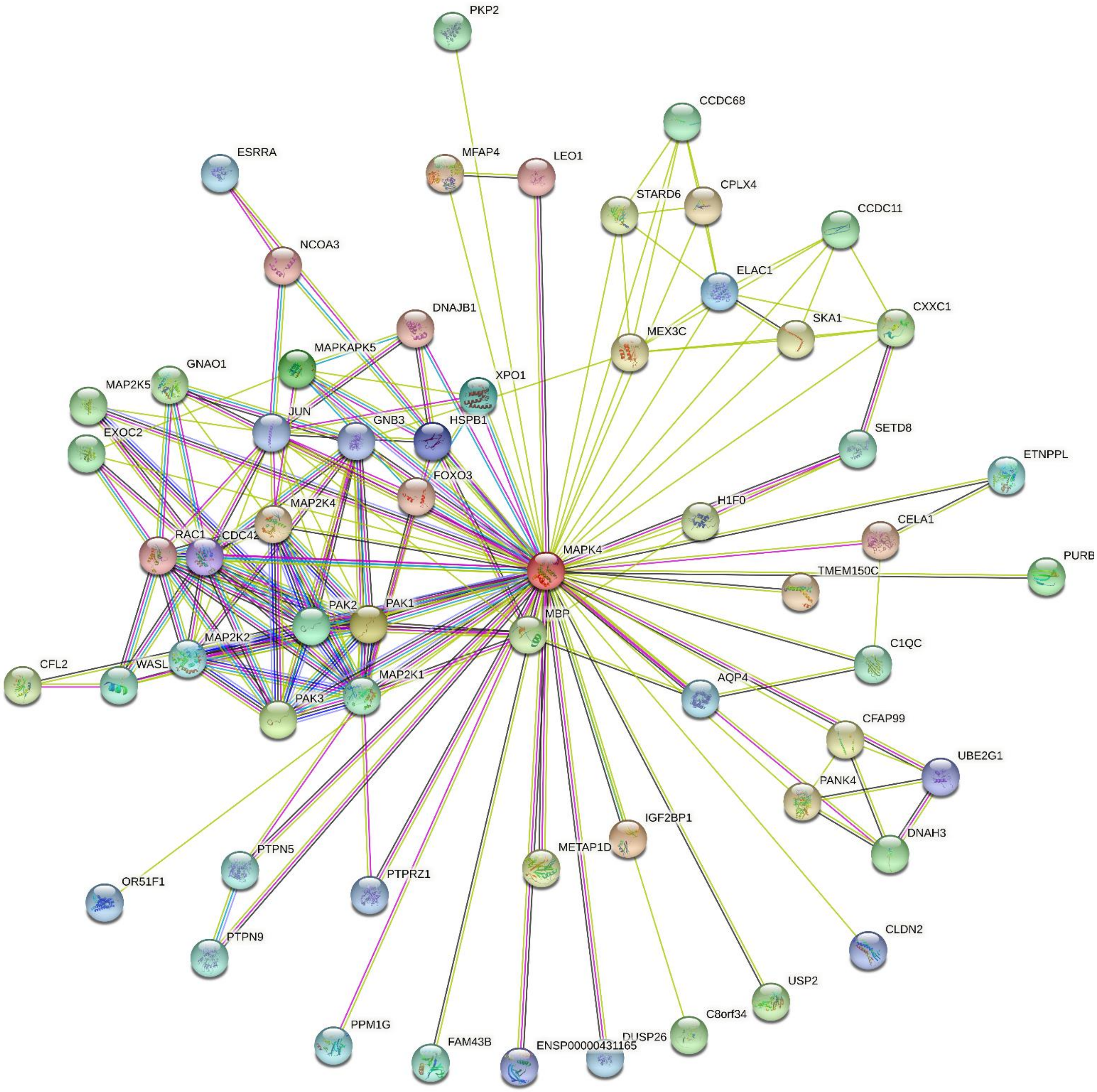
PKP2
CCDC68
ESRRA
MFAP4
LEO1
CPLX4
STARD6
CCDC11
NCOA3
ELAC1
DNAJB1
SKA1
CXXC1
MEX3C
MAPKAPK5
GNAO1
XPO1
MAP2K5
JUN
GNB3
HSPB1
SETD8
EXOC2
ETNPPL
FOXO3
H1F0
MAP2K4
CELA1
RAC1
CDC42
MAPK4
PURB
TMEM150C
PAK2
PAK1
MBP
C1QC
MAP2K2
CFL2
WASL
AQP4
PAK3
MAP2K1
CFAP99
UBE2G1
PANK4
IGF2BP1
DNAH3
PTPN5
METAP1D
OR51F1
PTPRZ1
PTPN9
CLDN2
USP2
PPM1G
C8orf34
FAM43B
ENSP00000431165
DUSP26

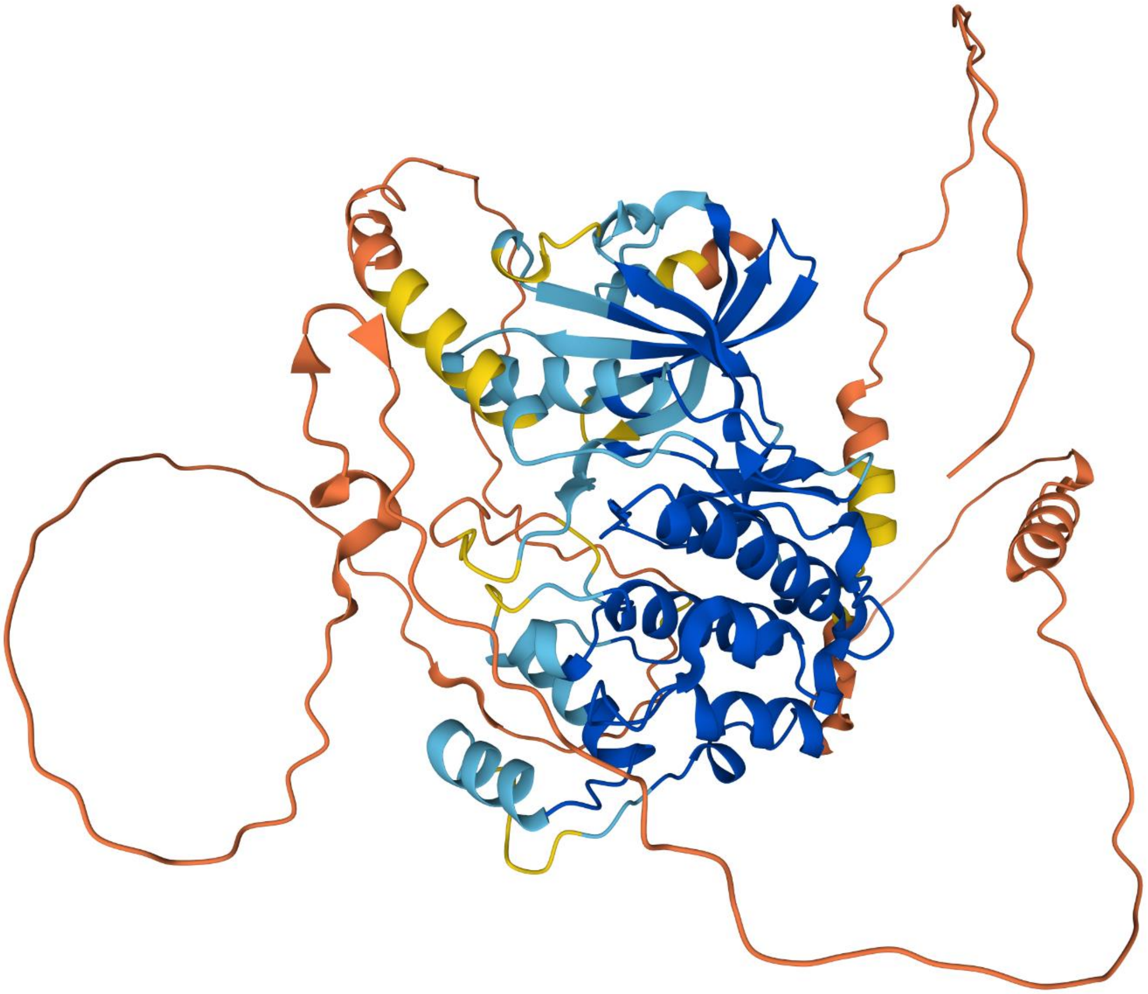

>sp|Q16659|MK06_HUMAN Mitogen-activated protein kinase 6 OS=Homo sapiens OX=9606 GN=MAPK6 PE=1 SV=1

MAEKFESLMNIHGFDLGSRYMDLKPLGCGGNGLVFSAVDNDCDKRVAIKKIVLTDPQSVKHALREIKIIRRLDHDNIVKVFEILG
PSGSQLTDDVGSLTELNSVYIVQEYMETDLANVLEQGPLLEEHARLFMYQLLRGLKYIHSANVLHRDLKPANLFINTEDLVLKIG
DFGLARIMDPHYSHKGHLSEGLVTKWYRSPRLLLSPNNYTKAIDMWAAGCIFAEMLTGKTLFAGAHELEQMQLILESIPVVHEED
RQELLSVIPVYIRNDMTEPHKPLTQLLPGISREALDFLEQILTFSPMDRLTAEEALSHPYMSIYSFPMDEPISSHPFHIEDEVDD
ILLMDETHSHIYNWERYHDCQFSEHDWPVHNNFDIDEVQLDPRALSDVTDEEEVQVDPRKYLDGDREKYLEDPAFDTNYSTEPCW
QYSDHHENKYCDLECSHTCNYKTRSSSYLDNLVWRESEVNHYYEPKLIIDLSNWKEQSKEKSDKKGKSKCERNGLVKAQIALEEA
SQQLAGKEREKNQGFDFDSFIAGTIQLSSQHEPTDVVDKLNDLNSSVSQLELKSLISKSVSQEKQEKGMANLAQLEALYQSSWDS
QFVSGGEDCFFINQFCEVRKDEQVEKENTYTSYLDKFFSRKEDTEMLETEPVEDGKLGERGHEEGFLNNSGEFLFNKQLESIGIP
QFHSPVGSPLKSIQATLTPSAMKSSPQIPHQTYSSILKHLN

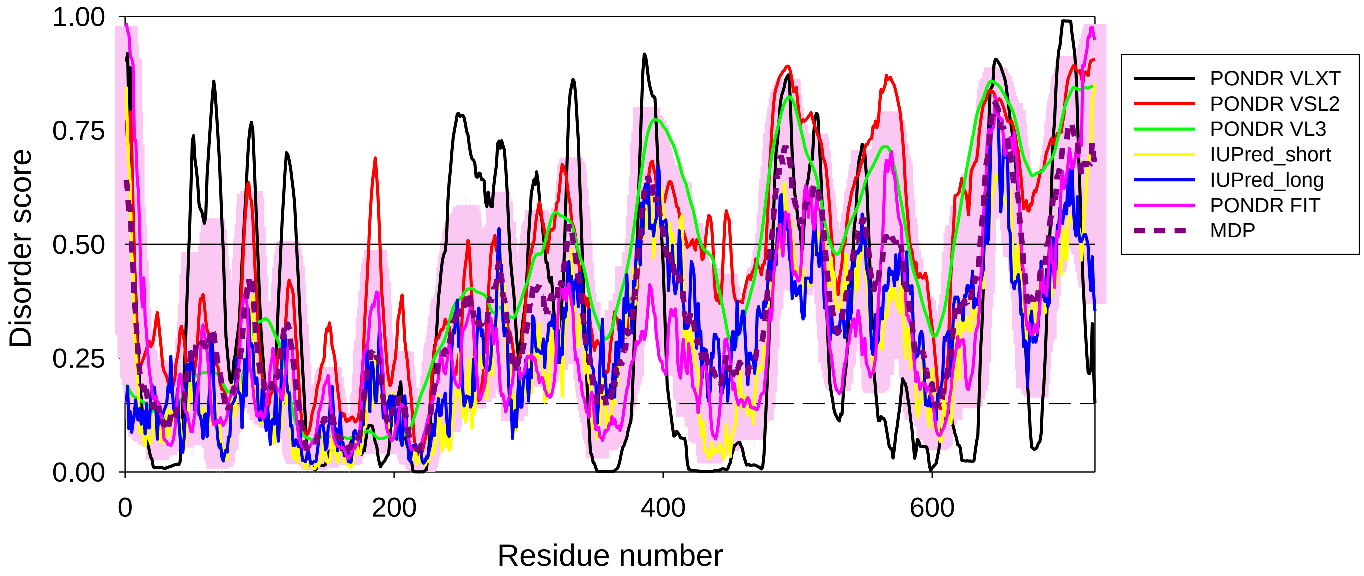

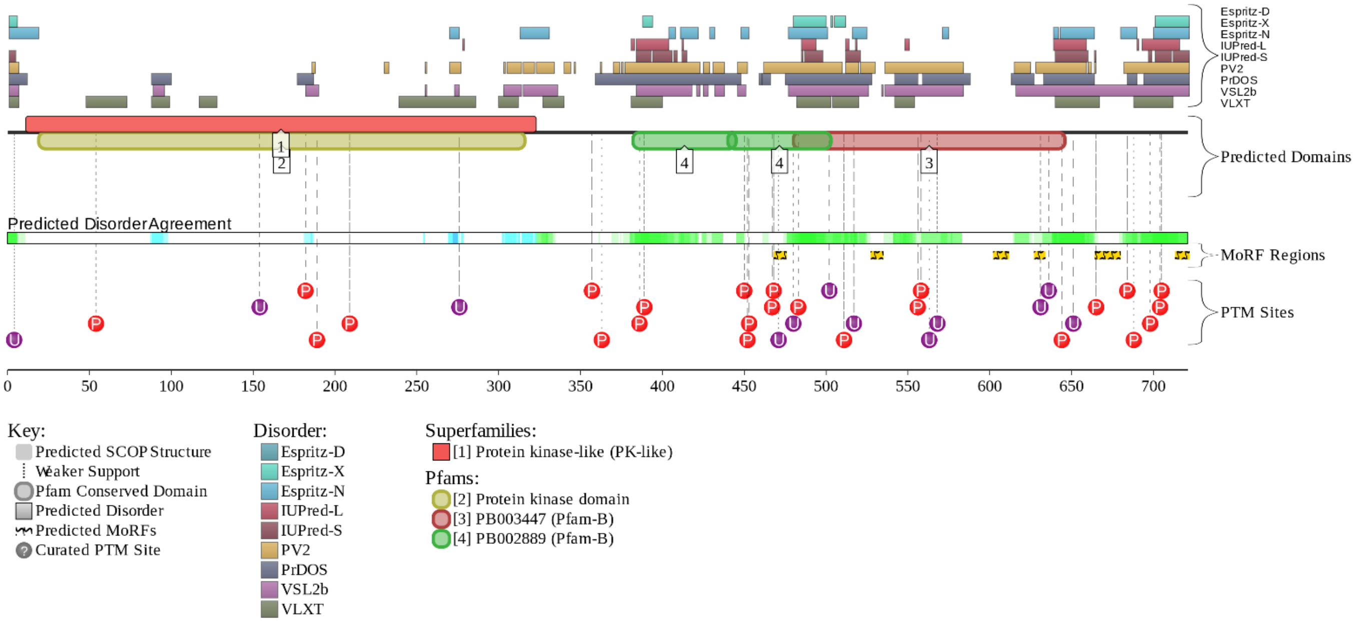

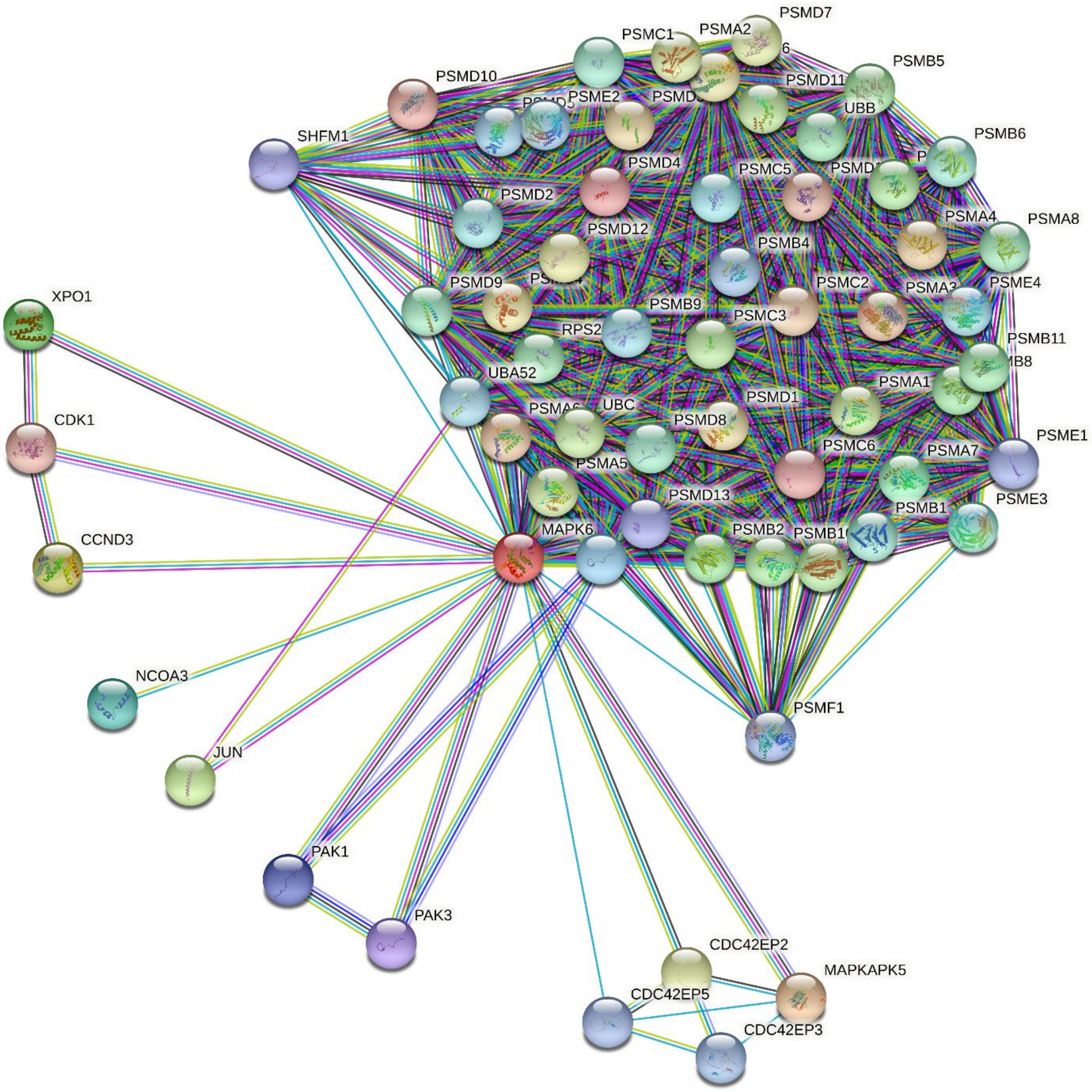
PSMD7
PSMC1
PSMA2
PSMB5
PSMD10
PSMD11
PSME2
UBB
SHFM1
PSMB6
PSMD4
PSMC5
PSMD2
PSMA4
PSMA8
PSMD12
PSMB4
XPO1
PSMD9
PSMC2
PSME4
PSMB9
PSMC3
PSMB11
UBA52
PSMA1
CDK1
UBC
PSMD1
PSMD8
PSME1
PSMC6
PSMA7
PSMA5
PSMD13
PSME3
PSMB1
MAPK6
PSMB2
CCND3
NCOA3
PSMF1
JUN
PAK1
PAK3
CDC42EP2
MAPKAPK5
CDC42EP5
CDC42EP3

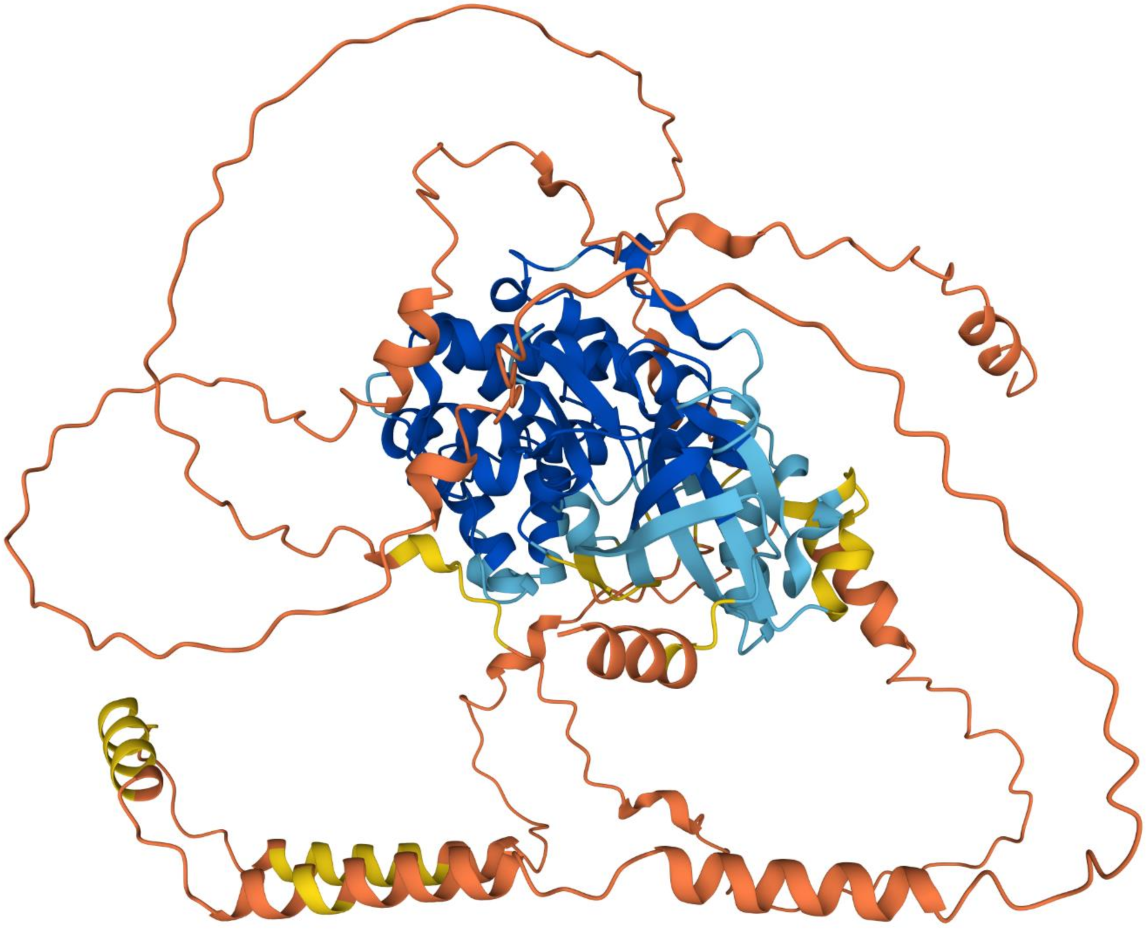

>sp|Q13164|MK07_HUMAN Mitogen-activated protein kinase 7 OS=Homo sapiens OX=9606
GN=MAPK7 PE=1 SV=2
MAEPLKEEDGEDGSAEPPGPVKAEPAHTAASVAAKNLALLKARSFDVTFDVGDEYEIIETIGNGAYGVVSSARRRLTGQQVAIKK
IPNAFDVVTNAKRTLRELKILKHFKHDNIIAIKDILRPTVPYGEFKSVYVVLDLMESDLHQIIHSSQPLTLEHVRYFLYQLLRGL
KYMHSAQVIHRDLKPSNLLVNENCELKIGDFGMARGLCTSPAEHQYFMTEYVATRWYRAPELMLSLHEYTQAIDLWSVGCIFGEM
LARRQLFPGKNYVHQLQLIMMVLGTPSPAVIQAVGAERVRAYIQSLPPRQPVPWETVYPGADRQALSLLGRMLRFEPSARISAAA
ALRHPFLAKYHDPDDEPDCAPPFDFAFDREALTRERIKEAIVAEIEDFHARREGIRQQIRFQPSLQPVASEPGCPDVEMPSPWAP
SGDCAMESPPPAPPPCPGPAPDTIDLTLQPPPPVSEPAPPKKDGAISDNTKAALKAALLKSLRSRLRDGPSAPLEAPEPRKPVTA
QERQREREEKRRRRQERAKEREKRRQERERKERGAGASGGPSTDPLAGLVLSDNDRSLLERWTRMARPAAPALTSVPAPAPAPTP
TPTPVQPTSPPPGPVAQPTGPQPQSAGSTSGPVPQPACPPPGPAPHPTGPPGPIPVPAPPQIATSTSLLAAQSLVPPPGLPGSST
PGVLPYFPPGLPPPDAGGAPQSSMSESPDVNLVTQQLSKSQVEDPLPPVFSGTPKGSGAGYGVGFDLEEFLNQSFDMGVADGPQD
GQADSASLSASLLADWLEGHGMNPADIESLQREIQMDSPMLLADLPDLQDP

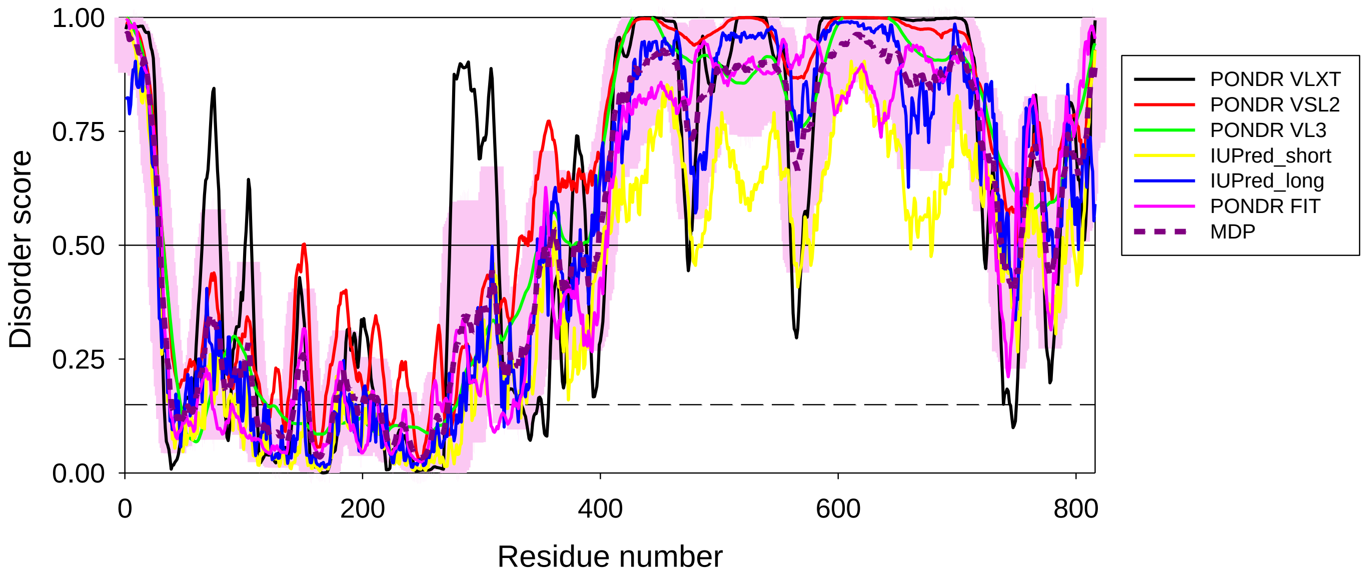

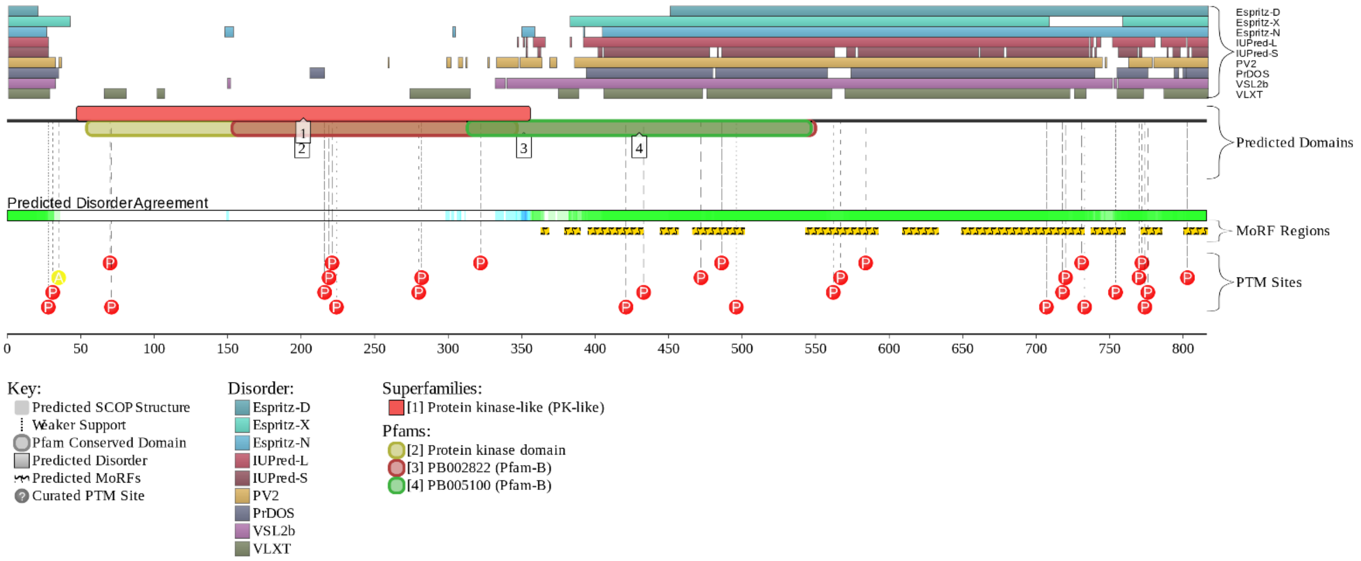

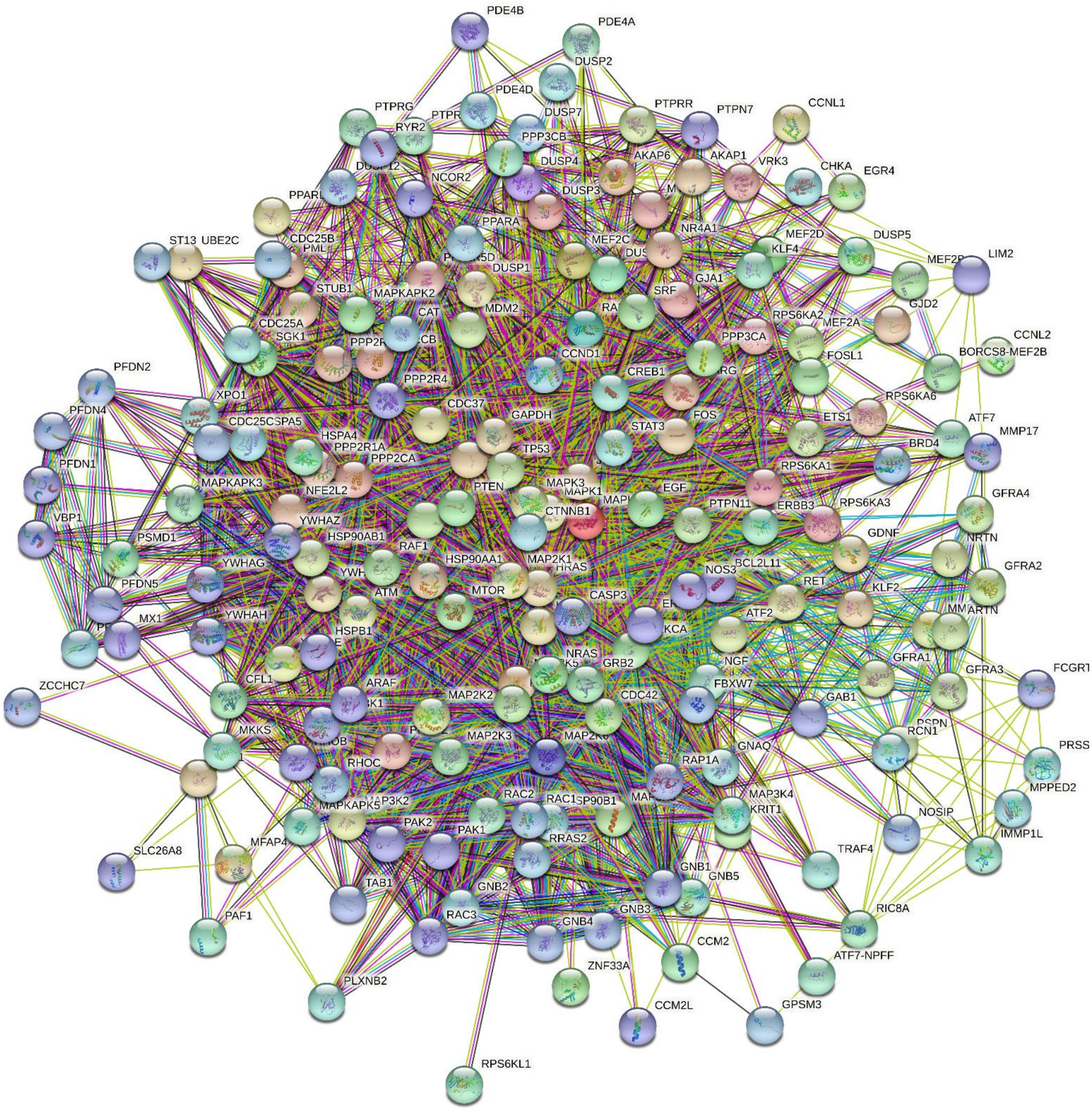
PDE4B
PDE4A
DUSP2
PDE4D
PTPRR
PTPN7
CCNL1
PTPRG
RYR2
PPP3CB
DUSP7
AKAP6
AKAP1
VRK3
CHKA
EGR4
NCOR2
DUSP4
DUSP3
NR4A1
MEF2D
DUSP5
PPARA
MEF2C
KLF4
LIM2
ST13
UBE2C
CDC25B
PML
DUSP1
GJA1
SRF
STUB1
MAPKAPK2
CAT
MDM2
RPS6KA2
MEF2A
GJD2
CCNL2
CDC25A
SGK1
PPP3CA
CCND1
BORCS8-MEF2B
FOSL1
PFDN2
PPP2R4
CREB1
XPO1
CDC37
RPS6KA6
PFDN4
CDC25C
HSPA5
GAPDH
FOS
STAT3
ETS1
ATF7
MMP17
HSPA4
PPP2R1A
TP53
BRD4
PFDN1
PPP2CA
RPS6KA1
MAPKAPK3
NFE2L2
PTEN
MAPK3
MAPK1
EGF
GFRA4
VBP1
YWHAZ
CTNNB1
PTPN11
ERBB3
RPS6KA3
PSMD1
HSP90AB1
RAF1
GDNF
NRTN
MAP2K1
HSP90AA1
HRAS
BCL2L11
NOS3
GFRA2
YWHAG
PFDN5
MTOR
ATM
RET
KLF2
CASP3
ATF2
ARTN
MX1
YWHAH
HSPB1
NRAS
NGF
GFRA1
GFRA3
GRB2
ZCCHC7
CFL1
ARAF
CDC42
FBXW7
MAP2K2
GAB1
PSPN
RCN1
MKKS
RHOB
MAP2K3
GNAQ
RHOC
RAP1A
MPPED2
MAPKAPK5
RAC2
RAC1
MAP3K4
KRIT1
NOSIP
PAK2
PAK1
RRAS2
SLC26A8
MFAP4
IMMP1L
TRAF4
TAB1
GNB1
GNB5
GNB2
RAC3
GNB3
RIC8A
PAF1
GNB4
CCM2
ZNF33A
ATF7-NPFF
PLXNB2
CCM2L
GPSM3
RPS6KL1

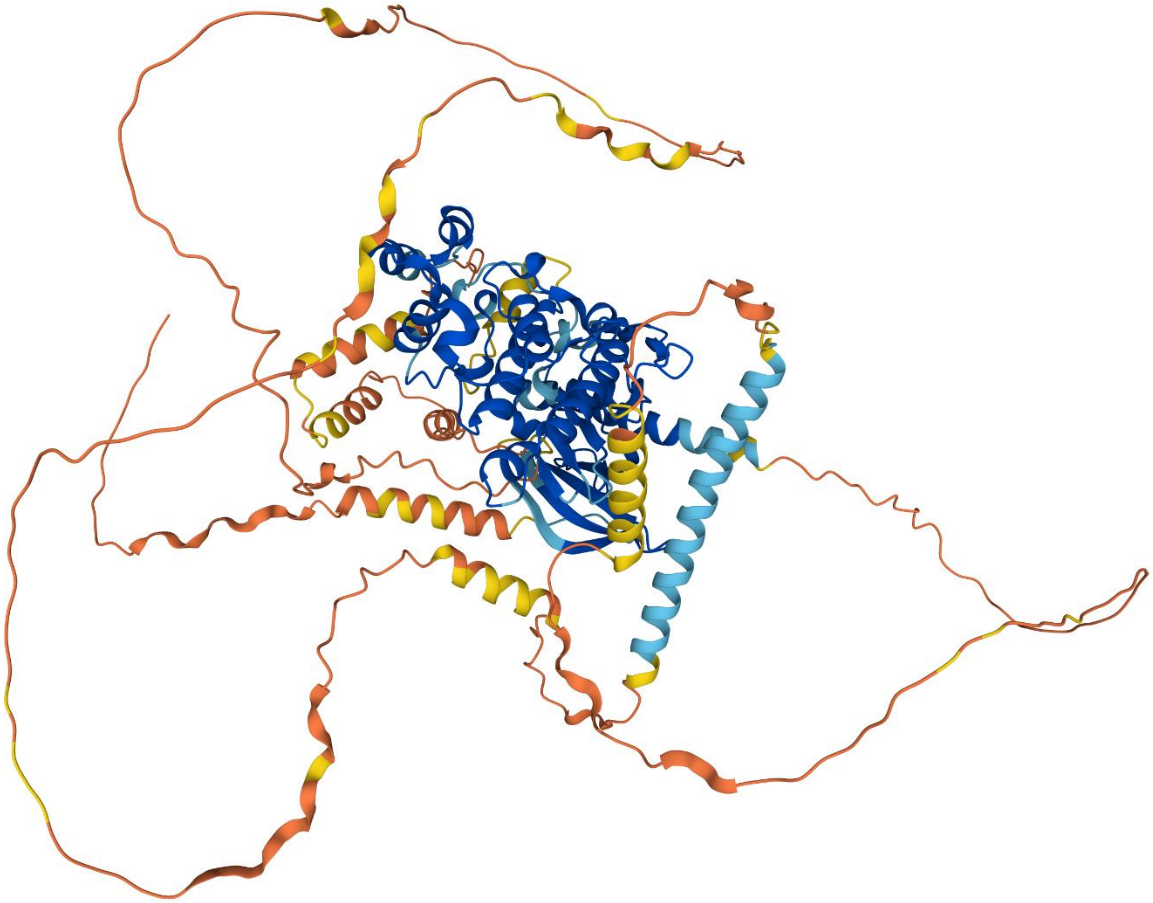

```
>sp|P45983|MK08_HUMAN Mitogen-activated protein kinase 8 OS=Homo sapiens OX=9606
GN=MAPK8 PE=1 SV=2
MSRSKRDNNFYSVEIGDSTFTVLKRYQNLKPIGSGAQGIVCAAYDAILERNVAIKKLSRPFQNQTHAKRAYRELVLMKCVNHKNI
IGLLNVFTPQKSLEEFQDVYIVMELMDANLCQVIQMELDHERMSYLLYQMLCGIKHLHSAGIIHRDLKPSNIVVKSDCTLKILDF
GLARTAGTSFMMTPYVVTRYYRAPEVILGMGYKENVDLWSVGCIMGEMVCHKILFPGRDYIDQWNKVIEQLGTPCPEFMKKLQPT
VRTYVENRPKYAGYSFEKLFPDVLFPADSEHNKLKASQARDLLSKMLVIDASKRISVDEALQHPYINVWYDPSEAEAPPPKIPDK
QLDEREHTIEEWKELIYKEVMDLEERTKNGVIRGQPSPLGAAVINGSQHPSSSSSVNDVSSMSTDPTLASDTDSSLEAAAGPLGC
CR
```

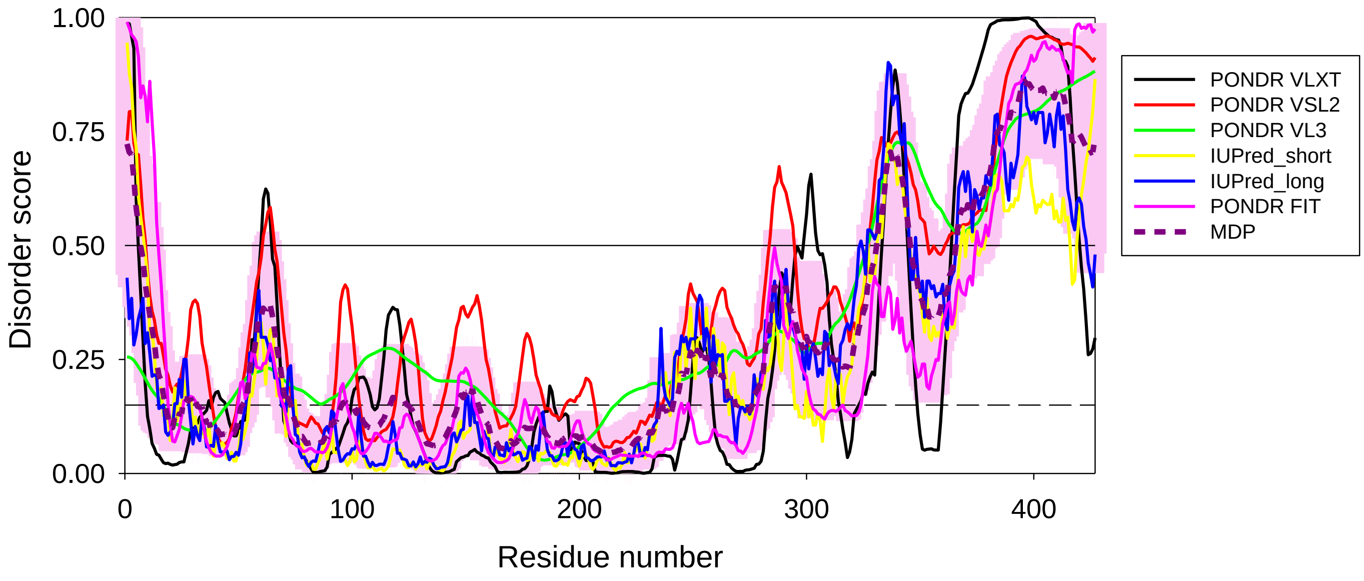

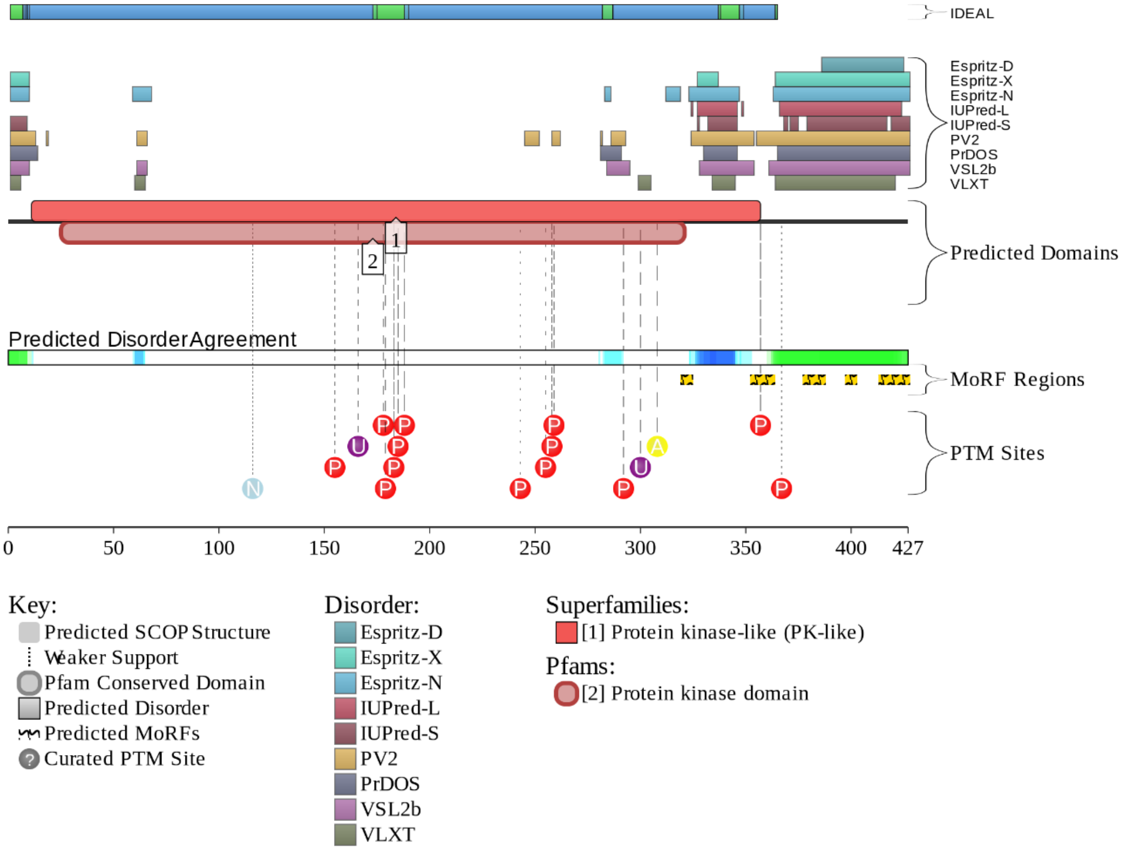

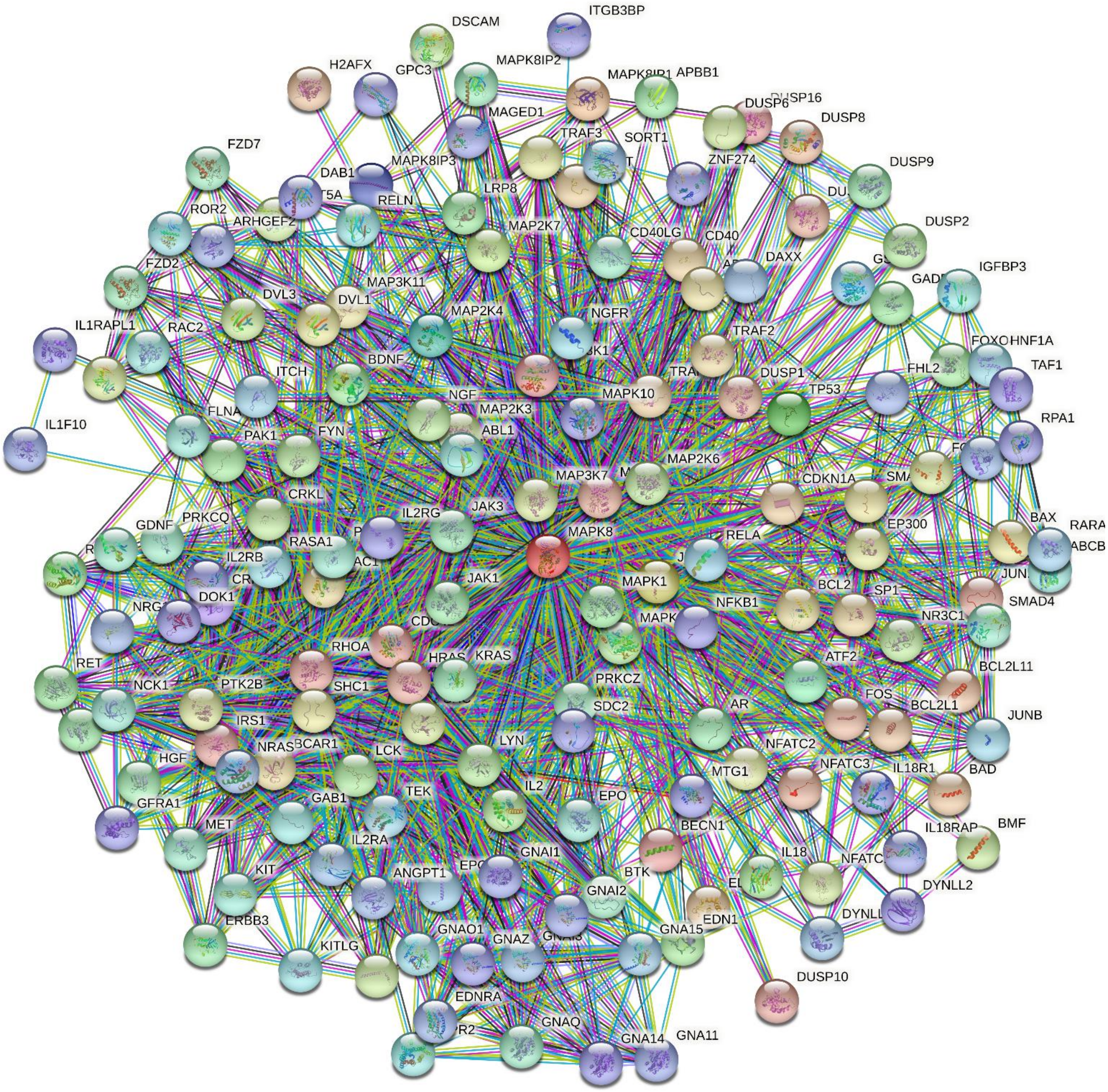
ITGB3BP
DSCAM
H2AFX
GPC3
MAPK8IP2
APBB1
DUSP6
DUSP8
MAGED1
TRAF3
SORT1
FZD7
DAB1
MAPK8IP3
ZNF274
DUSP9
RELN
LRP8
ROR2
ARHGEF2
MAP2K7
CD40LG
CD40
DUSP2
DAXX
IGFBP3
FZD2
DVL3
DVL1
MAP3K11
MAP2K4
NGFR
IL1RAPL1
RAC2
TRAF2
HNF1A
TAF1
ITCH
BDNF
FHL2
NGF
MAPK10
DUSP1
TP53
IL1F10
FLNA
MAP2K3
PAK1
FYN
ABL1
RPA1
MAP2K6
MAP3K7
CDKN1A
CRKL
JAK3
IL2RG
MAPK8
EP300
BAX
RARA
GDNF
PRKCQ
RELA
RASA1
IL2RB
DOK1
JAK1
MAPK1
NFKB1
BCL2
SP1
SMAD4
NR3C1
RHOA
HRAS
KRAS
ATF2
BCL2L11
RET
NCK1
PTK2B
SHC1
PRKCZ
SDC2
AR
FOS
BCL2L1
JUNB
IRS1
NRAS
BCAR1
LCK
LYN
NFATC2
NFATC3
HGF
MTG1
IL18R1
BAD
TEK
IL2
EPO
GAB1
GFRA1
MET
BECN1
IL18RAP
BMF
IL2RA
IL18
GNAI1
KIT
ANGPT1
BTK
GNAI2
DYNLL2
ERBB3
EDN1
GNAO1
GNAZ
GNA15
KITLG
DUSP10
EDNRA
GNAQ
GNA14
GNA11

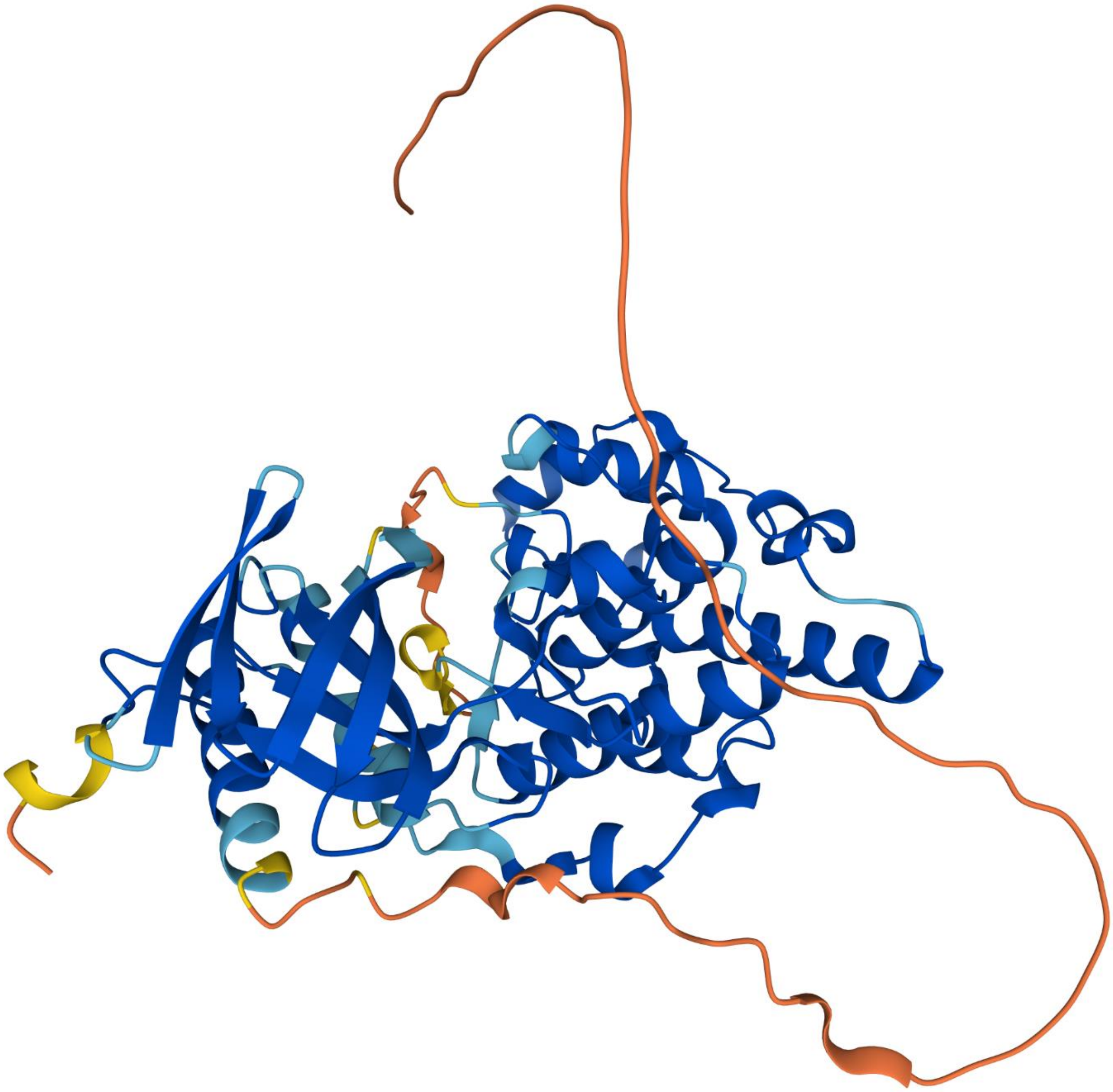

>sp|P45984|MK09_HUMAN Mitogen-activated protein kinase 9 OS=Homo sapiens OX=9606 GN=MAPK9 PE=1 SV=2
MSDSKCDSQFYSVQVADSTFTVLKRYQQLKPIGSGAQGIVCAAFDTVLGINVAVKKLSRPFQNQTHAKRAYRELVLLKCVNHKNI
ISLLNVFTPQKTLEEFQDVYLVMELMDANLCQVIHMELDHERMSYLLYQMLCGIKHLHSAGIIHRDLKPSNIVVKSDCTLKILDF
GLARTACTNFMMTPYVVTRYYRAPEVILGMGYKENVDIWSVGCIMGELVKGCVIFQGTDHIDQWNKVIEQLGTPSAEFMKKLQPT
VRNYVENRPKYPGIKFEELFPDWIFPSESERDKIKTSQARDLLSKMLVIDPDKRISVDEALRHPYITVWYDPAEAEAPPPQIYDA
QLEEREHAIEEWKELIYKEVMDWEERSKNGVVKDQPSDAAVSSNATPSQSSSINDISSMSTEQTLASDTDSSLDASTGPLEGCR

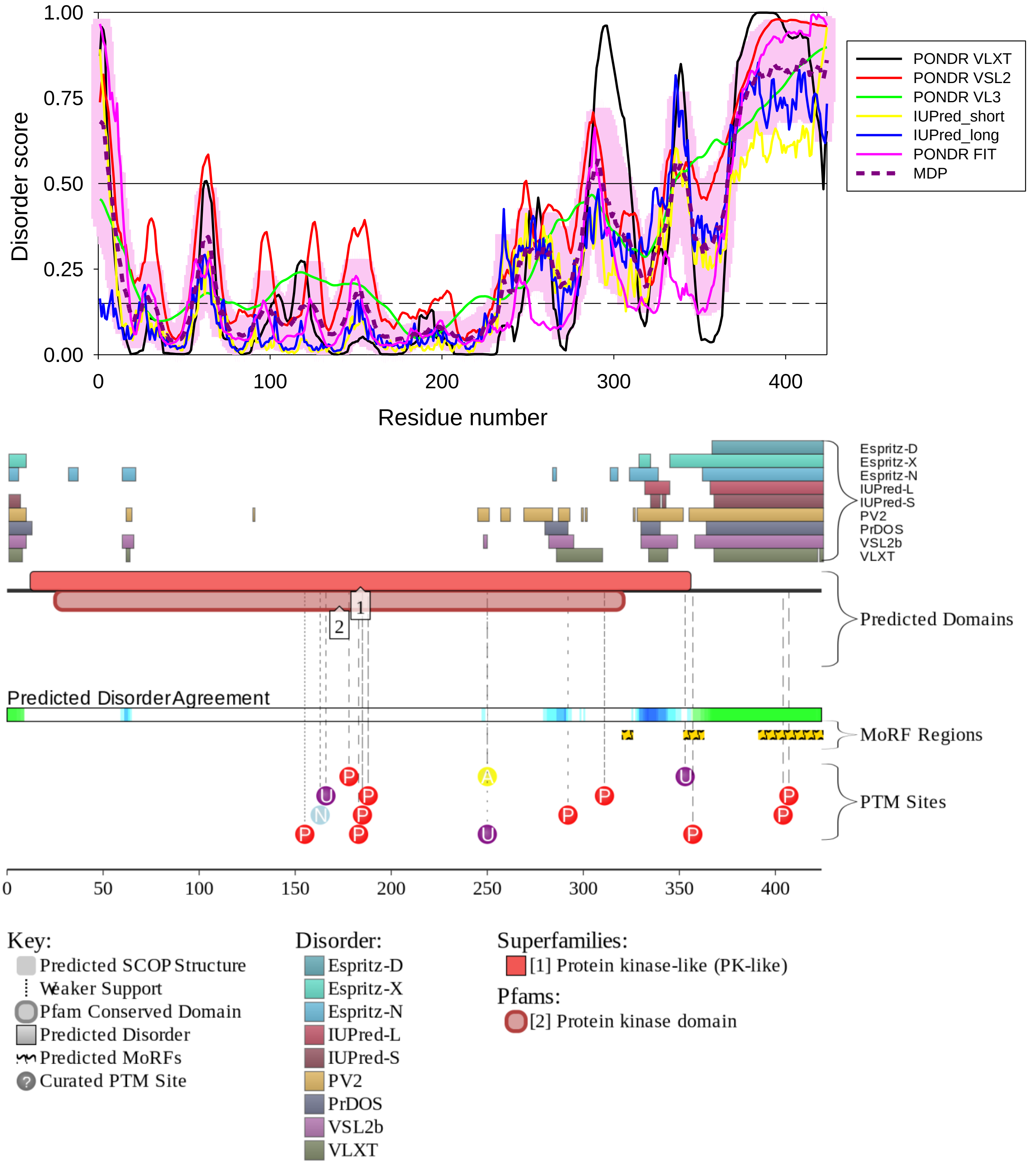

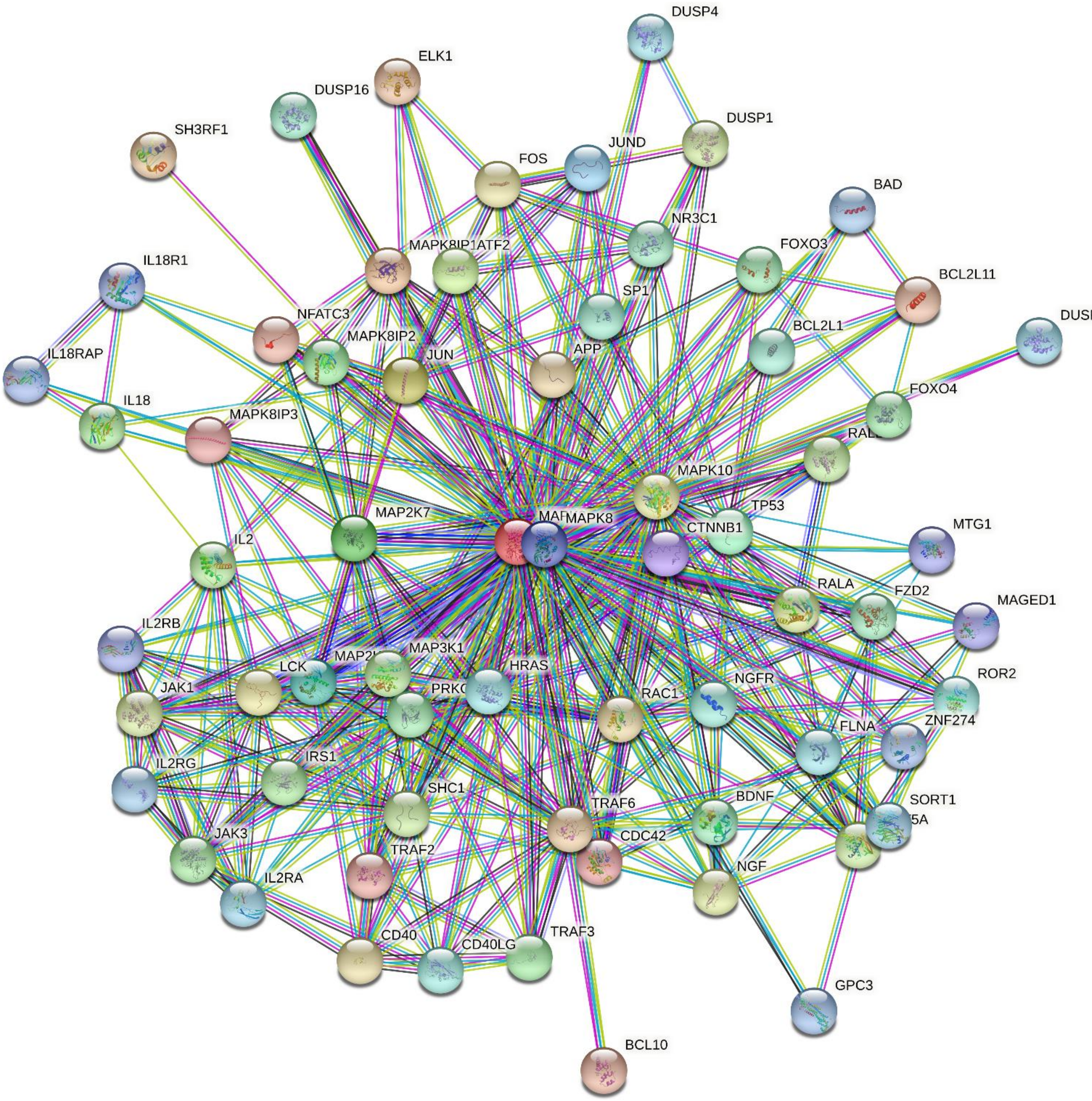

DUSP4
ELK1
DUSP16
DUSP1
SH3RF1
JUND
FOS
BAD
NR3C1
FOXO3
IL18R1
MAPK8IP1
ATF2
BCL2L11
SP1
NFATC3
BCL2L1
DUSP1
IL18RAP
MAPK8IP2
JUN
APP
FOXO4
IL18
MAPK8IP3
MAPK10
TP53
MAP2K7
MAPK8
CTNNB1
MTG1
IL2
RALA
FZD2
MAGED1
IL2RB
MAP3K1
LCK
HRAS
ROR2
JAK1
RAC1
NGFR
ZNF274
FLNA
IL2RG
IRS1
SHC1
SORT1
TRAF6
BDNF
JAK3
CDC42
TRAF2
NGF
IL2RA
CD40
CD40LG
TRAF3
GPC3
BCL10

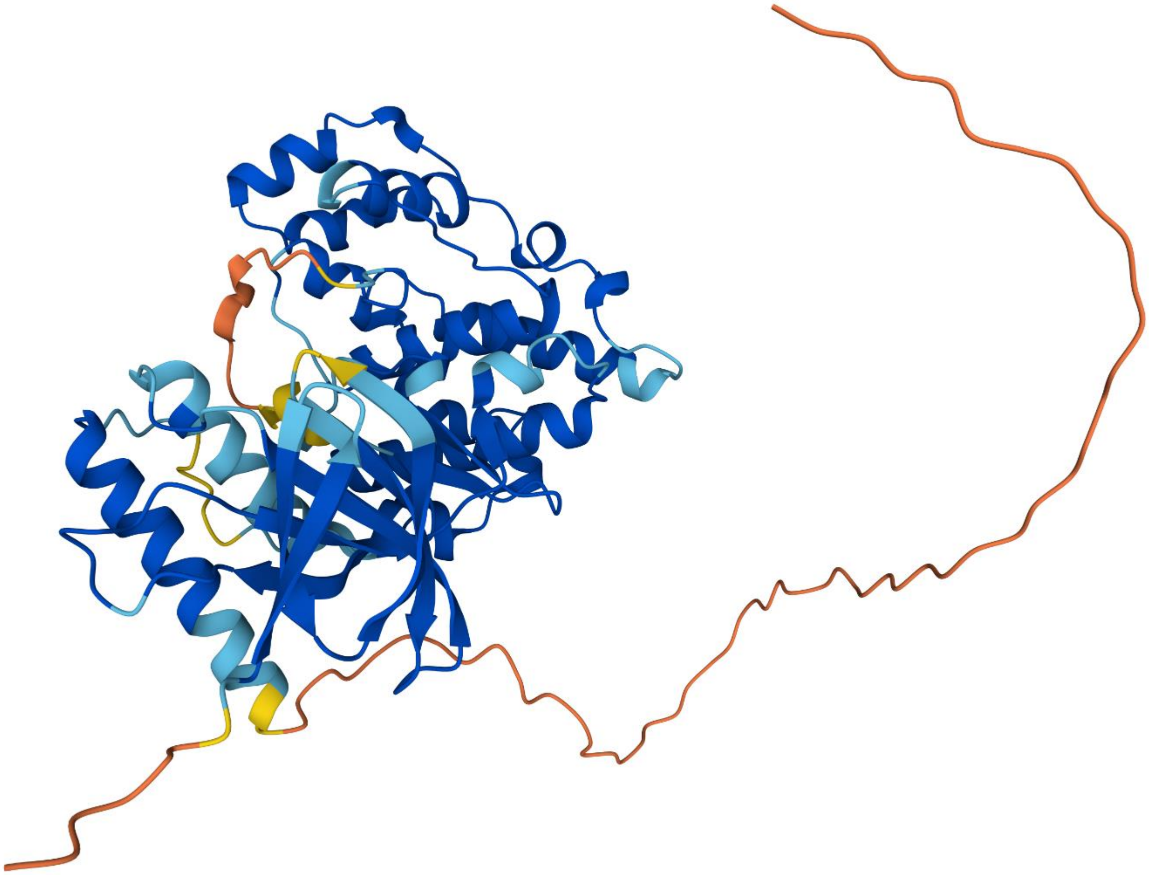

>sp|P53779|MK10_HUMAN Mitogen-activated protein kinase 10 OS=Homo sapiens OX=9606 GN=MAPK10 PE=1 SV=2
MSLHFLYYCSEPTLDVKIAFCQGFDKQVDVSYIAKHYNMSKSKVDNQFYSVEVGDSTFTVLKRYQNLKPIGSGAQGIVCAAYDAV
LDRNVAIKKLSRPFQNQTHAKRAYRELVLMKCVNHKNIISLLNVFTPQKTLEEFQDVYLVMELMDANLCQVIQMELDHERMSYLL
YQMLCGIKHLHSAGIIHRDLKPSNIVVKSDCTLKILDFGLARTAGTSFMMTPYVVTRYYRAPEVILGMGYKENVDIWSVGCIMGE
MVRHKILFPGRDYIDQWNKVIEQLGTPCPEFMKKLQPTVRNYVENRPKYAGLTFPKLFPDSLFPADSEHNKLKASQARDLLSKML
VIDPAKRISVDDALQHPYINVWYDPAEVEAPPPQIYDKQLDEREHTIEEWKELIYKEVMNSEEKTKNGVVKGQPSPSGAAVNSSE
SLPPSSSVNDISSMSTDQTLASDTDSSLEASAGPLGCCR

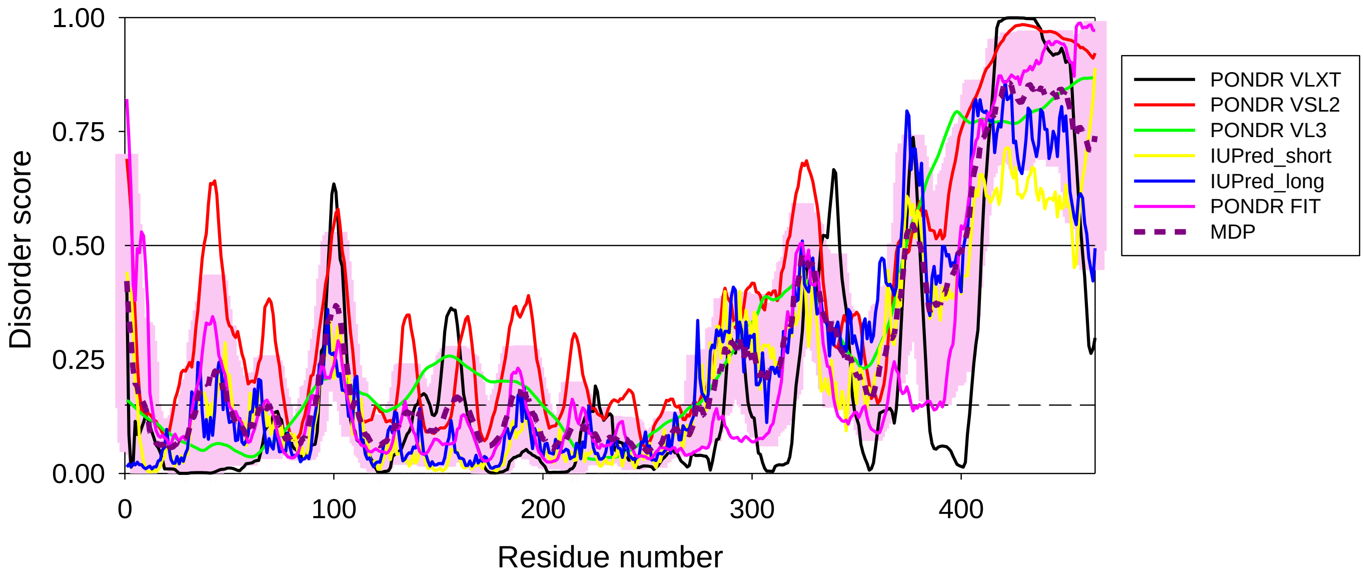

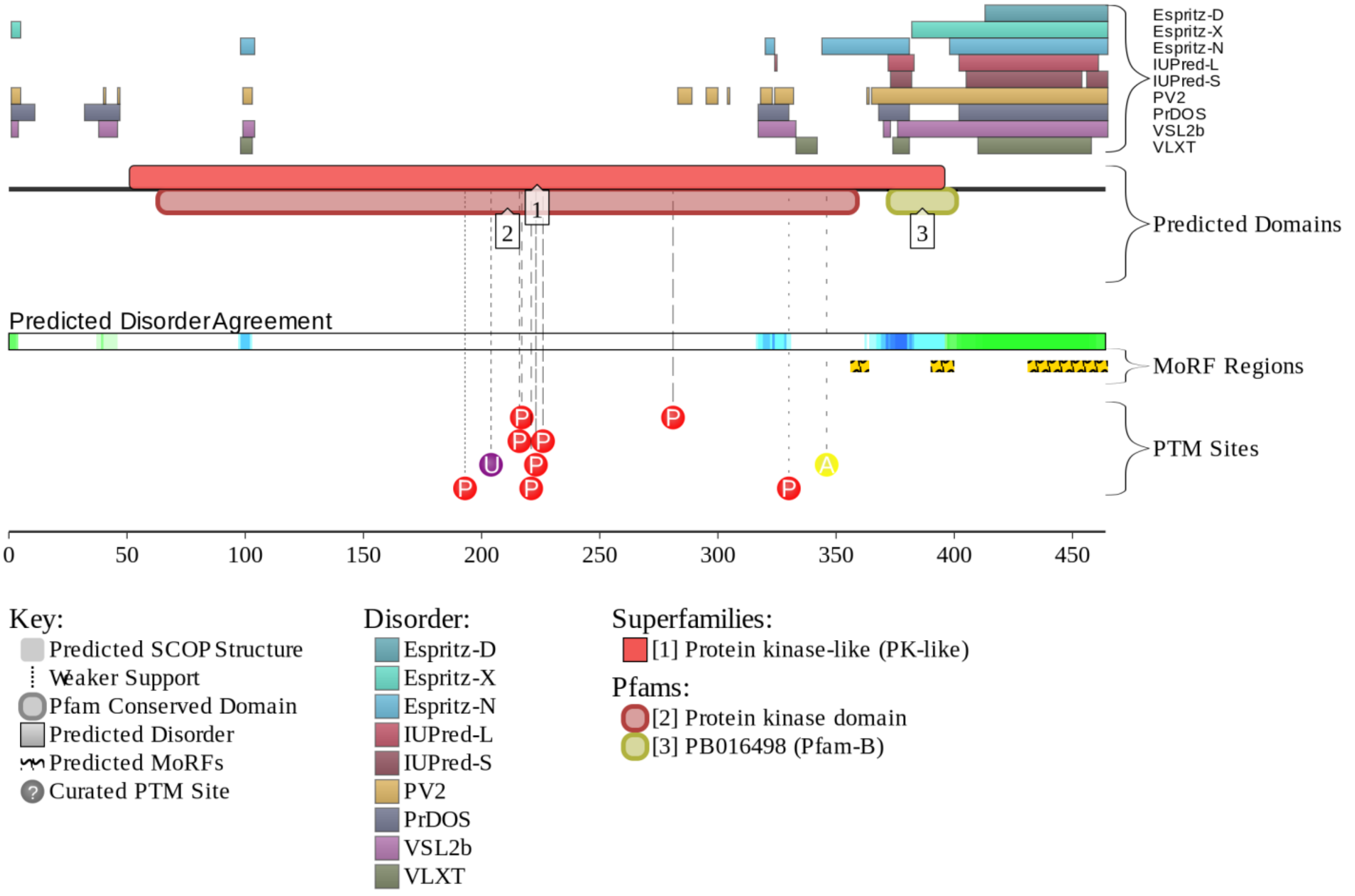

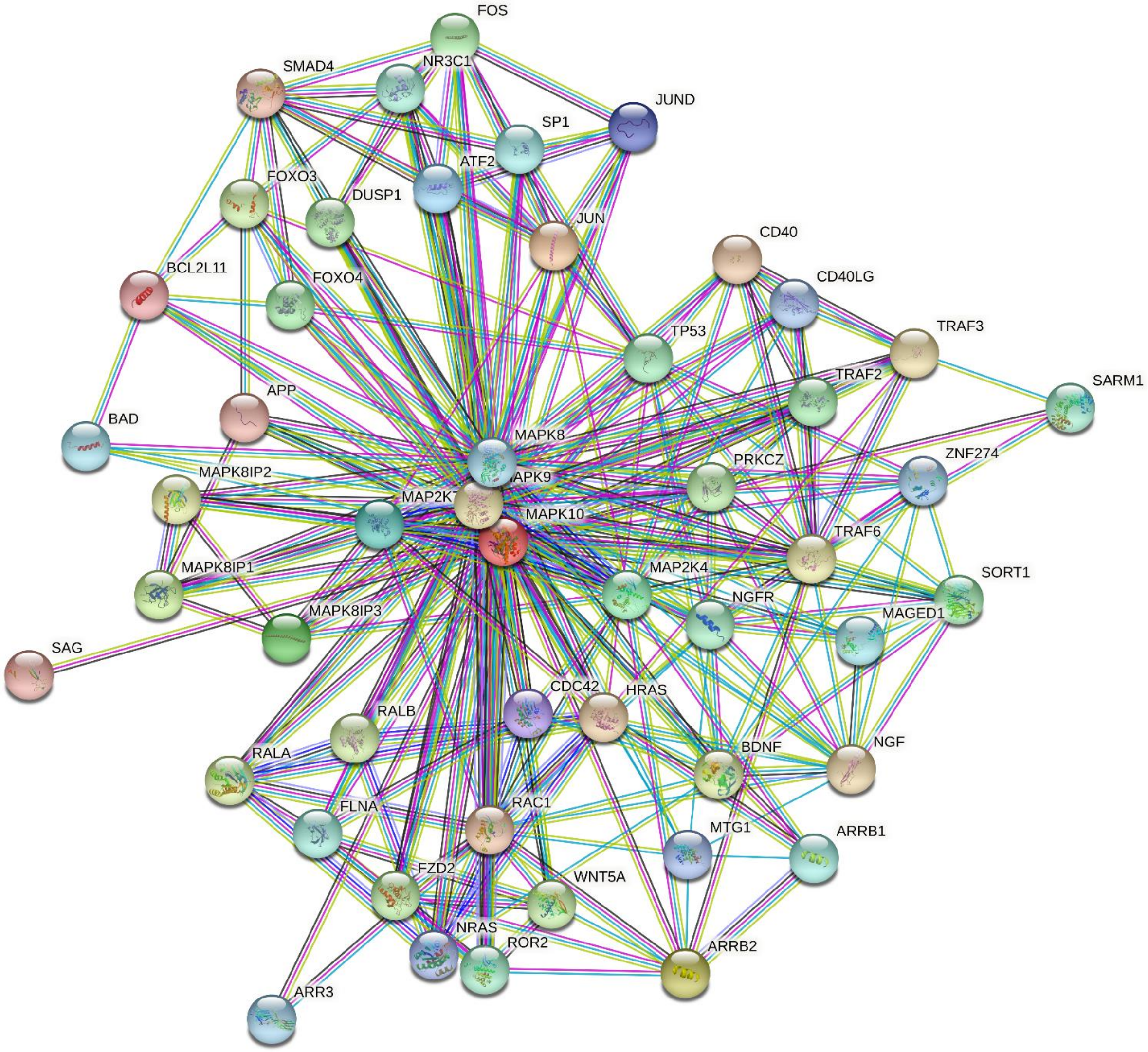
FOS
SMAD4
NR3C1
JUND
SP1
ATF2
FOXO3
DUSP1
JUN
CD40
BCL2L11
FOXO4
CD40LG
TRAF3
TP53
TRAF2
SARM1
APP
BAD
MAPK8
PRKCZ
ZNF274
MAPK8IP2
APK9
MAP2K7
MAPK10
TRAF6
SORT1
MAPK8IP1
MAP2K4
NGFR
MAPK8IP3
MAGED1
SAG
CDC42
HRAS
RALB
NGF
BDNF
RALA
FLNA
RAC1
MTG1
ARRB1
FZD2
WNT5A
NRAS
ROR2
ARRB2
ARR3

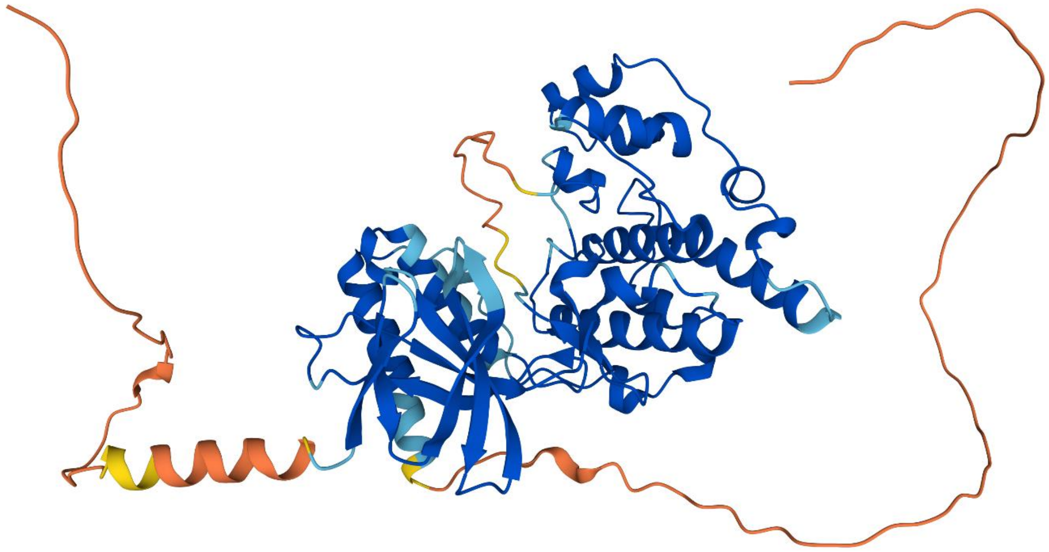

>sp|Q15759|MK11_HUMAN Mitogen-activated protein kinase 11 OS=Homo sapiens OX=9606 GN=MAPK11 PE=1 SV=2
MSGPRAGFYRQELNKTVWEVPQRLQGLRPVGSGAYGSVCSAYDARLRQKVAVKKLSRPFQSLIHARRTYRELRLLKHLKHENVIG
LLDVFTPATSIEDFSEVYLVTTLMGADLNNIVKCQALSDEHVQFLVYQLLRGLKYIHSAGIIHRDLKPSNVAVNEDCELRILDFG
LARQADEEMTGYVATRWYRAPEIMLNWMHYNQTVDIWSVGCIMAELLQGKALFPGSDYIDQLKRIMEVVGTPSPEVLAKISSEHA
RTYIQSLPPMPQKDLSSIFRGANPLAIDLLGRMLVLDSDQRVSAAEALAHAYFSQYHDPEDEPEAEPYDESVEAKERTLEEWKEL
TYQEVLSFKPPEPPKPPGSLEIEQ

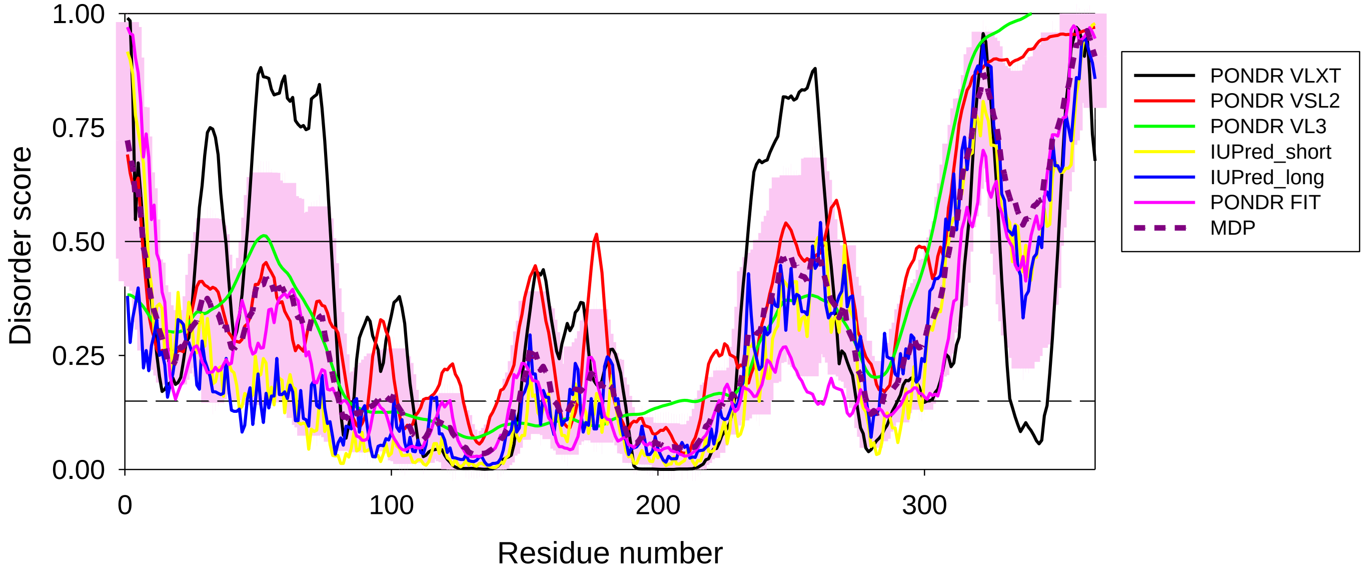

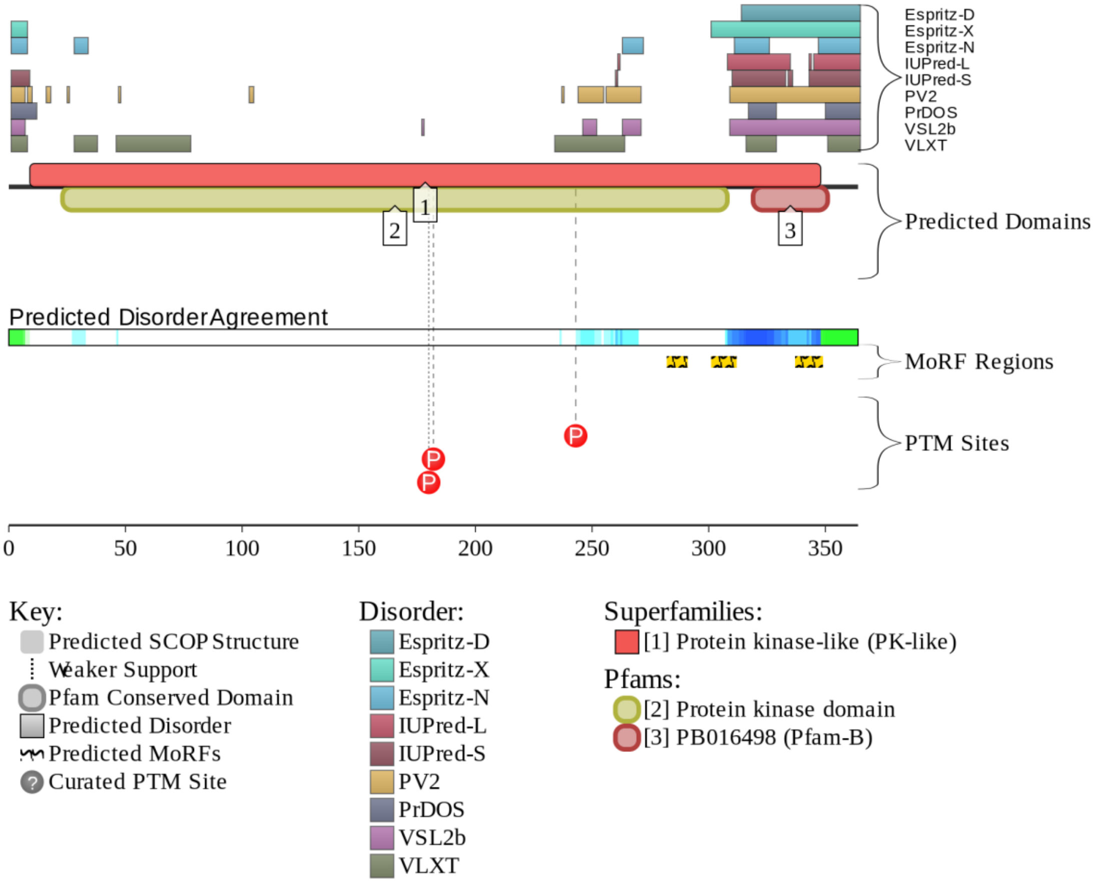

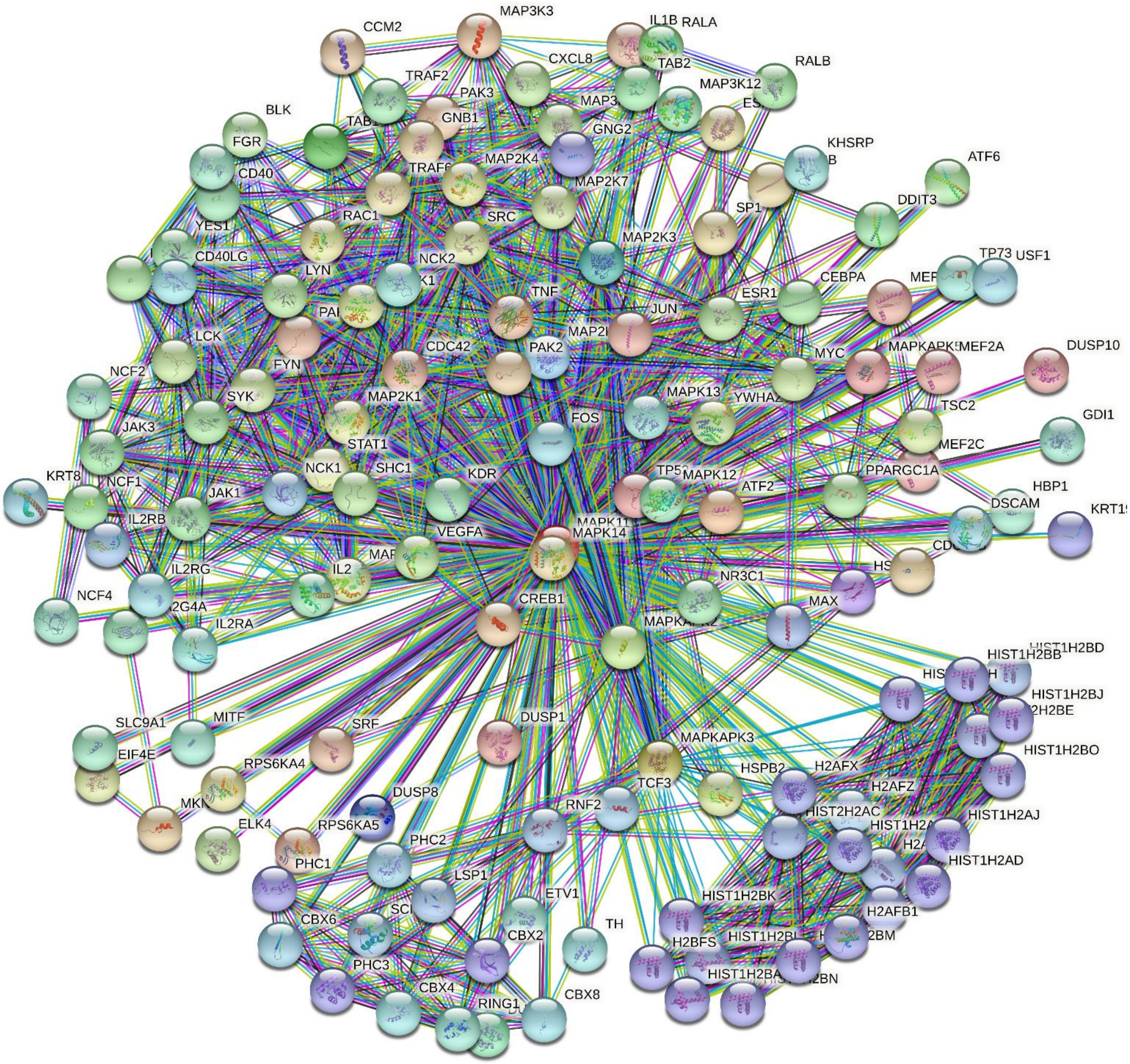
CCM2
MAP3K3
IL1B
RALA
CXCL8
TAB2
RALB
TRAF2
PAK3
MAP3K12
BLK
GNB1
GNG2
FGR
KHSRP
ATF6
CD40
TRAF6
MAP2K4
MAP2K7
SP1
DDIT3
RAC1
SRC
YES1
MAP2K3
CD40LG
NCK2
TP73
USF1
LYN
TNF
CEBPA
ESR1
JUN
LCK
PAK2
MYC
MEF2A
DUSP10
NCF2
CDC42
FYN
SYK
MAP2K1
MAPK13
TSC2
FOS
JAK3
STAT1
GDI1
MEF2C
NCK1
SHC1
KDR
PPARGC1A
KRT8
NCF1
JAK1
MAPK12
ATF2
HBP1
DSCAM
IL2RB
VEGFA
MAPK14
IL2
NR3C1
IL2RG
NCF4
CREB1
MAX
IL2RA
HIST1H2BD
HIST1H2BB
HIST1H2BJ
HIST1H2BO
SLC9A1
MITF
SRF
DUSP1
MAPKAPK3
EIF4E
RPS6KA4
HSPB2
H2AFX
TCF3
H2AFZ
DUSP8
HIST2H2AC
HIST1H2AJ
ELK4
RNF2
RPS6KA5
PHC2
PHC1
HIST1H2AD
LSP1
ETV1
HIST1H2BK
H2AFB1
CBX6
TH
CBX2
H2BFS
PHC3
CBX4
RING1
CBX8

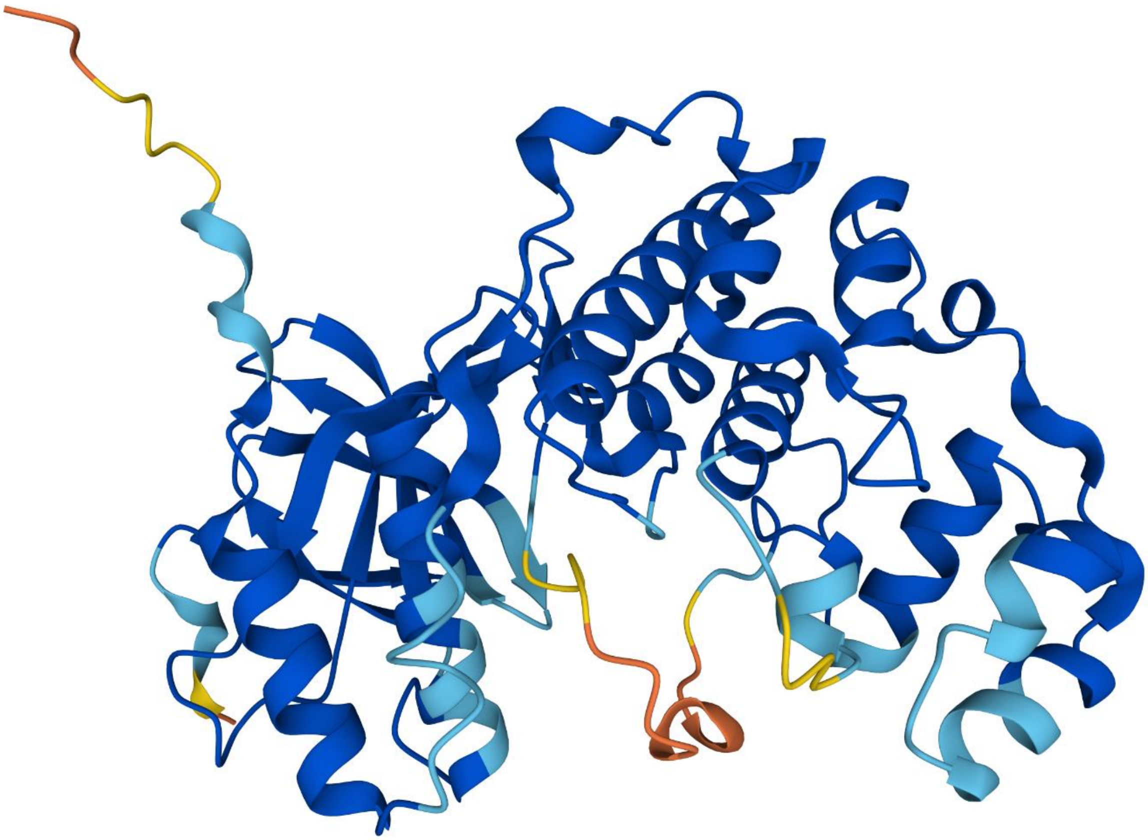

>sp|P53778|MK12_HUMAN Mitogen-activated protein kinase 12 OS=Homo sapiens OX=9606 GN=MAPK12 PE=1 SV=3
MSSPPPARSGFYRQEVTKTAWEVRAVYRDLQPVGSGAYGAVCSAVDGRTGAKVAIKKLYRPFQSELFAKRAYRELRLLKHMRHEN
VIGLLDVFTPDETLDDFTDFYLVMPFMGTDLGKLMKHEKLGEDRIQFLVYQMLKGLRYIHAAGIIHRDLKPGNLAVNEDCELKIL
DFGLARQADSEMTGYVVTRWYRAPEVILNWMRYTQTVDIWSVGCIMAEMITGKTLFKGSDHLDQLKEIMKVTGTPPAEFVQRLQS
DEAKNYMKGLPELEKKDFASILTNASPLAVNLLEKMLVLDAEQRVTAGEALAHPYFESLHDTEDEPQVQKYDDSFDDVDRTLDEW
KRVTYKEVLSFKPPRQLGARVSKETPL

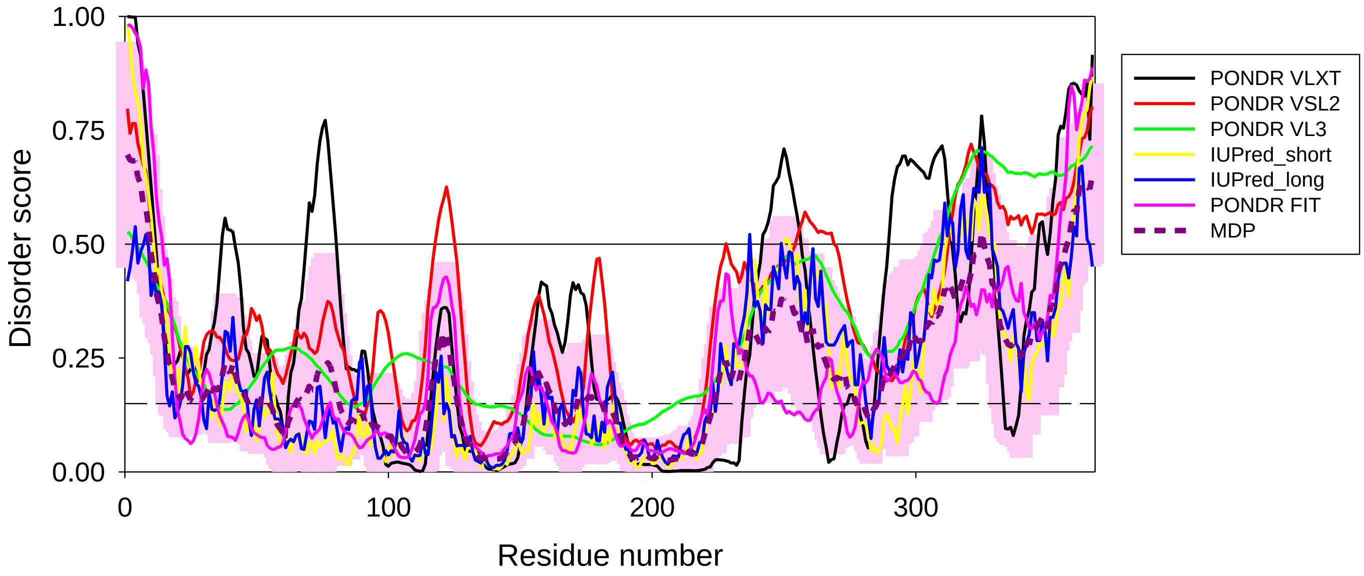

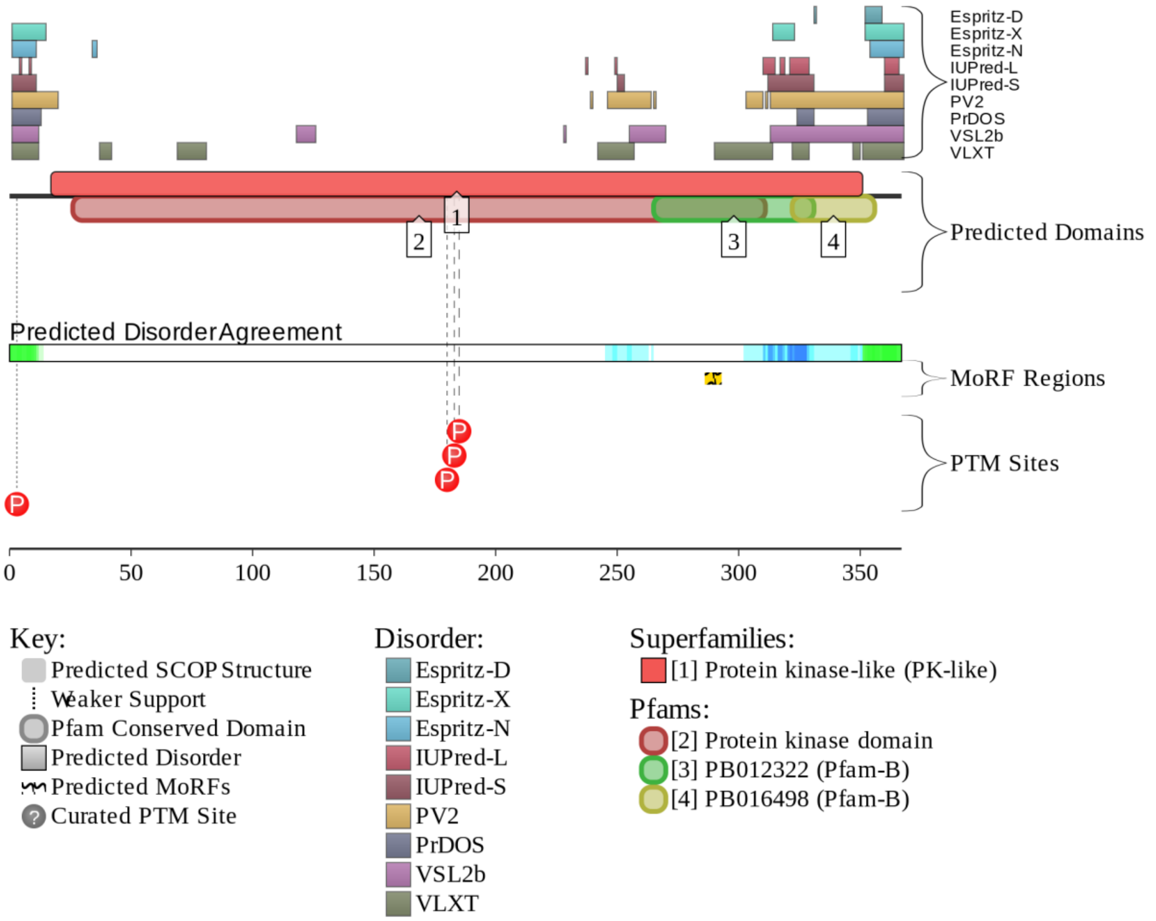

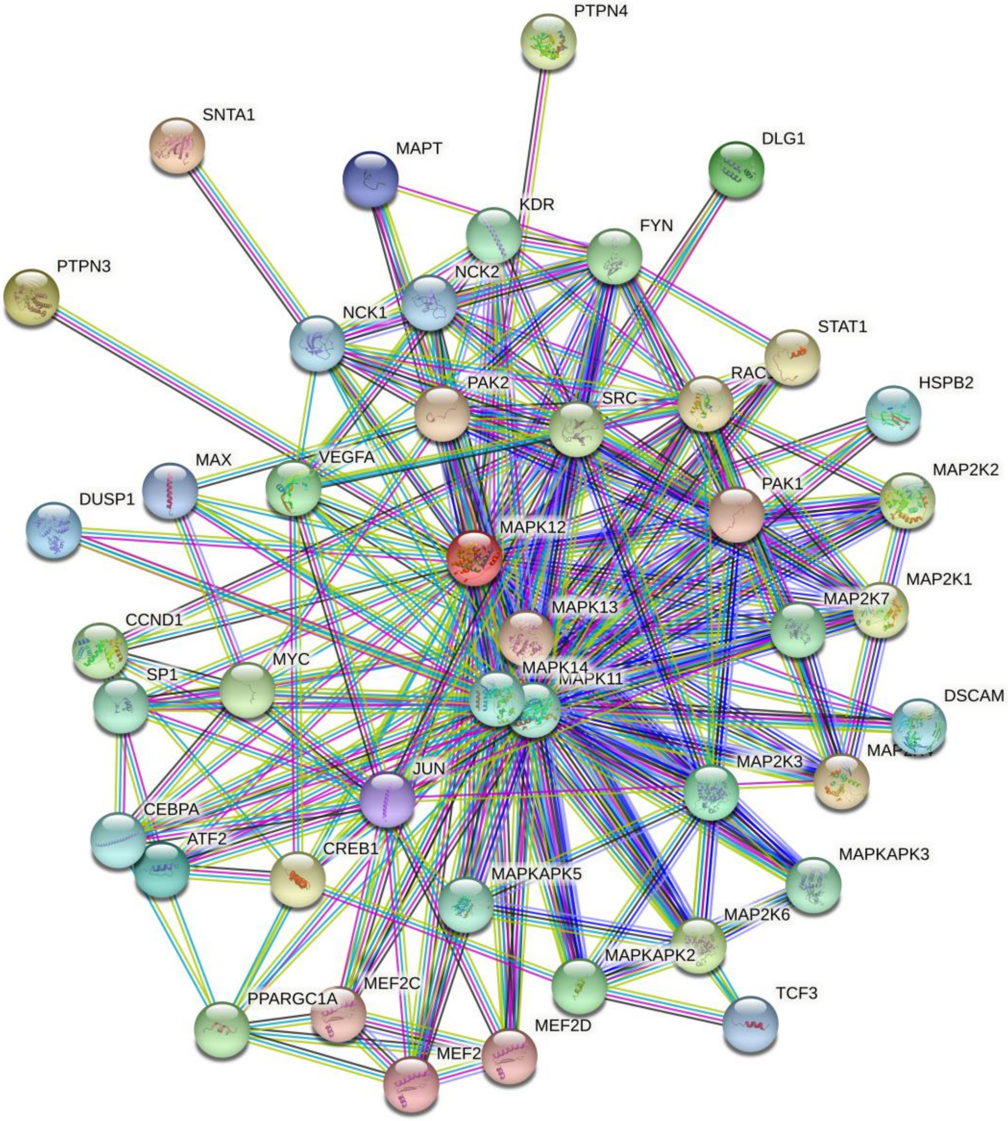
PTPN4
SNTA1
MAPT
DLG1
KDR
FYN
PTPN3
NCK2
NCK1
STAT1
HSPB2
PAK2
SRC
MAX
VEGFA
MAP2K2
DUSP1
PAK1
MAPK12
MAP2K1
MAP2K7
MAPK13
CCND1
MYC
MAPK14
MAPK11
SP1
DSCAM
MAP2K3
JUN
CEBPA
ATF2
CREB1
MAPKAPK3
MAPKAPK5
MAP2K6
MAPKAPK2
TCF3
MEF2C
PPARGC1A
MEF2D

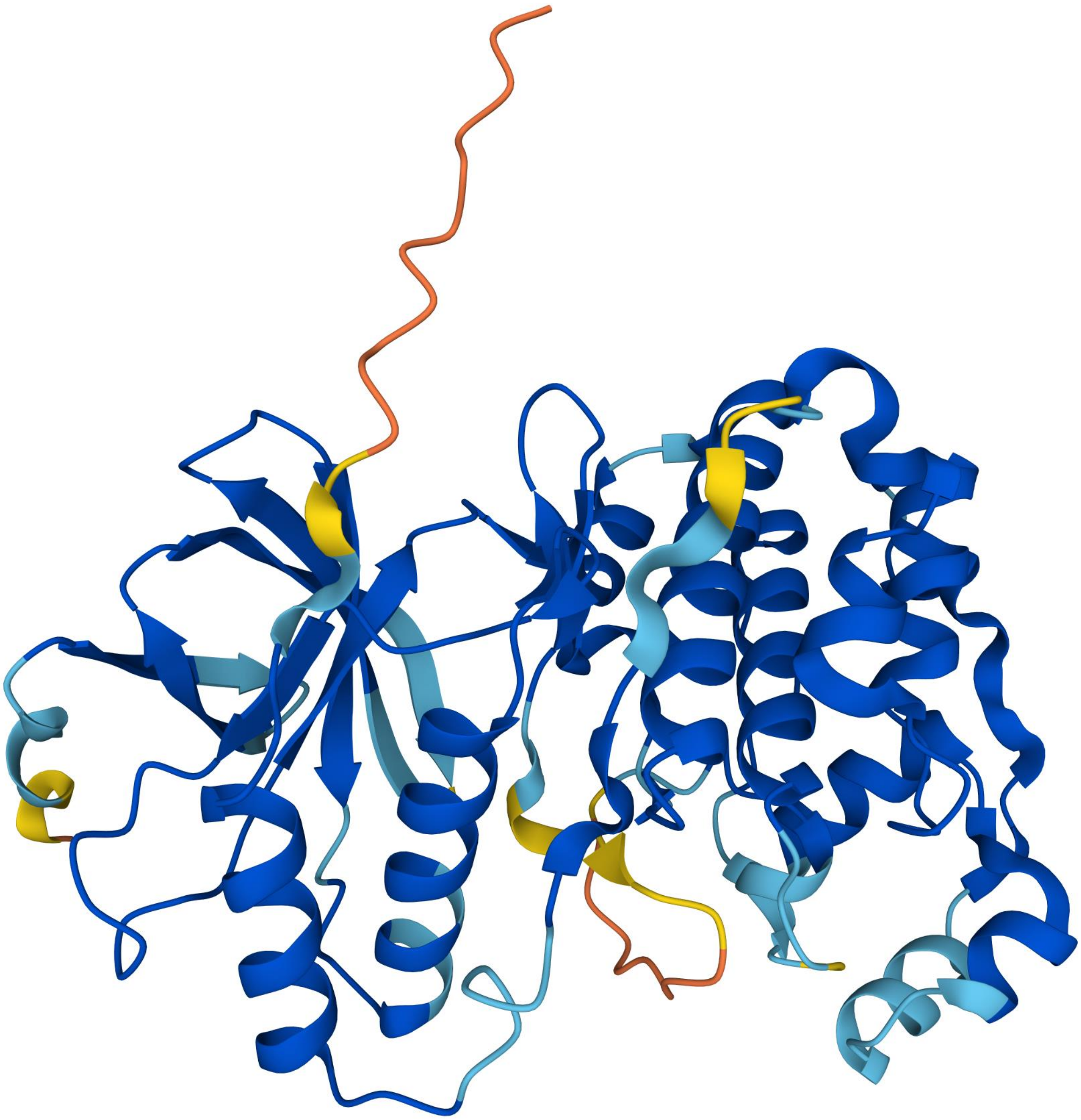

>sp|O15264|MK13_HUMAN Mitogen-activated protein kinase 13 OS=Homo sapiens OX=9606 GN=MAPK13 PE=1 SV=1

MSLIRKKGFYKQDVNKTAWELPKTYVSPTHVGSGAYGSVCSAIDKRSGEKVAIKKLSRPFQSEIFAKRAYRELLLLKHMQHENVI
GLLDVFTPASSLRNFYDFYLVMPFMQTDLQKIMGMEFSEEKIQYLVYQMLKGLKYIHSAGVVHRDLKPGNLAVNEDCELKILDFG
LARHADAEMTGYVVTRWYRAPEVILSWMHYNQTVDIWSVGCIMAEMLTGKTLFKGKDYLDQLTQILKVTGVPGTEFVQKLNDKAA
KSYIQSLPQTPRKDFTQLFPRASPQAADLLEKMLELDVDKRLTAAQALTHPFFEPFRDPEEETEAQQPFDDSLEHEKLTVDEWKQ
HIYKEIVNFSPIARKDSRRRSGMKL

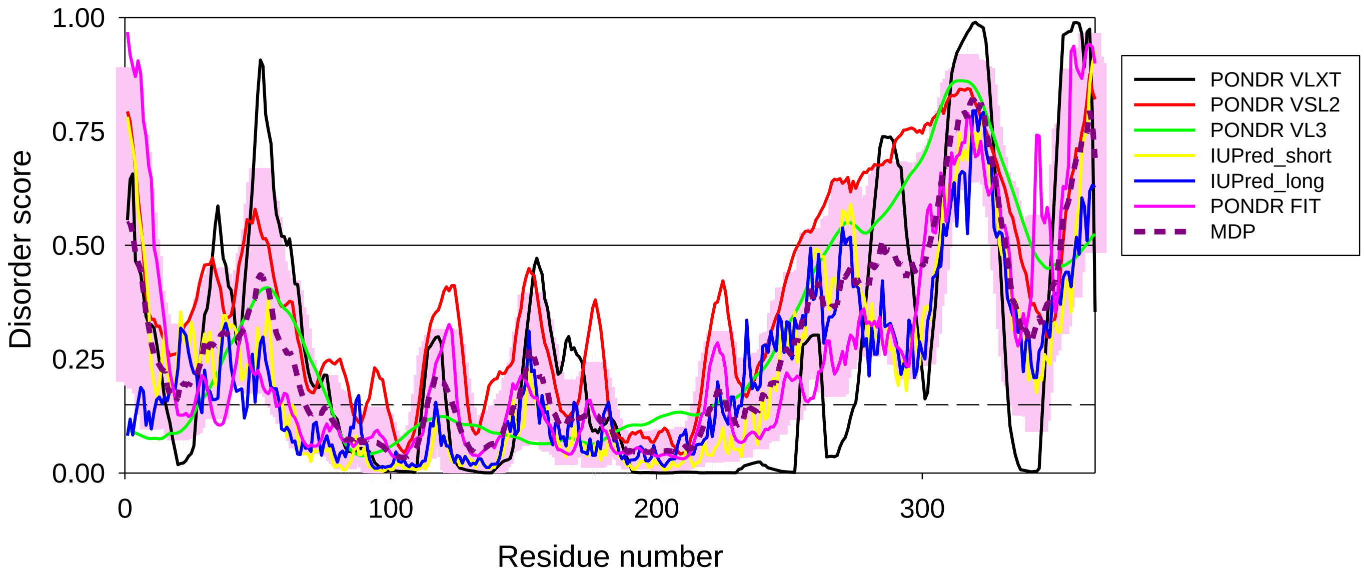

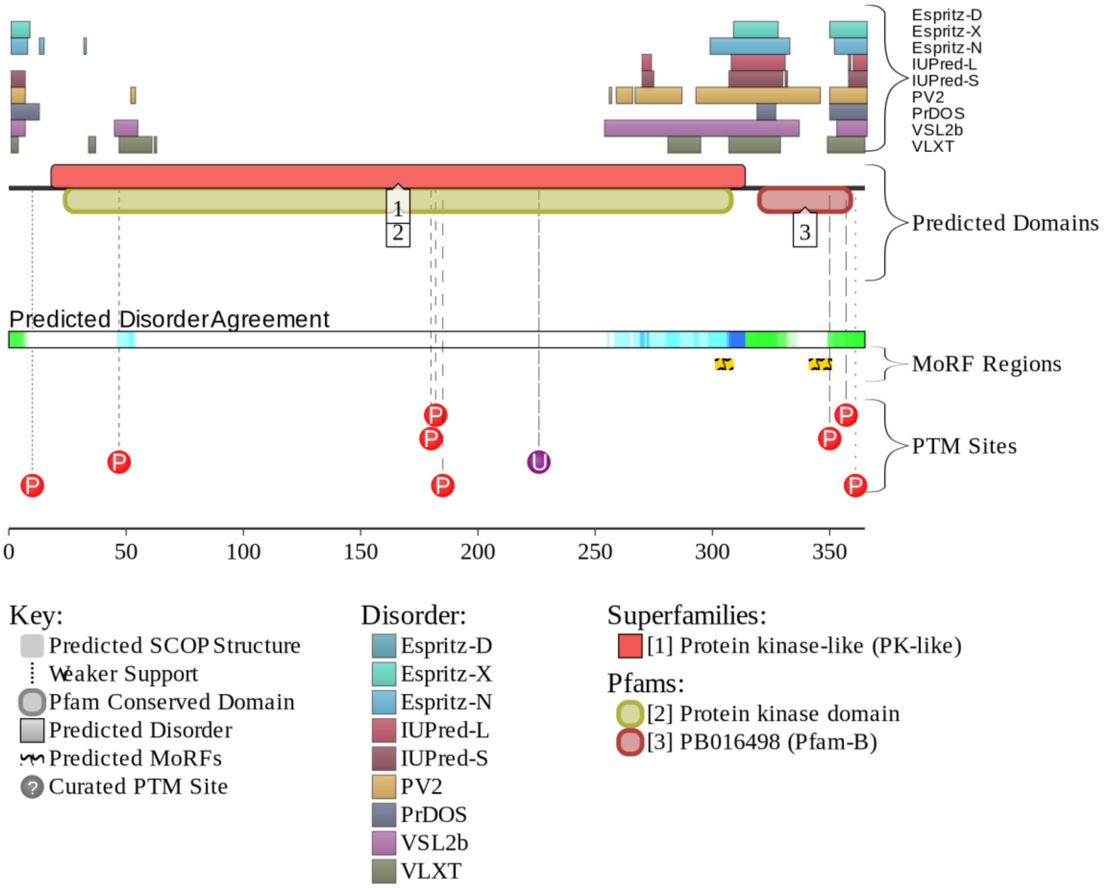

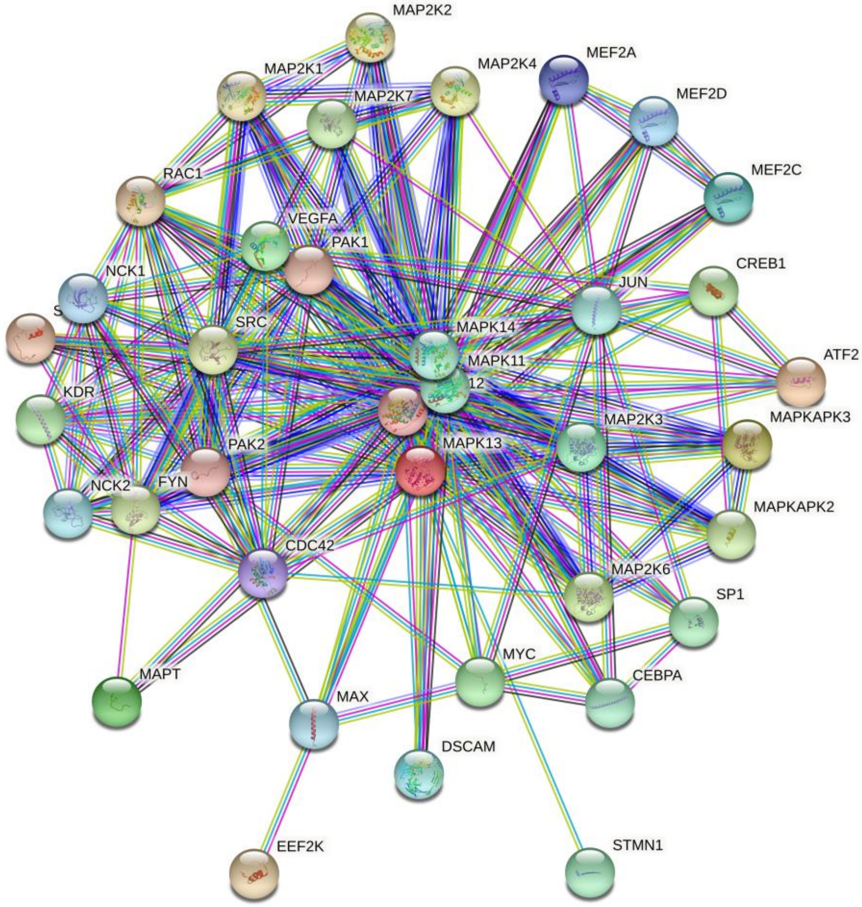
MAP2K2
MAP2K1
MAP2K4
MEF2A
MAP2K7
MEF2D
MEF2C
RAC1
VEGFA
PAK1
NCK1
CREB1
JUN
SRC
MAPK14
MAPK11
ATF2
KDR
MAP2K3
MAPKAPK3
PAK2
MAPK13
NCK2
FYN
MAPKAPK2
CDC42
MAP2K6
SP1
MYC
MAPT
CEBPA
MAX
DSCAM
EEF2K
STMN1

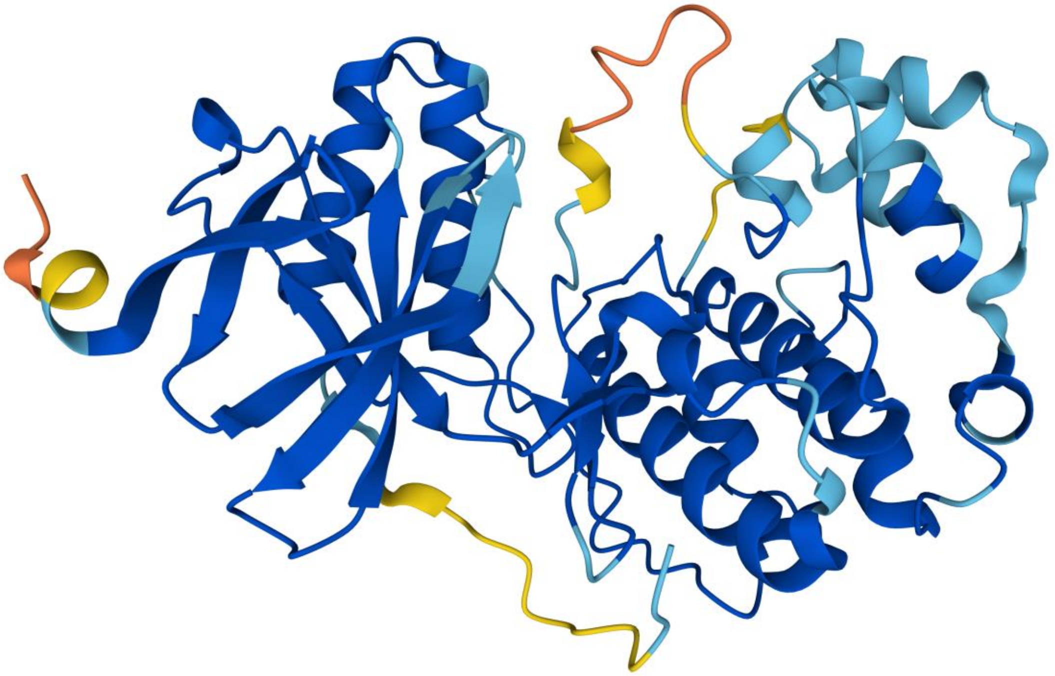

>sp|Q16539|MK14_HUMAN Mitogen-activated protein kinase 14 OS=Homo sapiens OX=9606
GN=MAPK14 PE=1 SV=3
MSQERPTFYRQELNKTIWEVPERYQNLSPVGSGAYGSVCAAFDTKTGLRVAVKKLSRPFQSIIHAKRTYRELRLLKHMKHENVIG
LLDVFTPARSLEEFNDVYLVTHLMGADLNNIVKCQKLTDDHVQFLIYQILRGLKYIHSADIIHRDLKPSNLAVNEDCELKILDFG
LARHTDDEMTGYVATRWYRAPEIMLNWMHYNQTVDIWSVGCIMAELLTGRTLFPGTDHIDQLKLILRLVGTPGAELLKKISSESA
RNYIQSLTQMPKMNFANVFIGANPLAVDLLEKMLVLDSDKRITAAQALAHAYFAQYHDPDDEPVADPYDQSFESRDLLIDEWKSL
TYDEVISFVPPPLDQEEMES

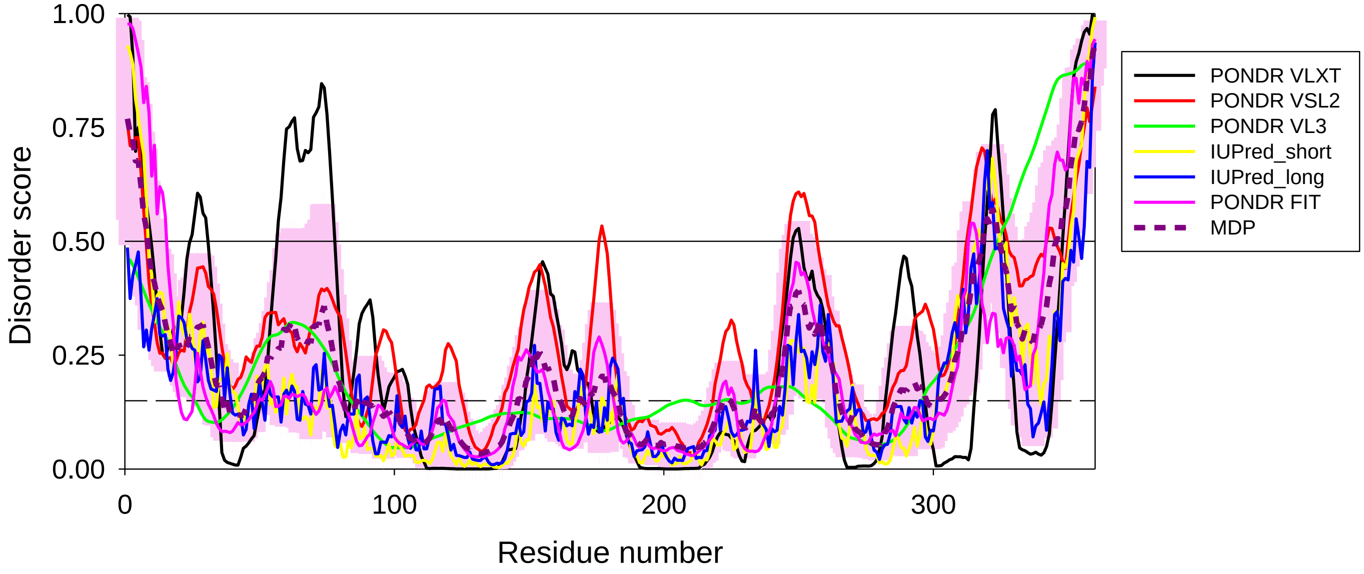

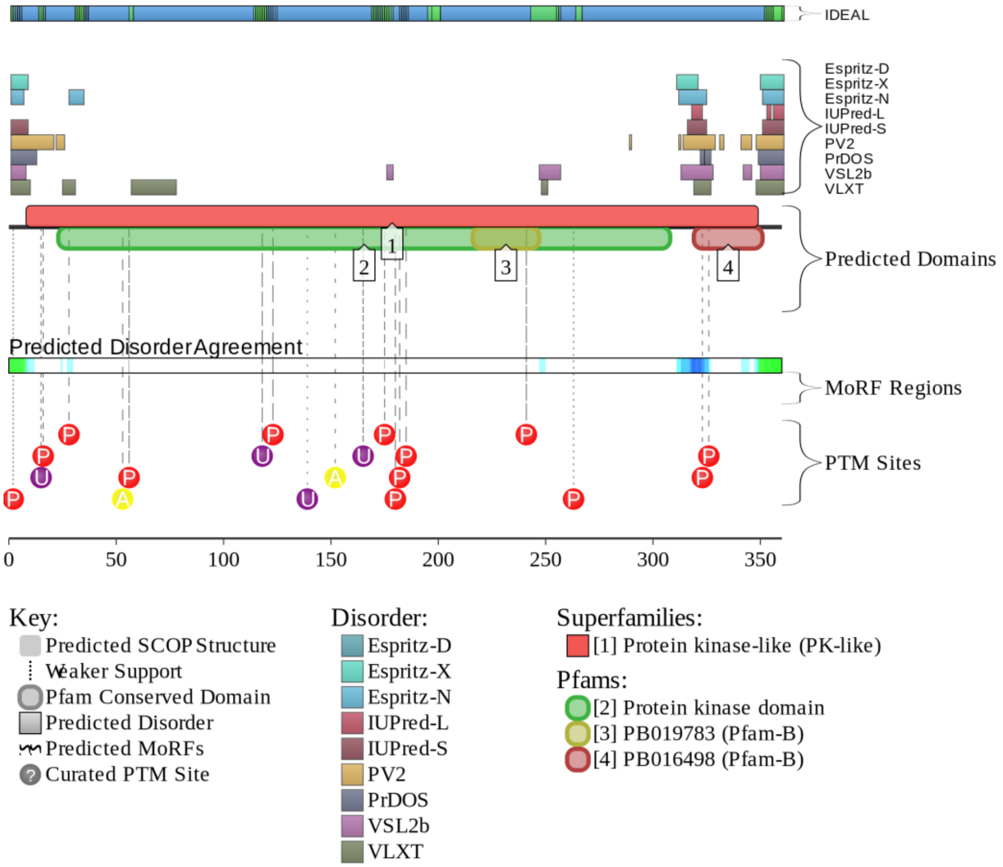

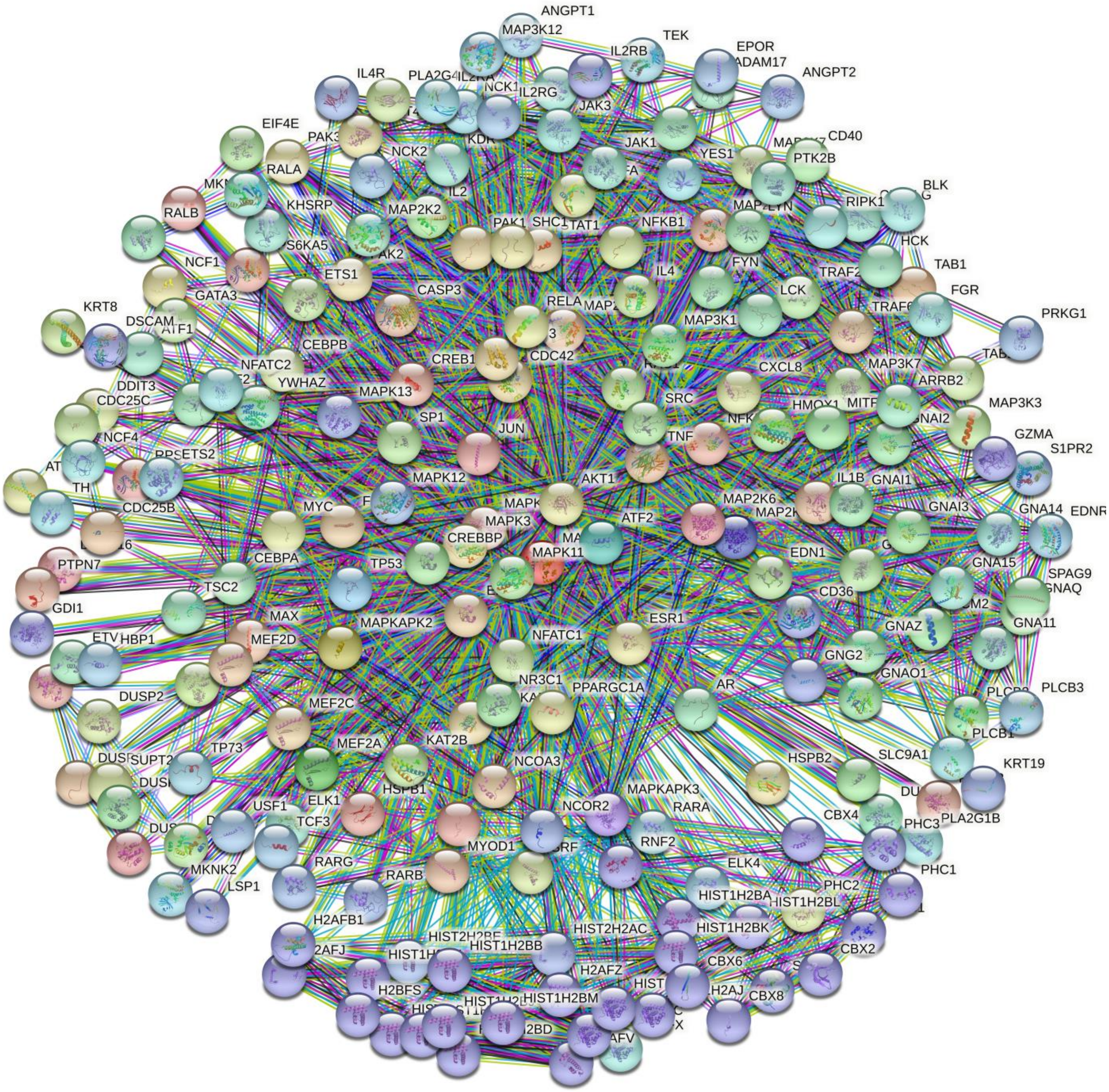
ANGPT1
MAP3K12
TEK
EPOR
ADAM17
ANGPT2
IL2RB
IL4R
NCK1
IL2RG
JAK3
EIF4E
PAK3
KDR
JAK1
CD40
YES1
PTK2B
NCK2
RALA
BLK
RIPK1
RALB
KHSRP
MAP2K2
IL2
PAK1
SHC1
NFKB1
HCK
NCF1
S6KA5
TAB1
ETS1
GATA3
IL4
FYN
TRAF2
CASP3
LCK
FGR
KRT8
DSCAM
ATF1
RELA
MAP3K1
PRKG1
CEBPB
NFATC2
CDC42
CREB1
CXCL8
MAP3K7
DDIT3
YWHAZ
MAPK13
ARRB2
CDC25C
SRC
HMOX1
MAP3K3
SP1
JUN
GZMA
NCF4
S1PR2
ETS2
MAPK12
AKT1
IL1B
GNAI1
TH
CDC25B
MYC
MAP2K6
GNAI3
GNA14
MAPK3
ATF2
CREBBP
PTPN7
MAPK11
EDN1
CEBPA
TP53
GNA15
SPAG9
GNAQ
TSC2
CD36
GDI1
MAX
ESR1
GNAZ
GNA11
MAPKAPK2
HBP1
MEF2D
NFATC1
GNG2
GNAO1
DUSP2
NR3C1
PPARGC1A
AR
PLCB3
MEF2C
PLCB1
TP73
KAT2B
MEF2A
SLC9A1
KRT19
HSPB2
NCOA3
HSPB1
ELK1
MAPKAPK3
NCOR2
RARA
USF1
TCF3
CBX4
PHC3
PLA2G1B
RNF2
MYOD1
ELK4
PHC1
RARG
RARB
MKNK2
LSP1
PHC2
HIST1H2BA
HIST1H2BL
H2AFB1
HIST2H2AC
HIST1H2BK
HIST1H2BB
CBX2
H2AFZ
CBX6
H2BFS
HIST1H2BM
H2AJ
CBX8

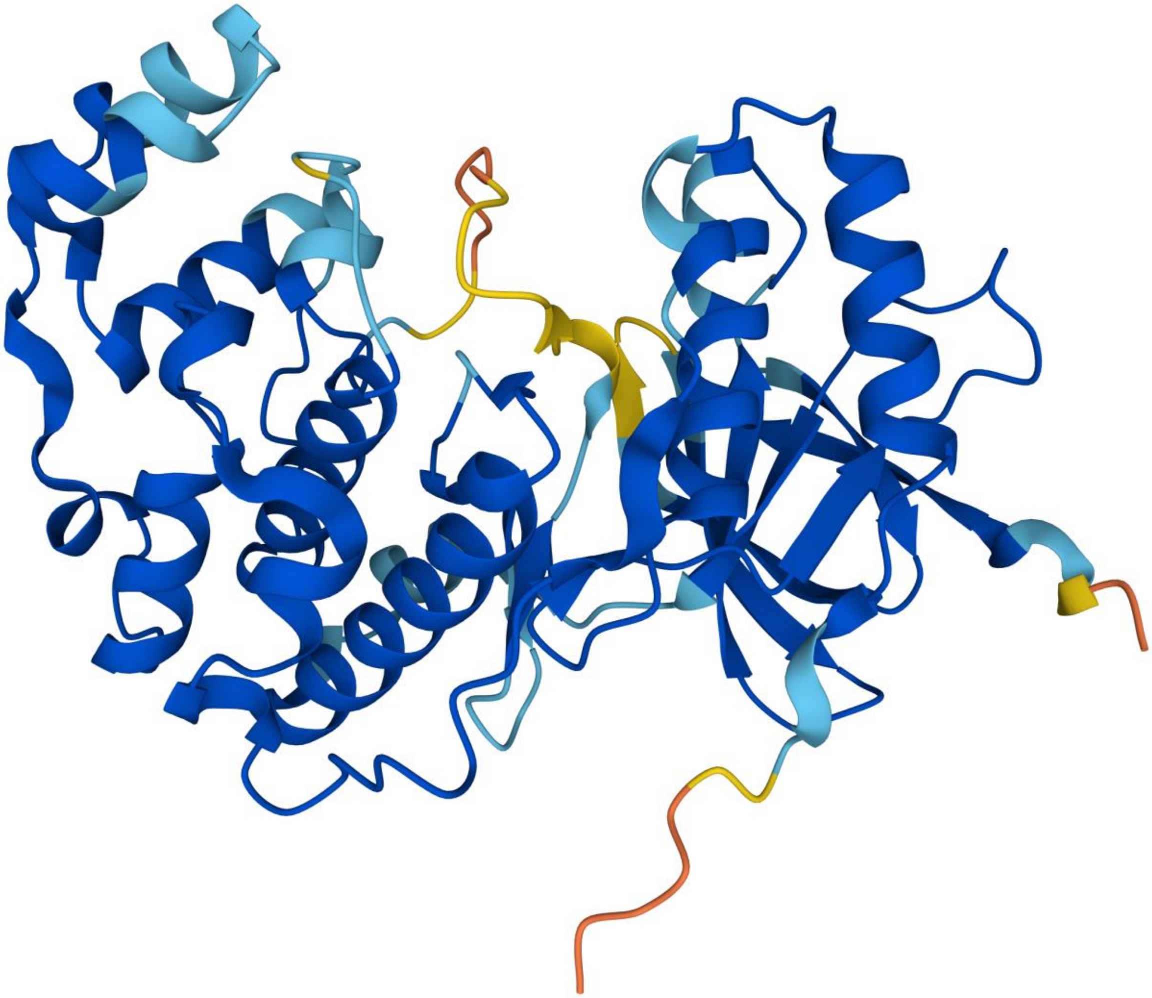

>sp|Q8TD08|MK15_HUMAN Mitogen-activated protein kinase 15 OS=Homo sapiens OX=9606 GN=MAPK15 PE=1 SV=1
MCTVVDPRIVRRYLLRRQLGQGAYGIVWKAVDRRTGEVVAIKKIFDAFRDKTDAQRTFREITLLQEFGDHPNIISLLDVIRAEND
RDIYLVFEFMDTDLNAVIRKGGLLQDVHVRSIFYQLLRATRFLHSGHVVHRDQKPSNVLLDANCTVKLCDFGLARSLGDLPEGPE
DQAVTEYVATRWYRAPEVLLSSHRYTLGVDMWSLGCILGEMLRGRPLFPGTSTLHQLELILETIPPPSEEDLLALGSGCRASVLH
QLGSRPRQTLDALLPPDTSPEALDLLRRLLVFAPDKRLSATQALQHPYVQRFHCPSDEWAREADVRPRAHEGVQLSVPEYRSRVY
QMILECGGSSGTSREKGPEGVSPSQAHLHKPRADPQLPSRTPVQGPRPRPQSSPGHDPAEHESPRAAKNVPRQNSAPLLQTALLG
NGERPPGAKEAPPLTLSLVKPSGRGAAPSLTSQAAAQVANQALIRGDWNRGGGVRVASVQQVPPRLPPEARPGRRMFSTSALQGA
QGGARALLGGYSQAYGTVCHSALGHLPLLEGHHV

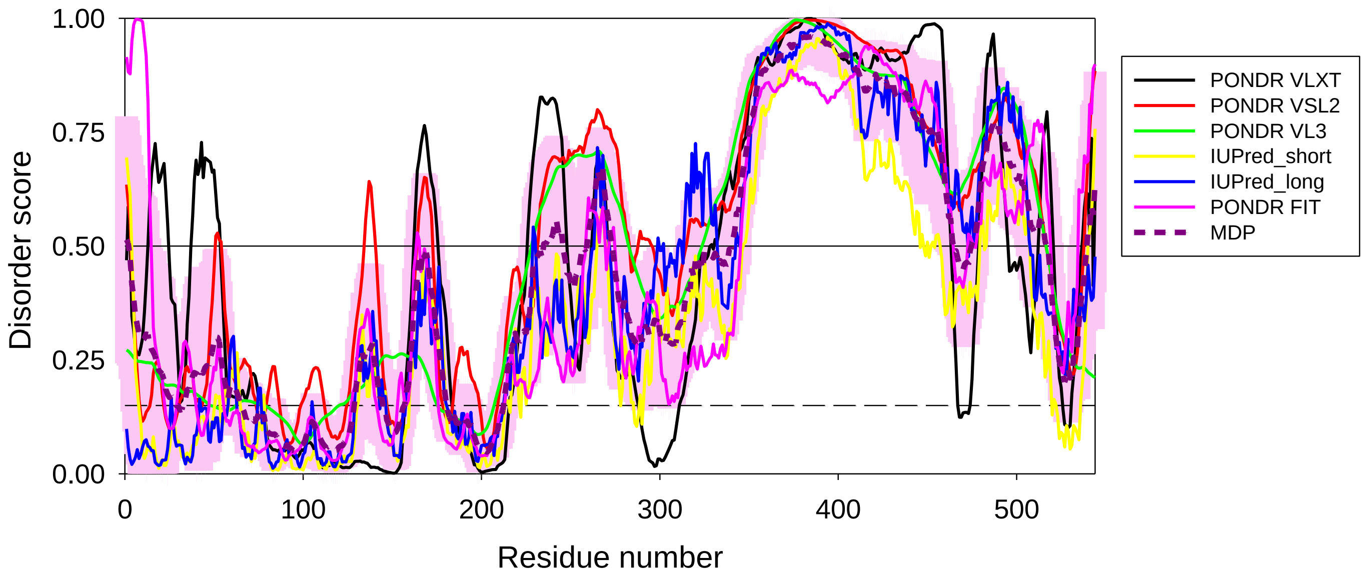

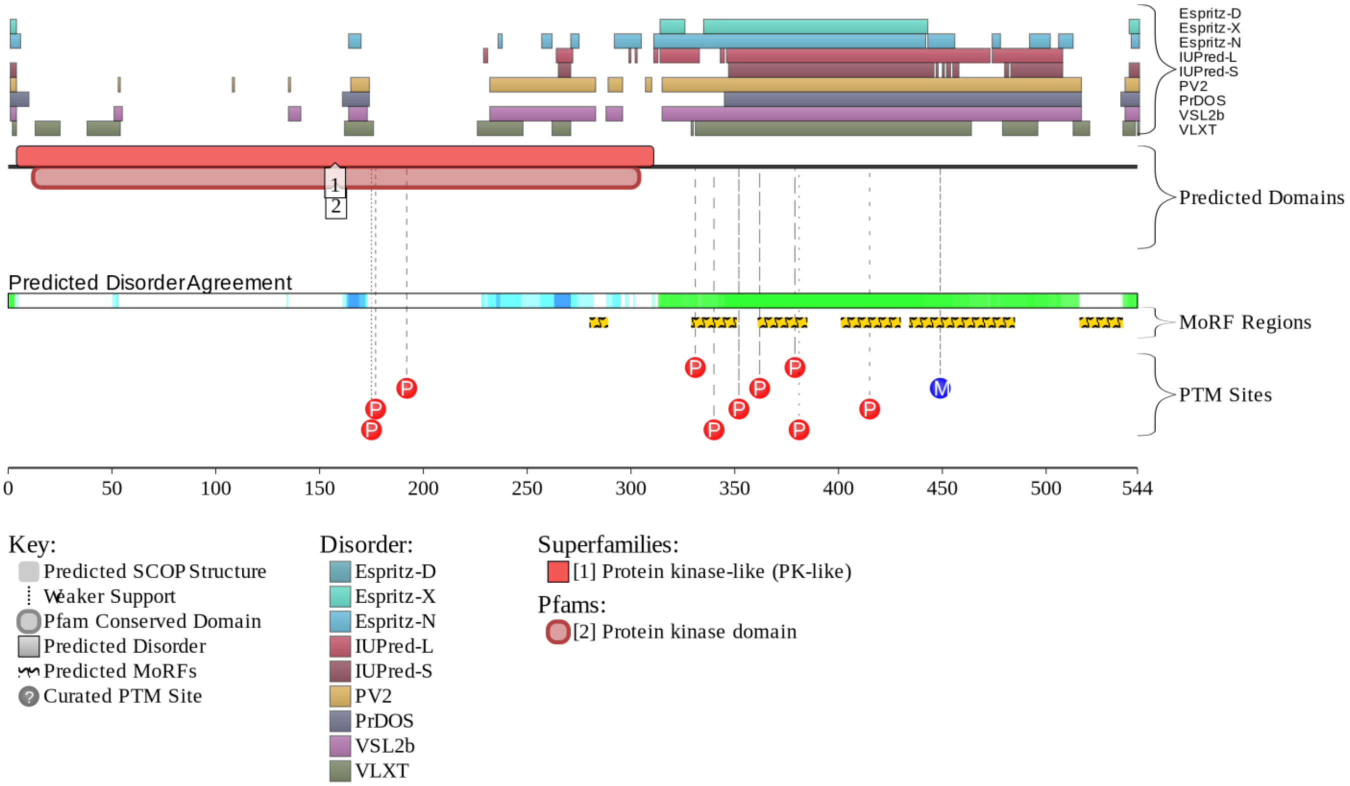

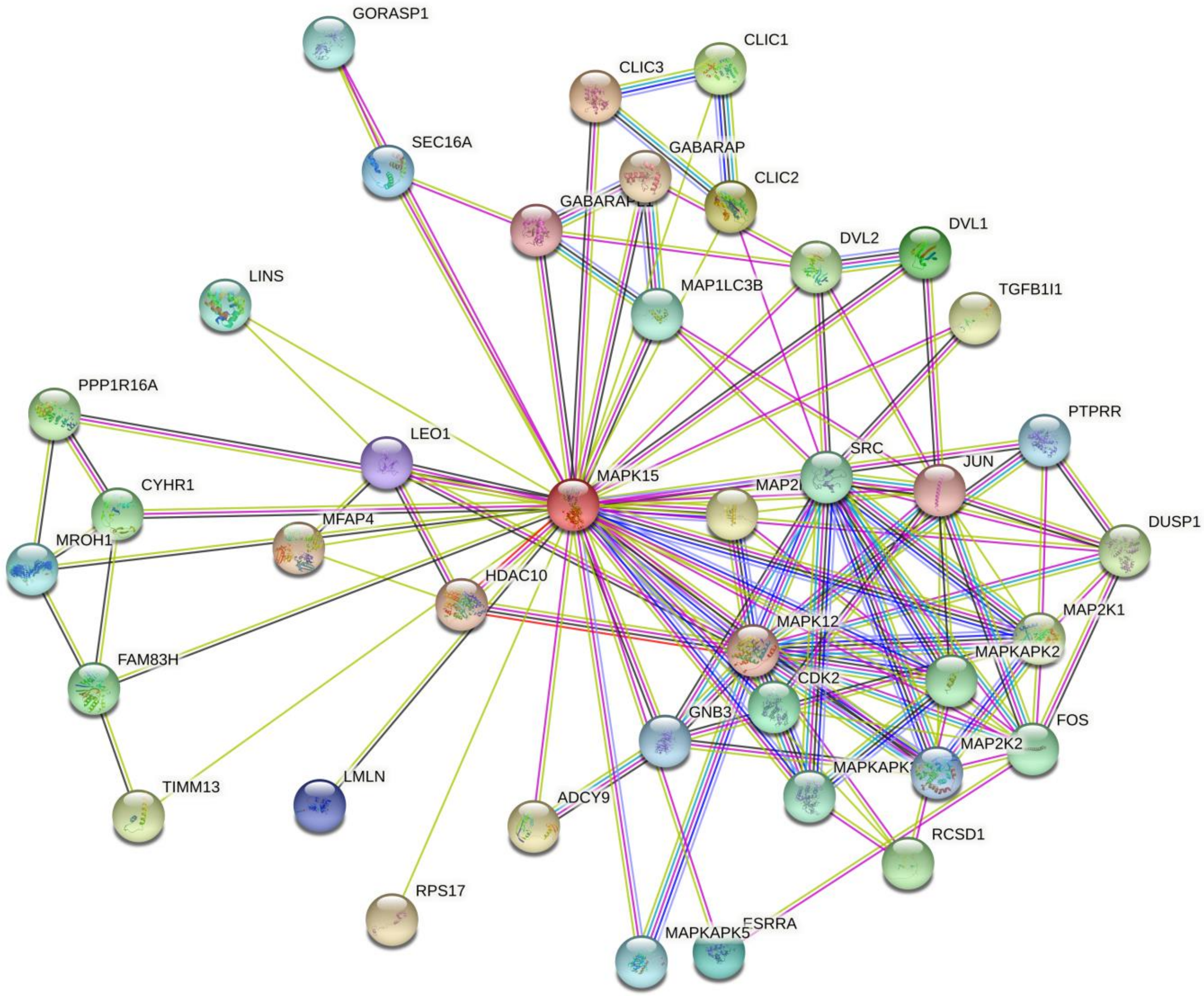

GORASP1
CLIC1
CLIC3
SEC16A
GABARAP
CLIC2
DVL2
DVL1
LINS
MAP1LC3B
TGFB1I1
PPP1R16A
LEO1
PTPRR
SRC
JUN
CYHR1
MAPK15
MFAP4
DUSP1
MROH1
HDAC10
MAP2K1
MAPK12
MAPKAPK2
FAM83H
CDK2
GNB3
FOS
MAP2K2
TIMM13
LMLN
ADCY9
RCSD1
RPS17
ESRRA
MAPKAPK5

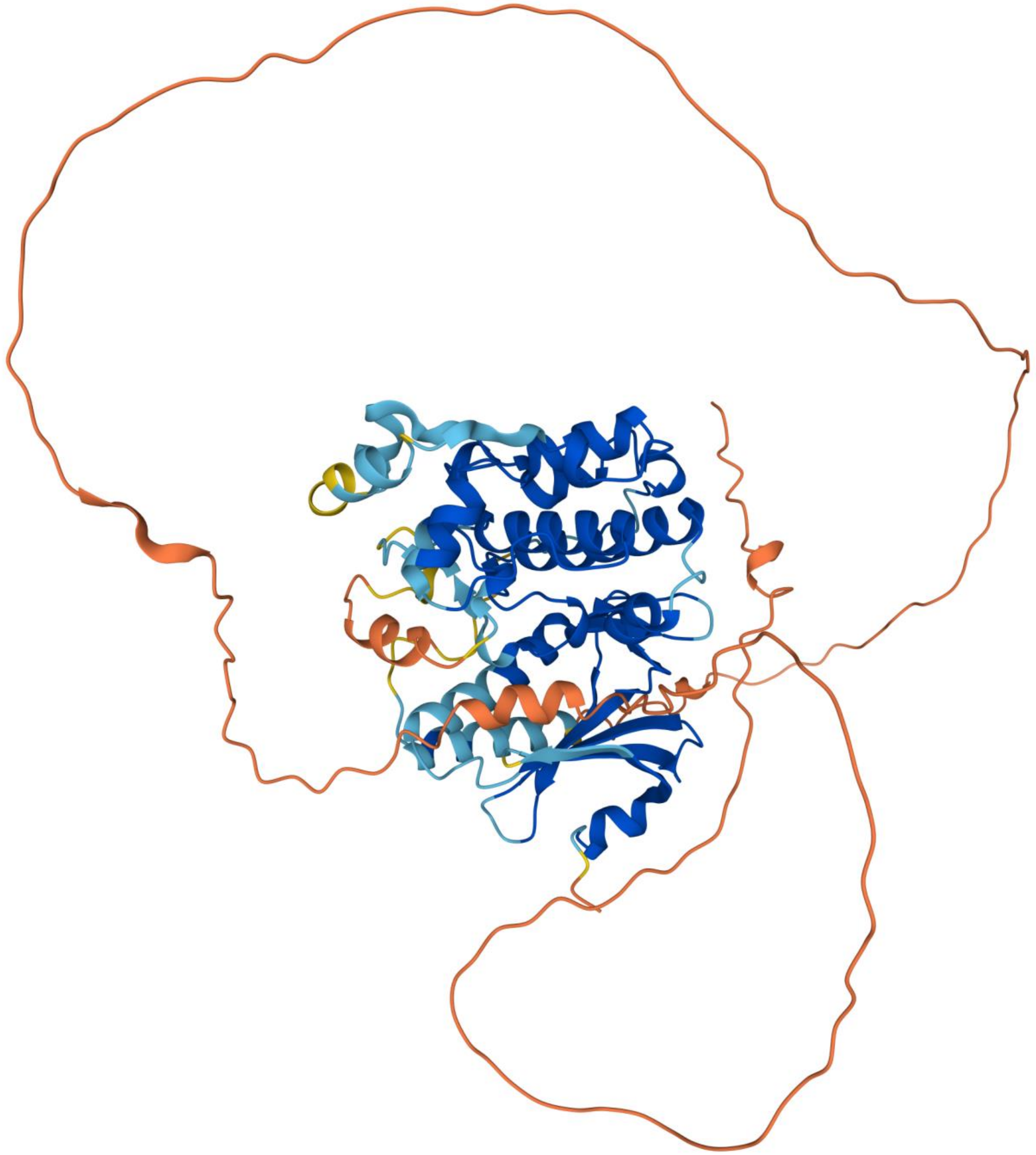

>sp|P05412|JUN_HUMAN Transcription factor Jun OS=Homo sapiens OX=9606 GN=JUN PE=1 SV=2
MTAKMETTFYDDALNASFLPSESGPYGYSNPKILKQSMTLNLADPVGSLKPHLRAKNSDLLTSPDVGLLKLASPELERLIIQSSN
GHITTTPTPTQFLCPKNVTDEQEGFAEGFVRALAELHSQNTLPSVTSAAQPVNGAGMVAPAVASVAGGSGSGGFSASLHSEPPVY
ANLSNFNPGALSSGGGAPSYGAAGLAFPAQPQQQQQPPHHLPQQMPVQHPRLQALKEEPQTVPEMPGETPPLSPIDMESQERIKA
ERKRMRNRIAASKCRKRKLERIARLEEKVKTLKAQNSELASTANMLREQVAQLKQKVMNHVNSGCQLMLTQQLQTF

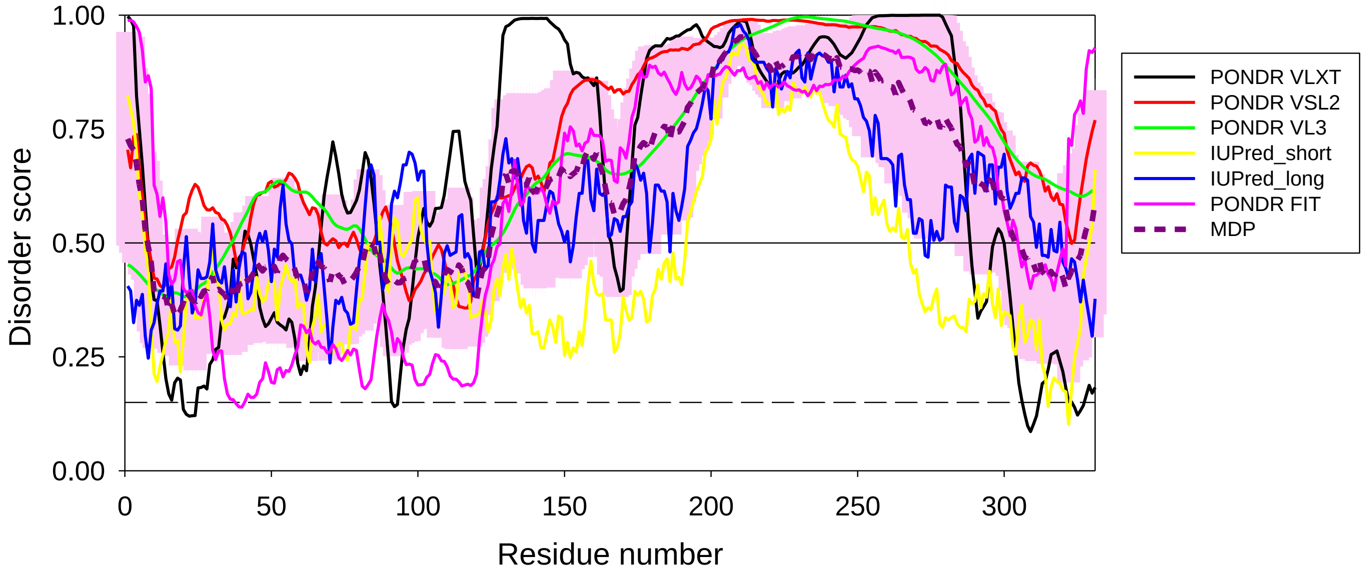

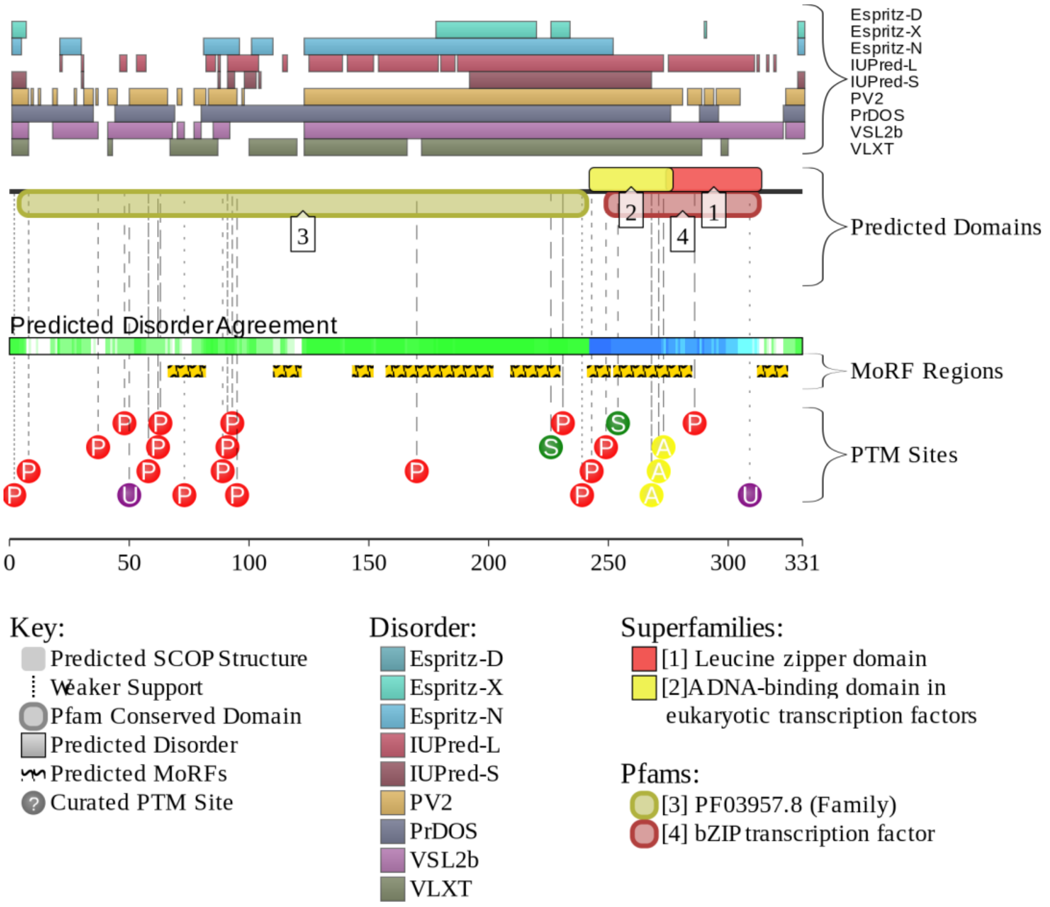

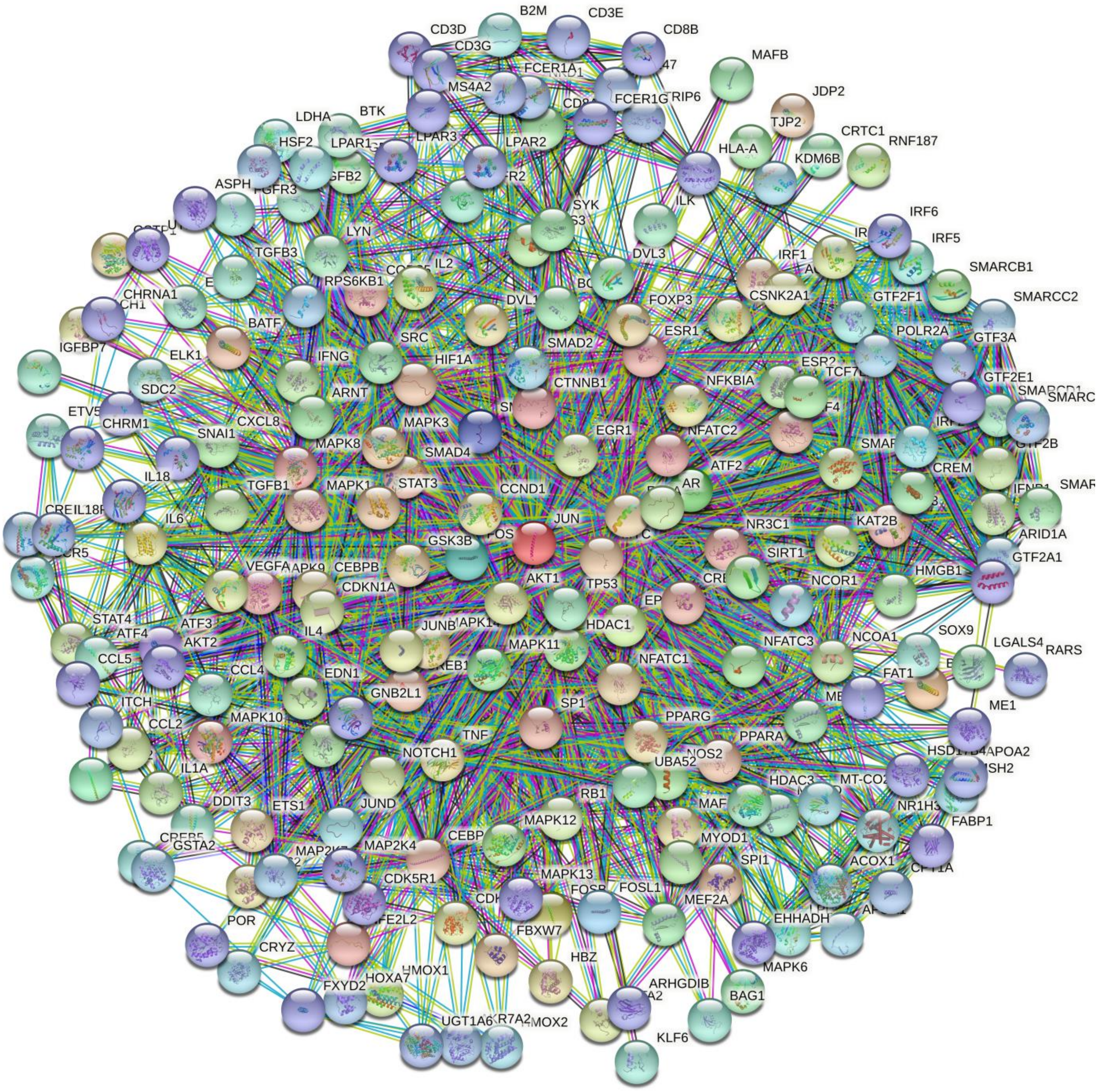
B2M
CD3E
CD3D
CD8B
CD3G
FCER1A
MAFB
MS4A2
FCER1G
JDP2
LDHA
BTK
TJP2
CRTC1
RNF187
HSF2
LPAR1
LPAR3
LPAR2
HLA-A
KDM6B
ASPH
FGFR3
SYK
ILK
IRF6
IRF5
LYN
IL2
DVL3
IRF1
SMARCB1
TGFB3
RPS6KB1
DVL1
FOXP3
CSNK2A1
GTF2F1
SMARCC2
CHRNA1
BATF
ESR1
POLR2A
GTF3A
IGFBP7
ELK1
SRC
IFNG
HIF1A
SMAD2
ESR2
GTF2E1
SDC2
ARNT
CTNNB1
NFKBIA
ETV5
CHRM1
CXCL8
MAPK3
NFATC2
SNAI1
MAPK8
SMAD4
EGR1
IL18
TGFB1
MAPK1
STAT3
CCND1
ATF2
AR
CREM
KAT2B
IL6
JUN
NR3C1
ARID1A
GSK3B
SIRT1
GTF2A1
VEGFA
CEBPB
AKT1
TP53
NCOR1
HMGB1
CDKN1A
STAT4
ATF4
ATF3
IL4
JUNB
HDAC1
SOX9
AKT2
MAPK11
NFATC3
NCOA1
LGALS4
RARS
CCL5
CCL4
NFATC1
FAT1
EDN1
GNB2L1
ME1
ITCH
CCL2
MAPK10
SP1
PPARG
TNF
PPARA
IL1A
NOTCH1
NOS2
UBA52
APOA2
HDAC3
MT-CO2
DDIT3
ETS1
JUND
RB1
NR1H3
MAPK12
FABP1
GSTA2
MAP2K4
CEBP
MYOD1
SPI1
CDK5R1
MAPK13
ACOX1
FOSB
FOSL1
MEF2A
EHHADH
FBXW7
POR
CRYZ
MAPK6
HMOX1
HBZ
FXYD2
HOXA7
ARHGDIB
BAG1
UGT1A6
HMOX2
KLF6

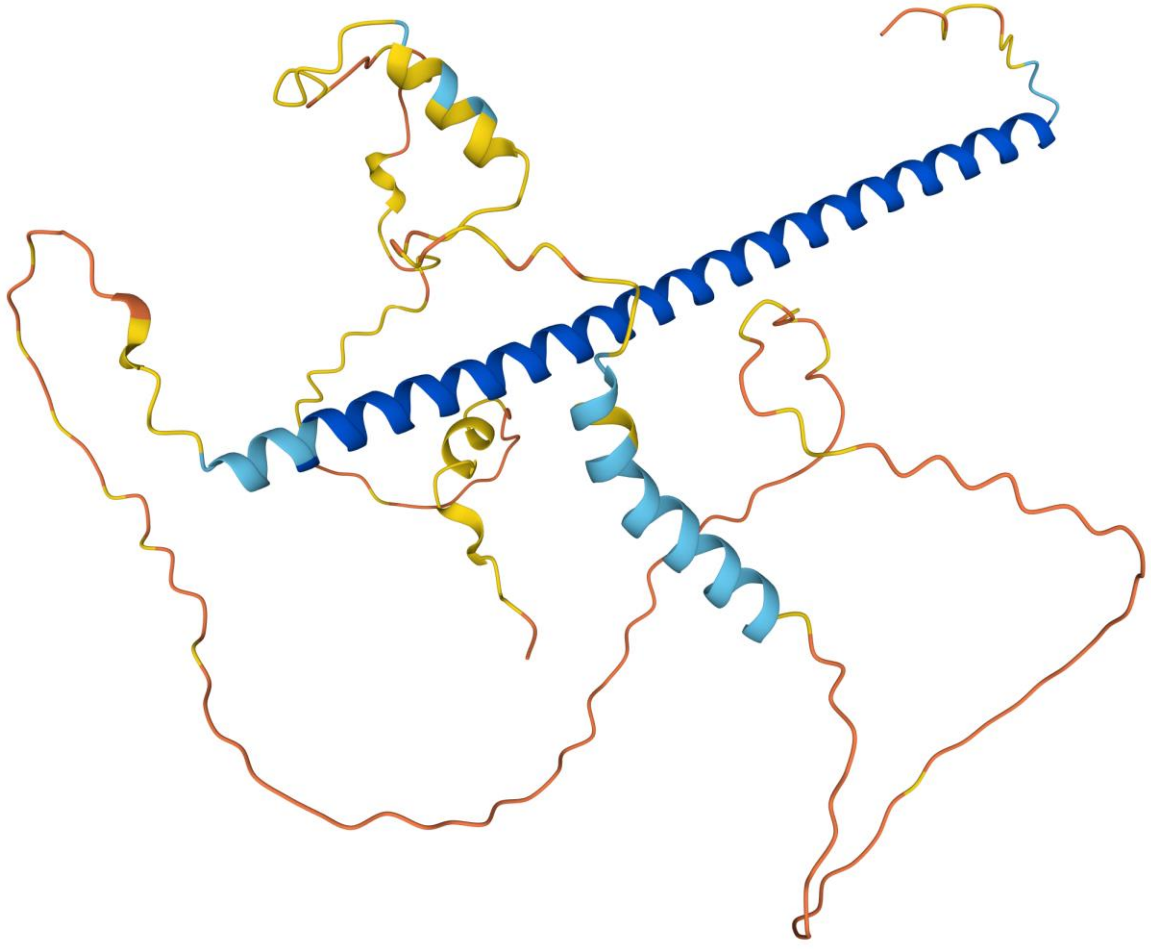

```
>sp|P01100|FOS_HUMAN Protein c-Fos OS=Homo sapiens OX=9606 GN=FOS PE=1 SV=1
MMFSGFNADYEASSSRCSSASPAGDSLSYYHSPADSFSSMGSPVNAQDFCTDLAVSSANFIPTVTAISTSPDLQWLVQPALVSSV
APSQTRAPHPFGVPAPSAGAYSRAGVVKTMTGGRAQSIGRRGKVEQLSPEEEEKRRIRRERNKMAAAKCRNRRRELTDTLQAETD
QLEDEKSALQTEIANLLKEKEKLEFILAAHRPACKIPDDLGFPEEMSVASLDLTGGLPEVATPESEEAFTLPLLNDPEPKPSVEP
VKSISSMELKTEPFDDFLFPASSRPSGSETARSVPDMDLSGSFYAADWEPLHSGSLGMGPMATELEPLCTPVVTCTPSCTAYTSS
FVFTYPEADSFPSCAAAHRKGSSSNEPSSDSLSSPTLLAL
```

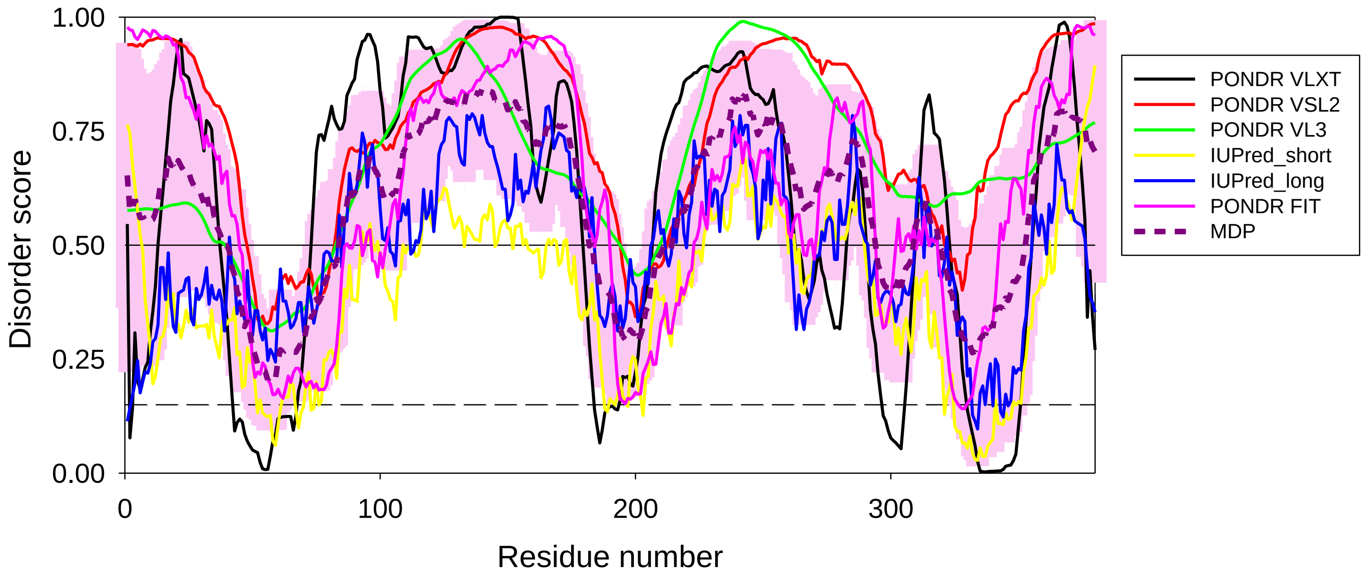

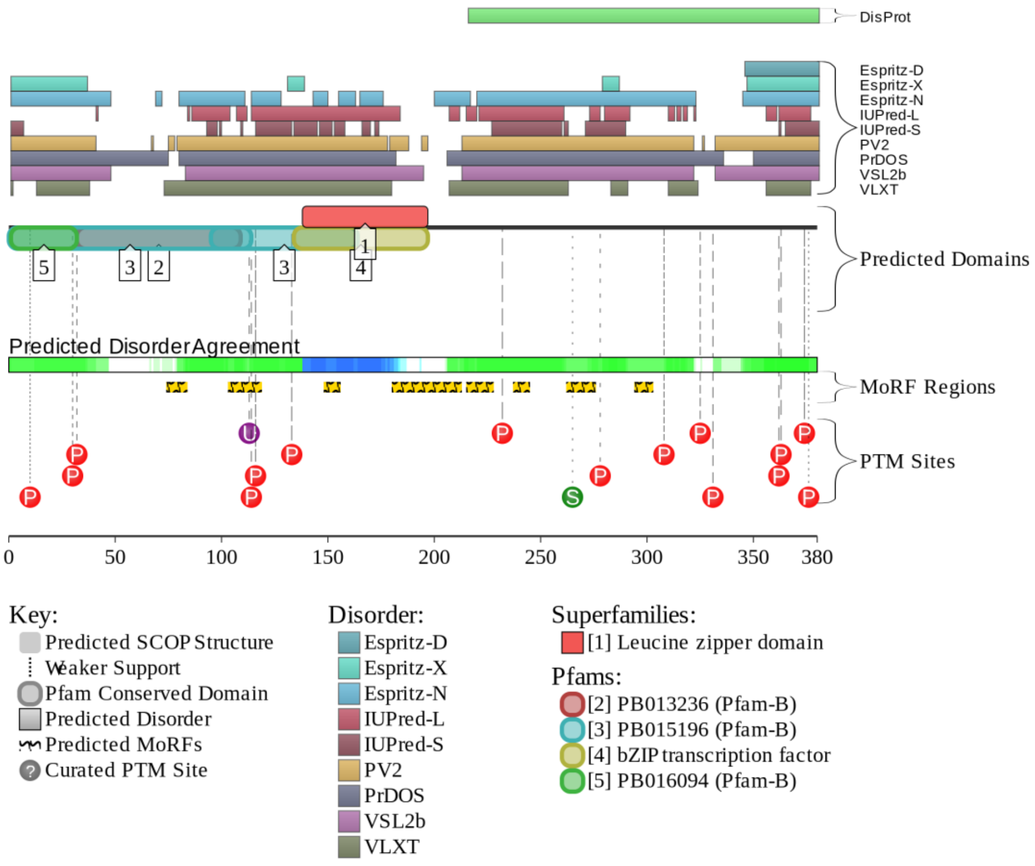

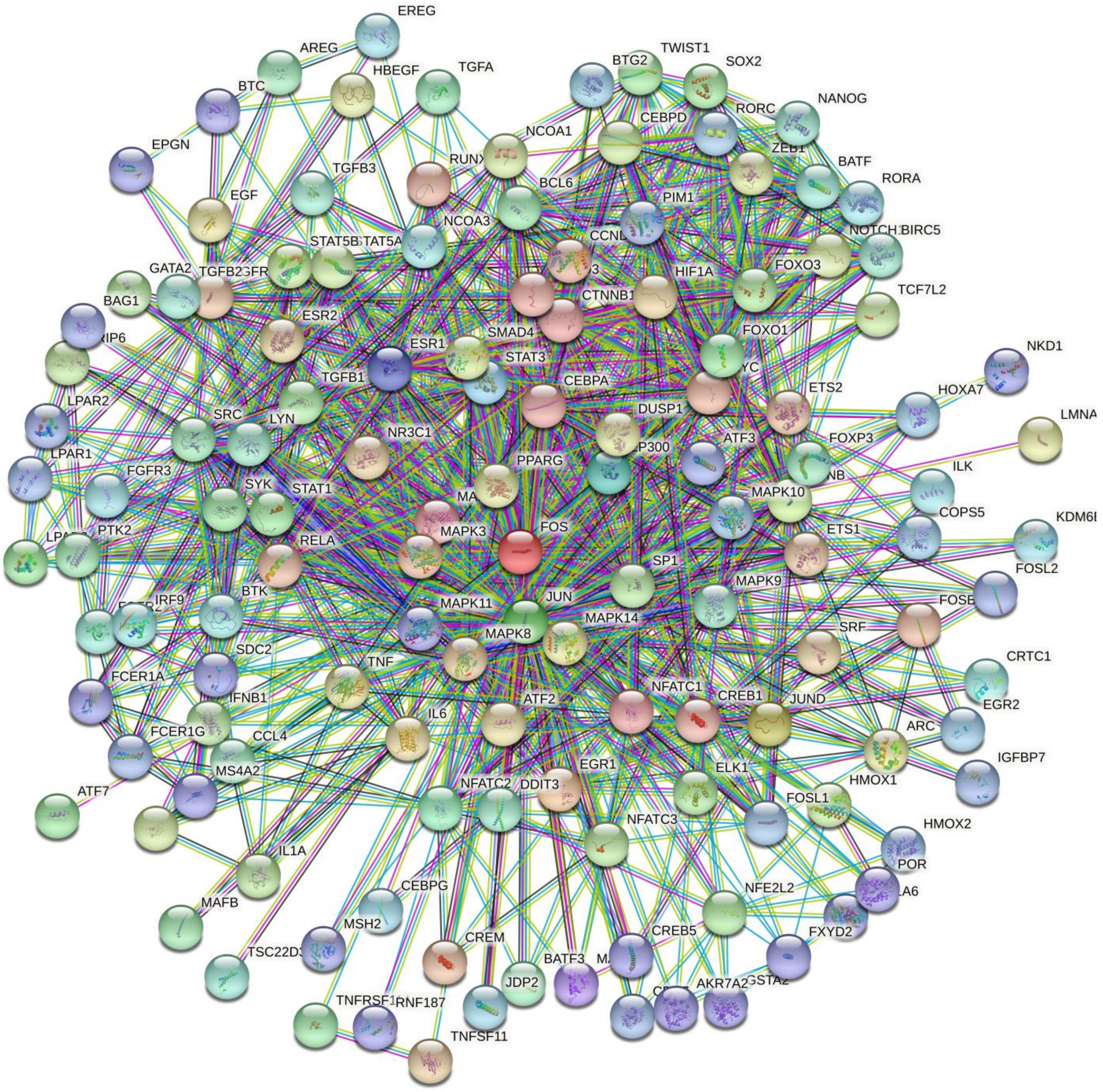
EREG
AREG
HBEGF
TGFA
BTC
TWIST1
BTG2
SOX2
NANOG
RORC
CEBPD
NCOA1
EPGN
BATF
RORA
TGFB3
BCL6
PIM1
EGF
NCOA3
BIRC5
STAT5B
STAT5A
GATA2
HIF1A
FOXO3
BAG1
CTNNB1
TCF7L2
ESR2
FOXO1
SMAD4
ESR1
NKD1
STAT3
TGFB1
CEBPA
ETS2
HOXA7
LMNA
LPAR2
DUSP1
SRC
LYN
NR3C1
ATF3
FOXP3
LPAR1
PPARG
ILK
FGFR3
SYK
STAT1
MAPK10
COPS5
PTK2
FOS
RELA
MAPK3
ETS1
FOSL2
SP1
MAPK9
BTK
IRF9
JUN
MAPK11
MAPK14
MAPK8
SDC2
SRF
CRTC1
TNF
FCER1A
IFNB1
ATF2
NFATC1
CREB1
JUND
EGR2
IL6
ARC
FCER1G
CCL4
IGFBP7
MS4A2
EGR1
ELK1
HMOX1
ATF7
NFATC2
DDIT3
FOSL1
NFATC3
HMOX2
IL1A
POR
CEBPG
NFE2L2
MAFB
MSH2
CREB5
FXYD2
CREM
TSC22D3
BATF3
JDP2
AKR7A2
RNF187
TNFSF11

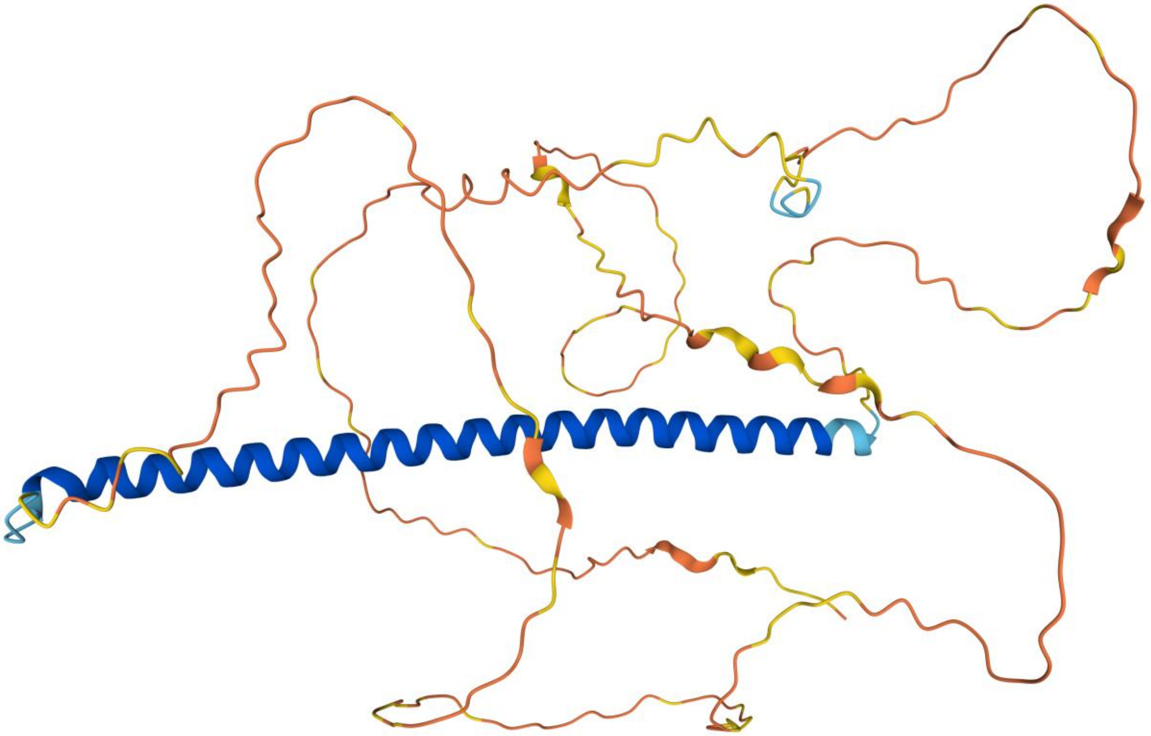

```
>sp|O15111|IKKA_HUMAN Inhibitor of nuclear factor kappa-B kinase subunit alpha
OS=Homo sapiens OX=9606 GN=CHUK PE=1 SV=2
MERPPGLRPGAGGPWEMRERLGTGGFGNVCLYQHRELDLKIAIKSCRLELSTKNRERWCHEIQIMKKLNHANVVKACDVPEELNI
LIHDVPLLAMEYCSGGDLRKLLNKPENCCGLKESQILSLLSDIGSGIRYLHENKIIHRDLKPENIVLQDVGGKIIHKIIDLGYAK
DVDQGSLCTSFVGTLQYLAPELFENKPYTATVDYWSFGTMVFECIAGYRPFLHHLQPFTWHEKIKKKDPKCIFACEEMSGEVRFS
SHLPQPNSLCSLVVEPMENWLQLMLNWDPQQRGGPVDLTLKQPRCFVLMDHILNLKIVHILNMTSAKIISFLLPPDESLHSLQSR
IERETGINTGSQELLSETGISLDPRKPASQCVLDGVRGCDSYMVYLFDKSKTVYEGPFASRSLSDCVNYIVQDSKIQLPIIQLRK
VWAEAVHYVSGLKEDYSRLFQGQRAAMLSLLRYNANLTKMKNTLISASQQLKAKLEFFHKSIQLDLERYSEQMTYGISSEKMLKA
WKEMEEKAIHYAEVGVIGYLEDQIMSLHAEIMELQKSPYGRRQGDLMESLEQRAIDLYKQLKHRPSDHSYSDSTEMVKIIVHTVQ
SQDRVLKELFGHLSKLLGCKQKIIDLLPKVEVALSNIKEADNTVMFMQGKRQKEIWHLLKIACTQSSARSLVGSSLEGAVTPQTS
AWLPPTSAEHDHSLSCVVTPQDGETSAQMIEENLNCLGHLSTIIHEANEEQGNSMMNLDWSWLTE
```

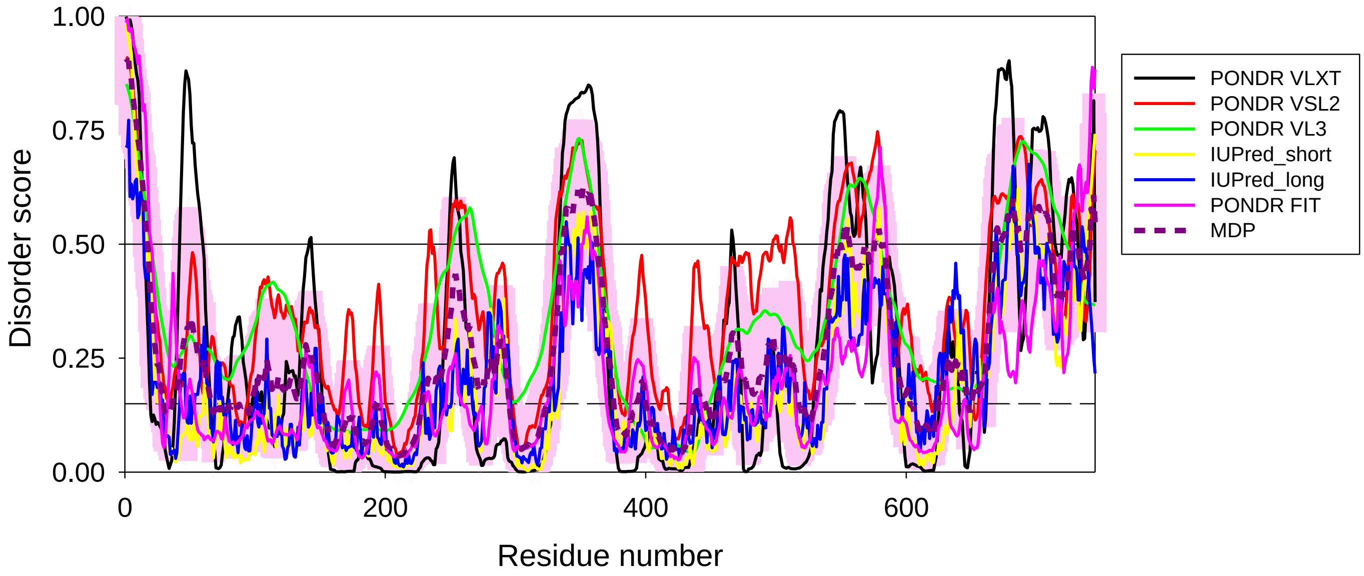

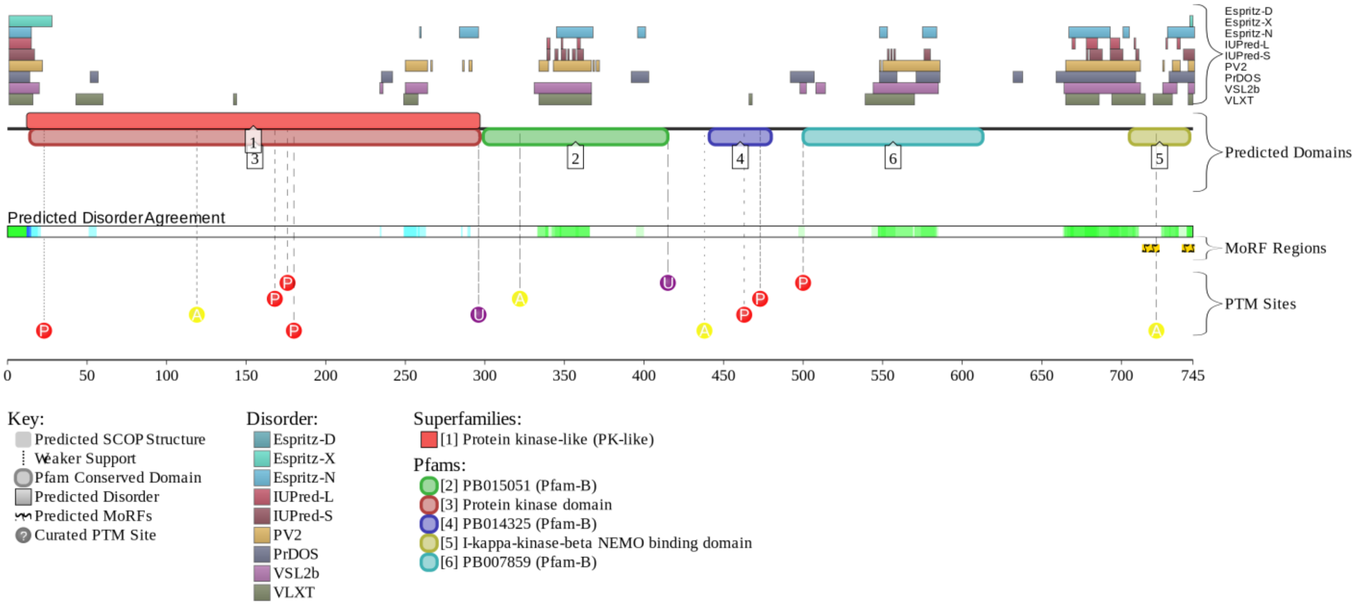

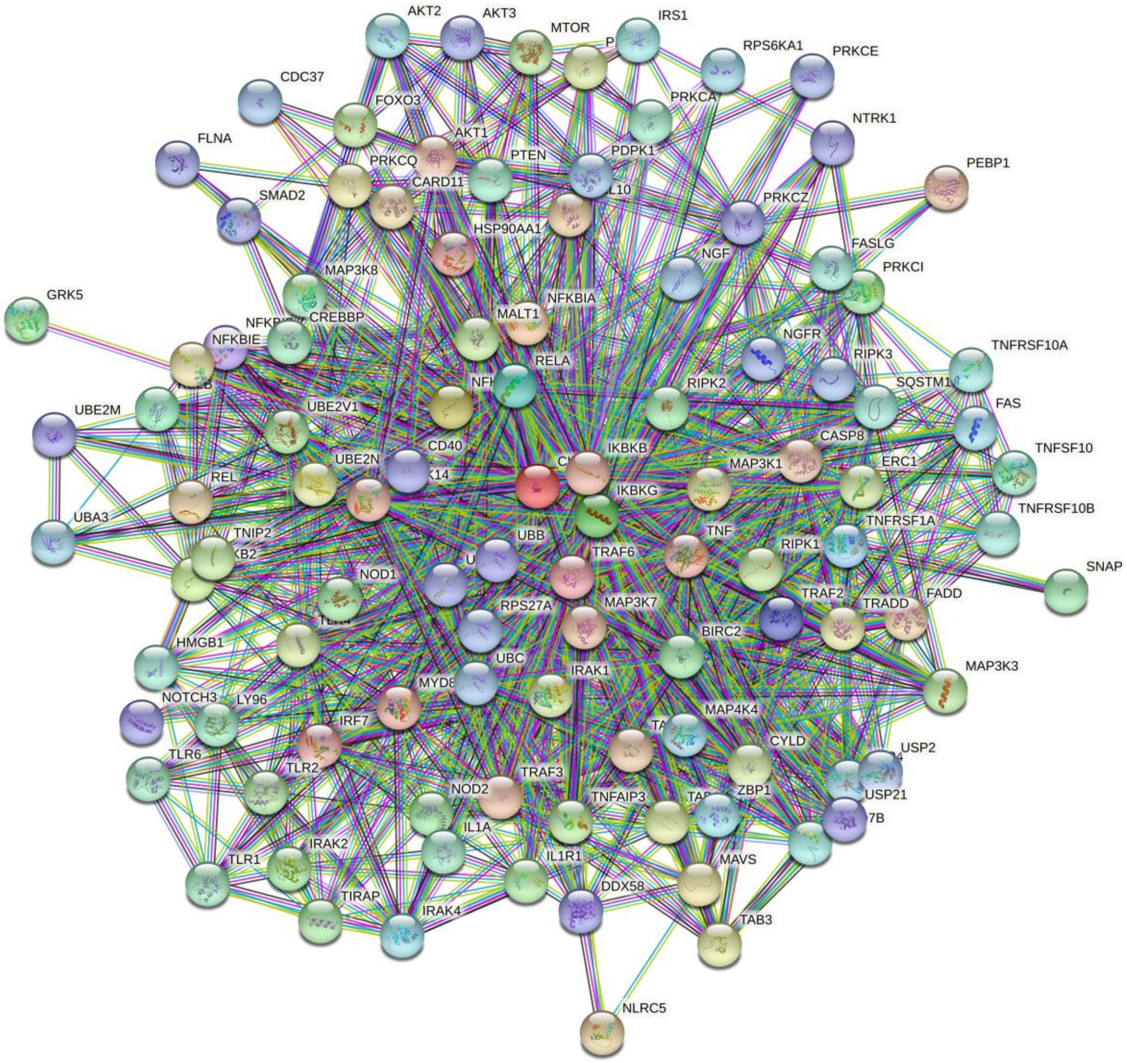
AKT2
AKT3
MTOR
IRS1
RPS6KA1
PRKCE
CDC37
FOXO3
PRKCA
NTRK1
FLNA
AKT1
PTEN
PDPK1
PEBP1
PRKCQ
CARD11
SMAD2
PRKCZ
HSP90AA1
FASLG
NGF
PRKCI
MAP3K8
GRK5
MALT1
NFKBIA
CREBBP
NFKBIE
NGFR
RELA
RIPK3
TNFRSF10A
RIPK2
SQSTM1
FAS
UBE2M
UBE2V1
CD40
CASP8
TNFSF10
UBE2N
IKBKB
MAP3K1
ERC1
REL
IKBKG
TNFRSF10B
UBA3
TNIP2
TNF
TNFRSF1A
UBB
RIPK1
SNAP
TRAF6
NOD1
TRAF2
TRADD
FADD
RPS27A
MAP3K7
BIRC2
HMGB1
UBC
IRAK1
MAP3K3
NOTCH3
LY96
MAP4K4
IRF7
CYLD
USP2
TLR6
TLR2
TRAF3
NOD2
ZBP1
USP21
TNFAIP3
IL1A
TLR1
IRAK2
IL1R1
MAVS
DDX58
TIRAP
IRAK4
TAB3
NLRC5

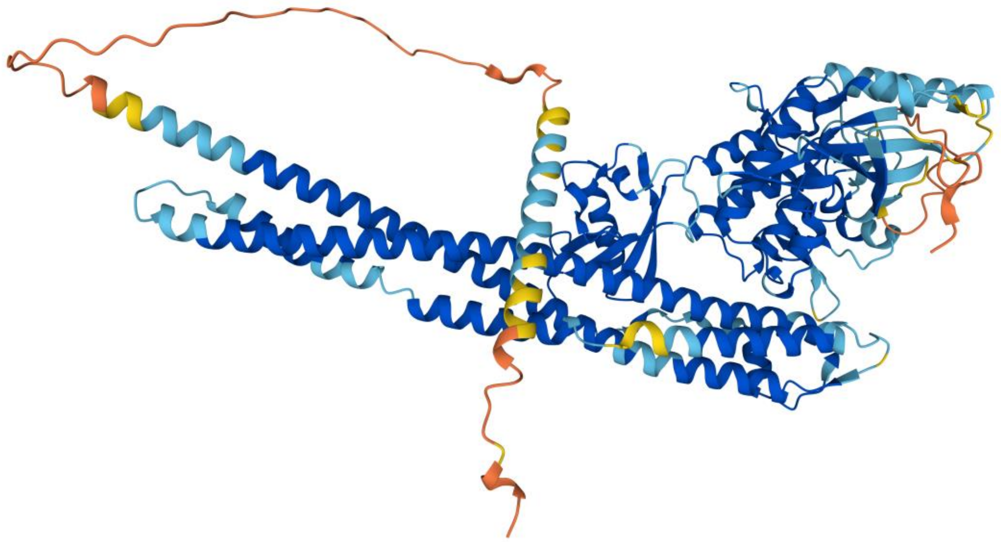

>sp|O14920|IKKB_HUMAN Inhibitor of nuclear factor kappa-B kinase subunit beta OS=Homo sapiens OX=9606 GN=IKBKB PE=1 SV=1
MSWSPSLTTQTCGAWEMKERLGTGGFGNVIRWHNQETGEQIAIKQCRQELSPRNRERWCLEIQIMRRLTHPNVVAARDVPEGMQN
LAPNDLPLLAMEYCQGGDLRKYLNQFENCCGLREGAILTLLSDIASALRYLHENRIIHRDLKPENIVLQQGEQRLIHKIIDLGYA
KELDQGSLCTSFVGTLQYLAPELLEQQKYTVTVDYWSFGTLAFECITGFRPFLPNWQPVQWHSKVRQKSEVDIVVSEDLNGTVKF
SSSLPYPNNLNSVLAERLEKWLQLMLMWHPRQRGTDPTYGPNGCFKALDDILNLKLVHILNMVTGTIHTYPVTEDESLQSLKARI
QQDTGIPEEDQELLQEAGLALIPDKPATQCISDGKLNEGHTLDMDLVFLFDNSKITYETQISPRPQPESVSCILQEPKRNLAFFQ
LRKVWGQVWHSIQTLKEDCNRLQQGQRAAMMNLLRNNSCLSKMKNSMASMSQQLKAKLDFFKTSIQIDLEKYSEQTEFGITSDKL
LLAWREMEQAVELCGRENEVKLLVERMMALQTDIVDLQRSPMGRKQGGTLDDLEEQARELYRRLREKPRDQRTEGDSQEMVRLLL
QAIQSFEKKVRVIYTQLSKTVVCKQKALELLPKVEEVVSLMNEDEKTVVRLQEKRQKELWNLLKIACSKVRGPVSGSPDSMNASR
LSQPGQLMSQPSTASNSLPEPAKKSEELVAEAHNLCTLLENAIQDTVREQDQSFTALDWSWLQTEEEEHSCLEQAS

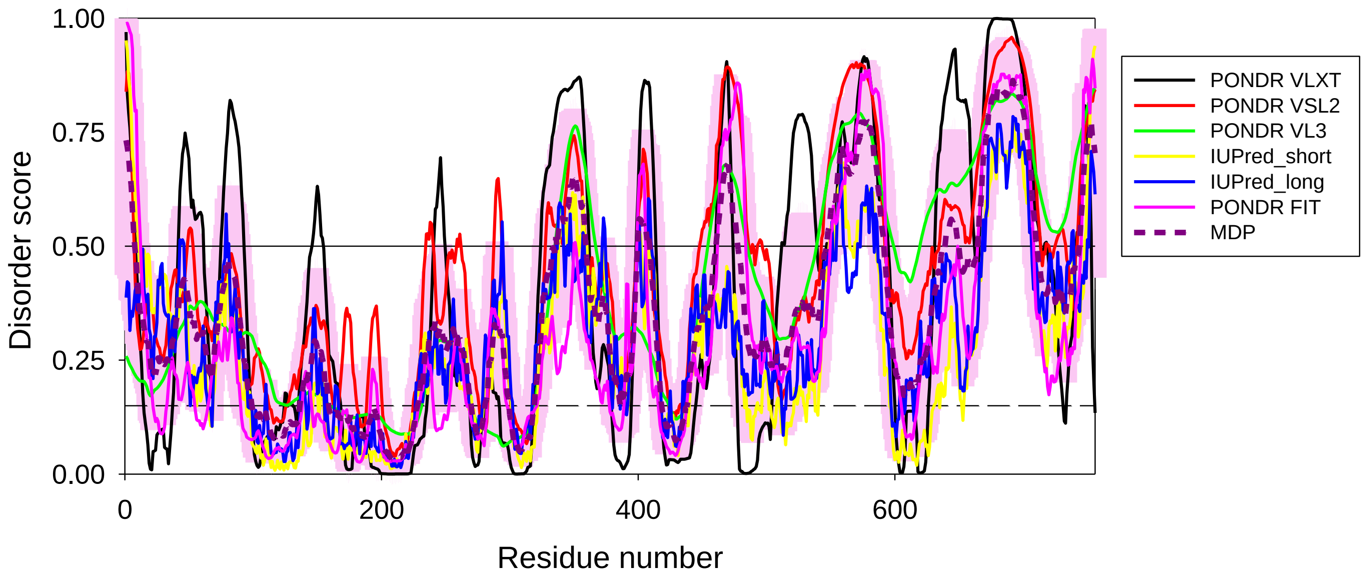

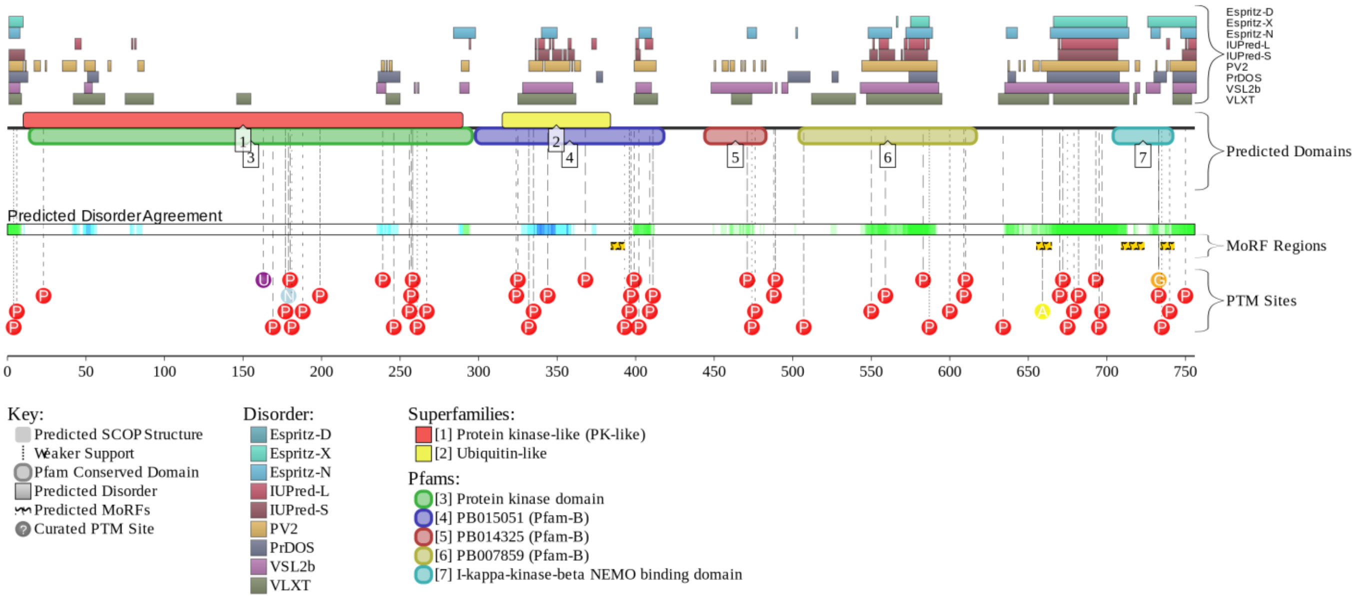

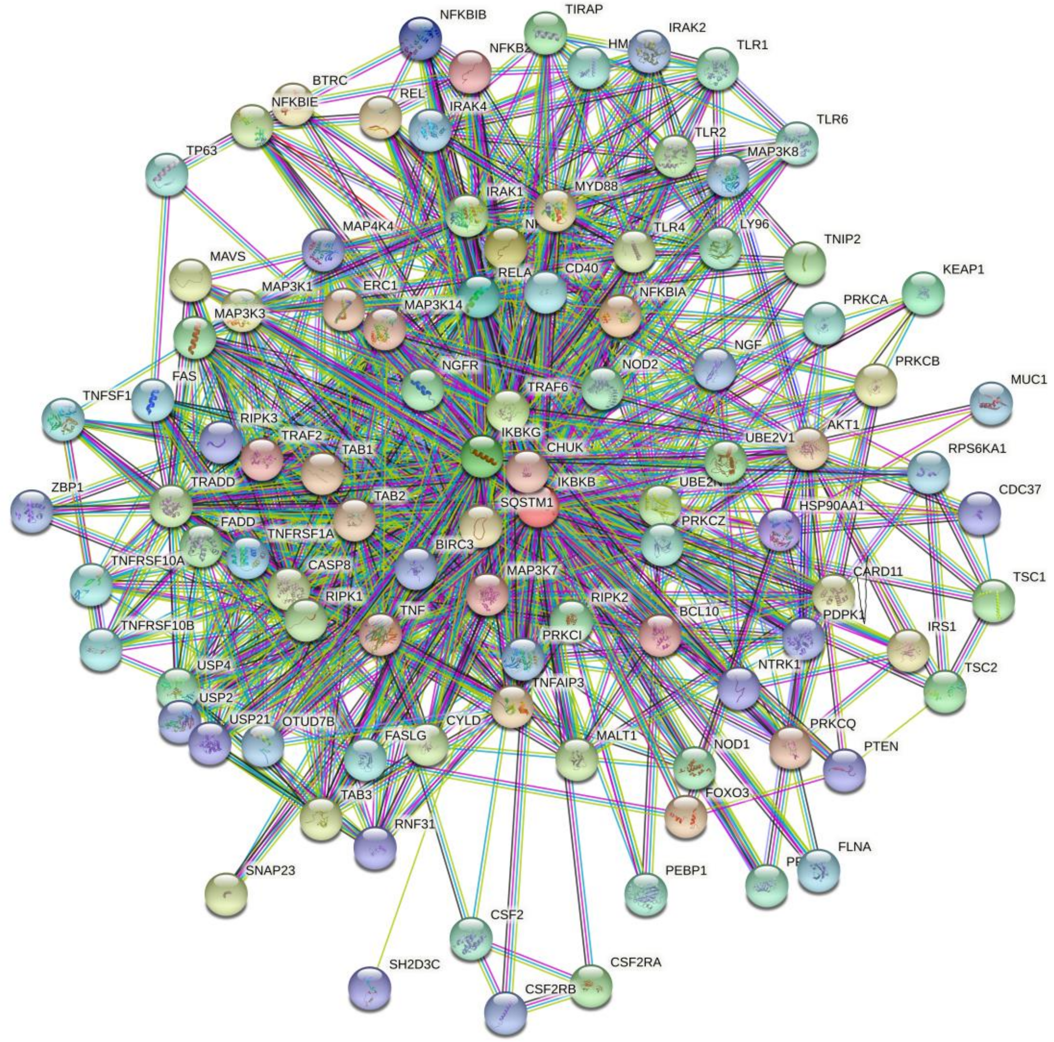
NFKBIB
TIRAP
IRAK2
TLR1
BTRC
REL
NFKBIE
IRAK4
TLR6
TP63
TLR2
MAP3K8
IRAK1
MYD88
MAP4K4
TLR4
LY96
TNIP2
MAVS
RELA
CD40
KEAP1
MAP3K1
ERC1
NFKBIA
MAP3K14
PRKCA
MAP3K3
NGF
PRKCB
FAS
NOD2
NGFR
MUC1
TRAF6
RIPK3
AKT1
IKBKG
UBE2V1
TRAF2
CHUK
TAB1
RPS6KA1
ZBP1
TRADD
IKBKB
CDC37
TAB2
SQSTM1
HSP90AA1
FADD
TNFRSF1A
PRKCZ
BIRC3
TNFRSF10A
CASP8
CARD11
TSC1
MAP3K7
RIPK2
RIPK1
TNF
BCL10
PDPK1
TNFRSF10B
IRS1
PRKCI
USP4
NTRK1
TSC2
TNFAIP3
USP2
USP21
OTUD7B
CYLD
PRKCQ
FASLG
MALT1
NOD1
PTEN
TAB3
FOXO3
RNF31
FLNA
PEBP1
SNAP23
CSF2
CSF2RA
SH2D3C
CSF2RB

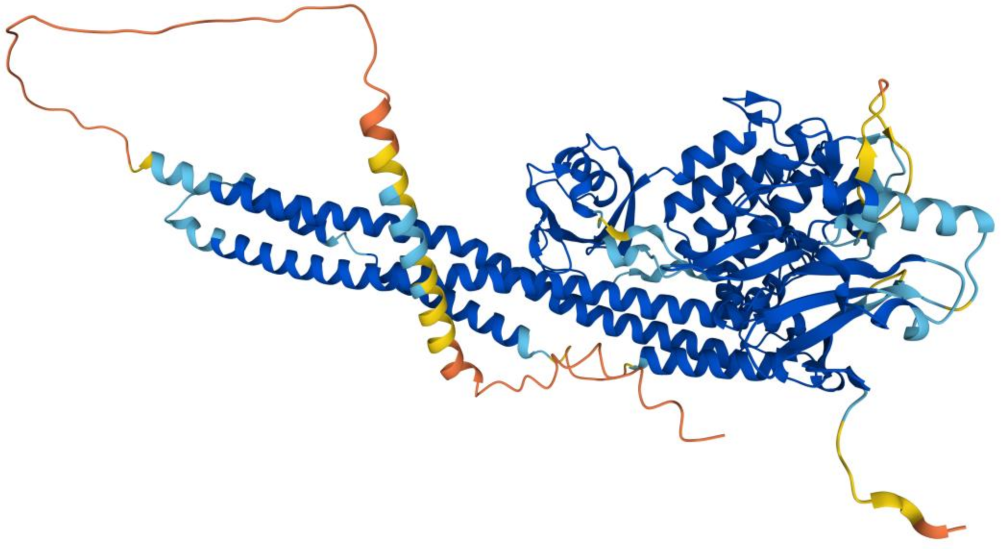

>sp|Q9Y6K9|NEMO_HUMAN NF-kappa-B essential modulator OS=Homo sapiens OX=9606 GN=IKBKG PE=1 SV=2
MNRHLWKSQLCEMVQPSGGPAADQDVLGEESPLGKPAMLHLPSEQGAPETLQRCLEENQELRDAIRQSNQILRERCEELLHFQAS
QREEKEFLMCKFQEARKLVERLGLEKLDLKRQKEQALREVEHLKRCQQQMAEDKASVKAQVTSLLGELQESQSRLEAATKECQAL
EGRARAASEQARQLESEREALQQQHSVQVDQLRMQGQSVEAALRMERQAASEEKRKLAQLQVAYHQLFQEYDNHIKSSVVGSERK
RGMQLEDLKQQLQQAEEALVAKQEVIDKLKEEAEQHKIVMETVPVLKAQADIYKADFQAERQAREKLAEKKELLQEQLEQLQREY
SKLKASCQESARIEDMRKRHVEVSQAPLPPAPAYLSSPLALPSQRRSPPEEPPDFCCPKCQYQAPDMDTLQIHVMECIE

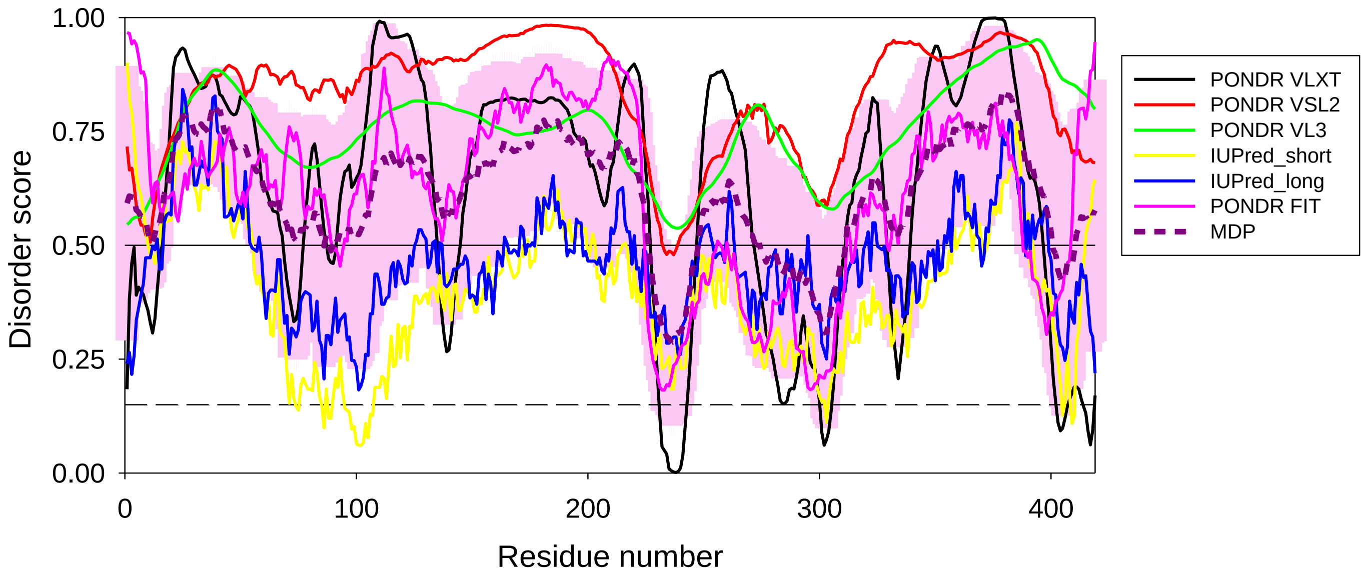

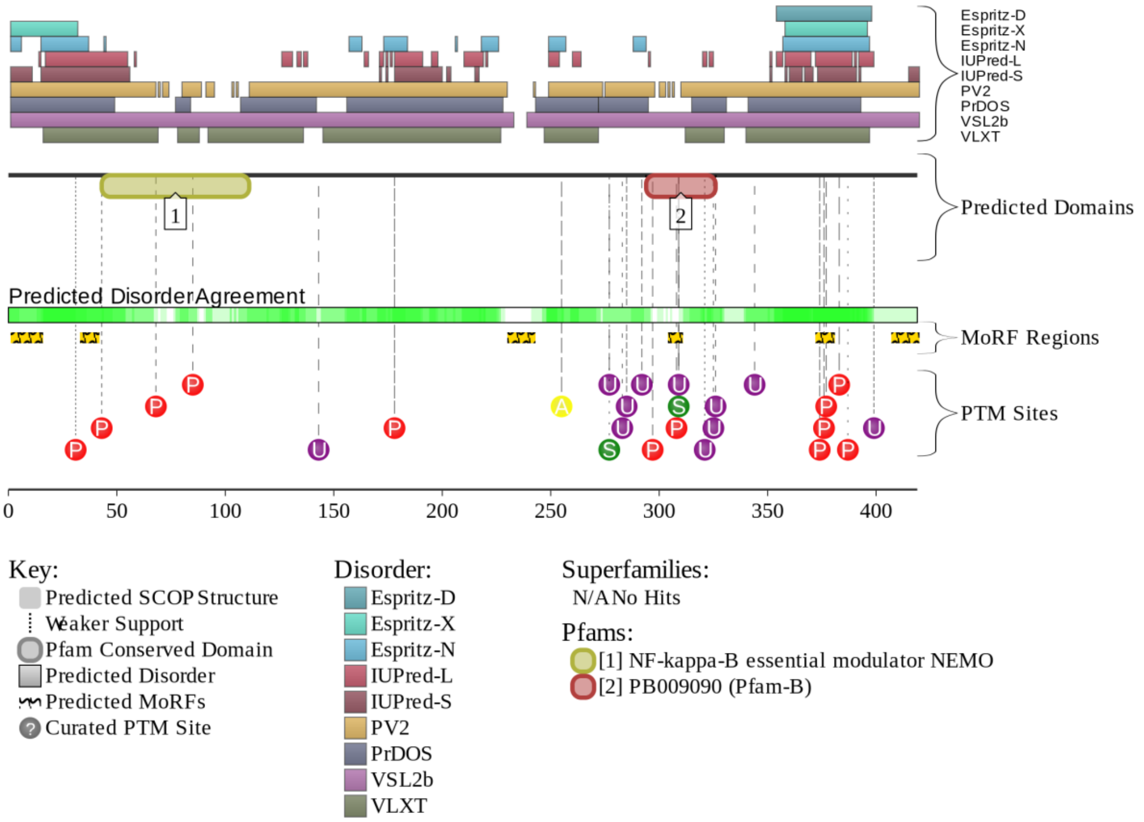

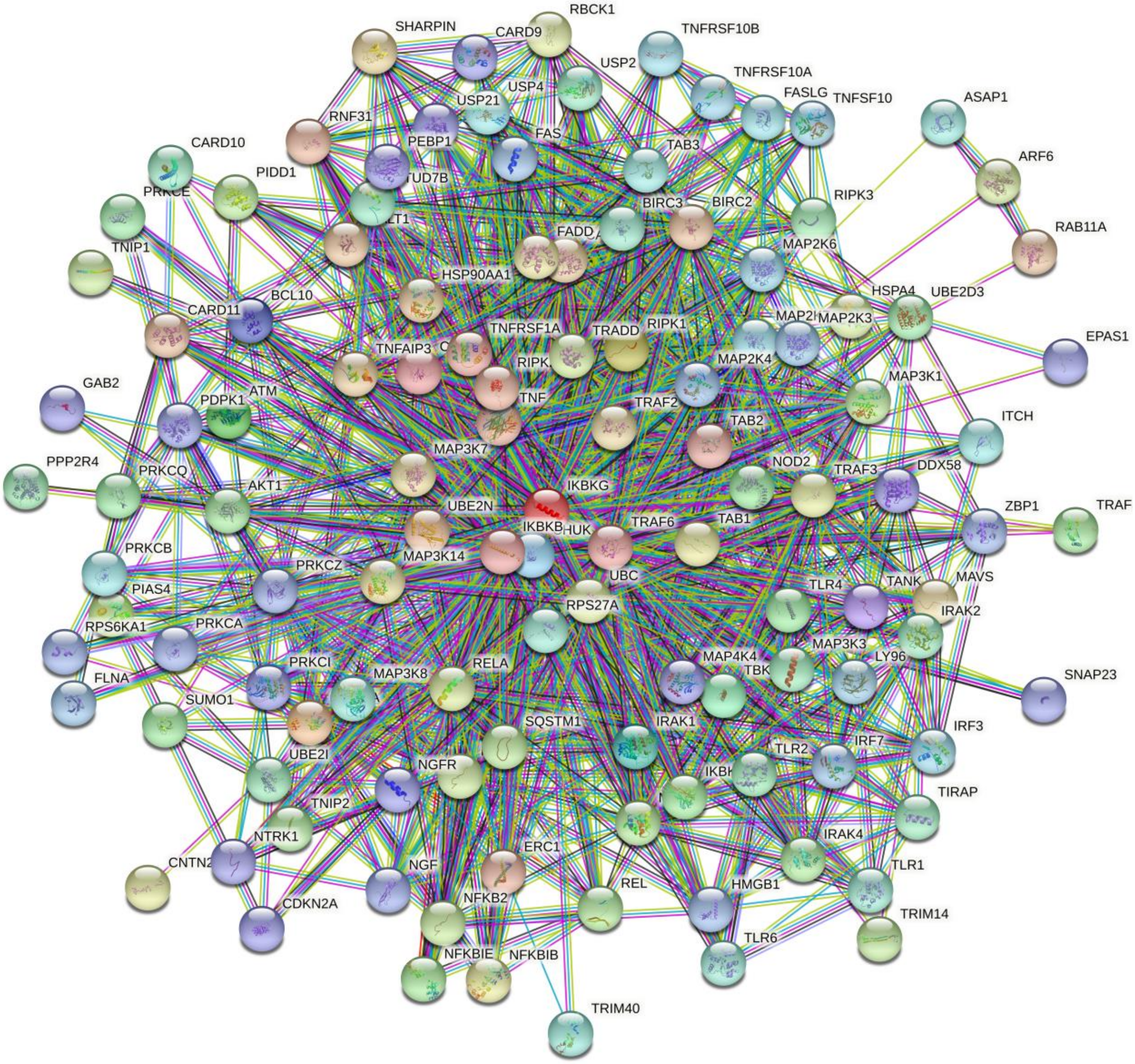
SHARPIN
RBCK1
CARD9
TNFRSF10B
USP2
USP4
TNFRSF10A
USP21
FASLG
TNFSF10
ASAP1
RNF31
FAS
PEBP1
TAB3
CARD10
ARF6
PIDD1
BIRC3
BIRC2
RIPK3
RAB11A
TNIP1
FADD
MAP2K6
HSP90AA1
HSPA4
UBE2D3
BCL10
CARD11
MAP2K3
TNFRSF1A
TRADD
RIPK1
EPAS1
TNFAIP3
MAP2K4
GAB2
ATM
TNF
MAP3K1
PDPK1
TRAF2
TAB2
ITCH
PPP2R4
MAP3K7
NOD2
TRAF3
DDX58
PRKCQ
AKT1
IKBKG
ZBP1
TRAF
UBE2N
IKBKB
TRAF6
TAB1
PRKCB
MAP3K14
UBC
TANK
MAVS
PIAS4
PRKCZ
TLR4
RPS27A
IRAK2
RPS6KA1
PRKCA
MAP4K4
MAP3K3
LY96
SNAP23
FLNA
PRKCI
MAP3K8
RELA
SUMO1
SQSTM1
IRAK1
IRF3
IRF7
TLR2
UBE2I
NGFR
TIRAP
TNIP2
NTRK1
IRAK4
ERC1
NGF
TLR1
REL
HMGB1
CDKN2A
NFKB2
TRIM14
TLR6
NFKBIE
NFKBIB
TRIM40

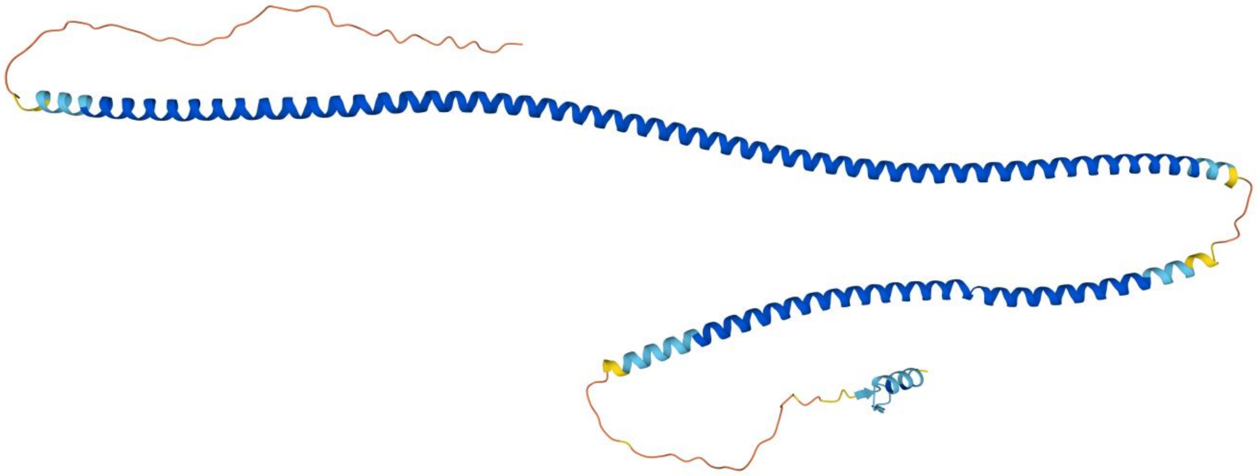

>sp|Q14164|IKKE_HUMAN Inhibitor of nuclear factor kappa-B kinase subunit epsilon OS=Homo sapiens OX=9606 GN=IKBKE PE=1 SV=1
MQSTANYLWHTDDLLGQGATASVYKARNKKSGELVAVKVFNTTSYLRPREVQVREFEVLRKLNHQNIVKLFAVEETGGSRQKVLV
MEYCSSGSLLSVLESPENAFGLPEDEFLVVLRCVVAGMNHLRENGIVHRDIKPGNIMRLVGEEGQSIYKLTDFGAARELDDDEKF
VSVYGTEEYLHPDMYERAVLRKPQQKAFGVTVDLWSIGVTLYHAATGSLPFIPFGGPRRNKEIMYRITTEKPAGAIAGAQRRENG
PLEWSYTLPITCQLSLGLQSQLVPILANILEVEQAKCWGFDQFFAETSDILQRVVVHVFSLSQAVLHHIYIHAHNTIAIFQEAVH
KQTSVAPRHQEYLFEGHLCVLEPSVSAQHIAHTTASSPLTLFSTAIPKGLAFRDPALDVPKFVPKVDLQADYNTAKGVLGAGYQA
LRLARALLDGQELMFRGLHWVMEVLQATCRRTLEVARTSLLYLSSSLGTERFSSVAGTPEIQELKAAAELRSRLRTLAEVLSRCS
QNITETQESLSSLNRELVKSRDQVHEDRSIQQIQCCLDKMNFIYKQFKKSRMRPGLGYNEEQIHKLDKVNFSHLAKRLLQVFQEE
CVQKYQASLVTHGKRMRVVHETRNHLRLVGCSVAACNTEAQGVQESLSKLLEELSHQLLQDRAKGAQASPPPIAPYPSPTRKDLL
LHMQELCEGMKLLASDLLDNNRIIERLNRVPAPPDV

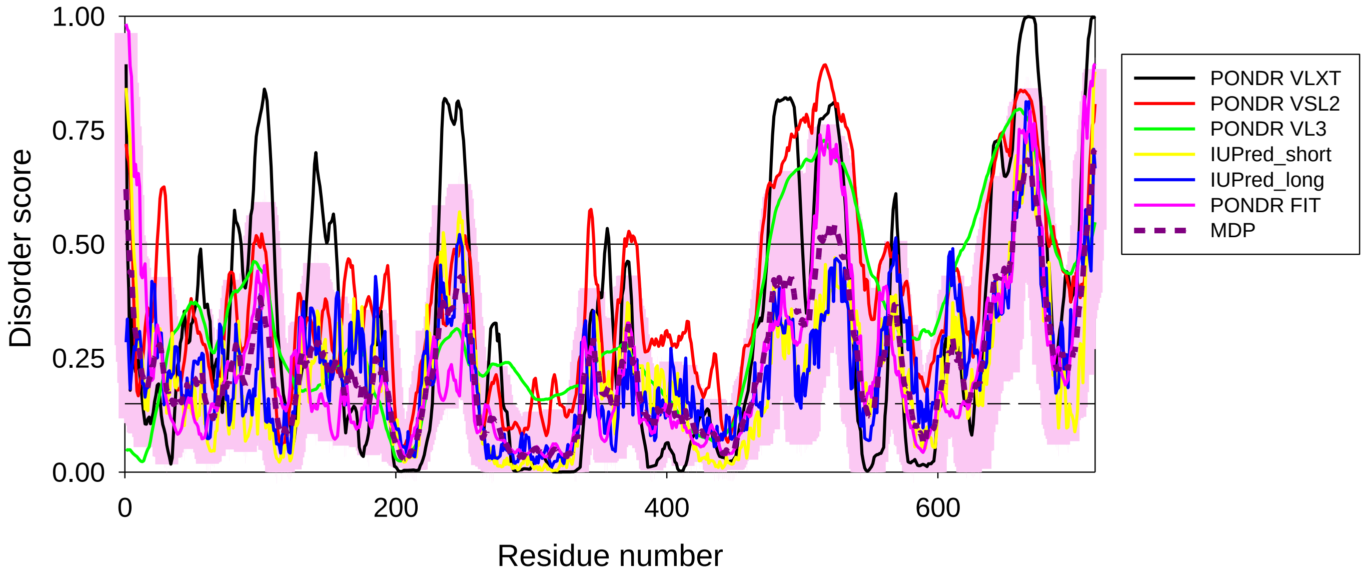

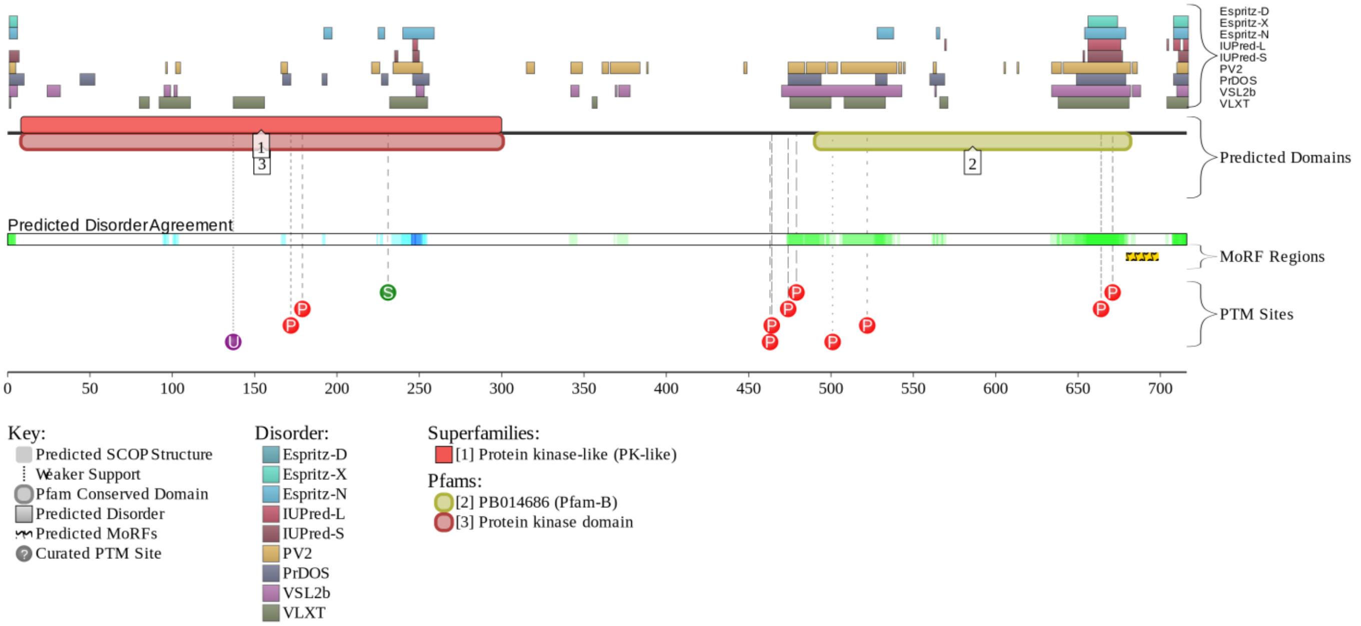

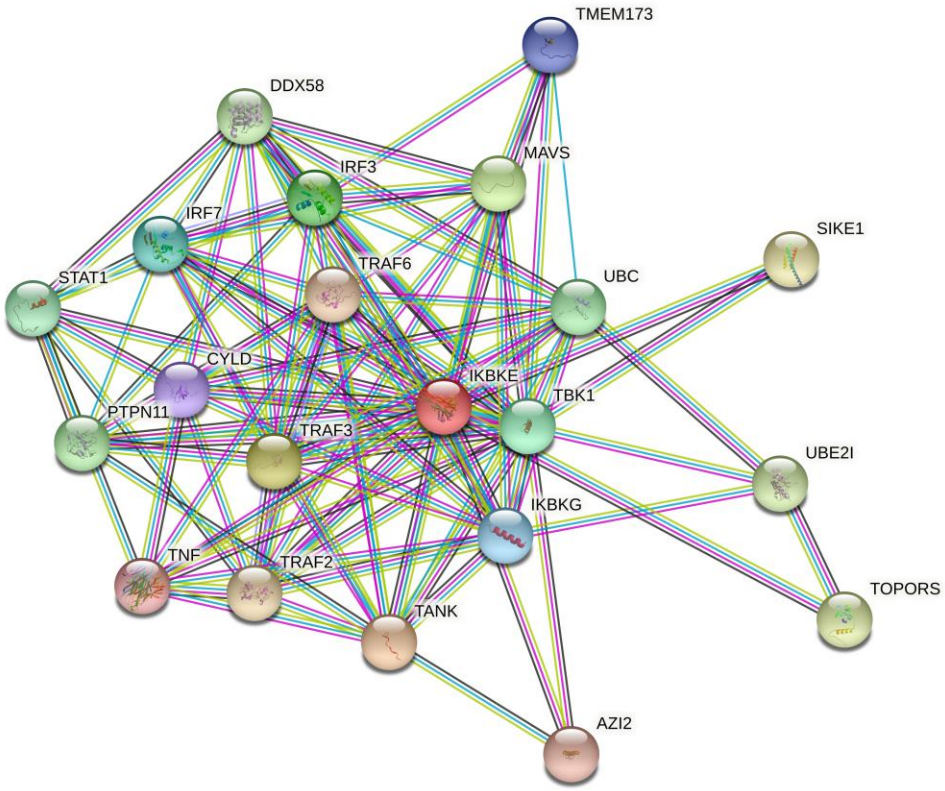
TMEM173
DDX58
MAVS
IRF3
IRF7
SIKE1
TRAF6
STAT1
UBC
CYLD
IKBKE
TBK1
PTPN11
TRAF3
UBE2I
IKBKG
TNF
TRAF2
TANK
TOPORS
AZI2

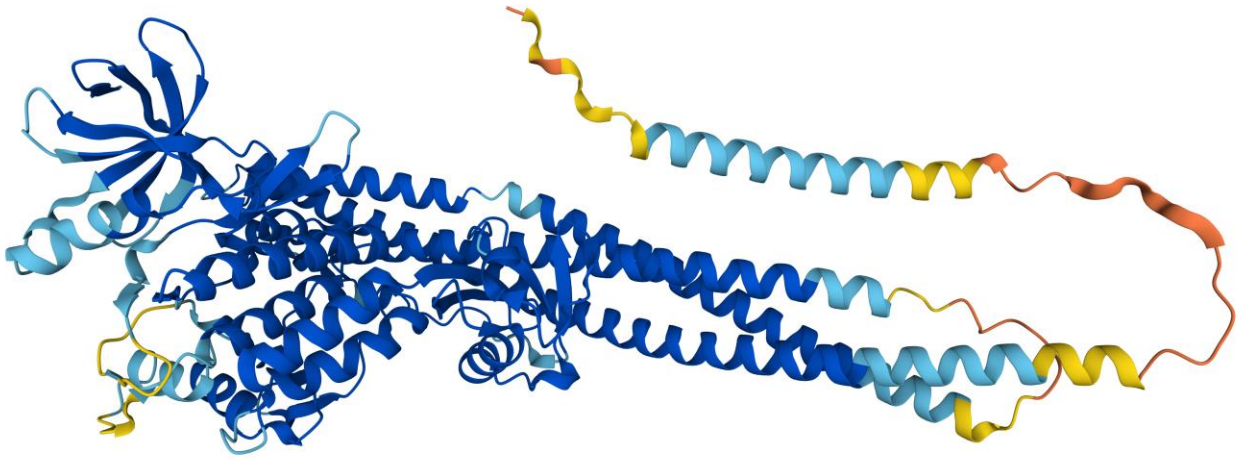

>sp|P25963|IKBA_HUMAN NF-kappa-B inhibitor alpha OS=Homo sapiens OX=9606 GN=NFKBIA PE=1 SV=1
MFQAAERPQEWAMEGPRDGLKKERLLDDRHDSGLDSMKDEEYEQMVKELQEIRLEPQEVPRGSEPWKQQLTEDGDSFLHLAIIHE
EKALTMEVIRQVKGDLAFLNFQNNLQQTPLHLAVITNQPEIAEALLGAGCDPELRDFRGNTPLHLACEQGCLASVGVLTQSCTTP
HLHSILKATNYNGHTCLHLASIHGYLGIVELLVSLGADVNAQEPCNGRTALHLAVDLQNPDLVSLLLKCGADVNRVTYQGYSPYQ
LTWGRPSTRIQQQLGQLTLENLQMLPESEDEESYDTESEFTEFTEDELPYDDCVFGGQRLTL

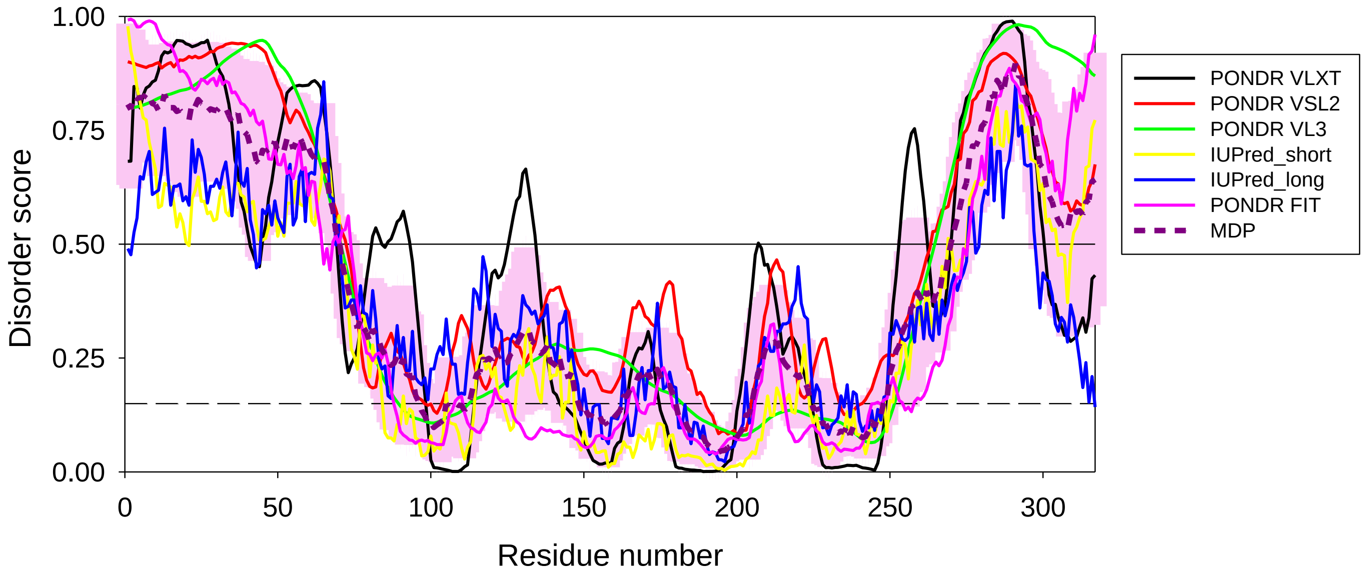

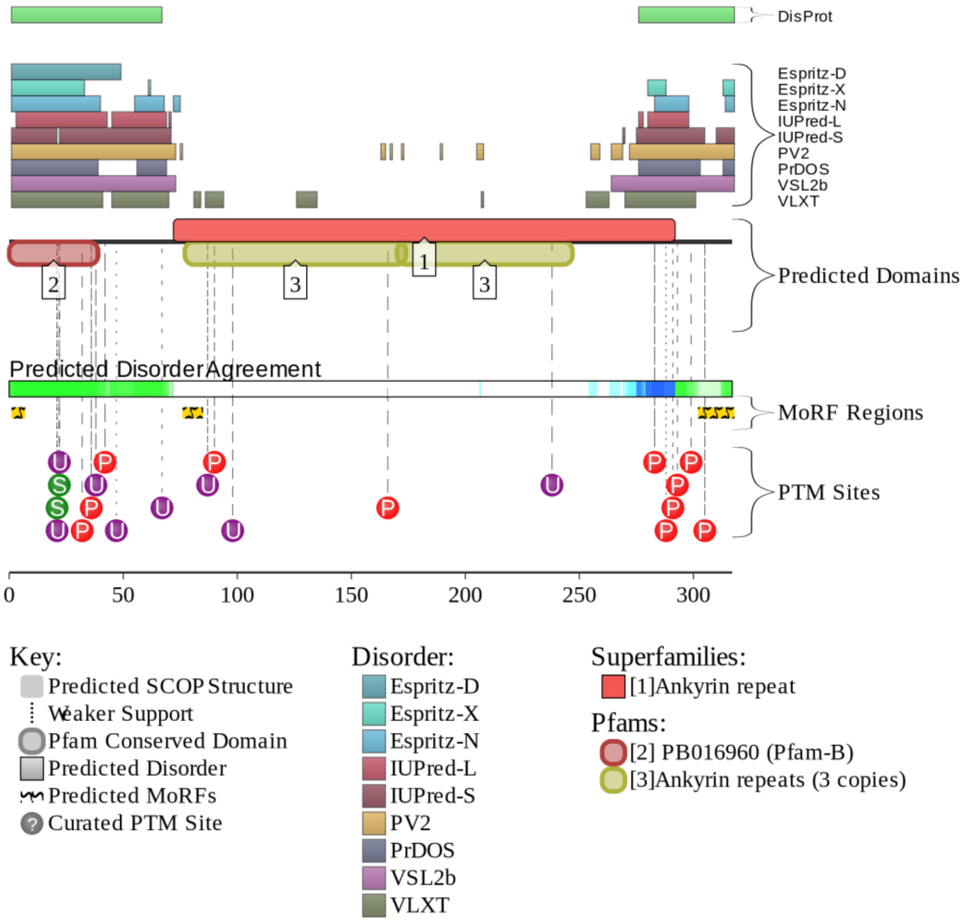

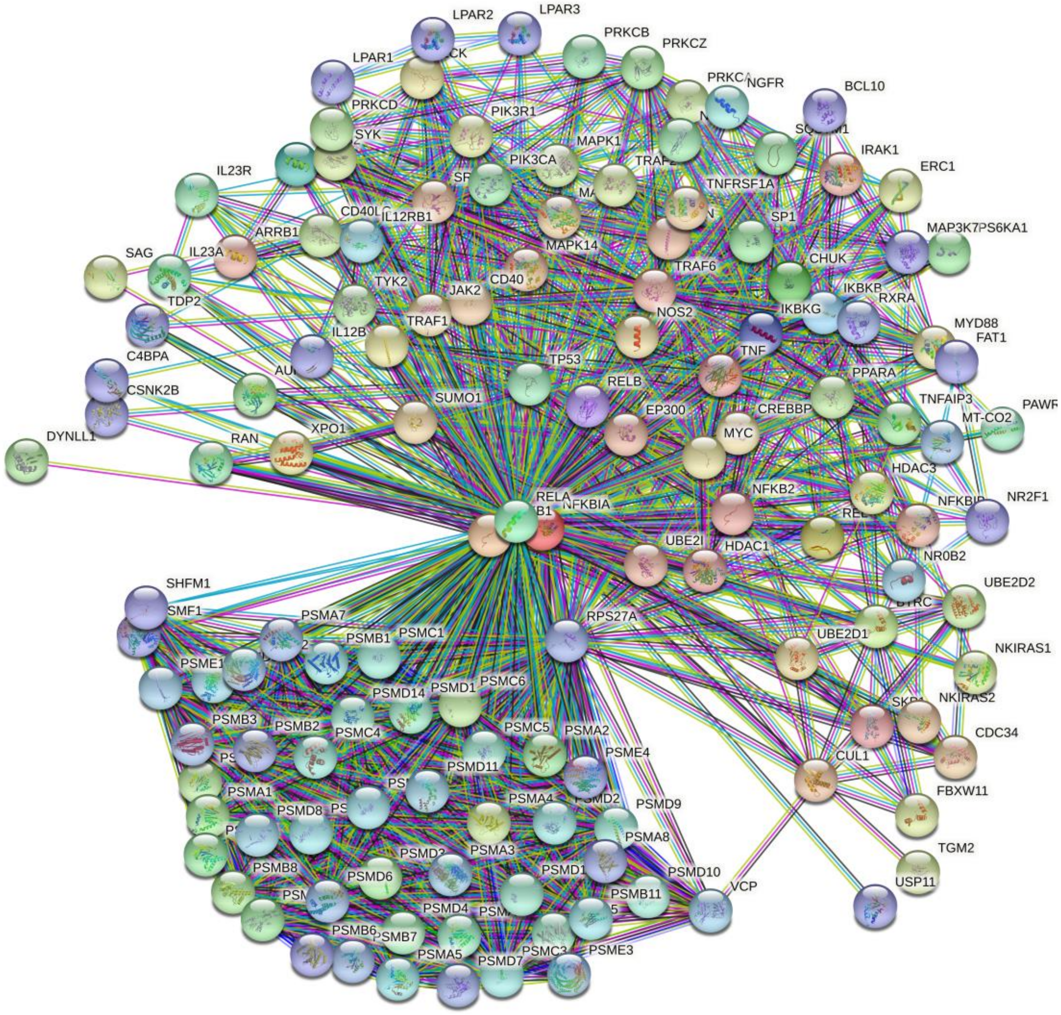
LPAR2
LPAR3
PRKCB
PRKCZ
LPAR1
PRKCD
PIK3R1
NGFR
BCL10
SYK
MAPK1
IRAK1
ERC1
IL23R
PIK3CA
TNFRSF1A
SP1
IL12RB1
MAP3K7
ARRB1
MAPK14
CHUK
SAG
IL23A
CD40
TRAF6
TYK2
JAK2
IKBKB
RXRA
TDP2
TRAF1
NOS2
IKBKG
MYD88
IL12B
FAT1
C4BPA
TNF
TP53
PPARA
CSNK2B
RELB
SUMO1
EP300
CREBBP
TNFAIP3
MT-CO2
DYNLL1
RAN
XPO1
MYC
HDAC3
RELA
NFKBIA
NFKB2
NR2F1
UBE2I
HDAC1
NR0B2
SHFM1
PSMA7
PSMB1
PSMC1
RPS27A
UBE2D2
UBE2D1
NKIRAS1
PSME1
PSMD14
PSMD1
PSMC6
NKIRAS2
PSMB3
PSMB2
PSMC4
PSMC5
PSMA2
CDC34
CUL1
PSME4
PSMD11
PSMA1
FBXW11
PSMA4
PSMD2
PSMD9
PSMD8
PSMA8
TGM2
PSMA3
USP11
PSMB8
PSMD6
PSMD10
VCP
PSMB11
PSMD4
PSMB6
PSMB7
PSMA5
PSMD7
PSMC3
PSME3

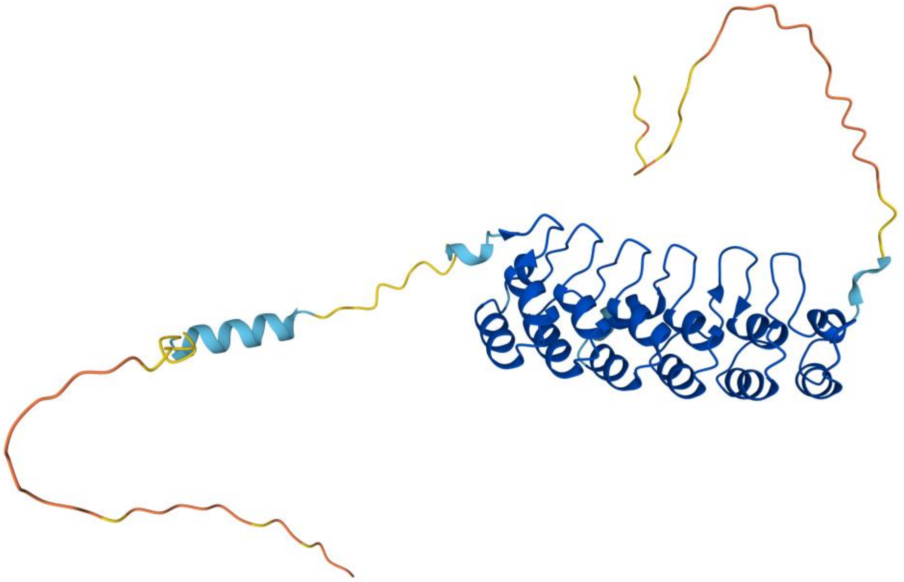

>sp|Q04206|TF65_HUMAN Transcription factor p65 OS=Homo sapiens OX=9606 GN=RELA PE=1 SV=2
MDELFPLIFPAEPAQASGPYVEIIEQPKQRGMRFRYKCEGRSAGSIPGERSTDTTKTHPTIKINGYTGPGTVRISLVTKDPPHRP
HPHELVGKDCRDGFYEAELCPDRCIHSFQNLGIQCVKKRDLEQAISQRIQTNNNPFQVPIEEQRGDYDLNAVRLCFQVTVRDPSG
RPLRLPPVLSHPIFDNRAPNTAELKICRVNRNSGSCLGGDEIFLLCDKVQKEDIEVYFTGPGWEARGSFSQADVHRQVAIVFRTP
PYADPSLQAPVRVSMQLRRPSDRELSEPMEFQYLPDTDDRHRIEEKRKRTYETFKSIMKKSPFSGPTDPRPPPRRIAVPSRSSAS
VPKPAPQPYPFTSSLSTINYDEFPTMVFPSGQISQASALAPAPPQVLPQAPAPAPAPAMVSALAQAPAPVPVLAPGPPQAVAPPA
PKPTQAGEGTLSEALLQLQFDDEDLGALLGNSTDPAVFTDLASVDNSEFQQLLNQGIPVAPHTTEPMLMEYPEAITRLVTGAQRP
PDPAPAPLGAPGLPNGLLSGDEDFSSIADMDFSALLSQISS

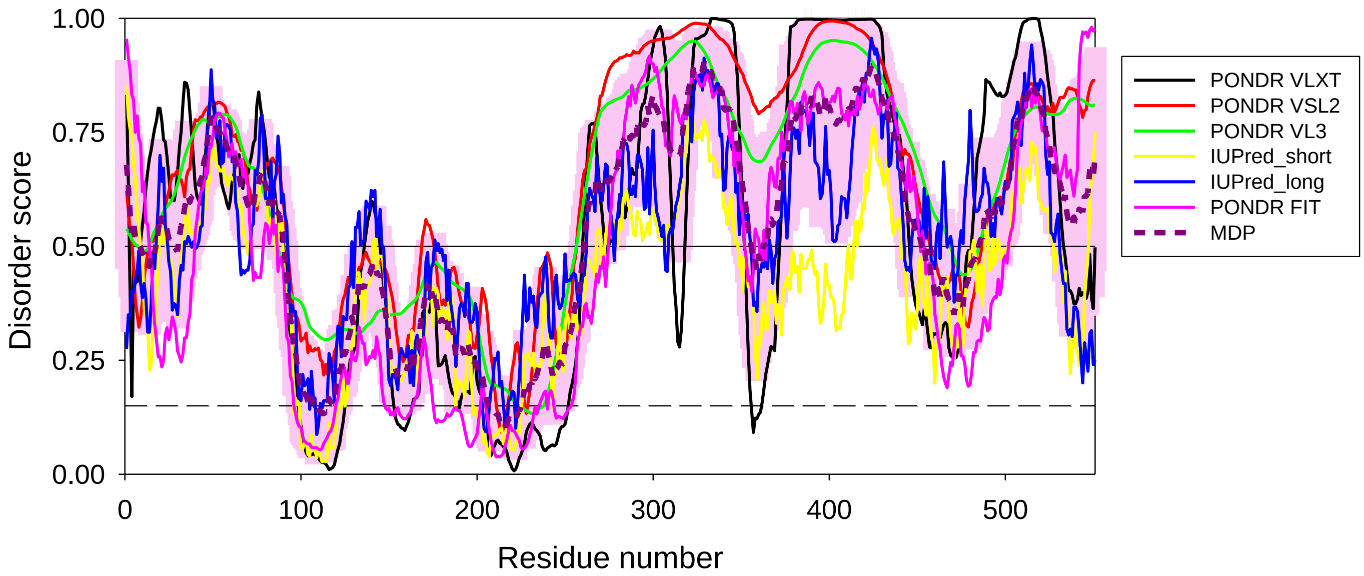

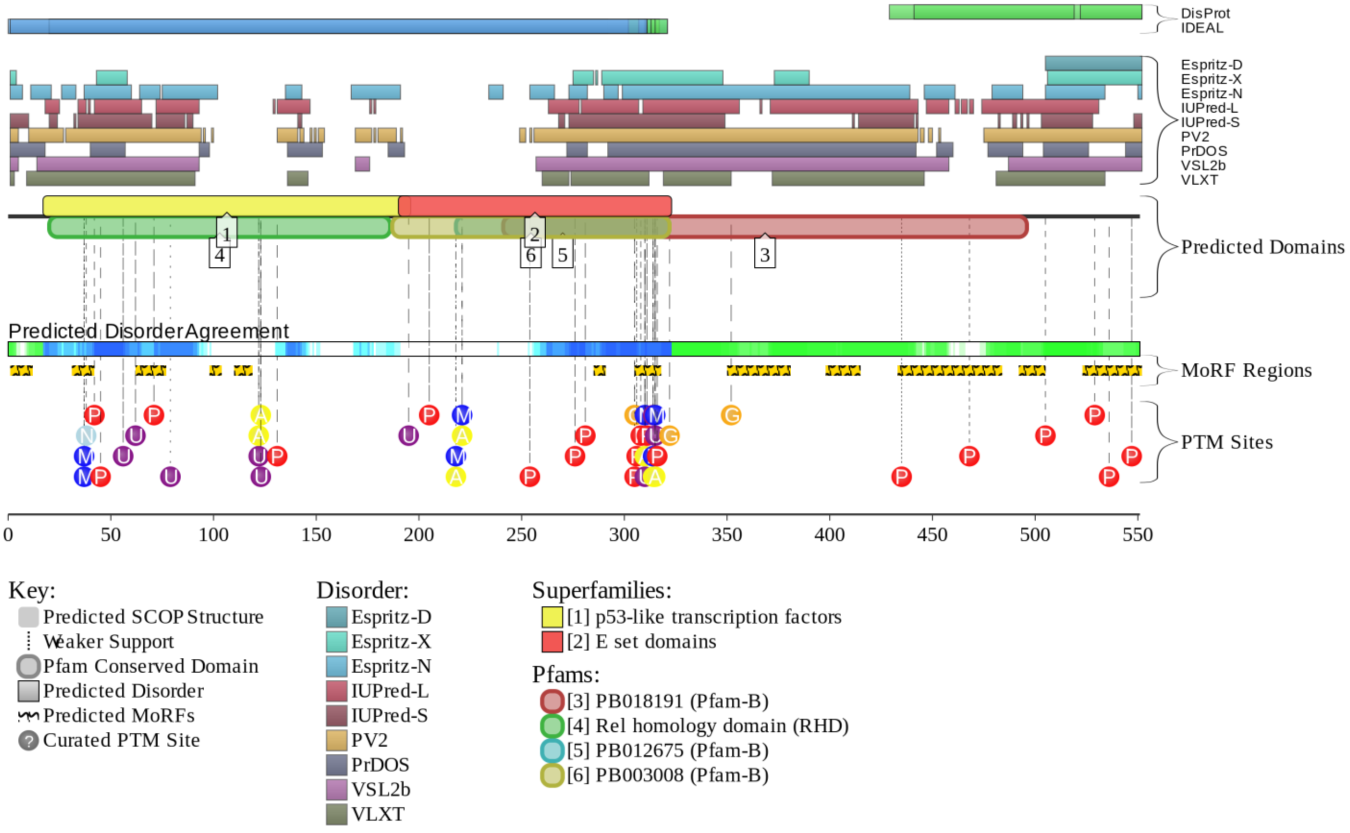

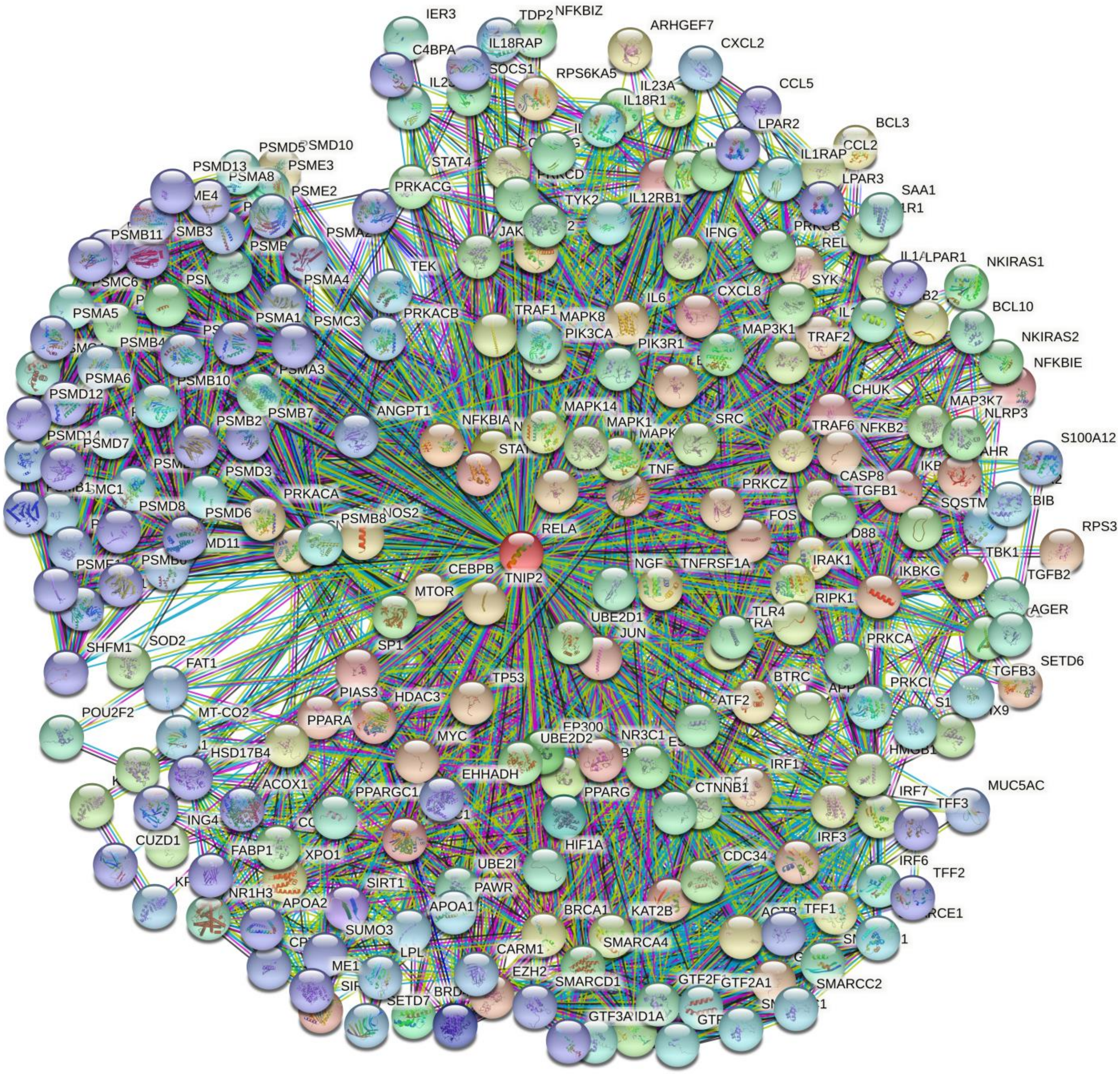
IER3
TDP2
NFKBIZ
ARHGEF7
C4BPA
IL18RAP
CXCL2
SOCS1
RPS6KA5
IL23A
IL18R1
CCL5
LPAR2
BCL3
PSMD10
CCL2
PSME3
PSMD13
PSMA8
STAT4
IL1RAP
PSME2
PRKACG
LPAR3
SAA1
TYK2
IL12RB1
PSMB11
IFNG
PSMA4
TEK
LPAR1
NKIRAS1
PSMC6
MAPK8
IL6
CXCL8
BCL10
PSMA5
TRAF1
PSMA1
PSMC3
PRKACB
PIK3CA
PIK3R1
MAP3K1
TRAF2
NKIRAS2
PSMB4
NFKBIE
PSMA3
PSMA6
PSMB10
CHUK
PSMD12
MAPK14
MAP3K7
ANGPT1
NFKBIA
MAPK1
SRC
NLRP3
PSMB2
PSMB7
TRAF6
NFKB2
S100A12
PSMD7
AHR
TNF
PSMD3
CASP8
PRKCZ
PRKACA
TGFB1
PSMD8
SQSTM
PSMD6
PSMB8
NOS2
FOS
RPS3
RELA
TBK1
NGF
TNFRSF1A
IRAK1
CEBPB
TNIP2
IKBKG
TGFB2
MTOR
RIPK1
AGER
UBE2D1
TLR4
JUN
PRKCA
SHFM1
SOD2
SP1
SETD6
FAT1
TGFB3
TP53
BTRC
PIAS3
POU2F2
HDAC3
ATF2
PRKCI
MT-CO2
PPARA
EP300
NR3C1
MYC
UBE2D2
HSD17B4
HMGB1
EHHADH
IRF1
ACOX1
CTNNB1
MUC5AC
PPARGC1
PPARG
IRF7
TFF3
ING4
HIF1A
IRF3
CUZD1
FABP1
XPO1
CDC34
UBE2I
IRF6
TFF2
SIRT1
NR1H3
PAWR
APOA2
APOA1
BRCA1
KAT2B
TFF1
SUMO3
SMARCA4
LPL
CARM1
ME1
EZH2
SMARCD1
GTF2F
GTF2A1
SMARCC2
SETD7
GTF3A

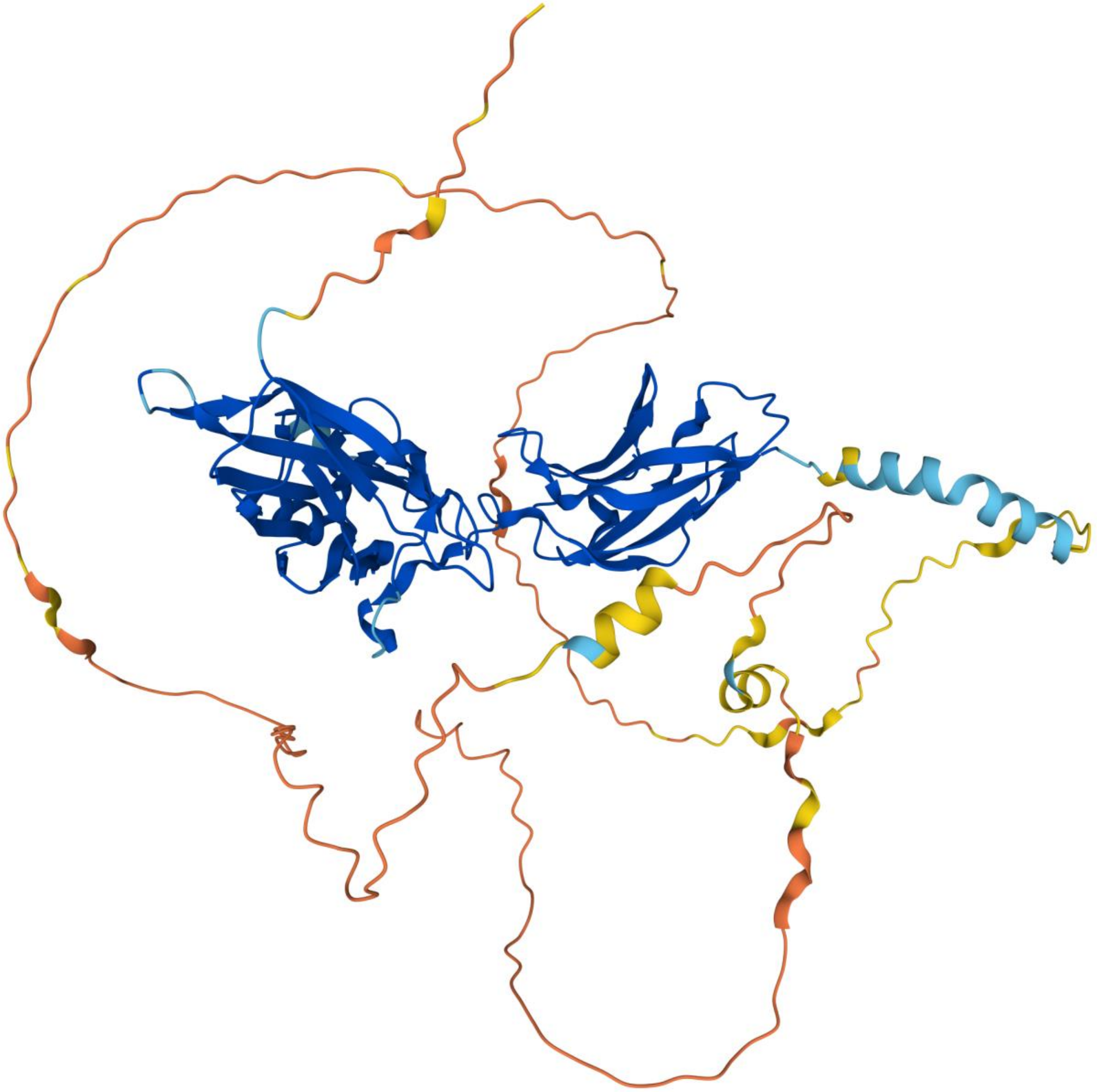

```
>sp|P19838|NFKB1_HUMAN Nuclear factor NF-kappa-B p105 subunit OS=Homo sapiens OX=9606
GN=NFKB1 PE=1 SV=2
MAEDDPYLGRPEQMFHLDPSLTHTIFNPEVFQPQMALPTDGPYLQILEQPKQRGFRFRYVCEGPSHGGLPGASSEKNKKSYPQVK
ICNYVGPAKVIVQLVTNGKNIHLHAHSLVGKHCEDGICTVTAGPKDMVVGFANLGILHVTKKKVFETLEARMTEACIRGYNPGLL
VHPDLAYLQAEGGGDRQLGDREKELIRQAALQQTKEMDLSVVRLMFTAFLPDSTGSFTRRLEPVVSDAIYDSKAPNASNLKIVRM
DRTAGCVTGGEEIYLLCDKVQKDDIQIRFYEEEENGGVWEGFGDFSPTDVHRQFAIVFKTPKYKDINITKPASVFVQLRRKSDLE
TSEPKPFLYYPEIKDKEEVQRKRQKLMPNFSDSFGGGSGAGAGGGGMFGSGGGGGGTGSTGPGYSFPHYGFPTYGGITFHPGTTK
SNAGMKHGTMDTESKKDPEGCDKSDDKNTVNLFGKVIETTEQDQEPSEATVGNGEVTLTYATGTKEESAGVQDNLFLEKAMQLAK
RHANALFDYAVTGDVKMLLAVQRHLTAVQDENGDSVLHLAIIHLHSQLVRDLLEVTSGLISDDIINMRNDLYQTPLHLAVITKQE
DVVEDLLRAGADLSLLDRLGNSVLHLAAKEGHDKVLSILLKHKKAALLLDHPNGDGLNAIHLAMMSNSLPCLLLLVAAGADVNAQ
EQKSGRTALHLAVEHDNISLAGCLLLEGDAHVDSTTYDGTTPLHIAAGRGSTRLAALLKAAGADPLVENFEPLYDLDDSWENAGE
DEGVVPGTTPLDMATSWQVFDILNGKPYEPEFTSDDLLAQGDMKQLAEDVKLQLYKLLEIPDPDKNWATLAQKLGLGILNNAFRL
SPAPSKTLMDNYEVSGGTVRELVEALRQMGYTEAIEVIQAASSPVKTTSQAHSLPLSPASTRQQIDELRDSDSVCDSGVETSFRK
LSFTESLTSGASLLTLNKMPHDYGQEGPLEGKI
```

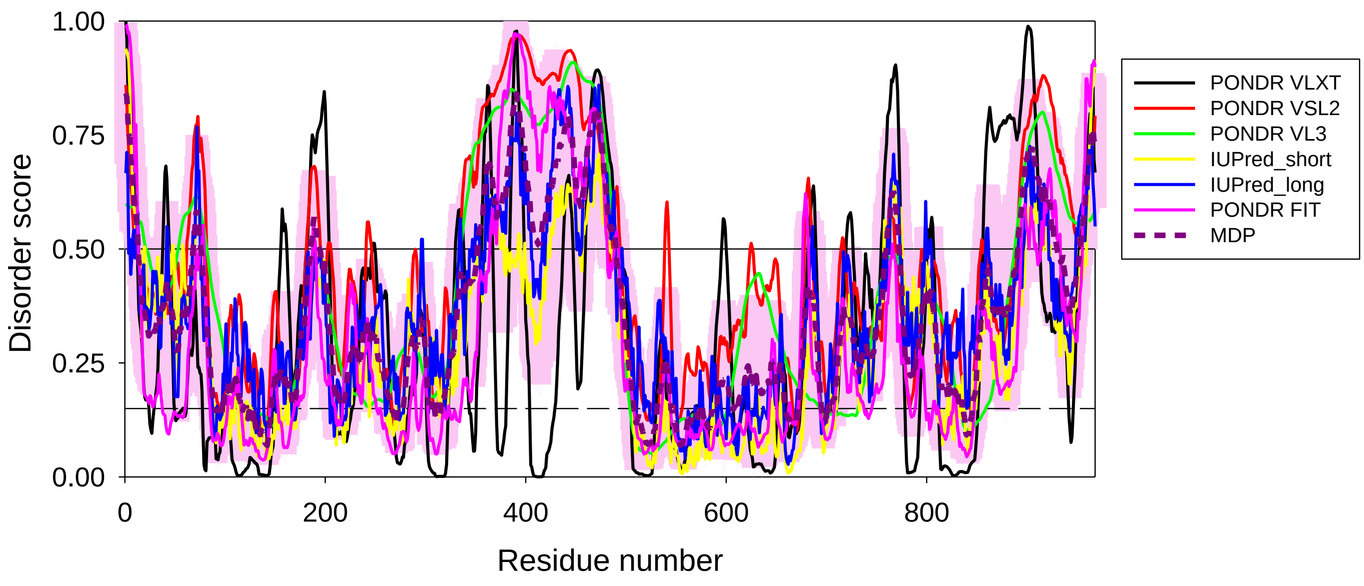

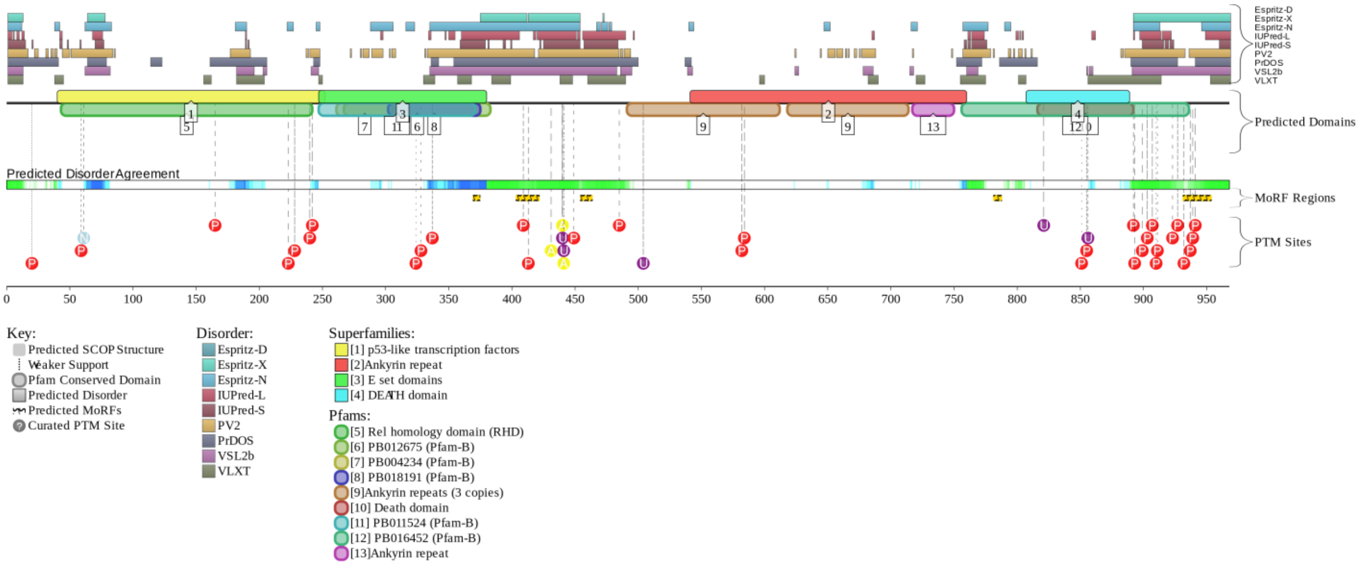

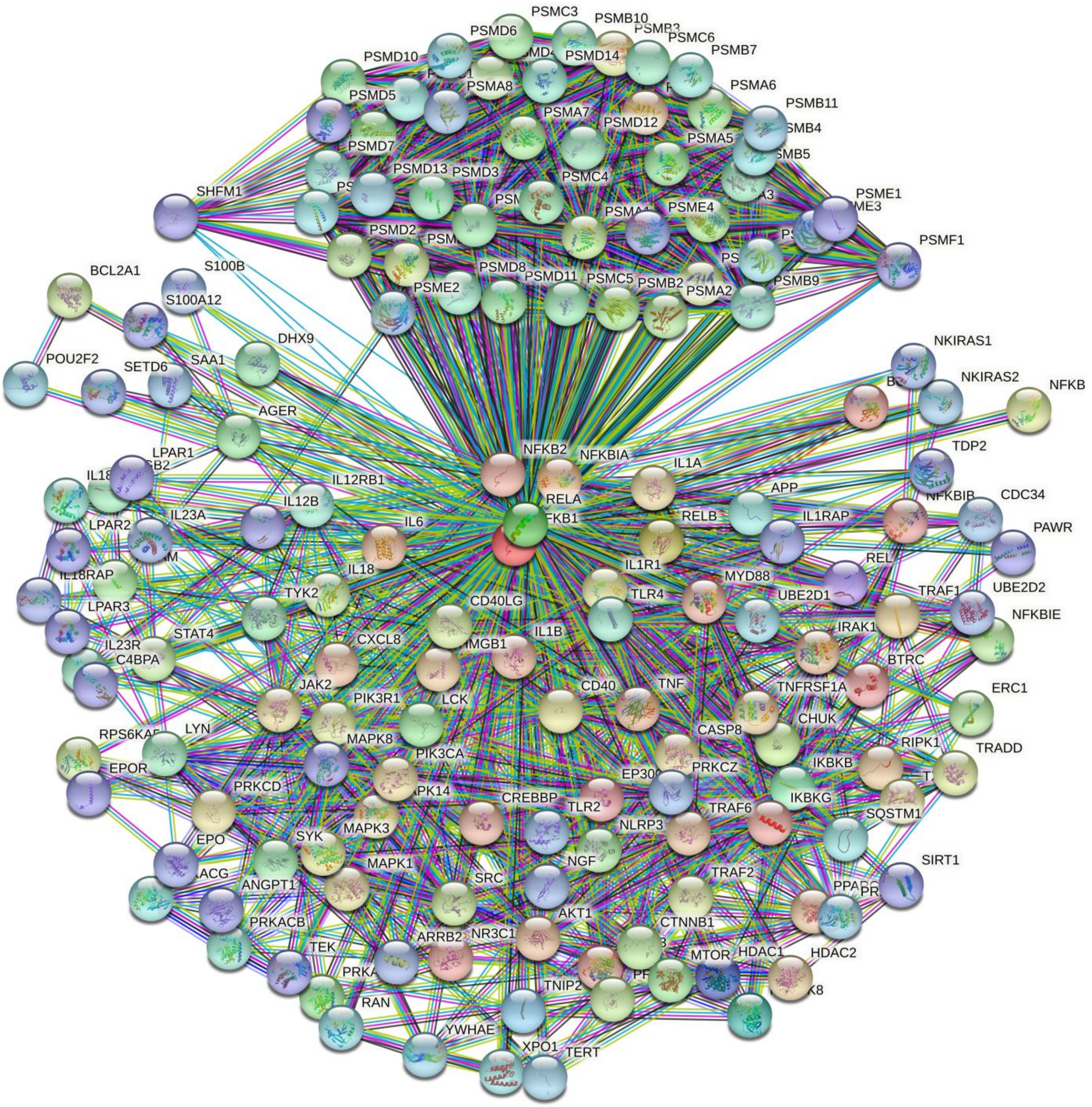
PSMC3
PSMB10
PSMB3
PSMD6
PSMC6
PSMB7
PSMD10
PSMD14
PSMA8
PSMA6
PSMD5
PSMB11
PSMB4
PSMA7
PSMD12
PSMA5
PSMB5
PSMD7
PSMD13
PSMD3
PSMC4
SHFM1
PSME1
PSME3
PSMD2
PSME4
PSMF1
BCL2A1
S100B
PSMD8
PSMD11
PSMC5
PSMB2
PSMA2
PSMB9
S100A12
PSME2
DHX9
NKIRAS1
POU2F2
SETD6
SAA1
NKIRAS2
NFKB
AGER
LPAR1
NFKB2
NFKBIA
TDP2
IL12RB1
IL1A
APP
RELA
NFKBIB
CDC34
IL23A
IL12B
RELB
IL1RAP
PAWR
LPAR2
IL6
REL
IL18RAP
IL18
IL1R1
MYD88
UBE2D2
TRAF1
LPAR3
TYK2
CD40LG
TLR4
UBE2D1
NFKBIE
STAT4
IL23R
CXCL8
IL1B
IRAK1
C4BPA
BTRC
JAK2
PIK3R1
LCK
CD40
TNF
TNFRSF1A
ERC1
CHUK
CASP8
LYN
MAPK8
RIPK1
TRADD
PIK3CA
IKBKB
EPOR
PRKCZ
PRKCD
CREBBP
TLR2
IKBKG
TRAF6
SQSTM1
MAPK3
NLRP3
SYK
EPO
SIRT1
MAPK1
NGF
TRAF2
ANGPT1
SRC
PRKACB
AKT1
CTNNB1
NR3C1
TEK
ARRB2
MTOR
HDAC1
HDAC2
TNIP2
RAN
YWHAE
XPO1
TERT

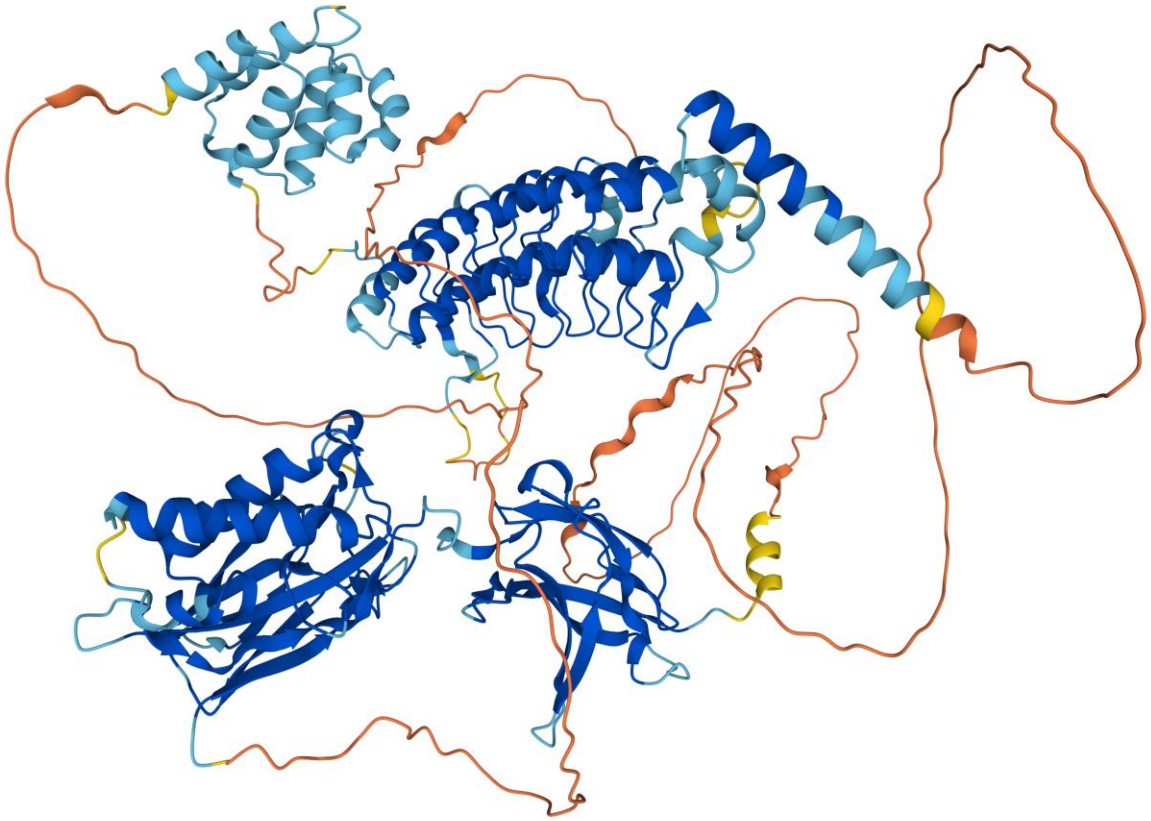

>sp|Q00653|NFKB2_HUMAN Nuclear factor NF-kappa-B p100 subunit OS=Homo sapiens OX=9606 GN=NFKB2 PE=1 SV=4
MESCYNPGLDGIIEYDDFKLNSSIVEPKEPAPETADGPYLVIVEQPKQRGFRFRYGCEGPSHGGLPGASSEKGRKTYPTVKICNY
EGPAKIEVDLVTHSDPPRAHAHSLVGKQCSELGICAVSVGPKDMTAQFNNLGVLHVTKKNMMGTMIQKLQRQRLRSRPQGLTEAE
QRELEQEAKELKKVMDLSIVRLRFSAFLRASDGSFSLPLKPVISQPIHDSKSPGASNLKISRMDKTAGSVRGGDEVYLLCDKVQK
DDIEVRFYEDDENGWQAFGDFSPTDVHKQYAIVFRTPPYHKMKIERPVTVFLQLKRKRGGDVSDSKQFTYYPLVEDKEEVQRKRR
KALPTFSQPFGGGSHMGGGSGGAAGGYGGAGGGGSLGFFPSSLAYSPYQSGAGPMGCYPGGGGGAQMAATVPSRDSGEEAAEPSA
PSRTPQCEPQAPEMLQRAREYNARLFGLAQRSARALLDYGVTADARALLAGQRHLLTAQDENGDTPLHLAIIHGQTSVIEQIVYV
IHHAQDLGVVNLTNHLHQTPLHLAVITGQTSVVSFLLRVGADPALLDRHGDSAMHLALRAGAGAPELLRALLQSGAPAVPQLLHM
PDFEGLYPVHLAVRARSPECLDLLVDSGAEVEATERQGGRTALHLATEMEELGLVTHLVTKLRANVNARTFAGNTPLHLAAGLGY
PTLTRLLLKAGADIHAENEEPLCPLPSPPTSDSDSDSEGPEKDTRSSFRGHTPLDLTCSTKVKTLLLNAAQNTMEPPLTPPSPAG
PGLSLGDTALQNLEQLLDGPEAQGSWAELAERLGLRSLVDTYRQTTSPSGSLLRSYELAGGDLAGLLEALSDMGLEEGVRLLRGP
ETRDKLPSTAEVKEDSAYGSQSVEQEAEKLGPPPEPPGGLCHGHPQPQVH

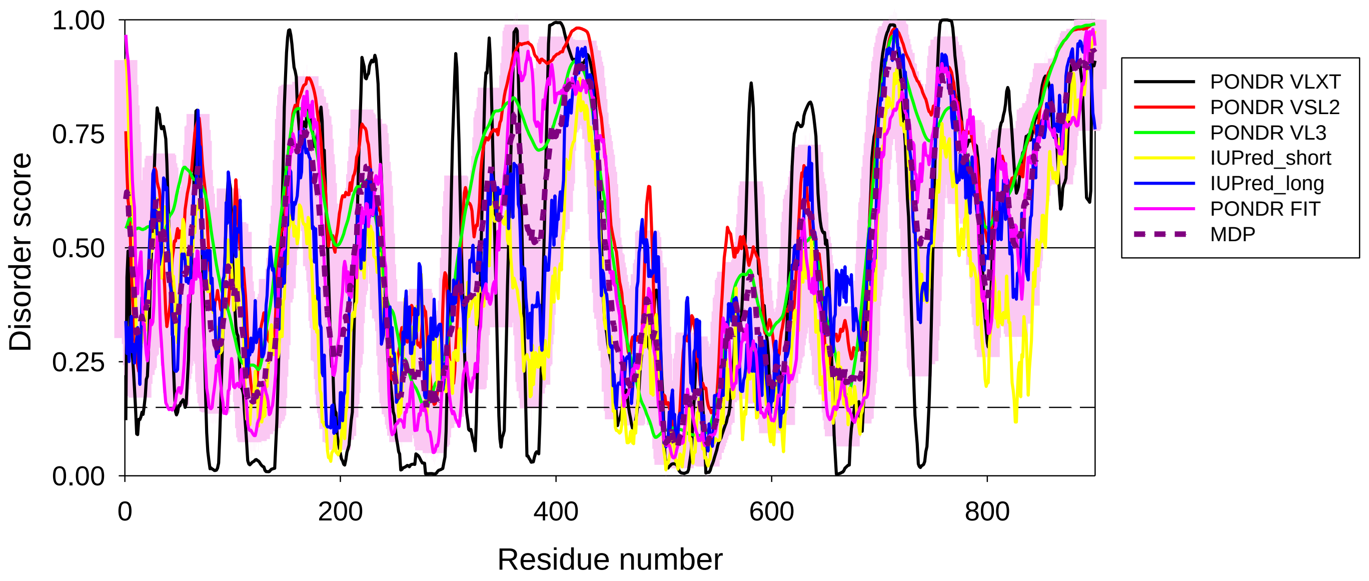

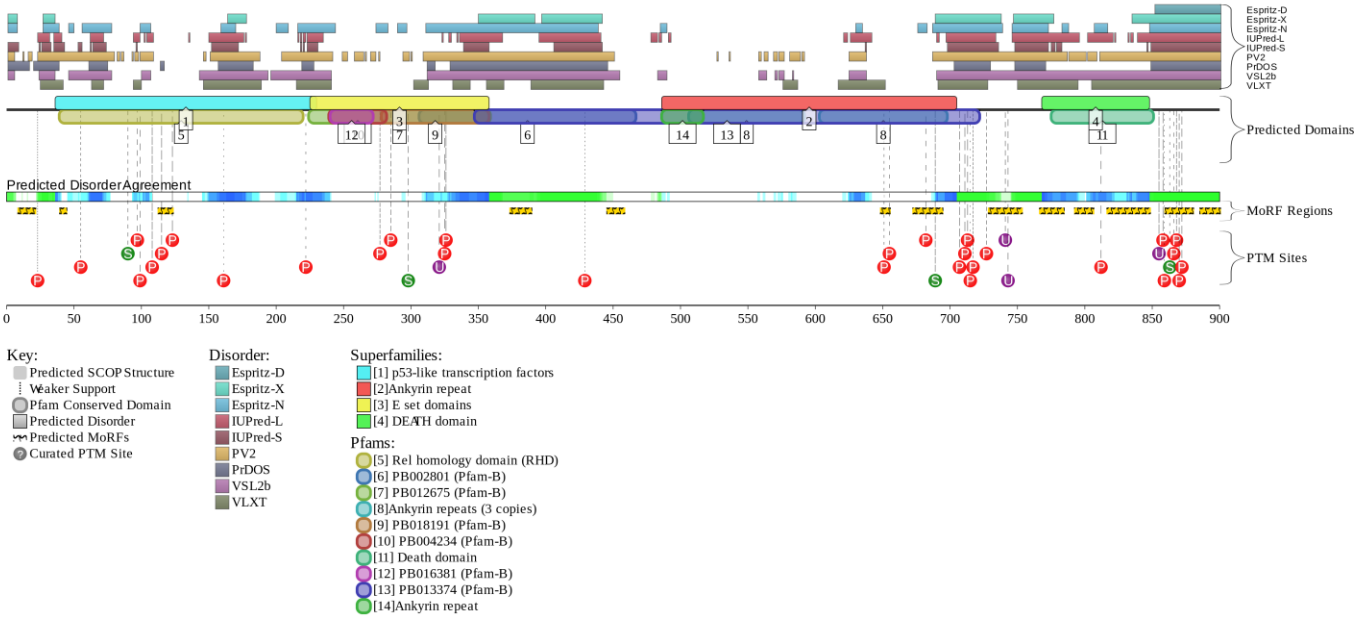

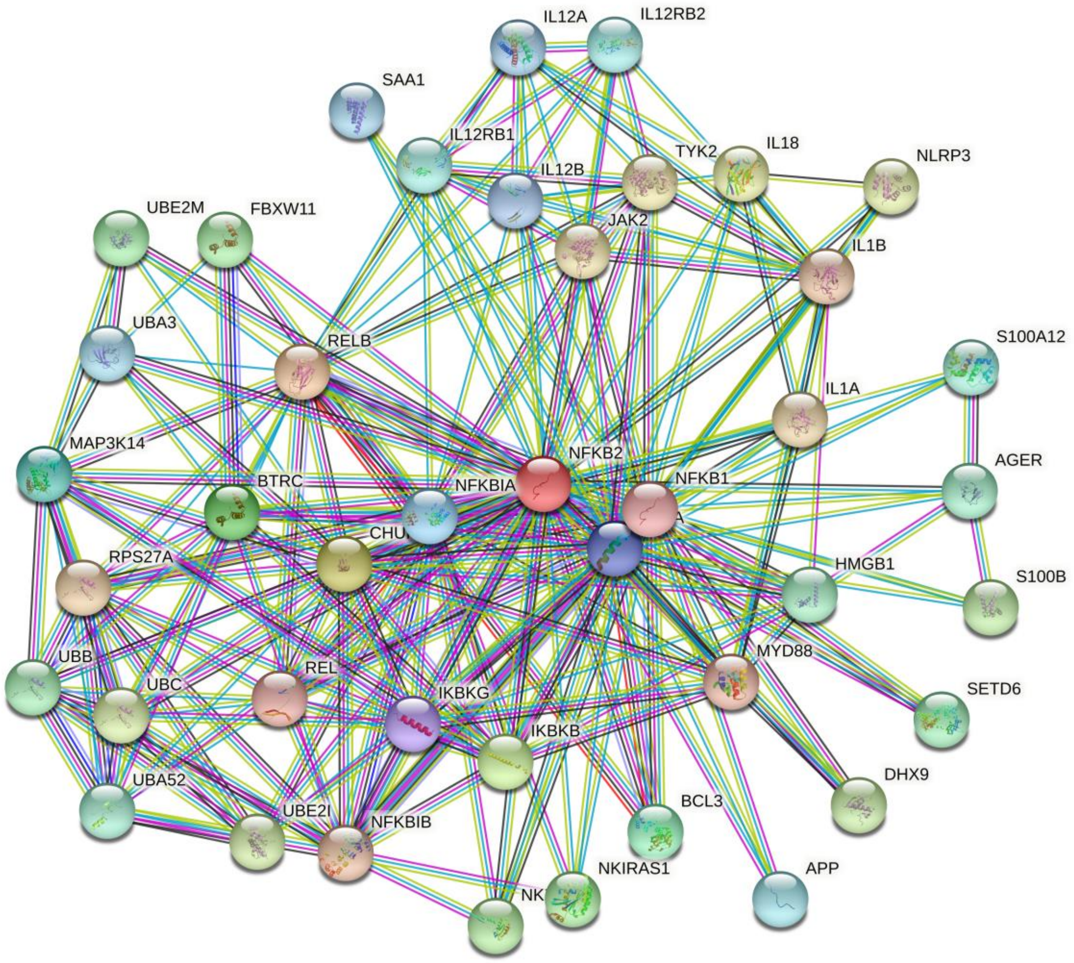
IL12A
IL12RB2
SAA1
IL12RB1
TYK2
IL18
NLRP3
IL12B
UBE2M
FBXW11
JAK2
IL1B
UBA3
RELB
S100A12
IL1A
MAP3K14
NFKB2
AGER
BTRC
NFKBIA
NFKB1
CHU
RPS27A
HMGB1
S100B
MYD88
UBB
REL
SETD6
UBC
IKBKG
IKBKB
UBA52
DHX9
BCL3
UBE2I
NFKBIB
NKIRAS1
APP

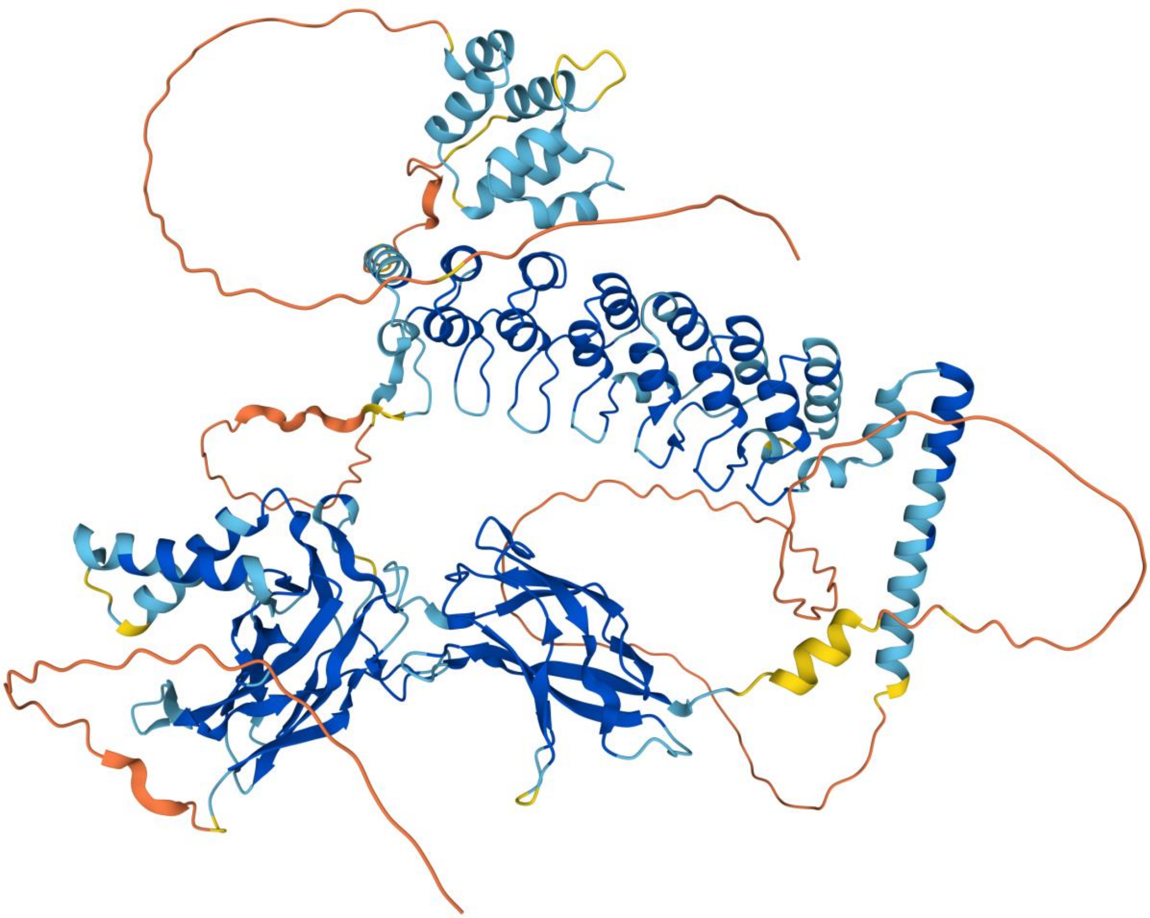

>sp|Q14653|IRF3_HUMAN Interferon regulatory factor 3 OS=Homo sapiens OX=9606 GN=IRF3 PE=1 SV=1
MGTPKPRILPWLVSQLDLGQLEGVAWVNKSRTRFRIPWKHGLRQDAQQEDFGIFQAWAEATGAYVPGRDKPDLPTWKRNFRSALN
RKEGLRLAEDRSKDPHDPHKIYEFVNSGVGDFSQPDTSPDTNGGGSTSDTQEDILDELLGNMVLAPLPDPGPPSLAVAPEPCPQP
LRSPSLDNPTPFPNLGPSENPLKRLLVPGEEWEFEVTAFYRGRQVFQQTISCPEGLRLVGSEVGDRTLPGWPVTLPDPGMSLTDR
GVMSYVRHVLSCLGGGLALWRAGQWLWAQRLGHCHTYWAVSEELLPNSGHGPDGEVPKDKEGGVFDLGPFIVDLITFTEGSGRSP
RYALWFCVGESWPQDQPWTKRLVMVKVVPTCLRALVEMARVGGASSLENTVDLHISNSHPLSLTSDQYKAYLQDLVEGMDFQGPG
ES

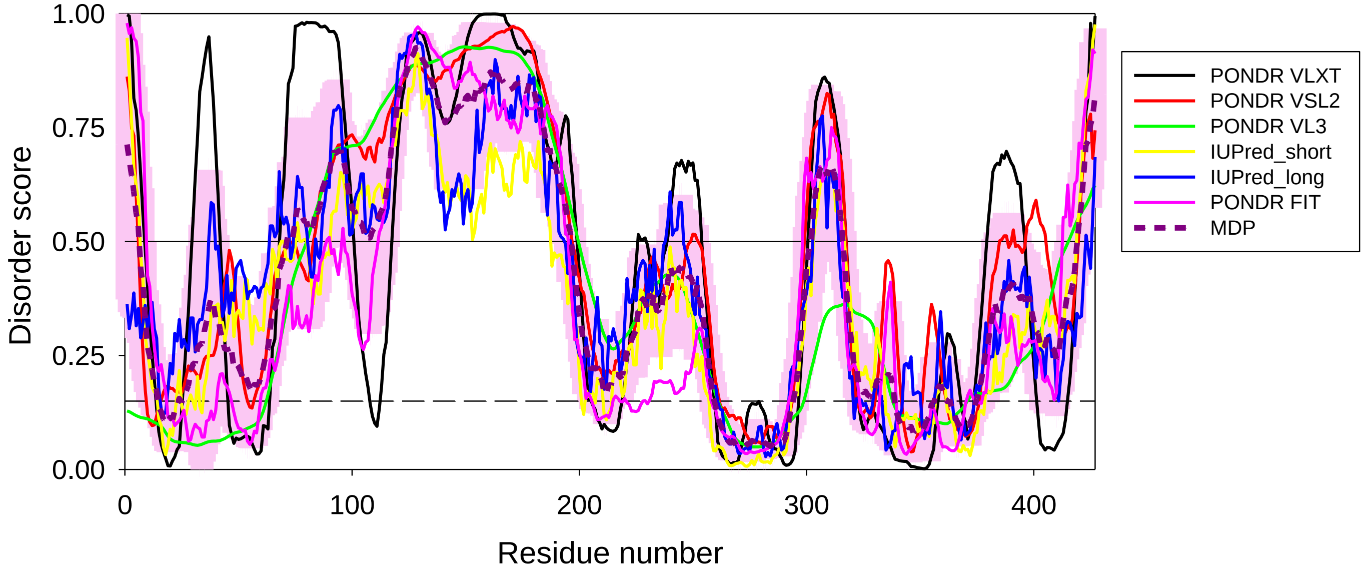

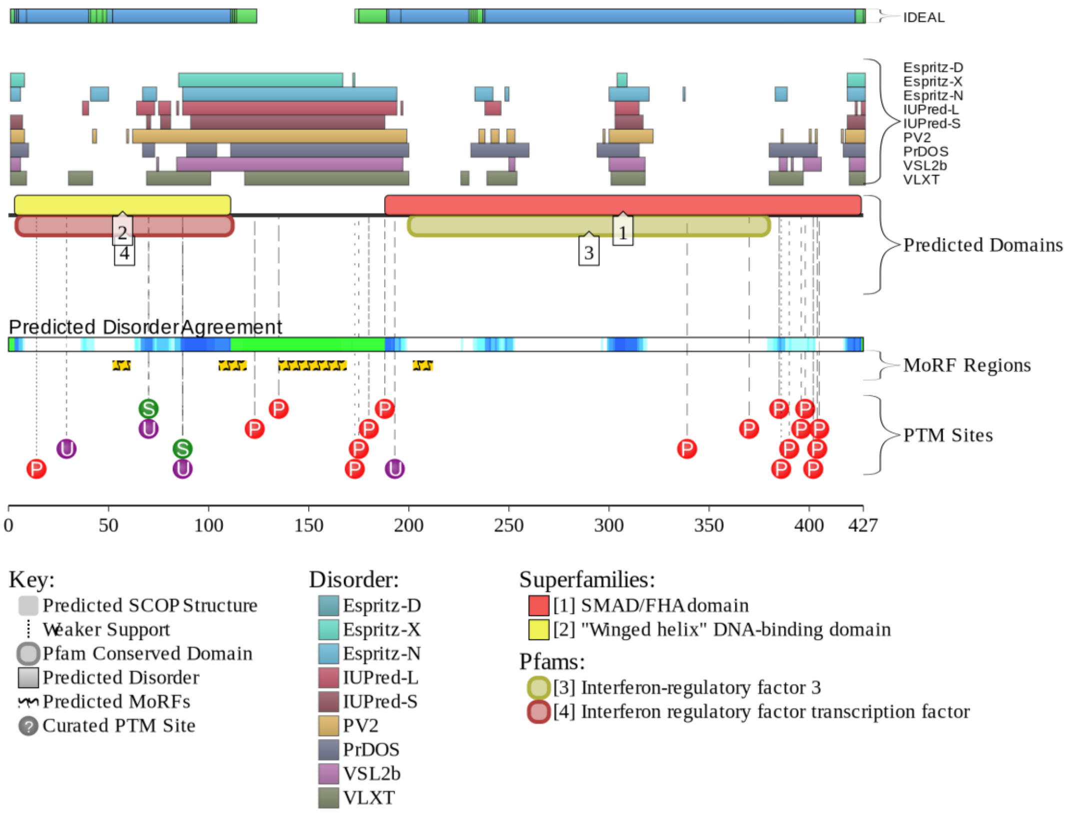

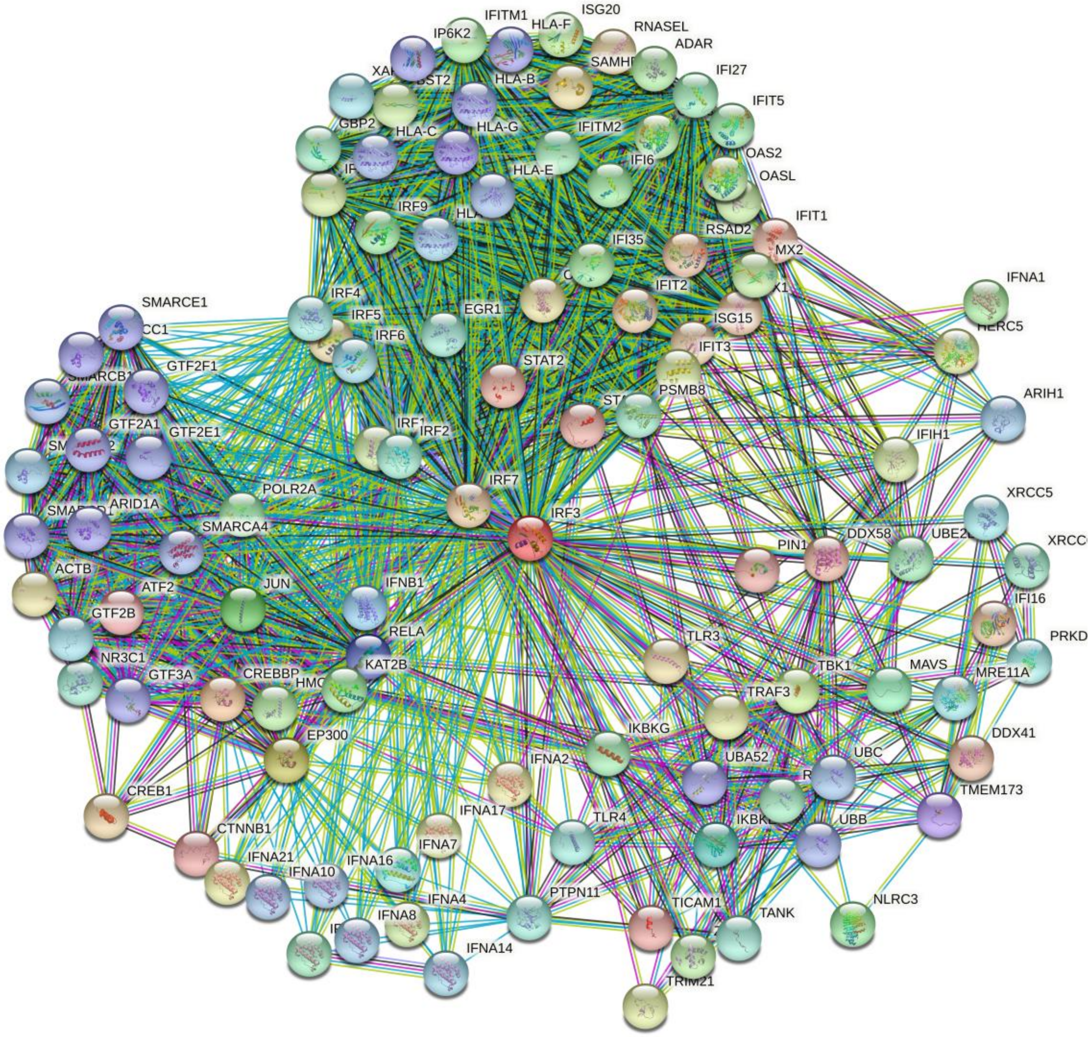
IFITM1
ISG20
HLA-F
RNASEL
IP6K2
ADAR
HLA-B
IFI27
IFIT5
GBP2
HLA-C
HLA-G
IFITM2
OAS2
IFI6
OASL
HLA-E
IRF9
RSAD2
IFIT1
IFI35
MX2
IFNA1
IFIT2
SMARCE1
IRF4
IRF5
EGR1
ISG15
IRF6
GTF2F1
STAT2
IFIT3
PSMB8
ARIH1
GTF2A1
GTF2E1
IRF2
IFIH1
POLR2A
IRF7
XRCC5
IRF3
ARID1A
SMARCA4
PIN1
DDX58
ACTB
ATF2
JUN
IFNB1
IFI16
GTF2B
RELA
NR3C1
KAT2B
TLR3
GTF3A
CREBBP
TBK1
MAVS
MRE11A
TRAF3
EP300
IKBKG
DDX41
IFNA2
UBA52
UBC
CREB1
TMEM173
IFNA17
TLR4
CTNNB1
UBB
IFNA21
IFNA7
IFNA16
IFNA10
PTPN11
NLRC3
IFNA4
TICAM1
TANK
IFNA8
IFNA14
TRIM21

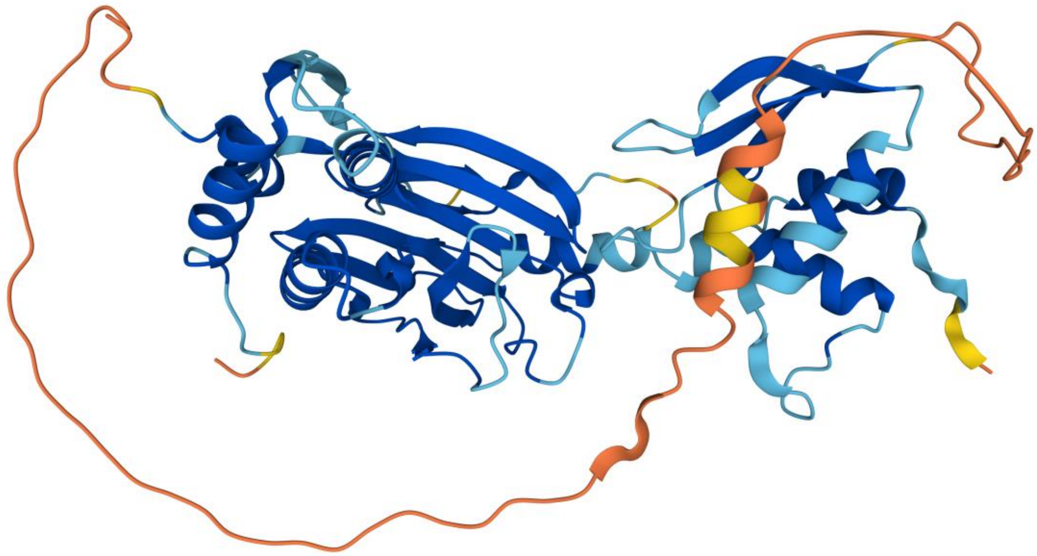

>sp|Q13568|IRF5_HUMAN Interferon regulatory factor 5 OS=Homo sapiens OX=9606 GN=IRF5 PE=1 SV=2
MNQSIPVAPTPPRRVRLKPWLVAQVNSCQYPGLQWVNGEKKLFCIPWRHATRHGPSQDGDNTIFKAWAKETGKYTEGVDEADPAK
WKANLRCALNKSRDFRLIYDGPRDMPPQPYKIYEVCSNGPAPTDSQPPEDYSFGAGEEEEEEEELQRMLPSLSLTEDVKWPPTLQ
PPTLRPPTLQPPTLQPPVVLGPPAPDPSPLAPPPGNPAGFRELLSEVLEPGPLPASLPPAGEQLLPDLLISPHMLPLTDLEIKFQ
YRGRPPRALTISNPHGCRLFYSQLEATQEQVELFGPISLEQVRFPSPEDIPSDKQRFYTNQLLDVLDRGLILQLQGQDLYAIRLC
QCKVFWSGPCASAHDSCPNPIQREVKTKLFSLEHFLNELILFQKGQTNTPPPFEIFFCFGEEWPDRKPREKKLITVQVVPVAARL
LLEMFSGELSWSADSIRLQISNPDLKDRMVEQFKELHHIWQSQQRLQPVAQAPPGAGLGVGQGPWPMHPAGMQ

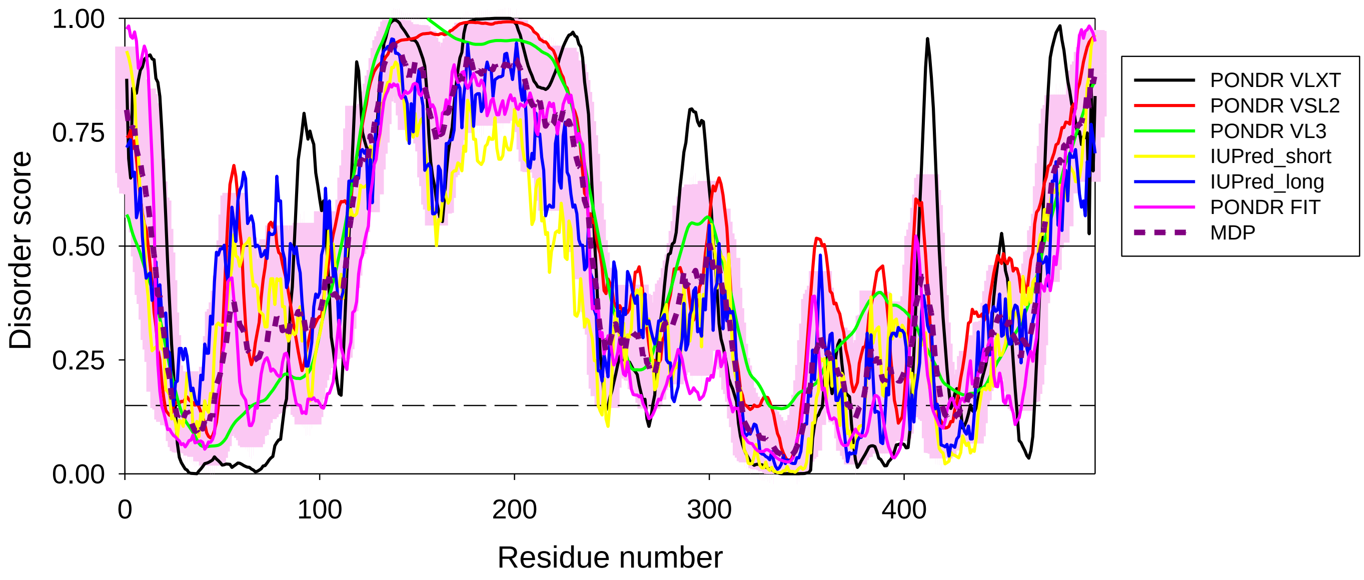

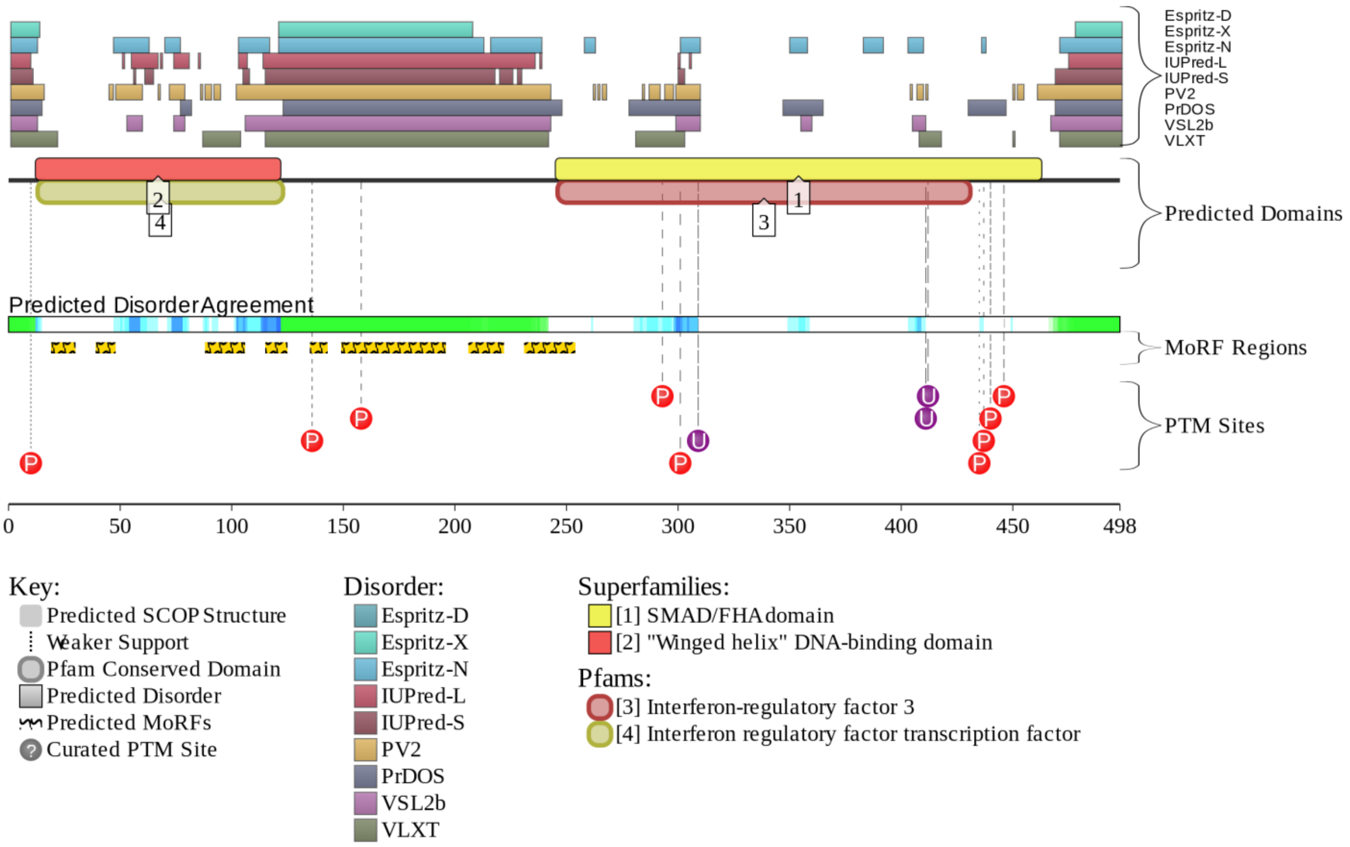

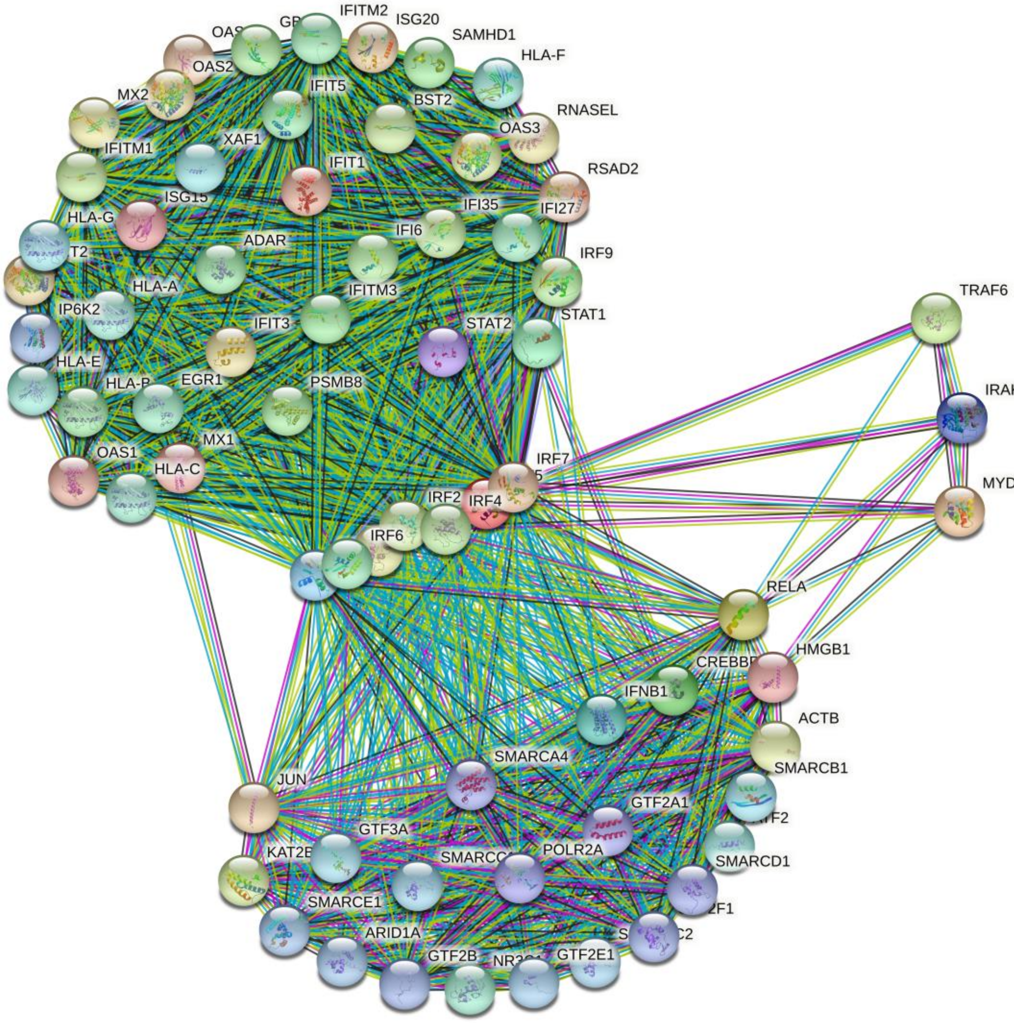
IFITM2
ISG20
SAMHD1
HLA-F
OAS2
MX2
IFIT5
BST2
RNASEL
OAS3
IFITM1
XAF1
IFIT1
RSAD2
ISG15
IFI35
IFI27
HLA-G
IFI6
ADAR
IRF9
HLA-A
IFITM3
IP6K2
IFIT3
STAT2
STAT1
TRAF6
HLA-E
HLA-B
EGR1
PSMB8
IRAK1
MX1
OAS1
HLA-C
IRF7
MYD88
IRF2
IRF4
IRF6
RELA
HMGB1
IFNB1
ACTB
SMARCA4
SMARCB1
JUN
GTF2A1
GTF3A
POLR2A
KAT2B
SMARCD1
SMARCE1
ARID1A
GTF2B
GTF2E1

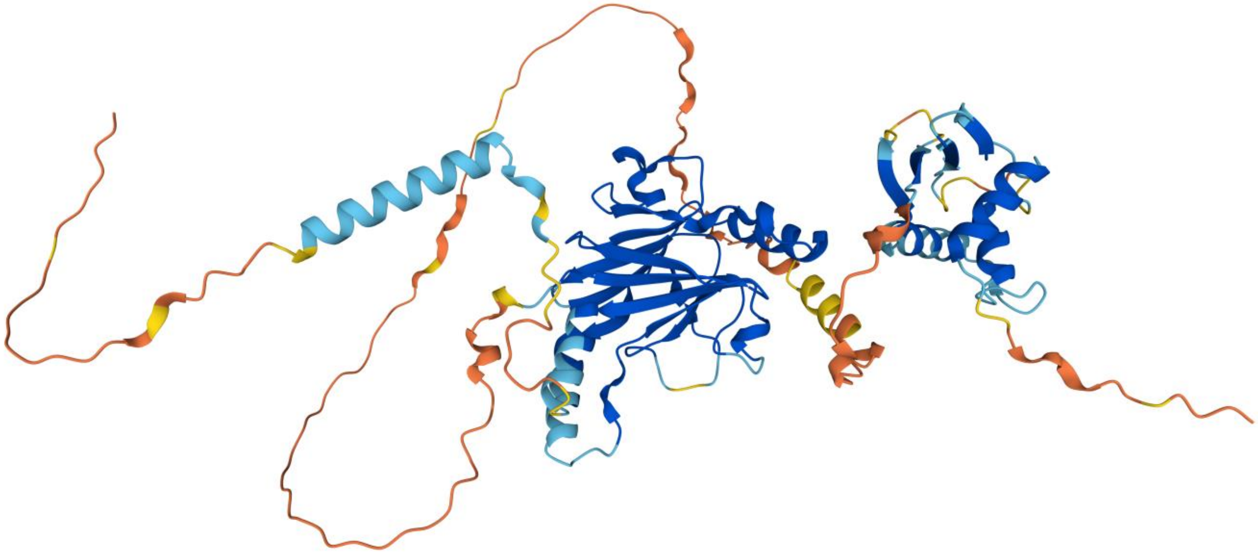

>sp|Q92985|IRF7_HUMAN Interferon regulatory factor 7 OS=Homo sapiens OX=9606 GN=IRF7 PE=1 SV=2
MALAPERAAPRVLFGEWLLGEISSGCYEGLQWLDEARTCFRVPWKHFARKDLSEADARIFKAWAVARGRWPPSSRGGGPPPEAET
AERAGWKTNFRCALRSTRRFVMLRDNSGDPADPHKVYALSRELCWREGPGTDQTEAEAPAAVPPPQGGPPGPFLAHTHAGLQAPG
PLPAPAGDKGDLLLQAVQQSCLADHLLTASWGADPVPTKAPGEGQEGLPLTGACAGGPGLPAGELYGWAVETTPSPGPQPAALTT
GEAAAPESPHQAEPYLSPSPSACTAVQEPSPGALDVTIMYKGRTVLQKVVGHPSCTFLYGPPDPAVRATDPQQVAFPSPAELPDQ
KQLRYTEELLRHVAPGLHLELRGPQLWARRMGKCKVYWEVGGPPGSASPSTPACLLPRNCDTPIFDFRVFFQELVEFRARQRRGS
PRYTIYLGFGQDLSAGRPKEKSLVLVKLEPWLCRVHLEGTQREGVSSLDSSSLSLCLSSANSLYDDIECFLMELEQPA

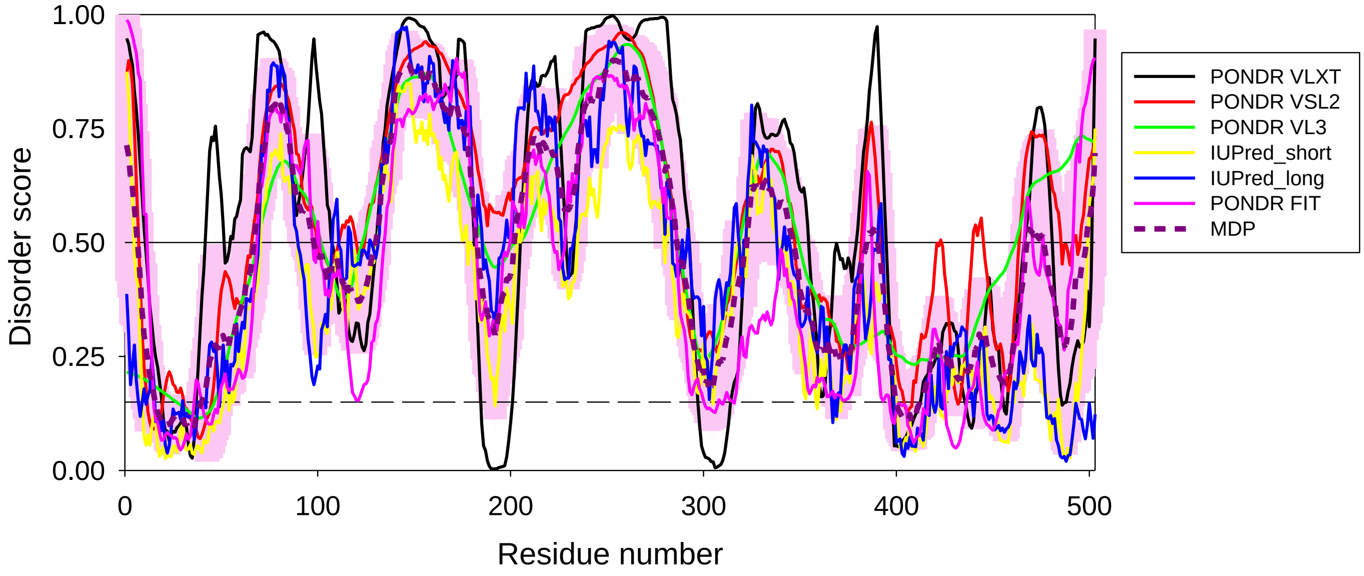

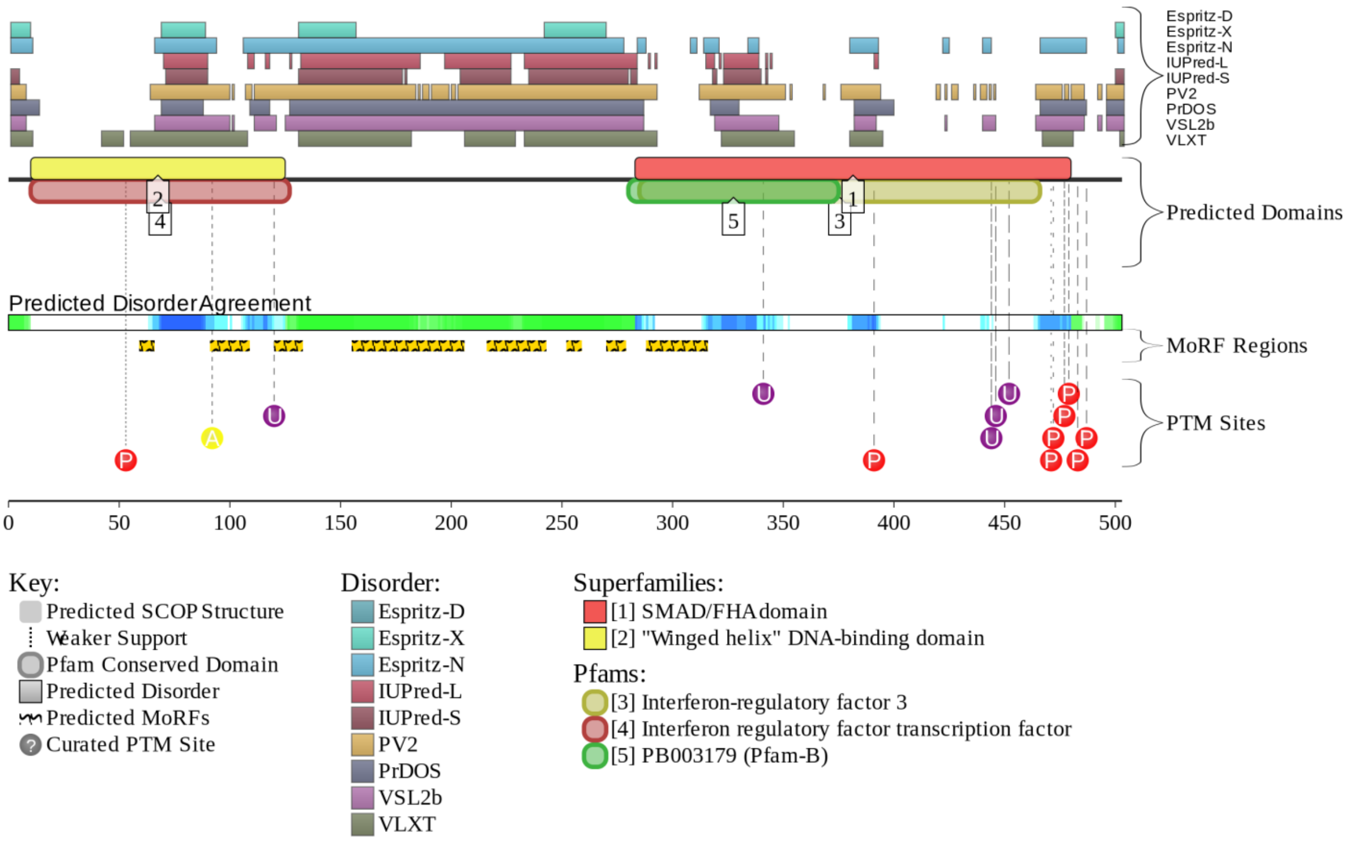

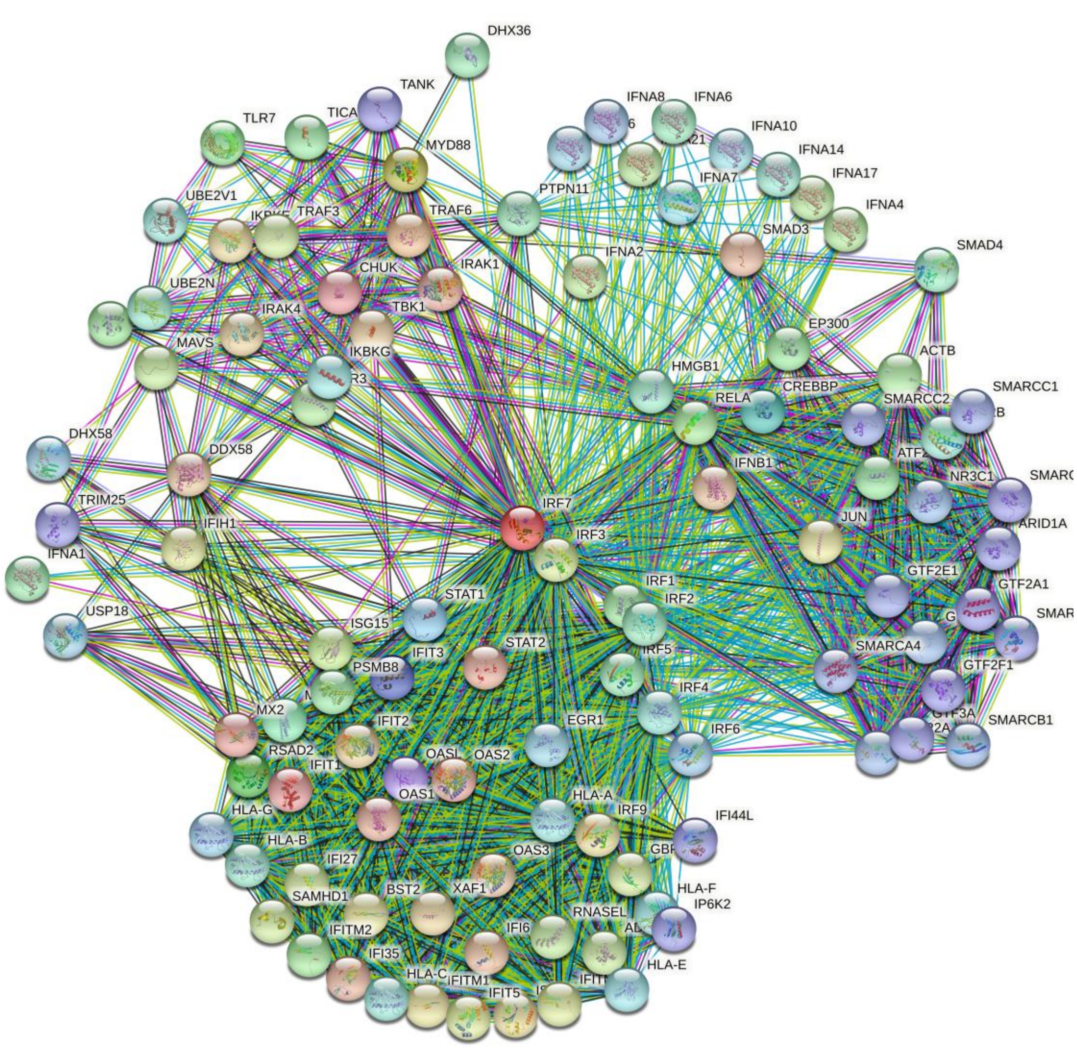
DHX36
TANK
TLR7
TICA
MYD88
IFNA8
IFNA6
IFNA10
IFNA14
IFNA17
IFNA4
PTPN11
IFNA7
UBE2V1
TRAF3
TRAF6
SMAD3
SMAD4
IFNA2
CHUK
IRAK1
UBE2N
IRAK4
TBK1
EP300
MAVS
IKBKG
HMGB1
ACTB
SMARCC1
RELA
CREBBP
SMARCC2
DHX58
DDX58
IFNB1
NR3C1
TRIM25
IRF7
IFIH1
JUN
ARID1A
IFNA1
IRF3
IRF1
GTF2E1
GTF2A1
USP18
STAT1
IRF2
ISG15
STAT2
IRF5
SMARCA4
PSMB8
IFIT3
GTF2F1
IRF4
MX2
IFIT2
EGR1
IRF6
SMARCB1
RSAD2
OASL
OAS2
IFIT1
OAS1
HLA-A
HLA-G
IRF9
IFI44L
HLA-B
IFI27
OAS3
HLA-F
SAMHD1
BST2
XAF1
IP6K2
RNASEL
IFITM2
IFI6
IFI35
HLA-E
HLA-C
IFITM1
IFIT5

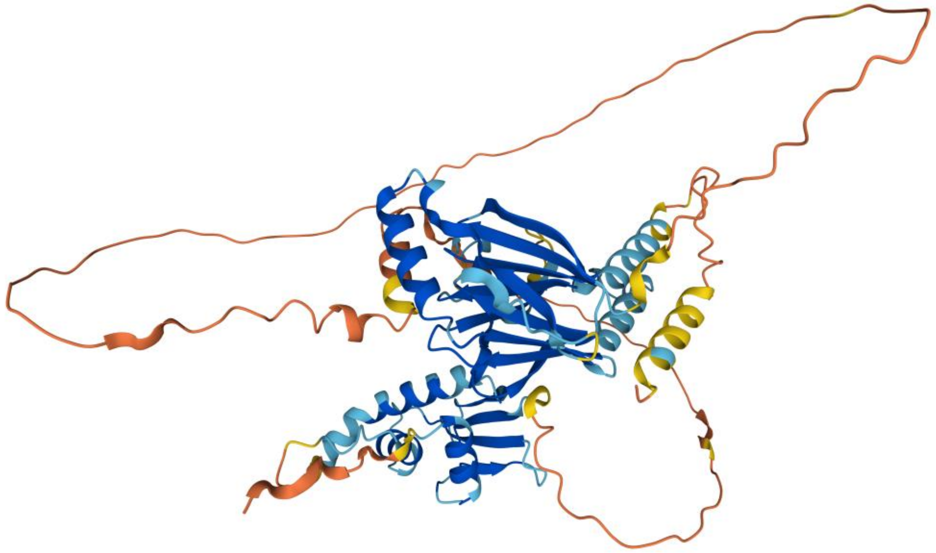

>sp|Q9UHD2|TBK1_HUMAN Serine/threonine-protein kinase TBK1 OS=Homo sapiens OX=9606 GN=TBK1 PE=1 SV=1
MQSTSNHLWLLSDILGQGATANVFRGRHKKTGDLFAIKVFNNISFLRPVDVQMREFEVLKKLNHKNIVKLFAIEEETTTRHKVLI
MEFCPCGSLYTVLEEPSNAYGLPESEFLIVLRDVVGGMNHLRENGIVHRDIKPGNIMRVIGEDGQSVYKLTDFGAARELEDDEQF
VSLYGTEEYLHPDMYERAVLRKDHQKKYGATVDLWSIGVTFYHAATGSLPFRPFEGPRRNKEVMYKIITGKPSGAISGVQKAENG
PIDWSGDMPVSCSLSRGLQVLLTPVLANILEADQEKCWGFDQFFAETSDILHRMVIHVFSLQQMTAHKIYIHSYNTATIFHELVY
KQTKIISSNQELIYEGRRLVLEPGRLAQHFPKTTEENPIFVVSREPLNTIGLIYEKISLPKVHPRYDLDGDASMAKAITGVVCYA
CRIASTLLLYQELMRKGIRWLIELIKDDYNETVHKKTEVVITLDFCIRNIEKTVKVYEKLMKINLEAAELGEISDIHTKLLRLSS
SQGTIETSLQDIDSRLSPGGSLADAWAHQEGTHPKDRNVEKLQVLLNCMTEIYYQFKKDKAERRLAYNEEQIHKFDKQKLYYHAT
KAMTHFTDECVKKYEAFLNKSEEWIRKMLHLRKQLLSLTNQCFDIEEEVSKYQEYTNELQETLPQKMFTASSGIKHTMTPIYPSS
NTLVEMTLGMKKLKEEMEGVVKELAENNHILERFGSLTMDGGLRNVDCL

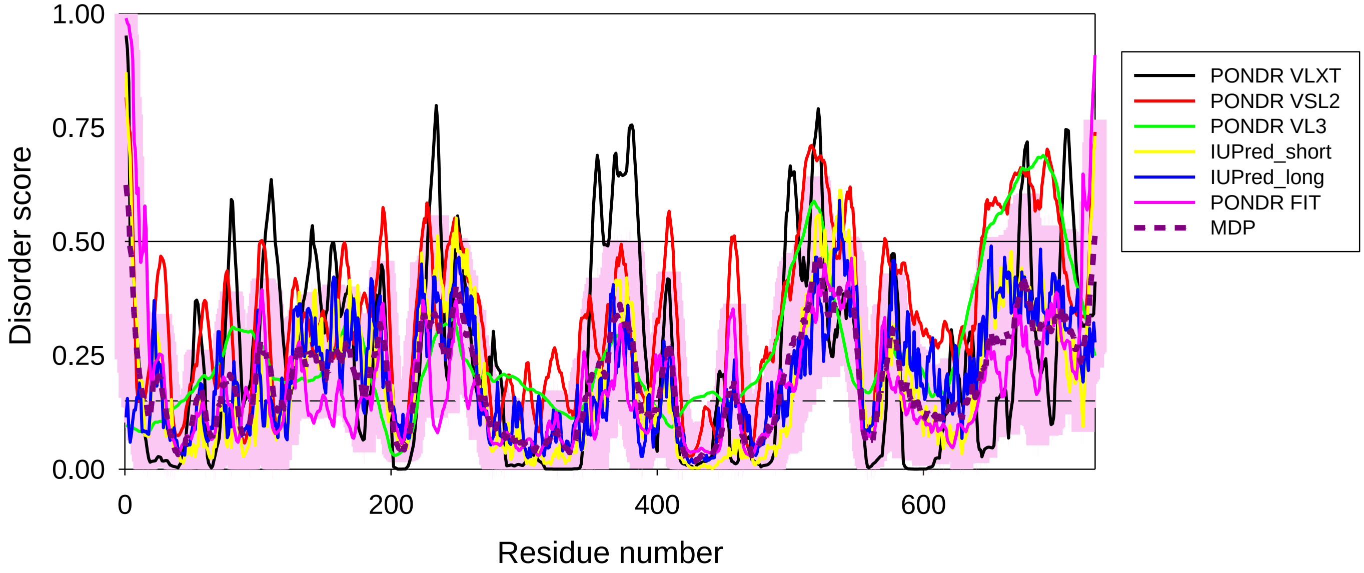

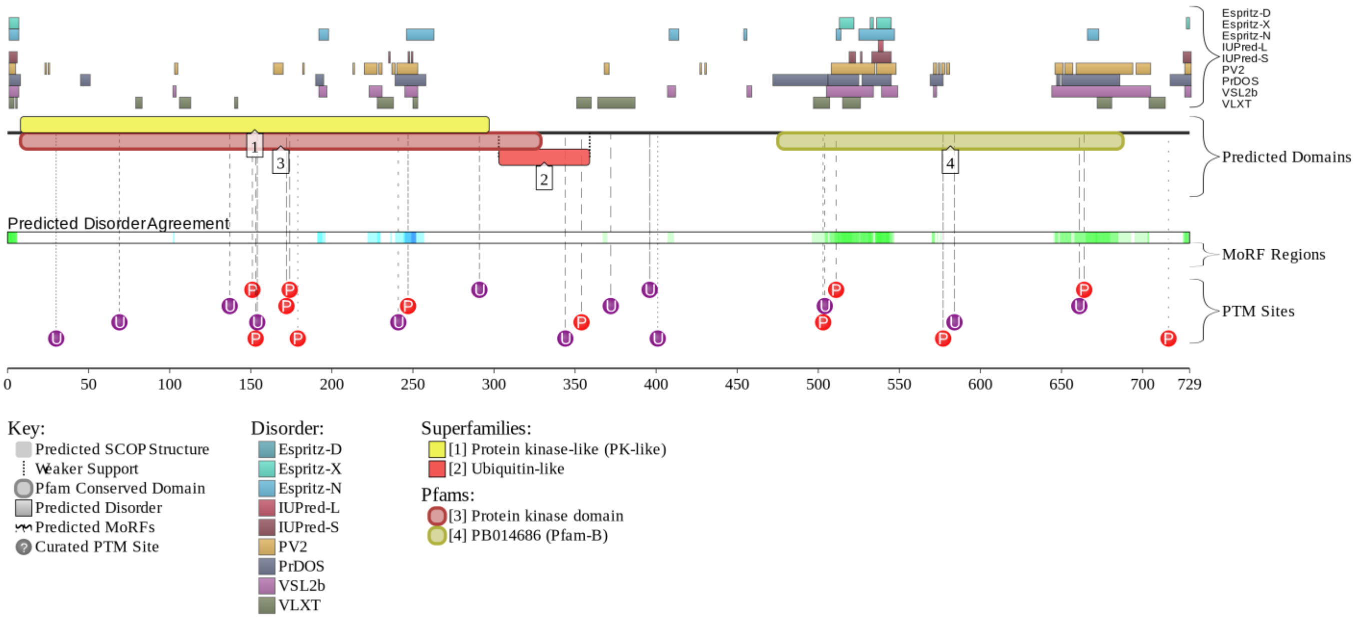

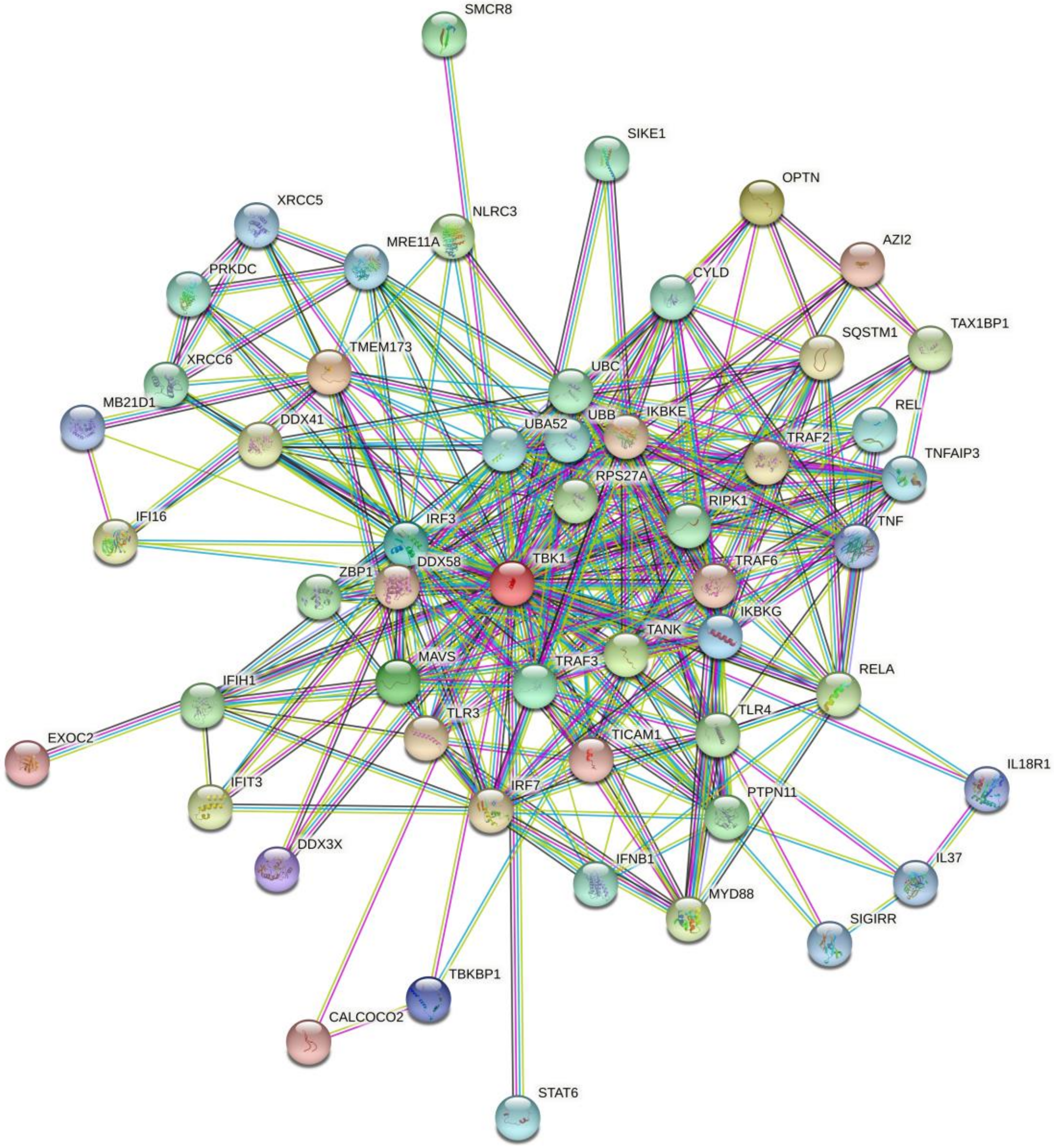
SMCR8
SIKE1
OPTN
XRCC5
NLRC3
AZI2
MRE11A
PRKDC
CYLD
SQSTM1
TAX1BP1
TMEM173
XRCC6
UBC
MB21D1
UBB
IKBKE
REL
DDX41
UBA52
TRAF2
TNFAIP3
RPS27A
IFI16
RIPK1
IRF3
TNF
TBK1
DDX58
TRAF6
ZBP1
IKBKG
TANK
MAVS
TRAF3
RELA
IFIH1
TLR3
TLR4
EXOC2
TICAM1
IL18R1
IFIT3
IRF7
PTPN11
DDX3X
IFNB1
IL37
MYD88
SIGIRR
TBKBP1
CALCOCO2
STAT6

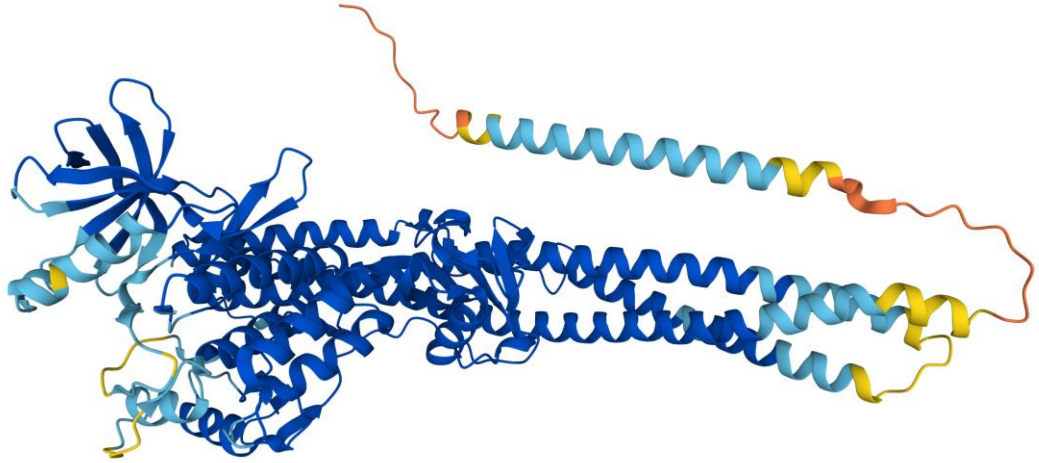

>sp|Q92844|TANK_HUMAN TRAF family member-associated NF-kappa-B activator OS=Homo sapiens OX=9606 GN=TANK PE=1 SV=2

MDKNIGEQLNKAYEAFRQACMDRDSAVKELQQKTENYEQRIREQQEQLSLQQTIIDKLKSQLLLVNSTQDNNYGCVPLLEDSETR
KNNLTLDQPQDKVISGIAREKLPKVRRQEVSSPRKETSARSLGSPLLHERGNIEKTFWDLKEEFHKICMLAKAQKDHLSKLNIPD
TATETQCSVPIQCTDKTDKQEALFKPQAKDDINRGAPSITSVTPRGLCRDEEDTSFESLSKFNVKFPPMDNDSTFLHSTPERPGI
LSPATSEAVCQEKFNMEFRDNPGNFVKTEETLFEIQGIDPIASAIQNLKTTDKTKPSNLVNTCIRTTLDRAACLPPGDHNALYVN
SFPLLDPSDAPFPSLDSPGKAIRGPQQPIWKPFPNQDSDSVVLSGTDSELHIPRVCEFCQAVFPPSITSRGDFLRHLNSHFNGET

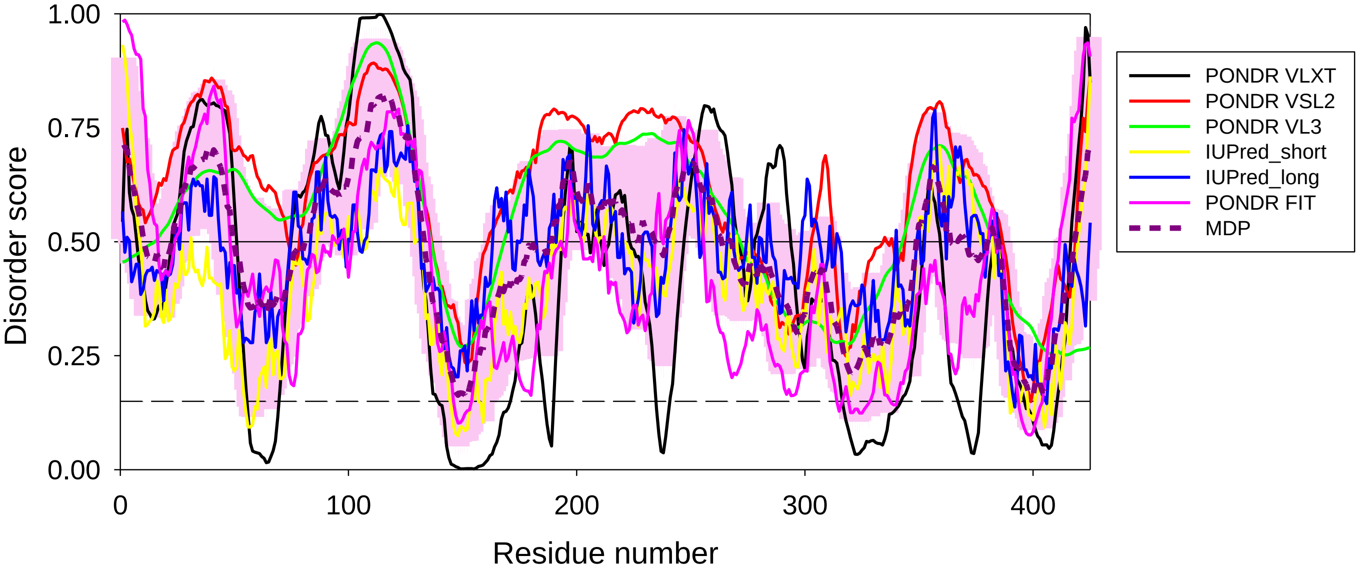

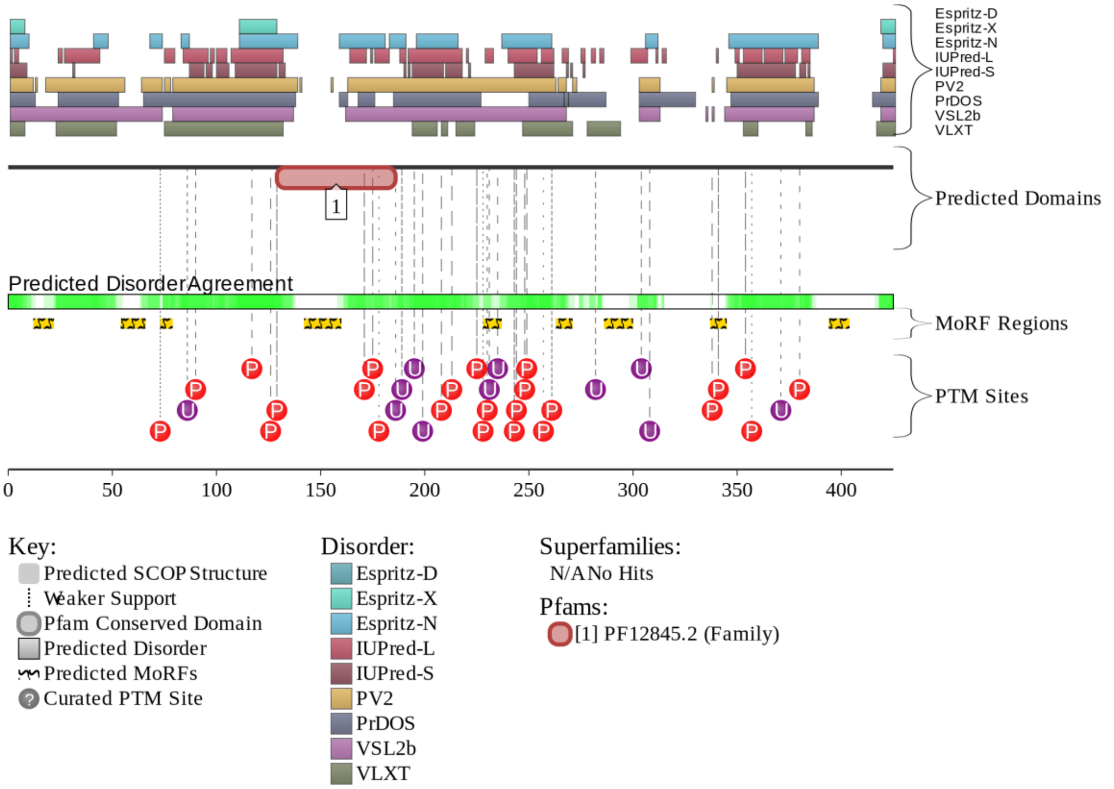

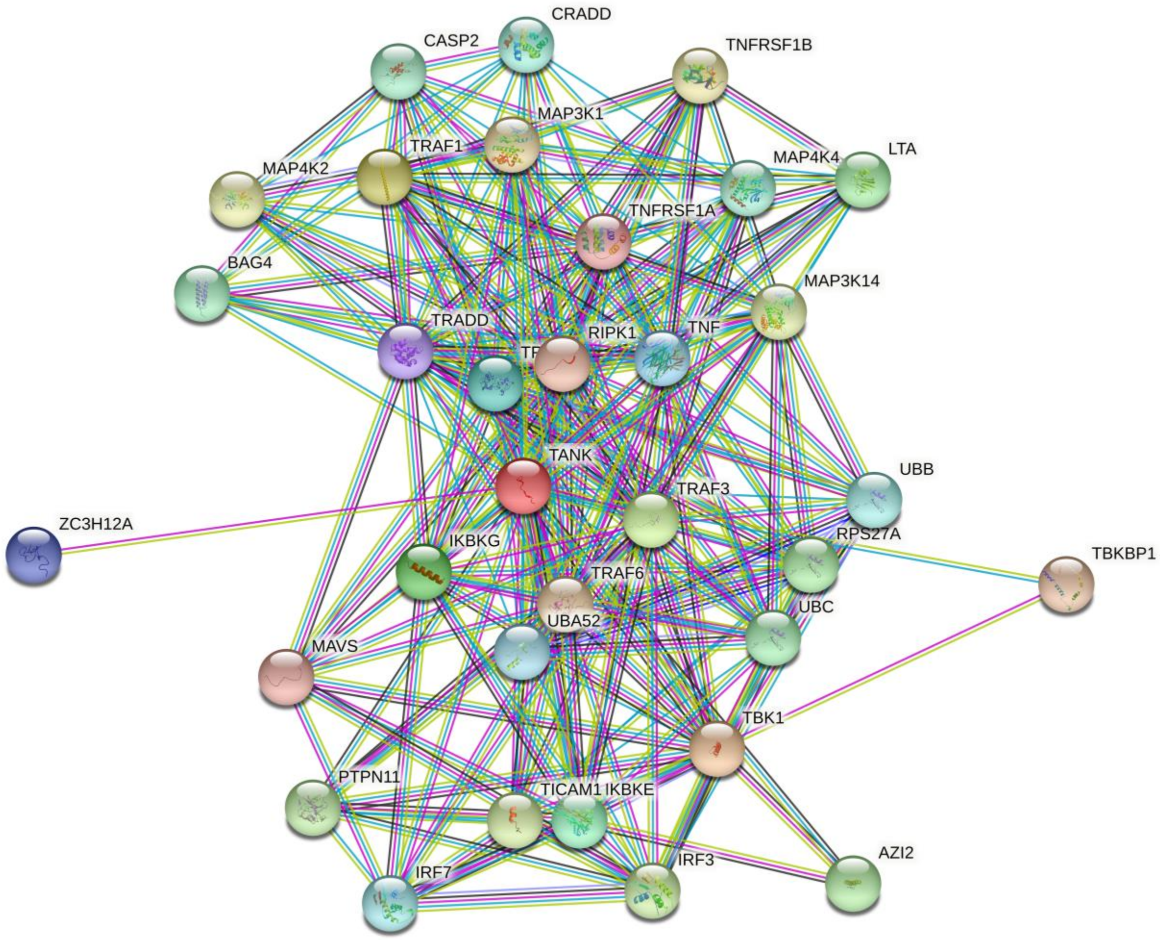
CRADD
CASP2
TNFRSF1B
MAP3K1
TRAF1
MAP4K2
MAP4K4
LTA
TNFRSF1A
BAG4
MAP3K14
TRADD
RIPK1
TNF
TANK
UBB
ZC3H12A
TRAF3
RPS27A
TBKBP1
IKBKG
TRAF6
UBA52
UBC
MAVS
TBK1
PTPN11
TICAM1
IKBKE
AZI2
IRF7
IRF3

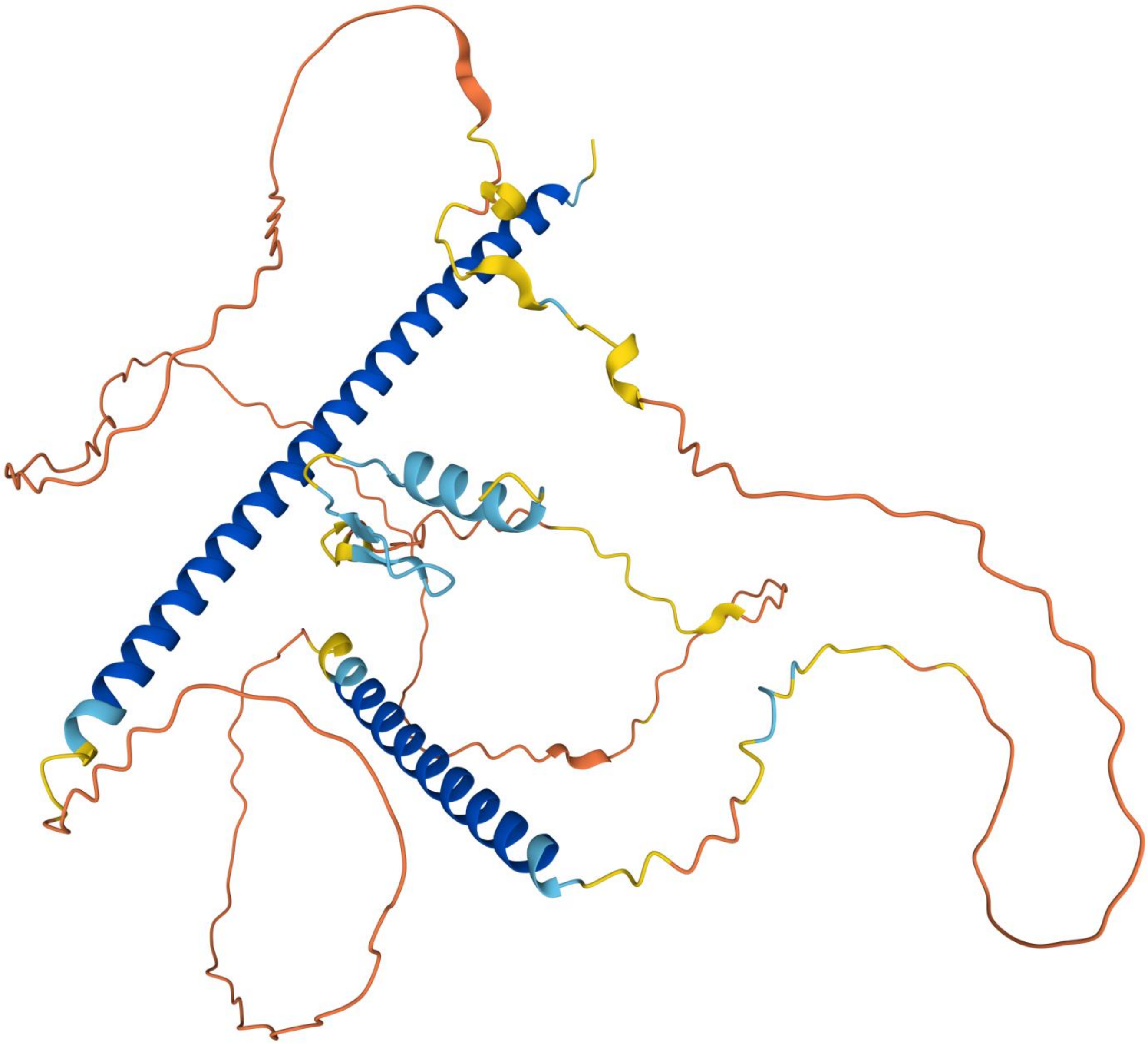

>sp|Q8IUC6|TCAM1_HUMAN TIR domain-containing adapter molecule 1 OS=Homo sapiens OX=9606 GN=TICAM1_TRIF PE=1 SV=1
MACTGPSLPSAFDILGAAGQDKLLYLKHKLKTPRPGCQGQDLLHAMVLLKLGQETEARISLEALKADAVARLVARQWAGVDSTED
PEEPPDVSWAVARLYHLLAEEKLCPASLRDVAYQEAVRTLSSRDDHRLGELQDEARNRCGWDIAGDPGSIRTLQSNLGCLPPSSA
LPSGTRSLPRPIDGVSDWSQGCSLRSTGSPASLASNLEISQSPTMPFLSLHRSPHGPSKLCDDPQASLVPEPVPGGCQEPEEMSW
PPSGEIASPPELPSSPPPGLPEVAPDATSTGLPDTPAAPETSTNYPVECTEGSAGPQSLPLPILEPVKNPCSVKDQTPLQLSVED
TTSPNTKPCPPTPTTPETSPPPPPPPPPSSTPCSAHLTPSSLFPSSLESSSEQKFYNFVILHARADEHIALRVREKLEALGVPDGA
TFCEDFQVPGRGELSCLQDAIDHSAFIILLLTSNFDCRLSLHQVNQAMMSNLTRQGSPDCVIPFLPLESSPAQLSSDTASLLSGL
VRLDEHSQIFARKVANTFKPHRLQARKAMWRKEQDTRALREQSQHLDGERMQAAALNAAYSAYLQSYLSYQAQMEQLQVAFGSHM
SFGTGAPYGARMPFGGQVPLGAPPPFPTWPGCPQPPPLHAWQAGTPPPPSPQPAAFPQSLPFPQSPAFPTASPAPPQSPGLQPLI
IHHAQMVQLGLNNHMWNQRGSQAPEDKTQEAE

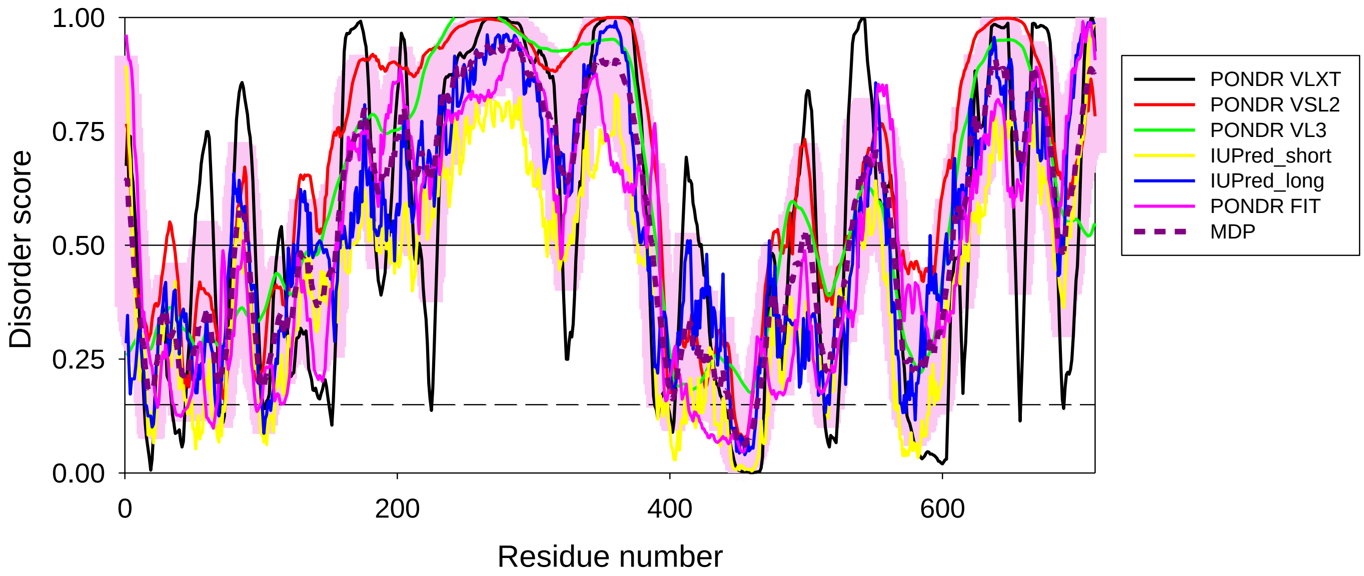

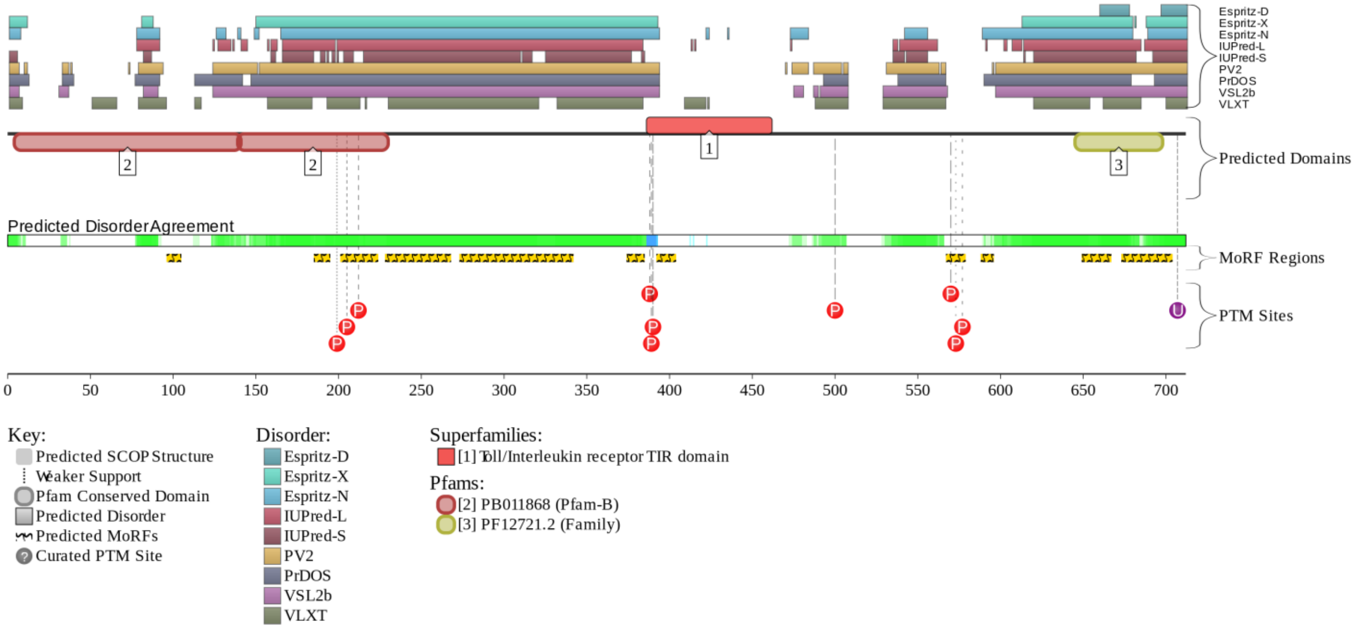

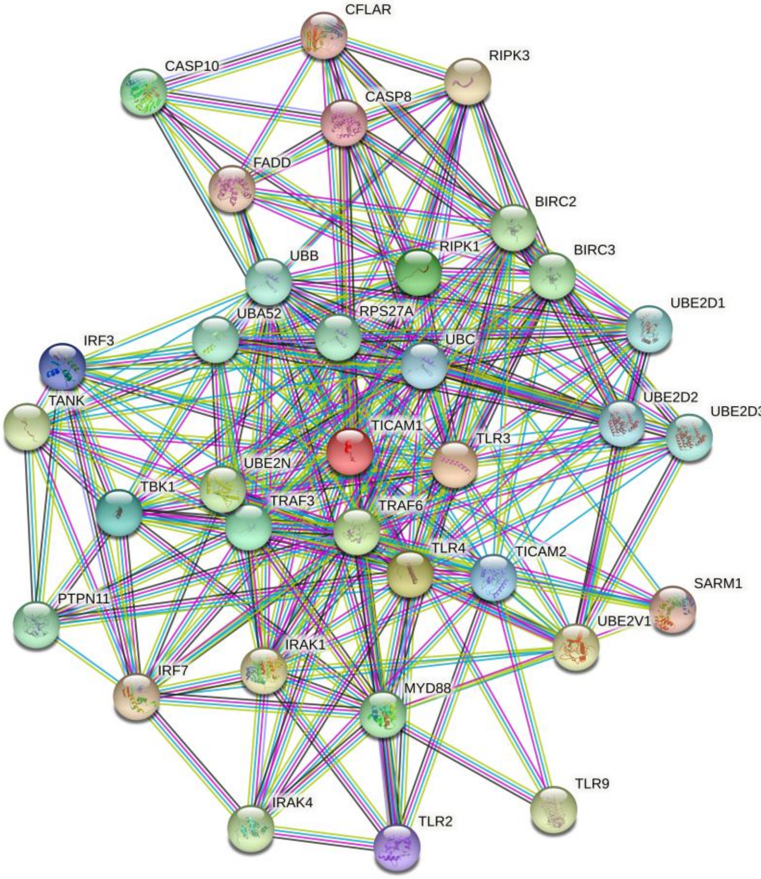
CFLAR
RIPK3
CASP10
CASP8
FADD
BIRC2
RIPK1
BIRC3
UBB
UBE2D1
UBA52
RPS27A
UBC
IRF3
TANK
UBE2D2
UBE2D3
TICAM1
TLR3
UBE2N
TBK1
TRAF3
TRAF6
TLR4
TICAM2
SARM1
PTPN11
UBE2V1
IRAK1
IRF7
MYD88
TLR9
IRAK4
TLR2

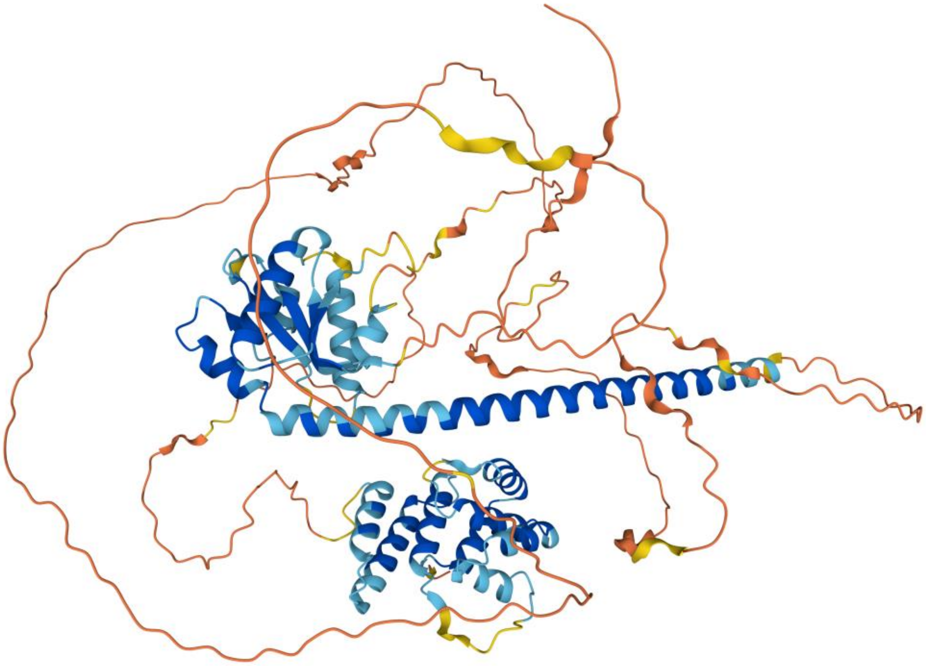

>sp|Q13546|RIPK1_HUMAN Receptor-interacting serine/threonine-protein kinase 1 OS=Homo sapiens OX=9606 GN=RIPK1 PE=1 SV=3
MQPDMSLNVIKMKSSDFLESAELDSGGFGKVSLCFHRTQGLMIMKTVYKGPNCIEHNEALLEEAKMMNRLRHSRVVKLLGVIIEE
GKYSLVMEYMEKGNLMHVLKAEMSTPLSVKGRIILEIIEGMCYLHGKGVIHKDLKPENILVDNDFHIKIADLGLASFKMWSKLNN
EEHNELREVDGTAKKNGGTLYYMAPEHLNDVNAKPTEKSDVYSFAVVLWAIFANKEPYENAICEQQLIMCIKSGNRPDVDDITEY
CPREIISLMKLCWEANPEARPTFPGIEEKFRPFYLSQLEESVEEDVKSLKKEYSNENAVVKRMQSLQLDCVAVPSSRSNSATEQP
GSLHSSQGLGMGPVEESWFAPSLEHPQEENEPSLQSKLQDEANYHLYGSRMDRQTKQQPRQNVAYNREEERRRRVSHDPFAQQRP
YENFQNTEGKGTAYSSAASHGNAVHQPSGLTSQPQVLYQNNGLYSSHGFGTRPLDPGTAGPRVWYRPIPSHMPSLHNIPVPETNY
LGNTPTMPFSSLPPTDESIKYTIYNSTGIQIGAYNYMEIGGTSSSLLDSTNTNFKEEPAAKYQAIFDNTTSLTDKHLDPIRENLG
KHWKNCARKLGFTQSQIDEIDHDYERDGLKEKVYQMLQKWVMREGIKGATVGKLAQALHQCSRIDLLSSLIYVSQN

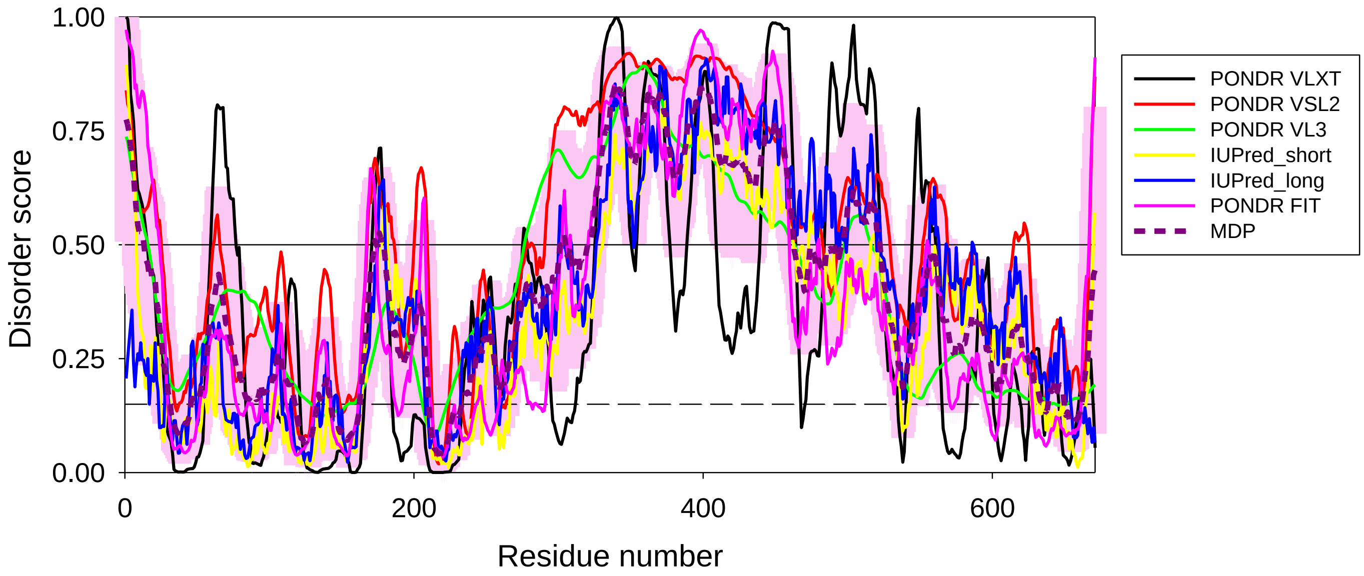

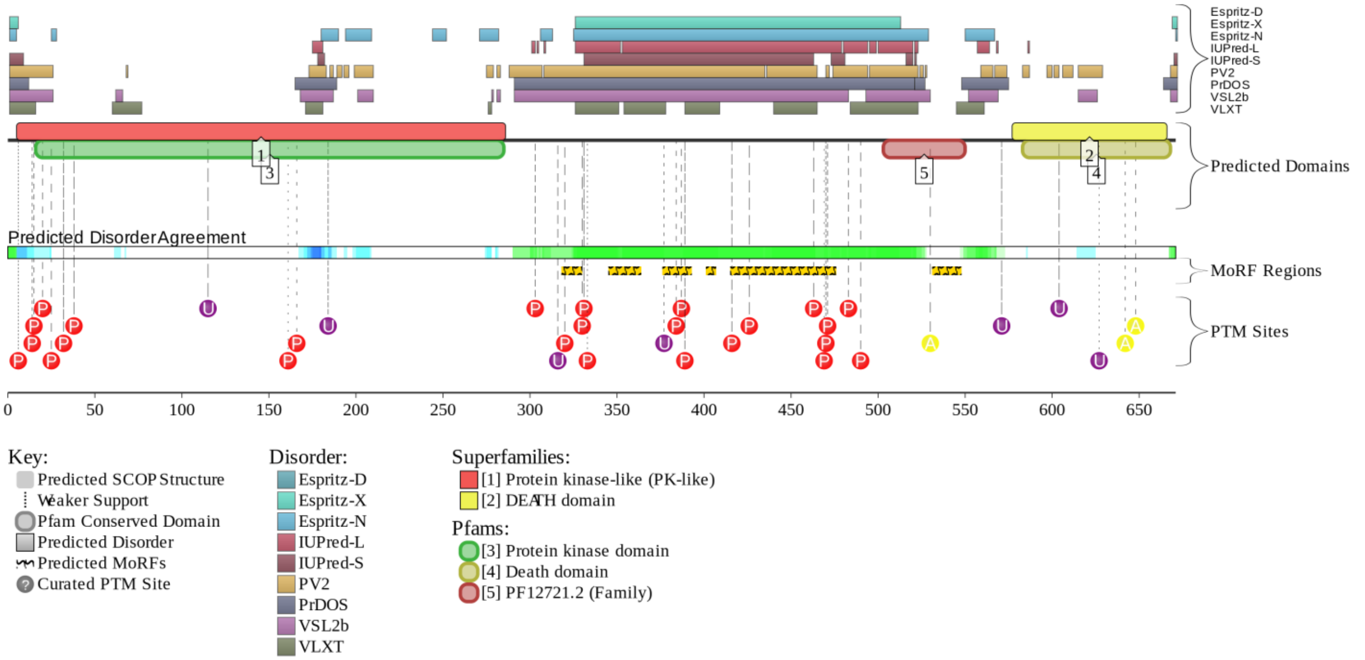

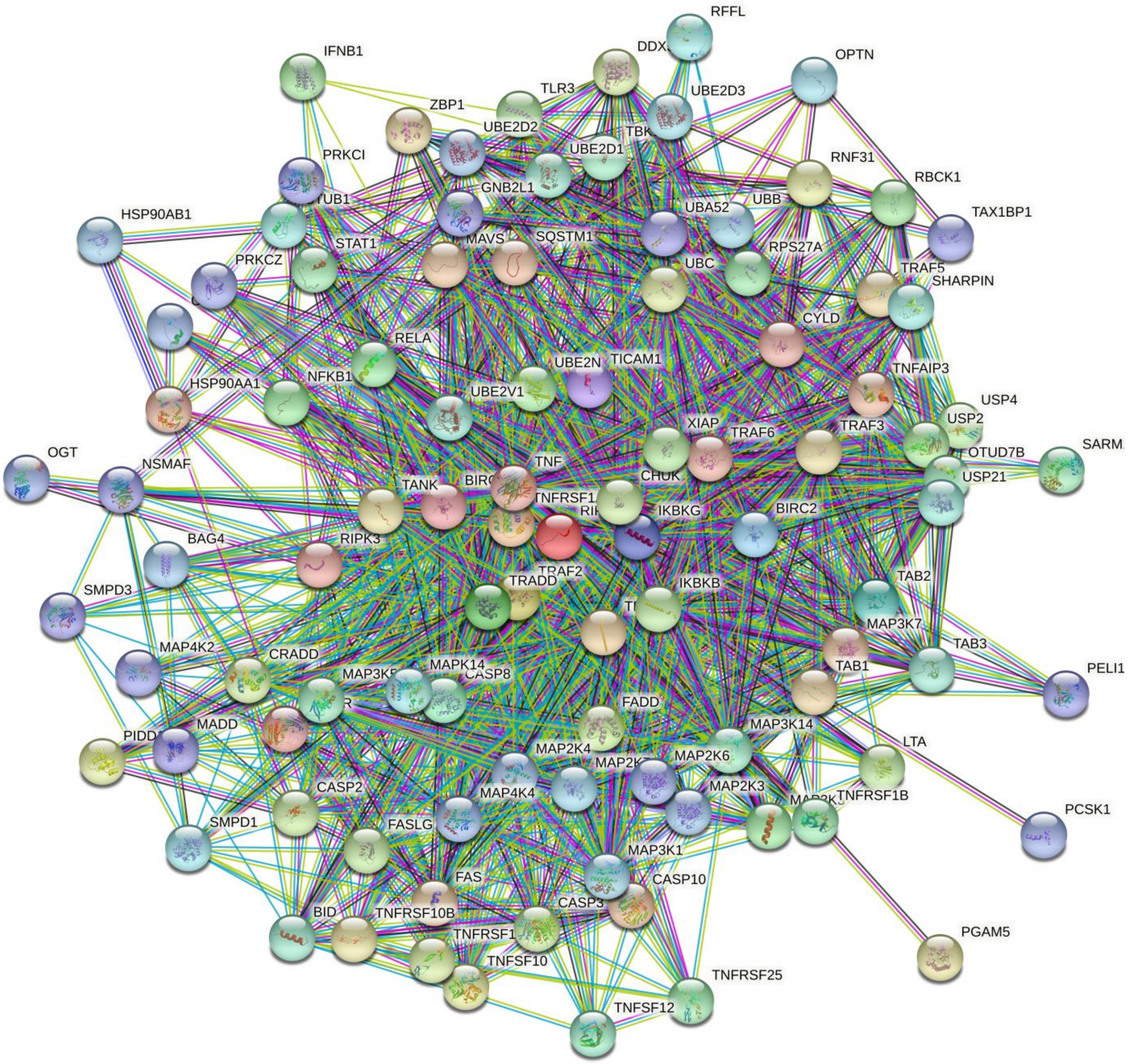
RFFL
IFNB1
OPTN
TLR3
UBE2D3
ZBP1
UBE2D2
UBE2D1
PRKCI
RNF31
RBCK1
GNB2L1
TAX1BP1
HSP90AB1
UBA52
UBB
MAVS
SQSTM1
STAT1
RPS27A
PRKCZ
UBC
TRAF5
SHARPIN
CYLD
RELA
TICAM1
UBE2N
NFKB1
HSP90AA1
TNFAIP3
USP4
UBE2V1
USP2
XIAP
TRAF6
TRAF3
OGT
NSMAF
OTUD7B
TNF
USP21
TANK
CHUK
IKBKG
BIRC2
BAG4
RIPK3
TRAF2
TRADD
SMPD3
IKBKB
TAB2
MAP3K7
MAP4K2
CRADD
TAB3
MAPK14
CASP8
TAB1
PELI1
FADD
MAP3K14
MADD
LTA
MAP2K4
MAP2K6
MAP2K3
TNFRSF1B
PCSK1
MAP4K4
CASP2
SMPD1
FASLG
MAP3K1
FAS
CASP10
BID
TNFRSF10B
CASP3
PGAM5
TNFSF10
TNFRSF25
TNFSF12

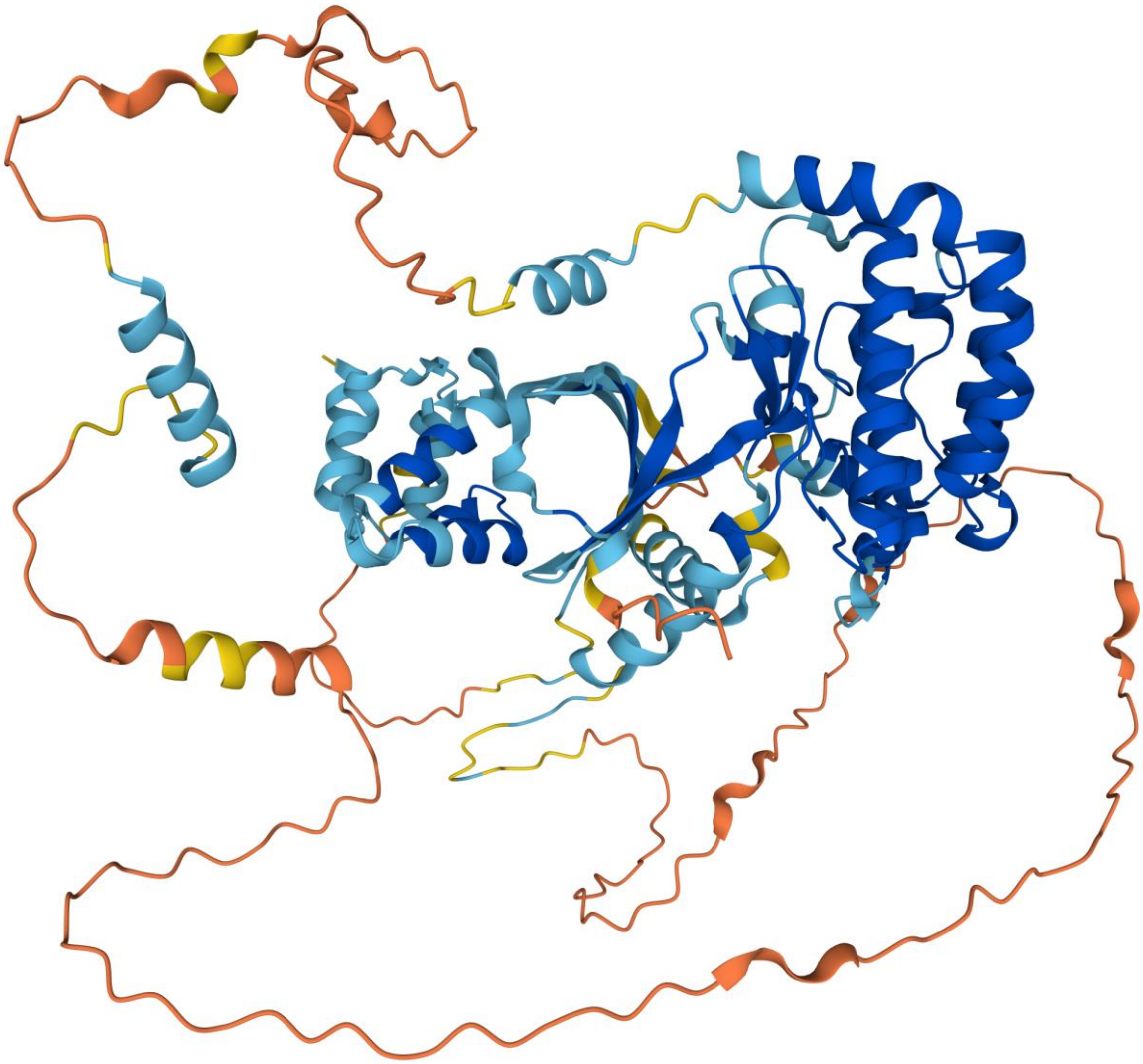

>sp|Q86XR7|TCAM2_HUMAN TIR domain-containing adapter molecule 2 OS=Homo sapiens OX=9606 GN=TICAM2 PE=1 SV=1
MGIGKSKINSCPLSLSWGKRHSVDTSPGYHESDSKKSEDLSLCNVAEHSNTTEGPTGKQEGAQSVEEMFEEEAEEEVFLKFVILH
AEDDTDEALRVQNLLQDDFGIKPGIIFAEMPCGRQHLQNLDDAVNGSAWTILLLTENFLRDTWCNFQFYTSLMNSVNRQHKYNSV
IPMRPLNNPLPRERTPFALQTINALEEESRGFPTQVERIFQESVYKTQQTIWKETRNMVQRQFIA

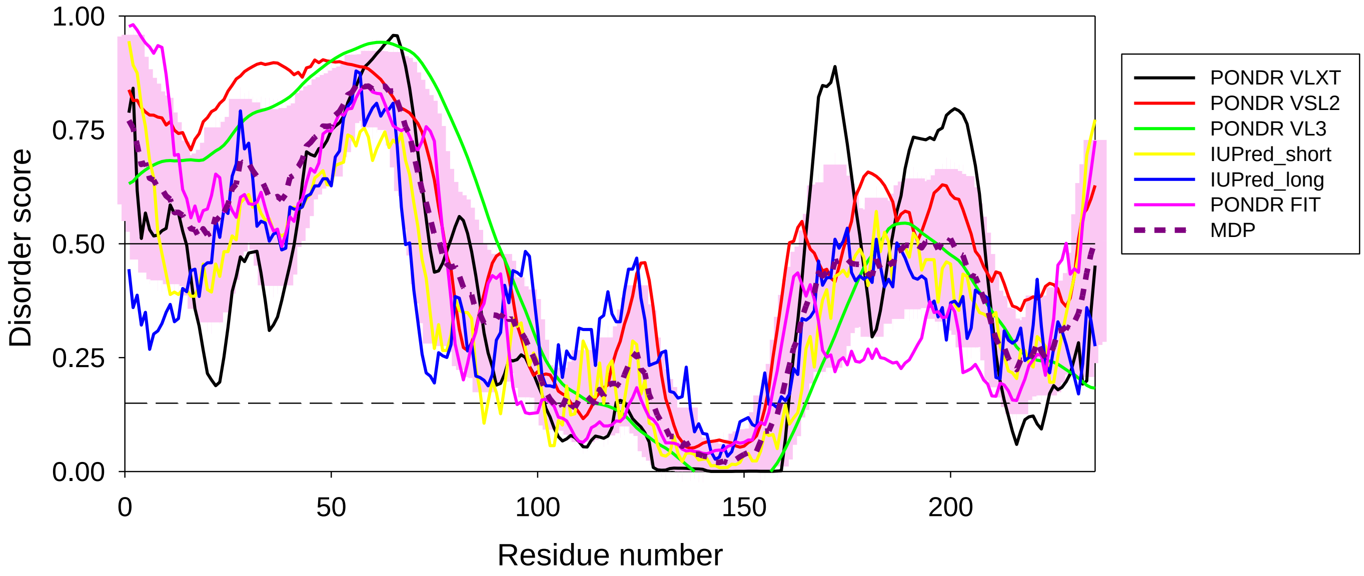

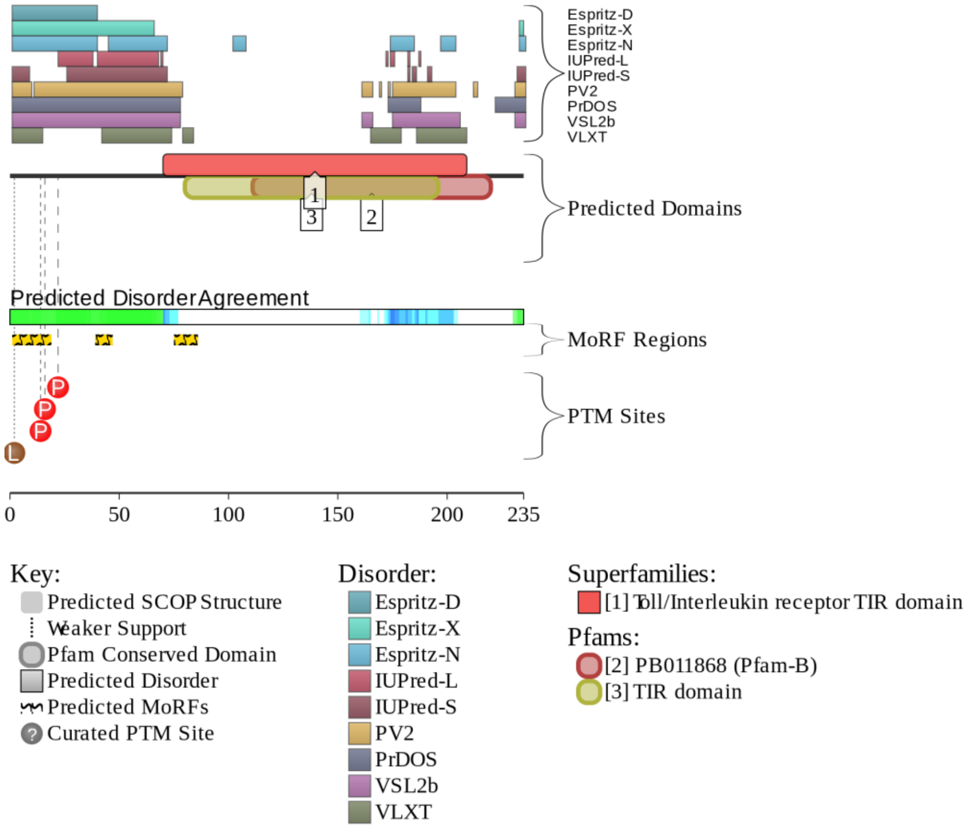

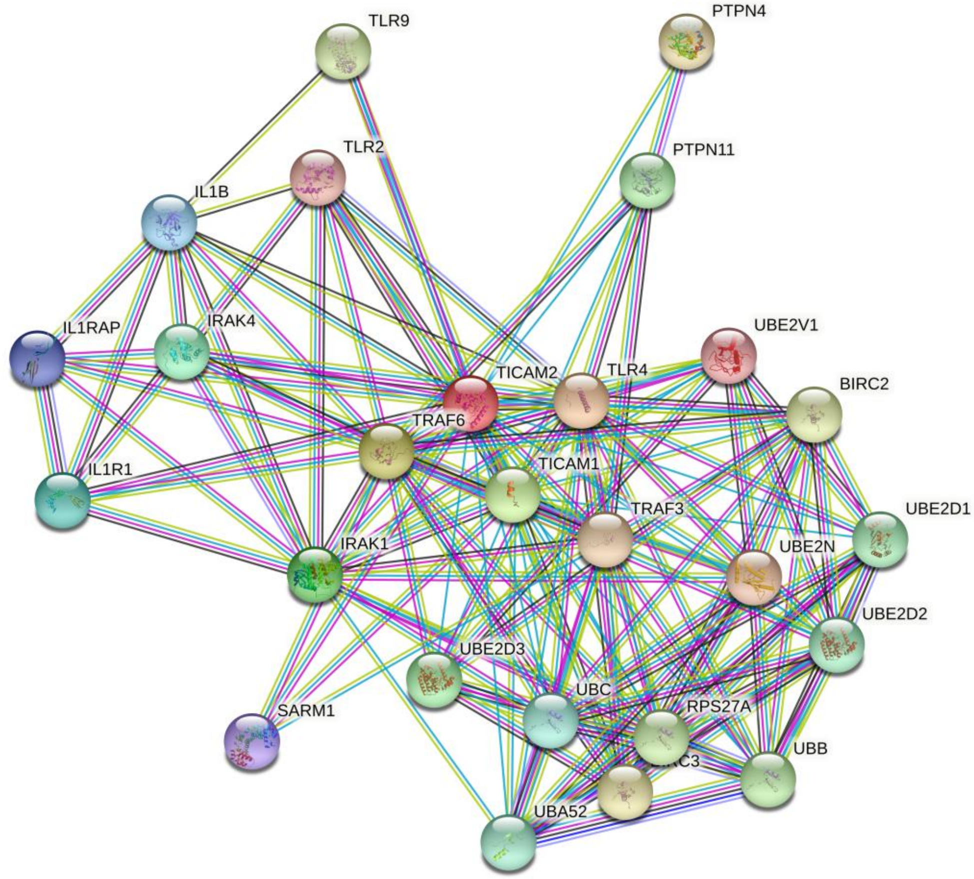
TLR9
PTPN4
TLR2
PTPN11
IL1B
IL1RAP
IRAK4
UBE2V1
TICAM2
TLR4
BIRC2
TRAF6
TICAM1
IL1R1
TRAF3
UBE2D1
IRAK1
UBE2N
UBE2D2
UBE2D3
UBC
SARM1
RPS27A
UBB
UBA52

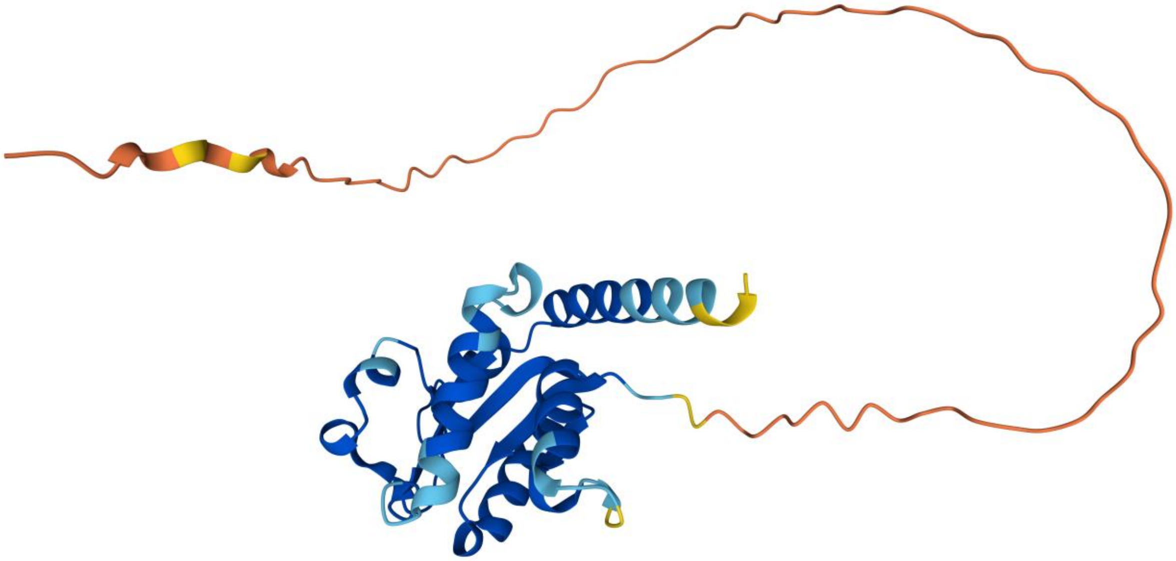

>sp|P01562|IFNA1_HUMAN Interferon alpha-1/13 OS=Homo sapiens OX=9606 GN=IFNA1 PE=1 SV=1
MASPFALLMVLVVLSCKSSCSLGCDLPETHSLDNRRTLMLLAQMSRISPSSCLMDRHDFGFPQEEFDGNQFQKAPAISVLHELIQ
QIFNLFTTKDSSAAWDEDLLDKFCTELYQQLNDLEACVMQEERVGETPLMNADSILAVKKYFRRITLYLTEKKYSPCAWEVVRAE
IMRSLSLSTNLQERLRRKE

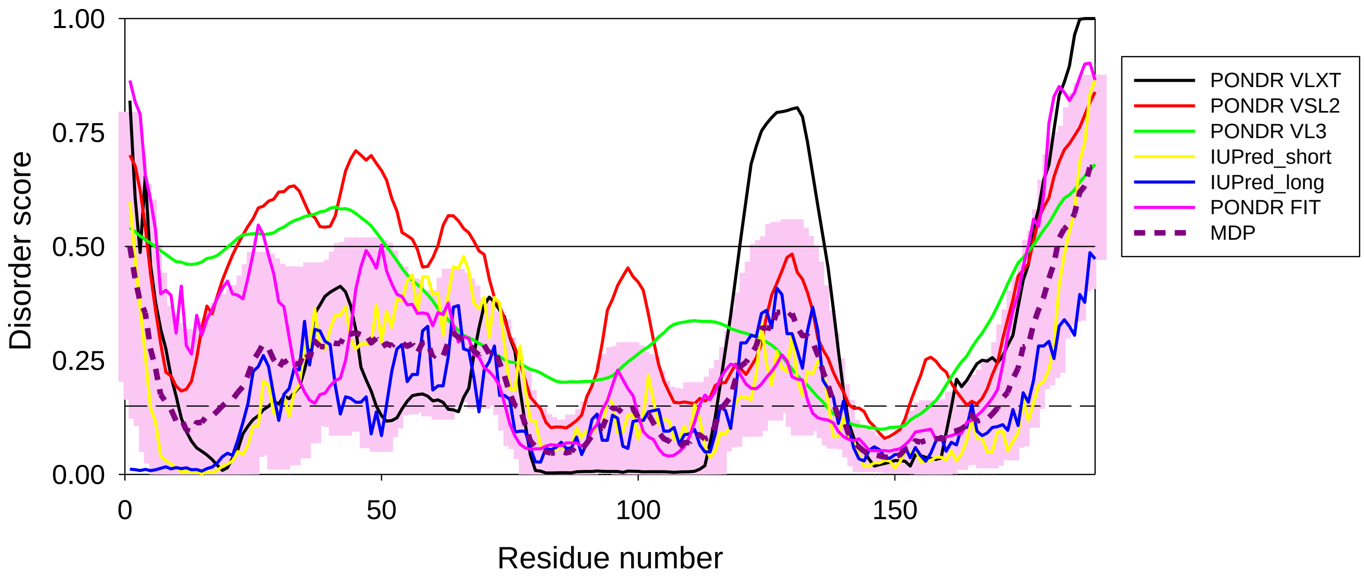

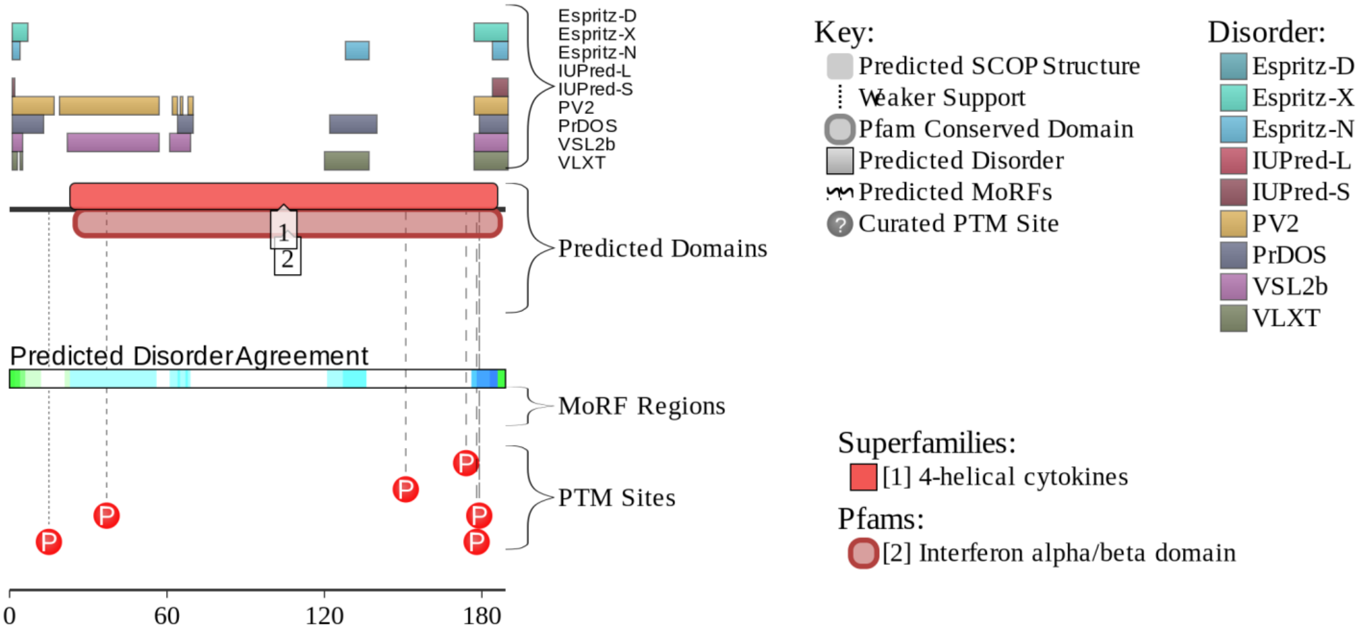

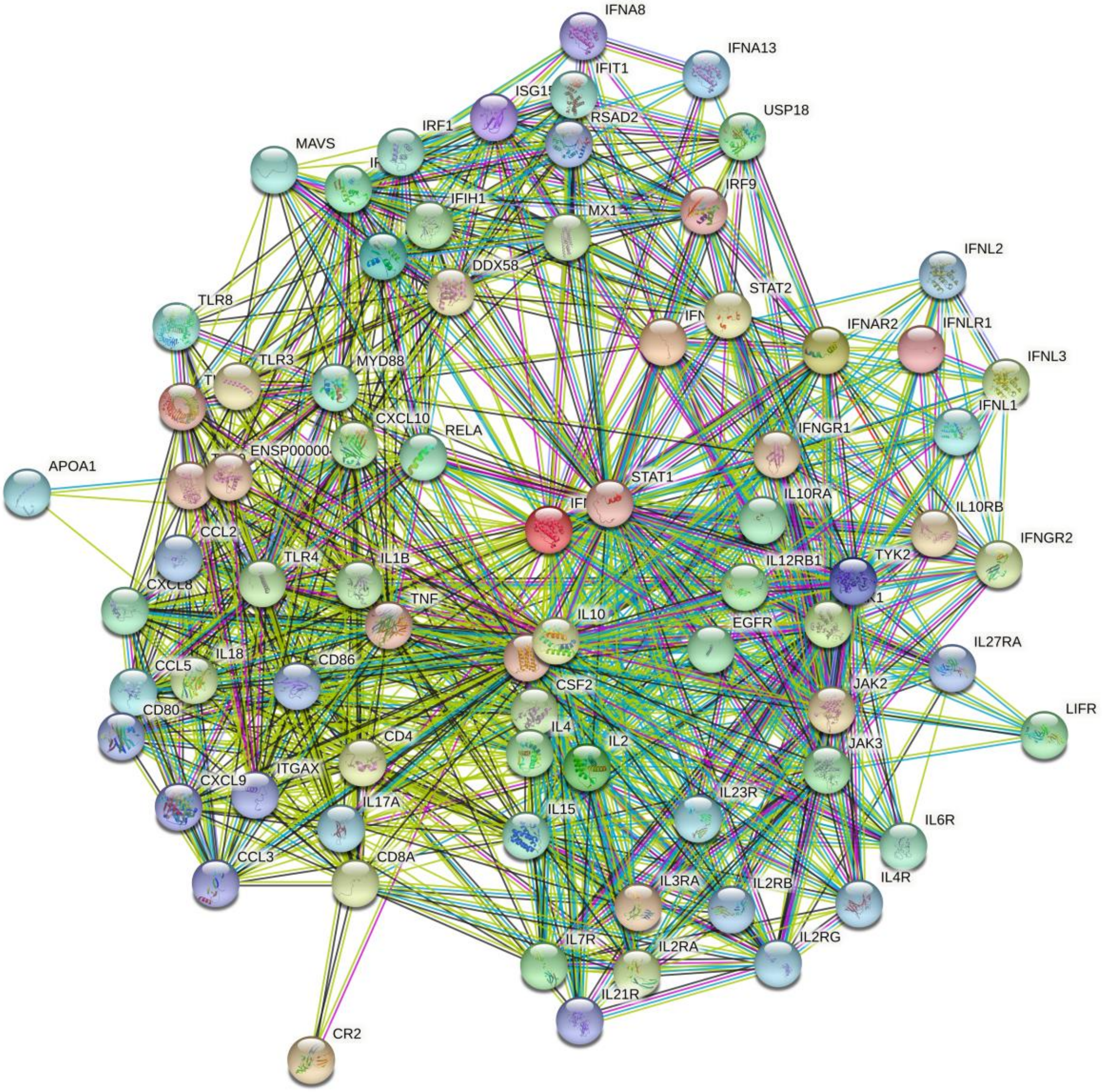
IFNA8
IFNA13
IFIT1
ISG15
USP18
RSAD2
IRF1
MAVS
IRF9
IFIH1
MX1
DDX58
IFNL2
TLR8
STAT2
IFNAR2
IFNLR1
IFNL3
TLR3
MYD88
IFNL1
CXCL10
IFNGR1
RELA
ENSP00000
APOA1
STAT1
IL10RA
IL10RB
CCL2
IFNGR2
TYK2
IL12RB1
TLR4
IL1B
TNF
IL10
EGFR
IL27RA
CCL5
IL18
CD86
CSF2
JAK2
CD80
LIFR
IL4
IL2
JAK3
CD4
ITGAX
CXCL9
IL17A
IL23R
IL15
IL6R
CCL3
CD8A
IL3RA
IL2RB
IL4R
IL7R
IL2RA
IL2RG
IL21R
CR2

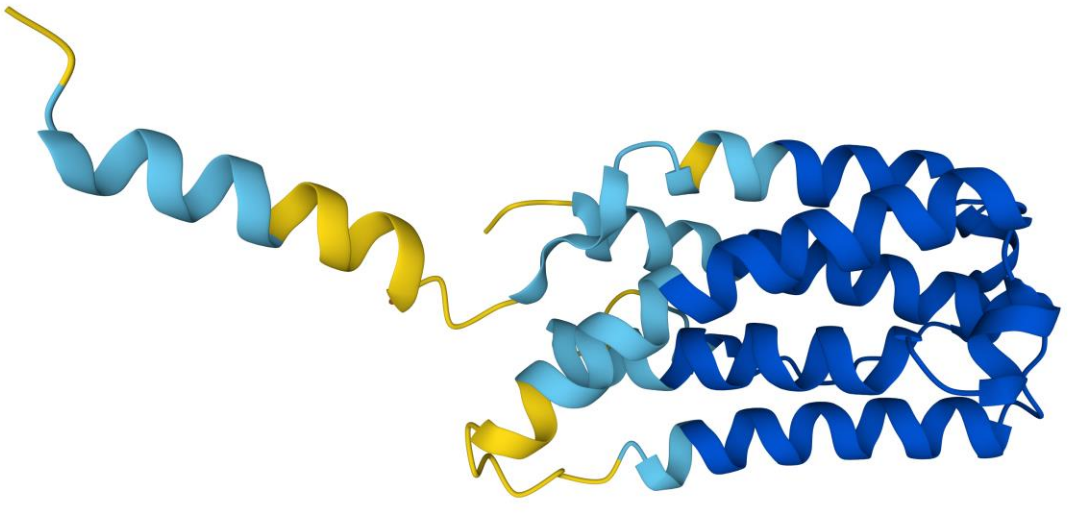

>sp|P01574|IFNB_HUMAN Interferon beta OS=Homo sapiens OX=9606 GN=IFNB1 PE=1 SV=1
MTNKCLLQIALLLCFSTTALSMSYNLLGFLQRSSNFQCQKLLWQLNGRLEYCLKDRMNFDIPEEIKQLQQFQKEDAALTIYEMLQ
NIFAIFRQDSSSTGWNETIVENLLANVYHQINHLKTVLEEKLEKEDFTRGKLMSSLHLKRYYGRILHYLKAKEYSHCAWTIVRVE
ILRNFYFINRLTGYLRN

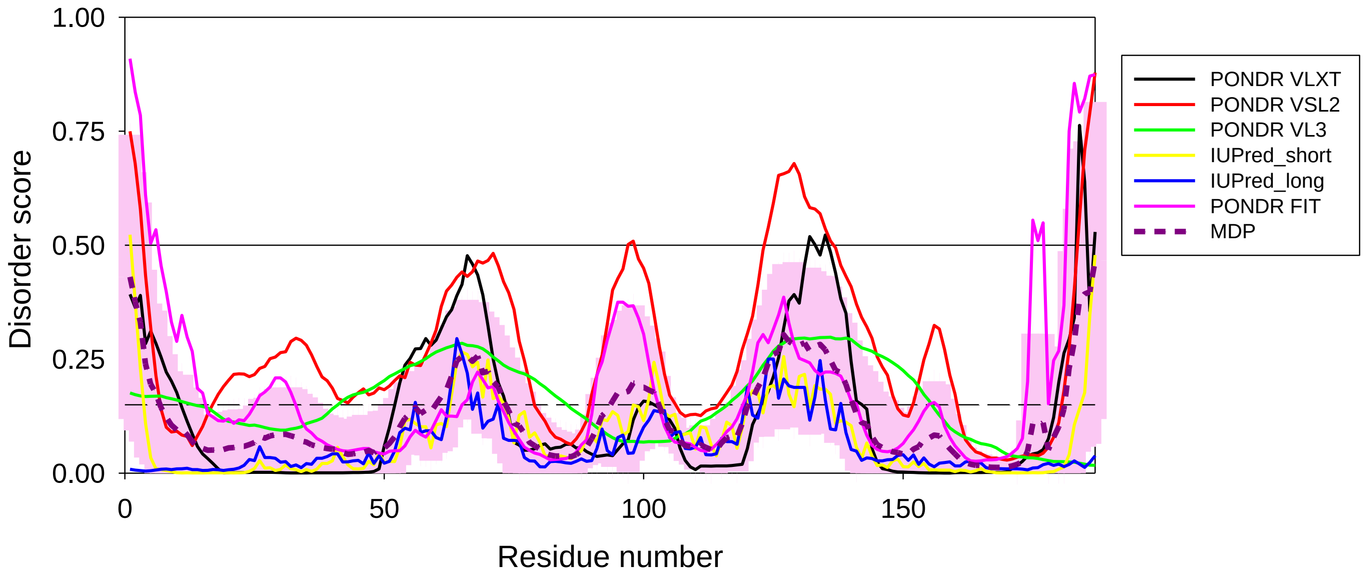

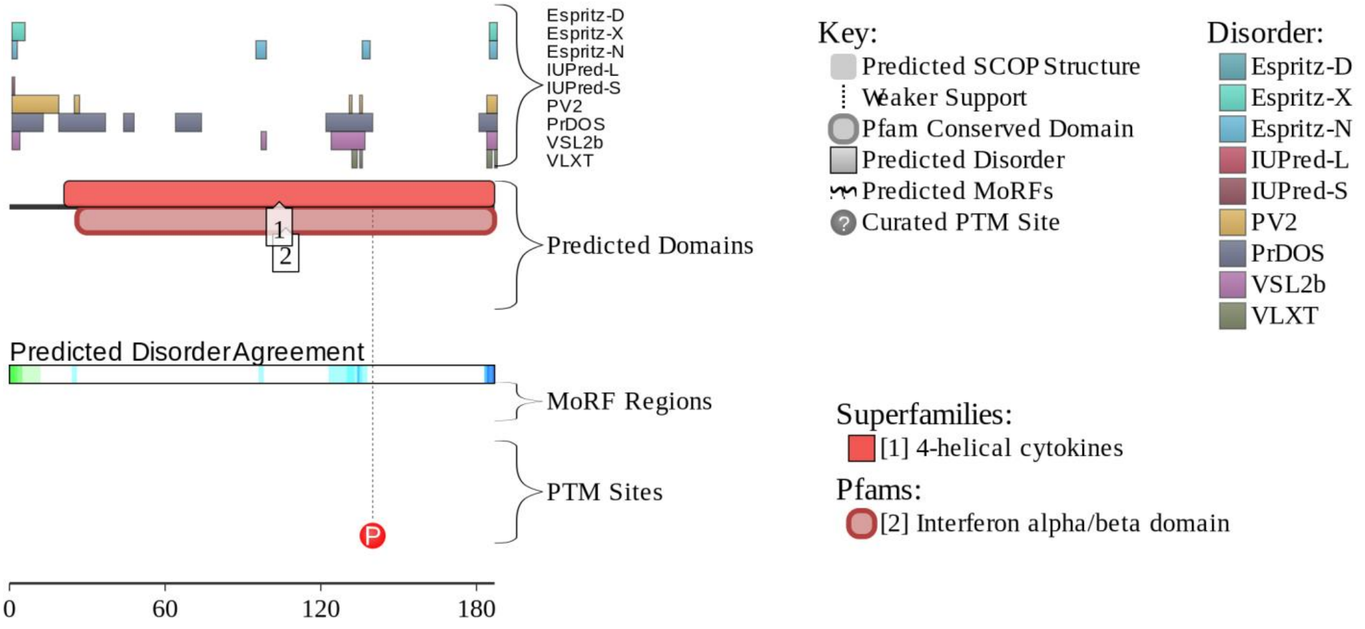

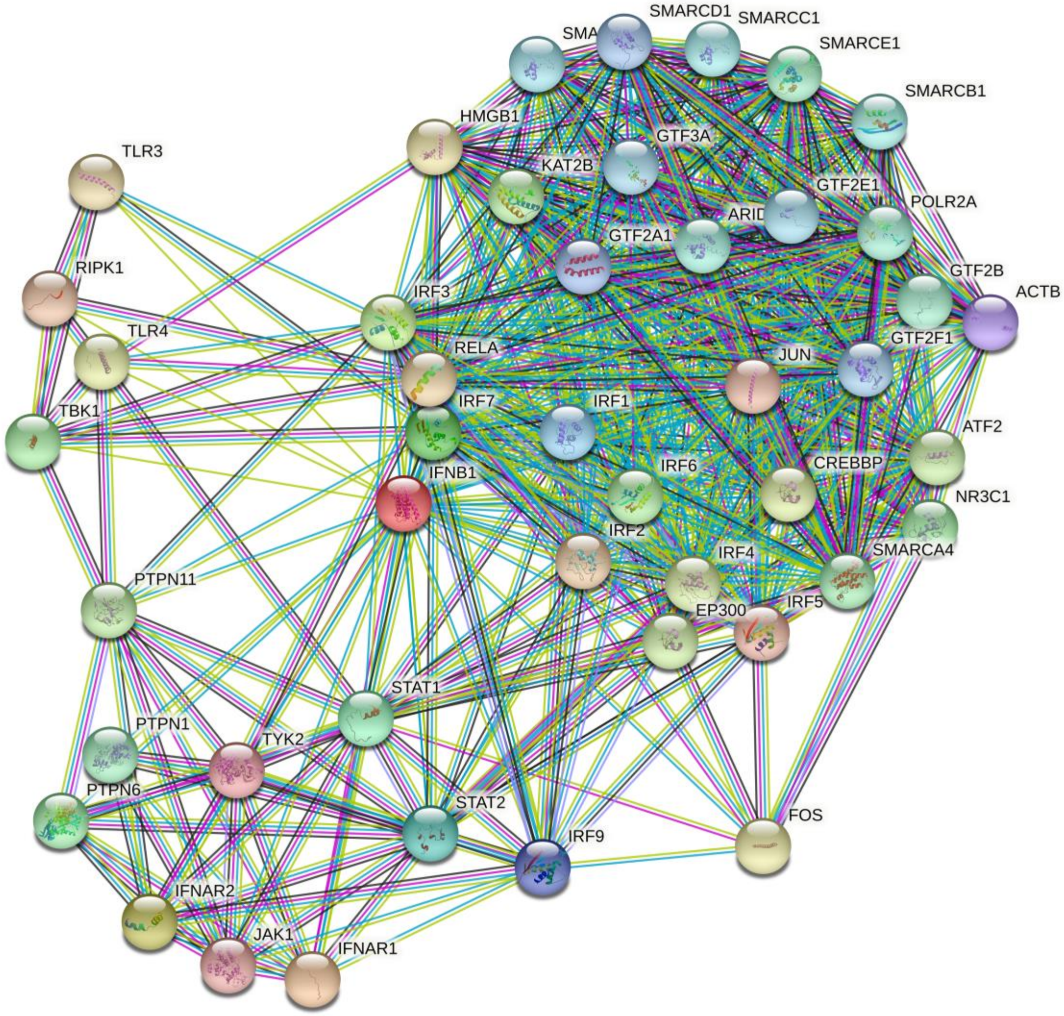
SMA
SMARCD1
SMARCC1
SMARCE1
SMARCB1
HMGB1
TLR3
GTF3A
KAT2B
GTF2E1
POLR2A
ARID
GTF2A1
RIPK1
GTF2B
ACTB
IRF3
TLR4
RELA
JUN
GTF2F1
TBK1
IRF7
IRF1
ATF2
IFNB1
CREBBP
IRF6
NR3C1
IRF2
SMARCA4
IRF4
PTPN11
EP300
IRF5
STAT1
PTPN1
TYK2
PTPN6
STAT2
FOS
IRF9
IFNAR2
JAK1
IFNAR1

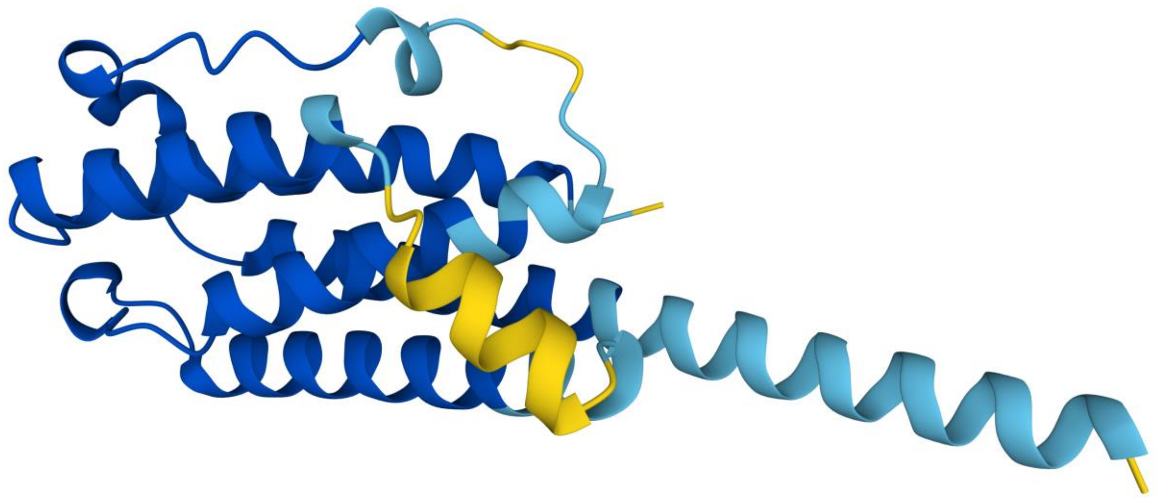

>sp|P05014|IFNA4_HUMAN Interferon alpha-4 OS=Homo sapiens OX=9606 GN=IFNA4 PE=1 SV=2
MALSFSLLMAVLVLSYKSICSLGCDLPQTHSLGNRRALILLAQMGRISHFSCLKDRHDFGFPEEEFDGHQFQKAQAISVLHEMIQ
QTFNLFSTEDSSAAWEQSLLEKFSTELYQQLNDLEACVIQEVGVEETPLMNEDSILAVRKYFQRITLYLTEKKYSPCAWEVVRAE
IMRSLSFSTNLQKRLRRKD

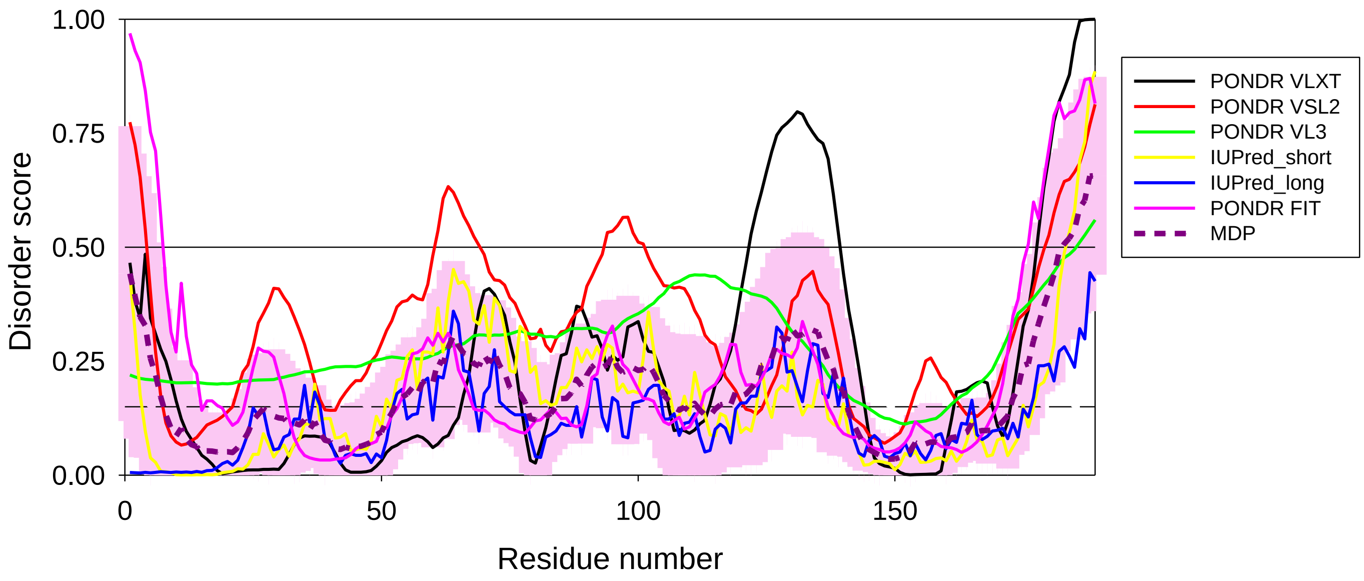

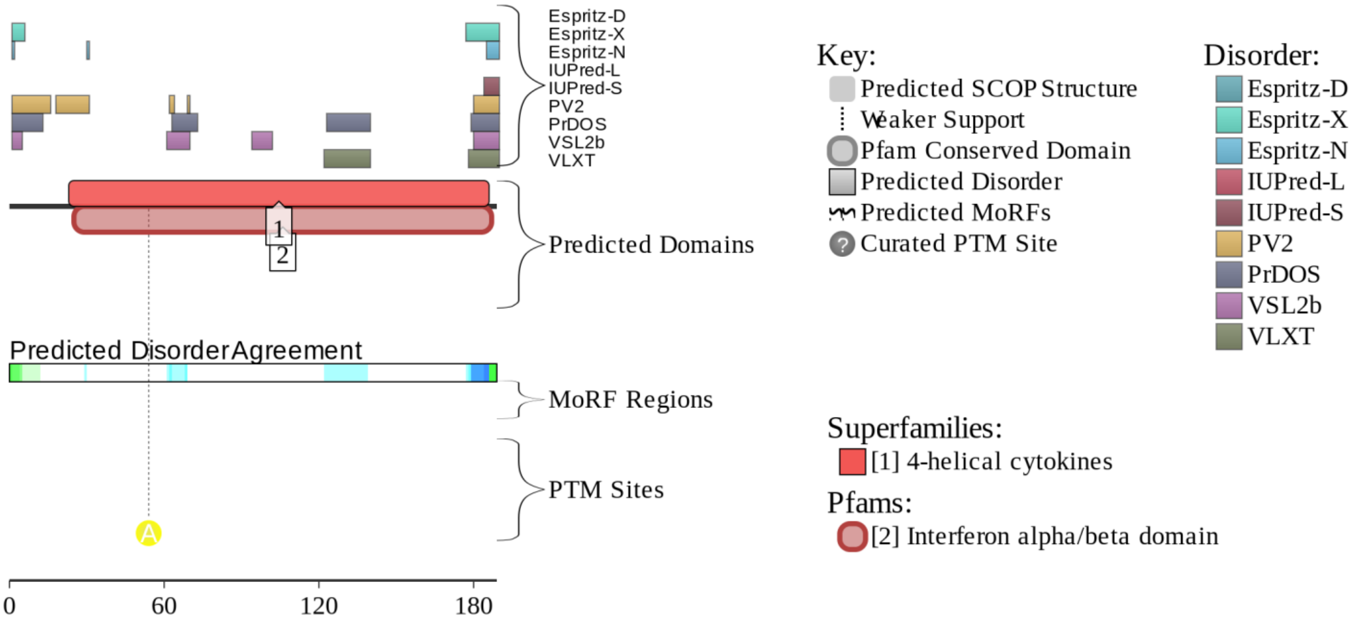

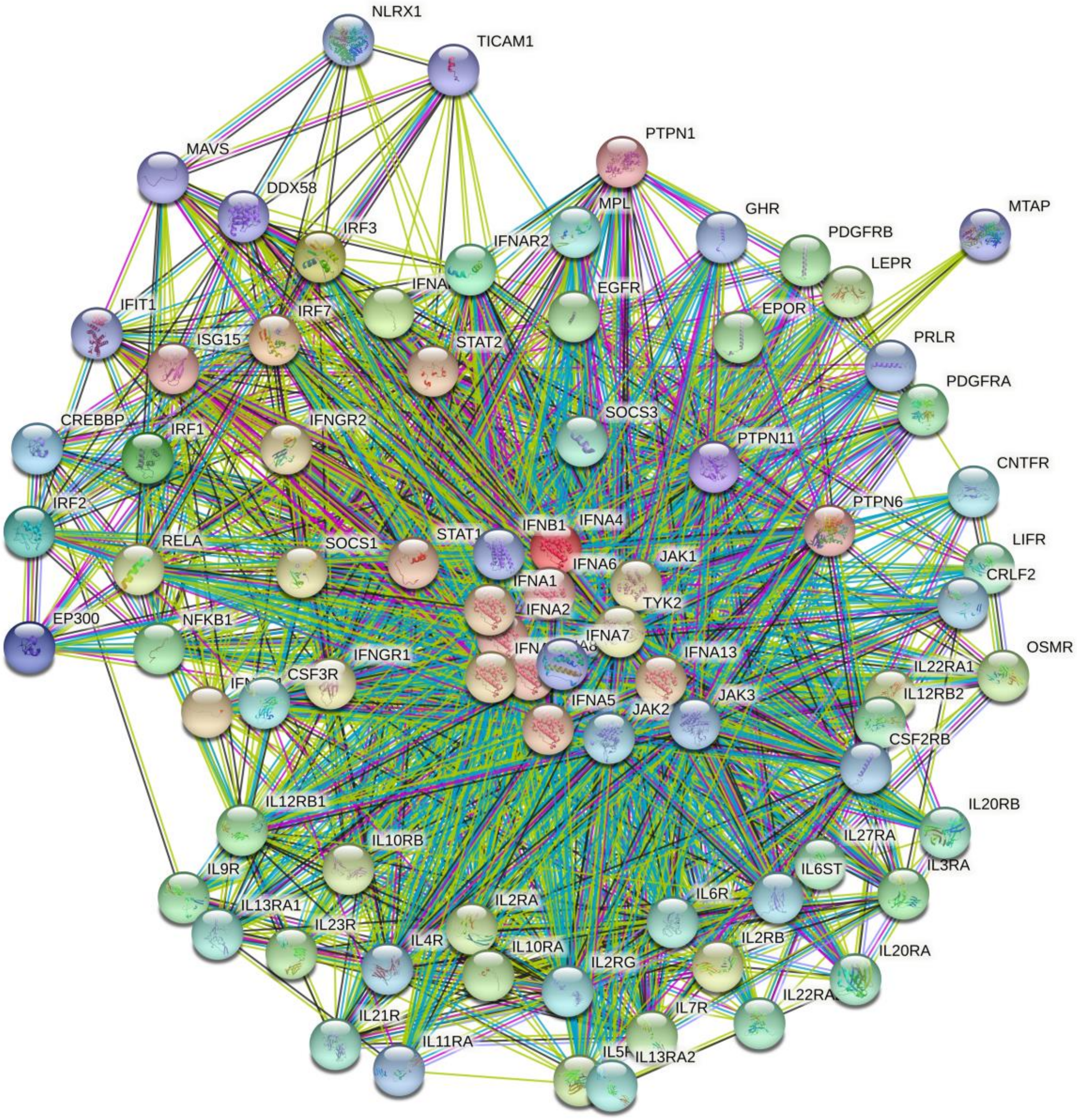
NLRX1
TICAM1
MAVS
DDX58
PTPN1
MPL
GHR
MTAP
IRF3
IFNAR2
PDGFRB
LEPR
IFIT1
IRF7
EGFR
EPOR
ISG15
STAT2
PRLR
PDGFRA
CREBBP
IRF1
IFNGR2
SOCS3
PTPN11
CNTFR
IRF2
PTPN6
RELA
SOCS1
STAT1
IFNB1
IFNA4
JAK1
LIFR
IFNA6
CRLF2
IFNA1
TYK2
EP300
NFKB1
IFNA2
IFNA7
OSMR
IFNGR1
IFNA13
CSF3R
IL22RA1
IL12RB2
JAK3
IFNA5
JAK2
CSF2RB
IL12RB1
IL20RB
IL10RB
IL27RA
IL9R
IL6ST
IL3RA
IL13RA1
IL6R
IL2RA
IL23R
IL4R
IL10RA
IL2RB
IL2RG
IL20RA
IL21R
IL7R
IL11RA
IL13RA2

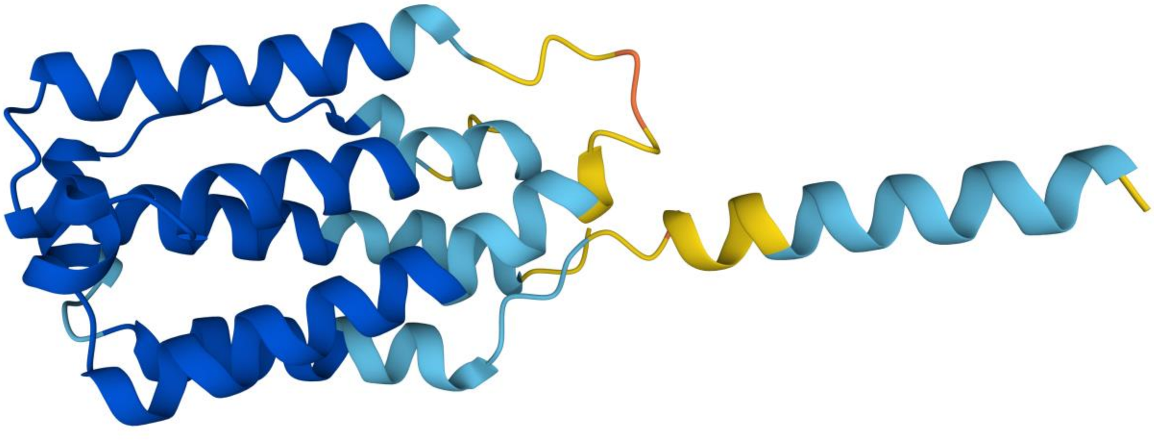

>sp|P08571|CD14_HUMAN Monocyte differentiation antigen CD14 OS=Homo sapiens OX=9606 GN=CD14 PE=1 SV=2
MERASCLLLLLLPLVHVSATTPEPCELDDEDFRCVCNFSEPQPDWSEAFQCVSAVEVEIHAGGLNLEPFLKRVDADADPRQYADT
VKALRVRRLTVGAAQVPAQLLVGALRVLAYSRLKELTLEDLKITGTMPPLPLEATGLALSSLRLRNVSWATGRSWLAELQQWLKP
GLKVLSIAQAHSPAFSCEQVRAFPALTSLDLSDNPGLGERGLMAALCPHKFPAIQNLALRNTGMETPTGVCAALAAAGVQPHSLD
LSHNSLRATVNPSAPRCMWSSALNSLNLSFAGLEQVPKGLPAKLRVLDLSCNRLNRAPQPDELPEVDNLTLDGNPFLVPGTALPH
EGSMNSGVVPACARSTLSVGVSGTLVLLQGARGFA

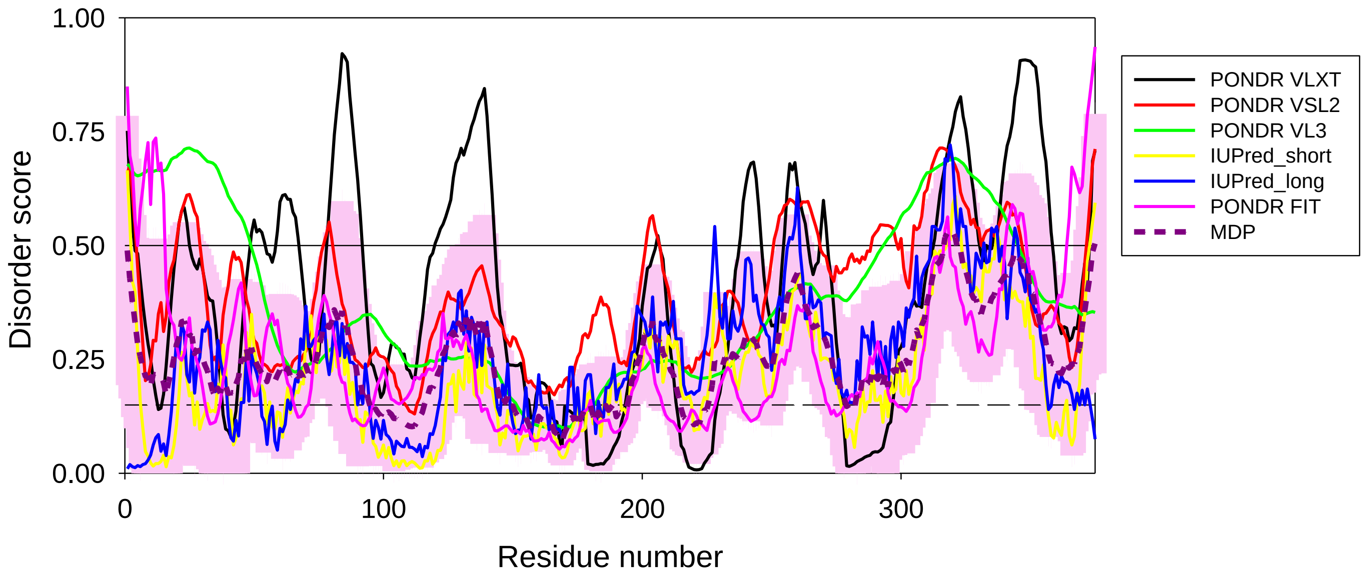

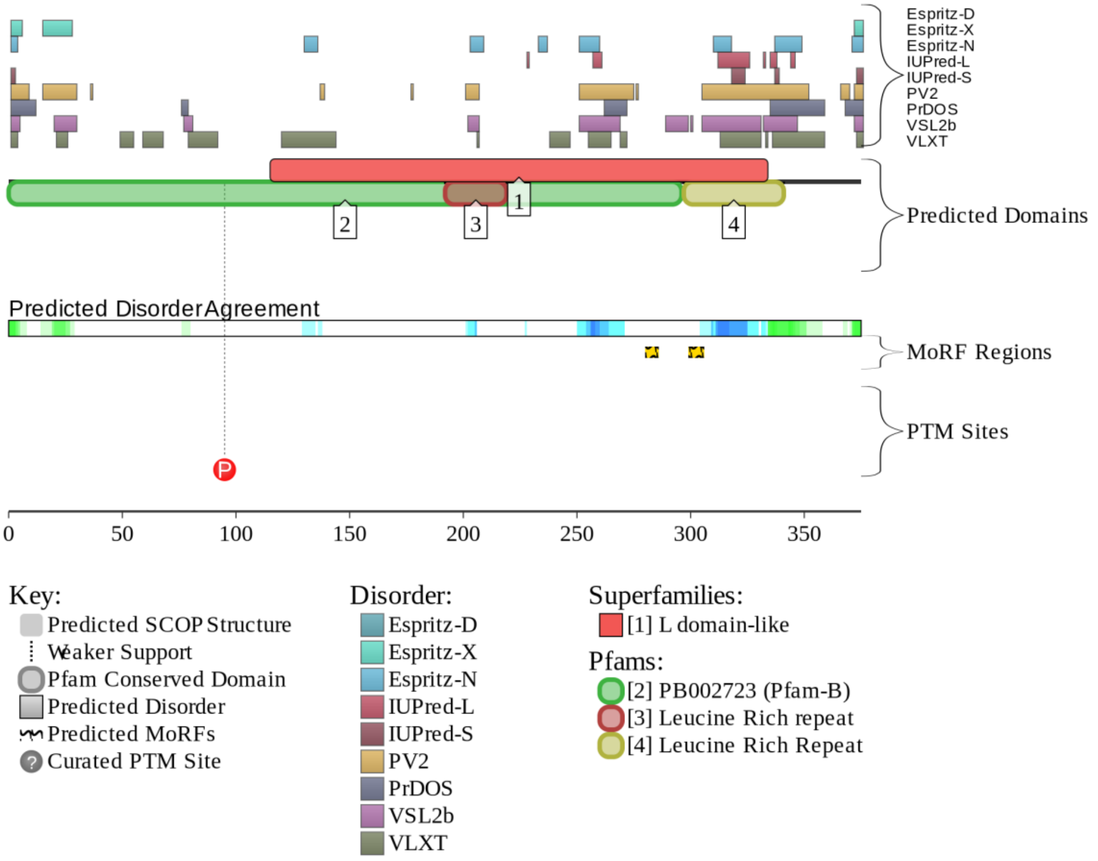

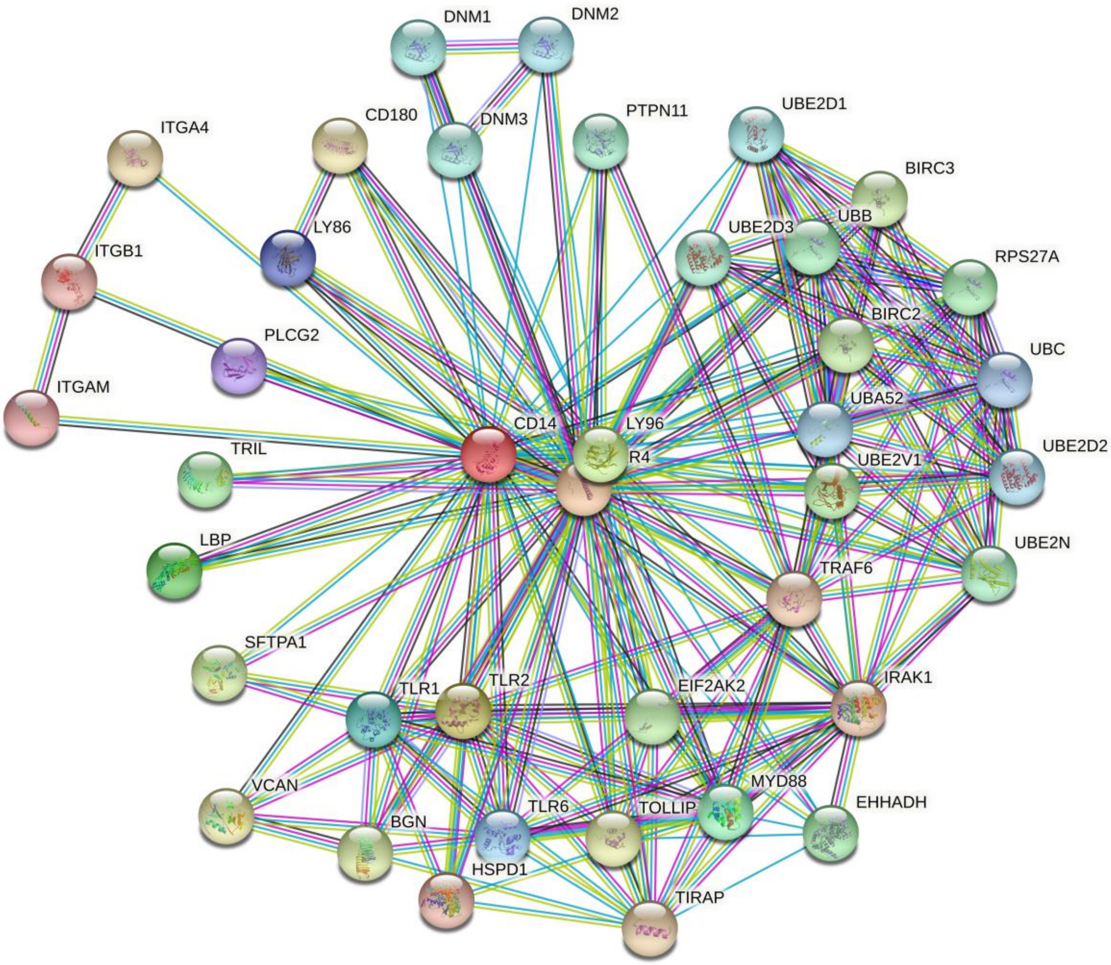
DNM1
DNM2
ITGA4
CD180
DNM3
PTPN11
UBE2D1
BIRC3
LY86
UBE2D3
UBB
ITGB1
RPS27A
BIRC2
PLCG2
UBC
ITGAM
UBA52
CD14
LY96
TRIL
R4
UBE2V1
UBE2D2
LBP
UBE2N
TRAF6
SFTPA1
TLR1
TLR2
EIF2AK2
IRAK1
VCAN
MYD88
EHHADH
BGN
TLR6
TOLLIP
HSPD1
TIRAP

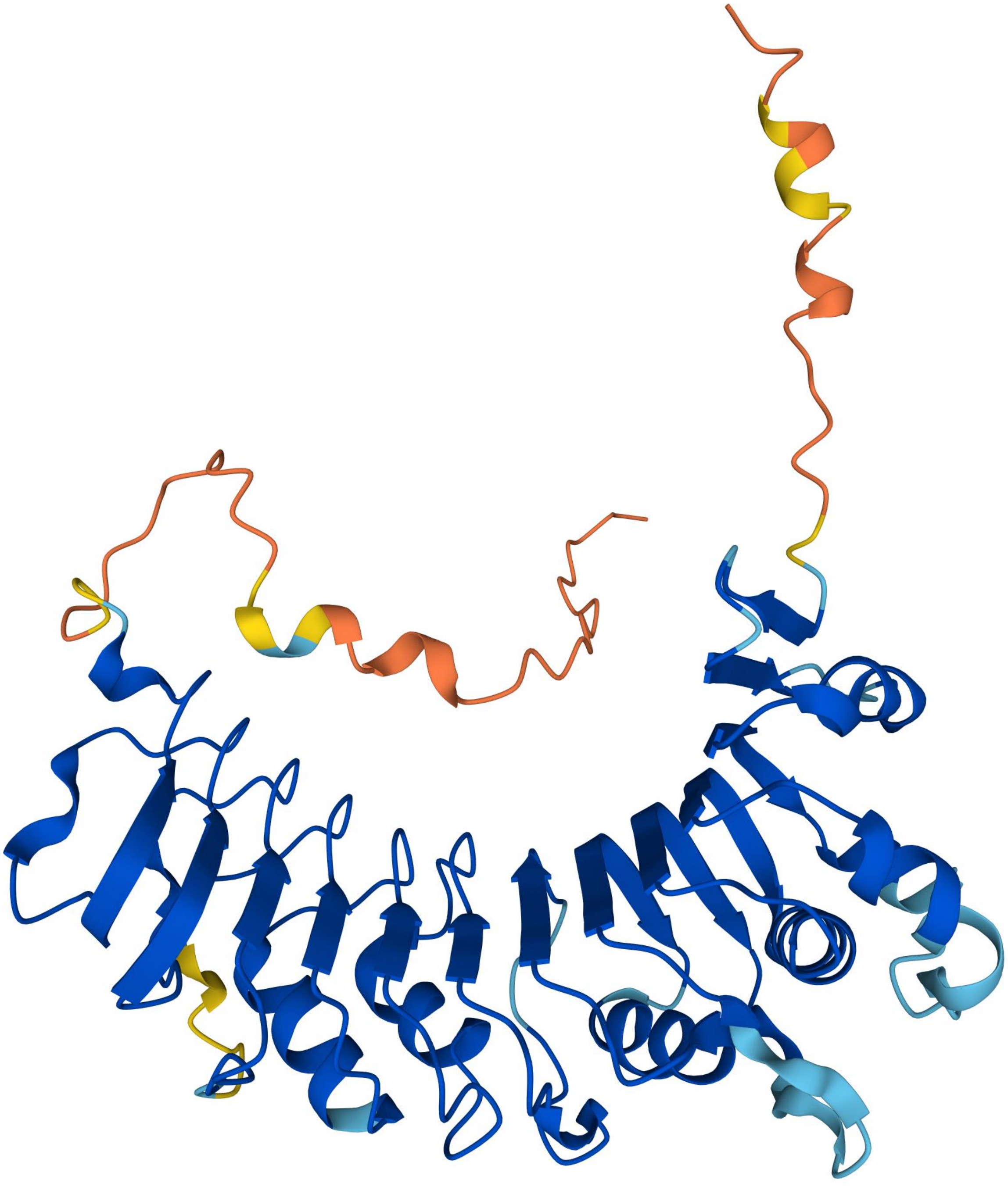

>sp|Q9Y6Y9|LY96_HUMAN Lymphocyte antigen 96 OS=Homo sapiens OX=9606 GN=LY96 PE=1 SV=2
MLPFLFFSTLFSSIFTEAQKQYWVCNSSDASISYTYCDKMQYPISINVNPCIELKRSKGLLHIFYIPRRDLKQLYFNLYITVNTM
NLPKRKEVICRGSDDDYSFCRALKGETVNTTISFSFKGIKFSKGKYKCVVEAISGSPEEMLFCLEFVILHQPNSN

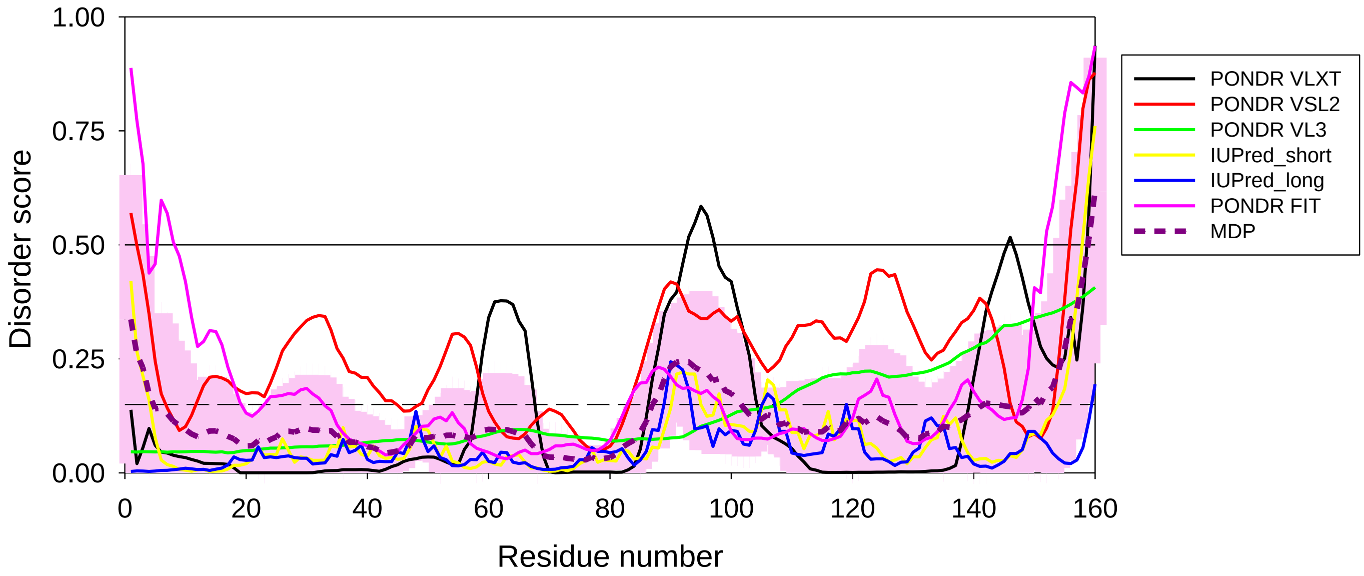

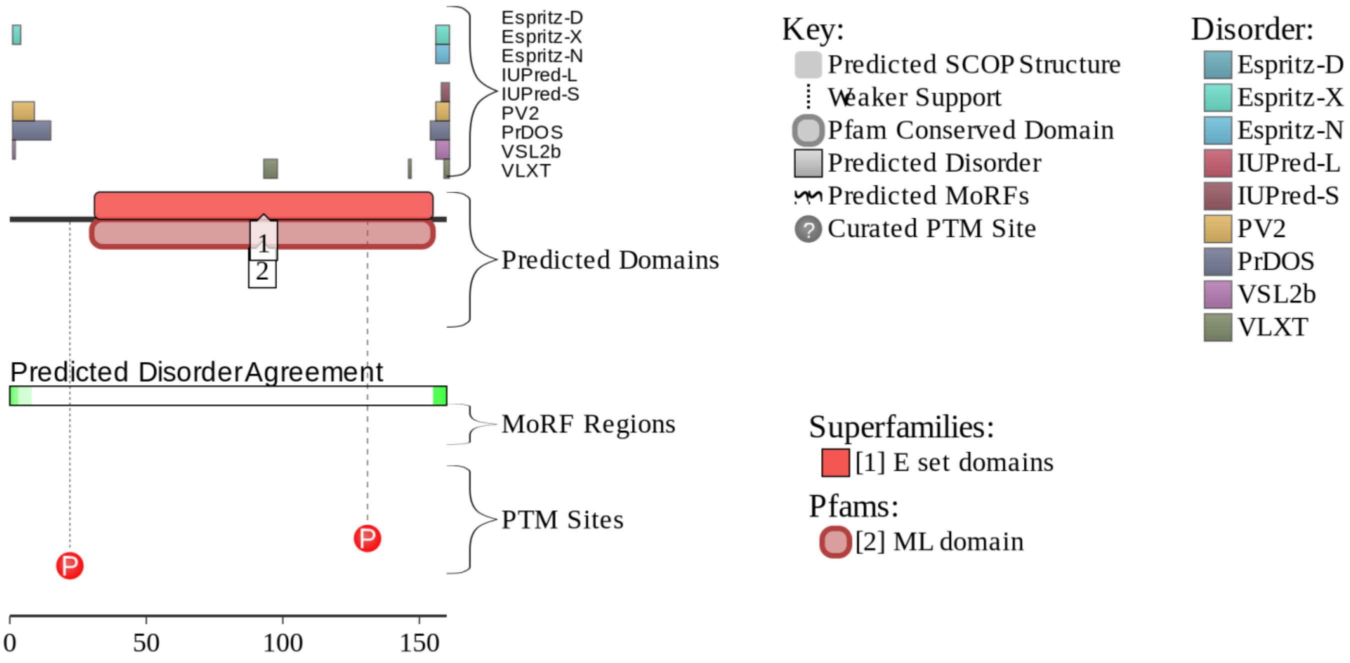

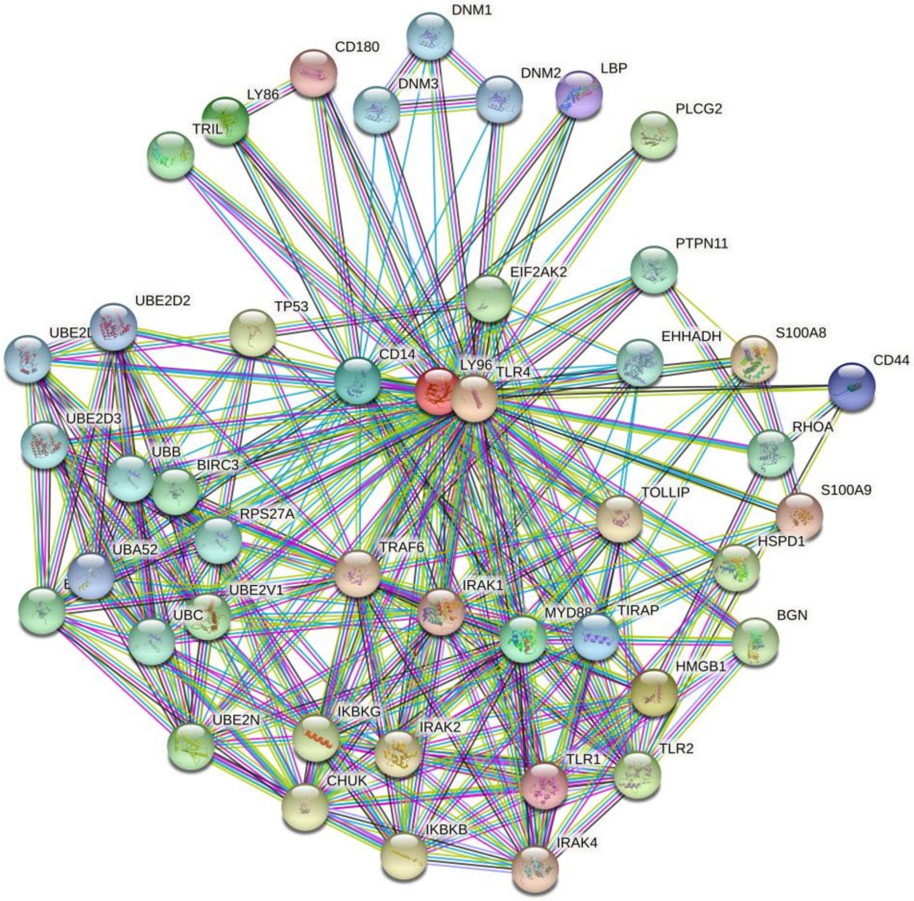
DNM1
CD180
LY86
DNM3
DNM2
LBP
TRIL
PLCG2
PTPN11
EIF2AK2
UBE2D2
TP53
S100A8
EHHADH
CD44
CD14
LY96
TLR4
UBE2D3
RHOA
UBB
BIRC3
S100A9
TOLLIP
RPS27A
HSPD1
UBA52
TRAF6
IRAK1
UBE2V1
UBC
TIRAP
BGN
HMGB1
UBE2N
IKBKG
IRAK2
TLR1
TLR2
CHUK
IKBKB
IRAK4

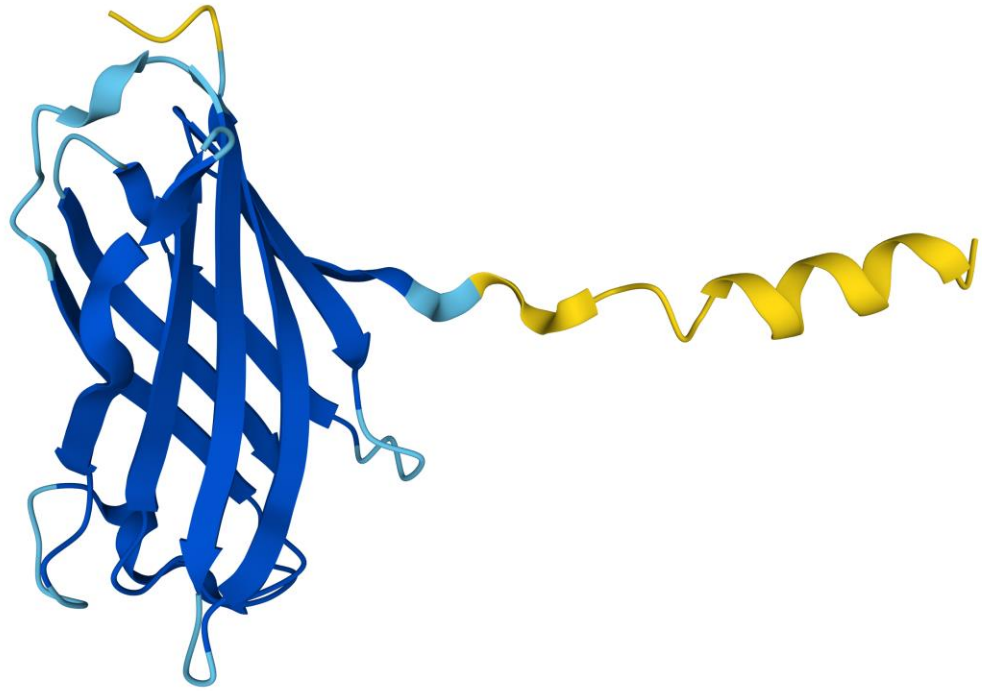

Supplementary Table S1. Some physico-chemical and intrinsic disorder-related features of HCV proteins, human TLRs, and major players of the TLR-regulated downstream signaling pathways

Protein name (UniProt ID)	Protein function	Length	PPIDR	ADS	Number of IDRs	Longest IDR	MoRFs	Number of interactors
**HCV proteome**								
HCV genome polyprotein (P27958)	Genome polyprotein, processed to individual viral proteins at maturation	3011	19.46	0.3224	40	2295-2417 (123)	1-52 (52)<br>76-94 (19)<br>2174-2213 (40)<br>2222-2234 (13)<br>2290-2301 (12)<br>2330-2412 (83)	259 (IntAct)
Core (2-177)	Mature core protein; packages viral RNA	176	68.18	0.6242	3	1-93 (93)	1-51 (51)<br>75-95 (21)	112[a]
E1 (192-383)	Envelope glycoprotein E1; mediates virus attachment to the host cell	192	6.77	0.2159	3	1-7 (7)	N/P	10[a]
E2 (384-746)	Envelope glycoprotein E2; mediates virus attachment to the host cell	363	9.37	0.2593	6	270-281 (12)	N/P	31[a]
p7 (747-809)	Viroporin; ion channel protein that acting in the assembly, envelopment and secretion of viral particles	63	12.70	0.1848	2	1-4 (4)<br>60-63 (4)	N/P	2 (UniProt)
NS2 (810-1026)	Cysteine protease; required for the proteolytic auto-cleavage	217	4.61	0.1579	2	1-5 (5)<br>213-217 (5)	N/P	31[a]
NS3 (1027-1657)	Serine protease/ helicase with three enzymatic activities: serine protease with a chymotrypsin-like fold, NTPase and RNA helicase	631	22.03	0.3442	16	109-142 (34)	N/P	242[a]
NS4A (1658-1711)	Non-structural protein 4A; non-covalent peptide cofactor of NS3 serine protease	54	18.52	0.2504	2	48-54 (7)	N/P	11[a]

NS4B (1712-1972)	Non-structural protein 4B; induces membrane alterations serving as a scaffold for the virus replication complex	261	9.20	0.2621	4	219-228 (10)	N/P	16[a]
NS5A (1973-2420)	Non-structural protein 5A; indispensable for viral replication and assembly	448	47.99	0.5411	6	323-448 (126)	202-241 (40)<br>250-262 (13)<br>318-329 (12)<br>358-448 (91)	139[a]
NS5B (2421-3011)	RNA-directed RNA polymerase; performs primer-template recognition and RNA synthesis during viral replication	591	15.40	0.3120	11	85-102 (18)	N/P	66[a]
**Human toll-like receptors**								
TLR1 (Q15399)	Toll-like receptor 1	786	11.58	0.2904	11	384-422 (39)	N/P	89 (0.400)[b]
TLR2 (O60603)	Toll-like receptor 2	784	14.92	0.3074	13	19-57 (39)	N/P	41[c]
TLR3 (O15455)	Toll-like receptor 3	904	13.05	0.3050	12	152-209 (58)	N/P	26
TLR4 (O00206)	Toll-like receptor 4	839	9.30	0.2670	10	359-381 (23)	N/P	84
TLR5 (O60602)	Toll-like receptor 5	858	7.11	0.2458	7	462-476 (15)<br>836-850 (15)	N/P	134 (0.400)
TLR6 (Q9Y2C9)	Toll-like receptor 6	796	7.16	0.2550	11	763-574 (12)	N/P	132 (0.400)
TLR7 (Q9NYK1)	Toll-like receptor 7	1049	12.77	0.3055	11	593-625 (33)<br>666-698 (33)	N/P	51 (0.700)[d]
TLR8 (Q9NR97)	Toll-like receptor 8	1041	18.64	0.3135	13	707-765 (59)	N/P	24 (0.700)
TLR9 (Q9NR96)	Toll-like receptor 9	1032	11.05	0.2857	14	62-86 (25)	N/P	54 (0.700)
TLR10 (Q9BXR5)	Toll-like receptor 10	811	9.12	0.2587	9	793-811 (19)	N/P	89 (0.400)
**Members of the signaling pathways induced by the toll-like receptors**								
MAL/ TIRAP (P58753)	MYD88 adapter-like; Toll/ interleukin-1 receptor domain-containing adapter protein	221	42.53	0.5508	3	1-88 (88)	1-9 (9)<br>14-33 (20)	24
MYD88 (Q99836)	Myeloid differentiation primary response protein MyD88	296	19.93	0.3572	3	109-130 (30)	N/P	66
IRAK1 (P51617)	IL-1 receptor-associated kinase 1	712	52.67	0.5516	9	501-712 (212)	139-147 (9)<br>158-163 (6)<br>492-502 (11)	67

							518-527 (10)<br>557-577 (21)<br>590-621 (32)<br>640-650 (11)<br>660-697 (38)	
IRAK4 (Q9NWZ3)	IL-1 receptor-associated kinase 4	460	33.48	0.4224	7	107-173 (67)	164-173 (10)<br>428-437 (10)	25
TRAF6 (Q9Y4K3)	TNF receptor-associated factor 6	522	23.37	0.3888	8	1-52 (52)	N/P	152
TAK1/ MAP3K7 (O43318)	Transformation growth factor-β (TGF-β)-activated kinase; Mitogen-activated protein kinase kinase kinase 7	606	57.10	0.5751	8	296-415 (120)	281-292 (12)<br>298-304 (7)<br>316-324 (9)<br>344-357 (14)<br>363-376 (14)<br>388-407 (20)<br>414-434 (21)<br>438-448 (11)<br>456-468 (13)<br>494-515 (22)<br>517-548 (32)<br>580-589 (10)	92
MAPK1 (P28482)	Mitogen-activated protein kinase 1; Extracellular signal-regulated kinase 2	350	16.67	0.3075	3	315-360 (46)	N/P	271
MAPK3 (P27361)	Mitogen-activated protein kinase 3; Extracellular signal-regulated kinase 1	379	22.96	0.3569	7	1-36 (36)	N/P	277
MAPK4 (P31152)	Mitogen-activated protein kinase 4	587	41.06	0.4576	11	467-546 (90)	333-344 (12)<br>353-362 (10)<br>422-443 (22)<br>454-465 (12)<br>486-500 (15)<br>532-552 (21)	59 (0.400)

							561-570 (10)<br>576-587 (12)	
MAPK6 (Q16659)	Mitogen-activated protein kinase 6	721	43.13	0.4644	16	616-721 (106)	468-475 (8)<br>527-534 (8)<br>602-611 (10)<br>627-633 (7)<br>664-679 (16)<br>713-721 (9)	61
MAPK7 (Q13164)	Mitogen-activated protein kinase 7	816	63.24	0.6389	6	340-751 (412)	363-368 (6)<br>379-389 (11)<br>395-432 (38)<br>444-456 (13)<br>466-501 (36)<br>543-592 (50)<br>609-633 (25)<br>649-732 (84)<br>737-760 (24)<br>771-785 (15)<br>800-816 (17)	187 (0.400)
MAPK8/JNK1 (P45983)	Mitogen-activated protein kinase 8; c-Jun N-terminal kinase 1	427	27.63	0.3735	5	361-427 (67)	319-324 (6)<br>352-363 (12)<br>377-387 (11)<br>397-402 (6)<br>413-427 (15)	162
MAPK9/JNK2 (P45984)	Mitogen-activated protein kinase 9; c-Jun N-terminal kinase 2	424	27.36	0.3783	6	358-424 (67)	320-325 (6)<br>352-362 (11)<br>391-424 (34)	65
MAPK10/JNK3 (P53779)	Mitogen-activated protein kinase 10; c-Jun N-terminal kinase 3	464	26.72	0.3747	6	376-464 (89)	356-363 (8)<br>390-399 (10)<br>431-464 (34)	49
MAPK11 (Q15759)	Mitogen-activated protein kinase 11; Mitogen-activated protein kinase p38 beta	364	21.15	0.3771	5	309-364 (56)	282-290 (9)<br>301-311 (11)<br>337-348 (12)	139

MAPK12 (P53778)	Mitogen-activated protein kinase 12; Mitogen-activated protein kinase p38 gamma	367	24.52	0.3432	5	311-367 (55)	286-293 (7)	43
MAPK13 (O15264)	Mitogen-activated protein kinase 13; Mitogen-activated protein kinase p38 delta	365	30.68	0.3859	4	254-336 (83)	301-308 (8)<br>341-350 (10)	36
MAPK14 (Q16539)	Mitogen-activated protein kinase 14; Mitogen-activated protein kinase p38 alpha	360	13.89	0.2952	6	313-327 (15)	N/P	207
MAPK15 (Q8TD08)	Mitogen-activated protein kinase 15	544	53.31	0.5137	8	315-516 (302)	280-288 (9)<br>329-350 (22)<br>361-384 (24)<br>401-429 (29)<br>434-484 (51)<br>516-536 (21)	39 (0.400)
AP-1/C-JUN (P05412)	Transcription factor Jun	331	82.78	0.7235	8	123-322 (200)	66-81 (16)<br>110-121 (12)<br>143-151 (9)<br>157-201 (45)<br>209-229 (21)<br>241-250 (10)<br>252-284 (33)<br>312-324 (13)	209
AP-1/C-FOS (P01100)	Protein c-FOS	380	83.42	0.7553	4	213-321 (112)	74-83 (10)<br>103-118 (16)<br>148-155 (8)<br>180-212 (33)<br>215-227 (13)<br>237-244 (8)<br>262-275 (14)<br>294-302 (9)	131
IKKα (O15111)	Inhibitor of nuclear factor-κB (NF-κB) kinase subunit alpha	745	24.56	0.3623	10	664-711 (48)	71-723 (11)<br>738-745 (8)	104

IKKβ (O14920)	Inhibitor of nuclear factor-κB (NF-κB) kinase subunit beta	756	35.45	0.4382	16	635-713 (79)	394-392 (9)<br>655-664 (10)<br>709-723 (15)<br>734-742 (9)	95
IKKγ (Q9Y6K9)	Inhibitor of nuclear factor-κB (NF-κB) kinase subunit gamma; NF-kappa-B essential modulator (NEMO)	419	98.57	0.8288	2	1-232 (232)	1-15 (15)<br>33-41 (9)<br>230-242 (13)<br>304-310 (7)<br>372-380 (9)<br>407-419(13)	116
IKKε (Q14164)	Inhibitor of nuclear factor-κB (NF-κB) kinase subunit epsilon	716	23.88	0.3811	13	470-542 (73)	679-698 (20)	20
NFKBIA/IKBA (P25963)	NF-kappa-B inhibitor alpha; I-kappa-B-alpha	317	39.75	0.4695	2	1-72 (72)	1-6 (6)<br>78-84 (9)<br>302-317 (16)	133
p65/RELA/NFKB3 (Q04206)	Transcription factor p65; Nuclear factor NF-kappa-B p65 subunit	551	64.61	0.6443	5	257-457 (201)	1-11 (11)<br>31-41 (11)<br>62-75 (15)<br>98-103 (6) 110-113 (9) 285-290 (6) 305-317 (13)<br>350-380 (31)<br>398-414 (17)<br>433-483 (51)<br>492-504 (13)<br>523-551 (29)	235
p50/NFKB1 (P19838)	Nuclear factor NF-kappa-B p105 subunit	968	33.16	0.4490	15	335-482 (148)	369-374 (6)<br>403-421 (19)<br>454-463 (10)<br>781-787 (7)<br>931-953 (23)	156
p52/NFKB2 (Q00653)	Nuclear factor NF-kappa-B p100 subunit	900	59.89	0.5837	14	690-900 (211)	8-21 (14)<br>39-44 (6)	41

							112-123 (12)<br>373-389 (17)<br>445-458 (14)<br>648-655 (8)<br>672-694 (23)<br>728-753 (26)<br>766-784 (19)<br>792-806 (15)<br>816-848 (33)<br>859-880 (22)<br>885-900 (16)	
IRF3 (Q14653)	Interferon regulatory factor 3	427	37.94	0.4565	9	84-196 (113)	52-60 (9)<br>105-118 (14)<br>135-168 (34)<br>202-211 (10)	109
IRF5 (Q13568)	Interferon regulatory factor 5	498	43.17	0.5151	8	106-242 (137)	19-29 (11)<br>39-47 (9)<br>88-105 (18)<br>115-124 (10)<br>135-142 (8)<br>149-195 (47)<br>206-221 (16)<br>231-253 (23)	68
IRF7 (Q92985)	Interferon regulatory factor 7	503	58.05	0.5520	12	125-268 (162)	59-65 (7)<br>91-108 (18)<br>120-132 (13)<br>155-205 (51)<br>216-242 (27)<br>252-258 (7)<br>270-278 (9)<br>288-315 (28)	104
TBK1 (Q9UHD2)	Serine/threonine-protein kinase TBK1; TANK-binding kinase 1	729	10.62	0.3349	12	644-704 (61)	N/F	52

CD14 (P08571)	Cluster of differentiation 14; Monocyte differentiation antigen CD14	375	25.87	0.2891	10	305-330 (26)	280-285 (6)<br>299-305 (7)	39
TANK (Q92844)	TRAF family member-associated NF-kappa-B activator	425	70.35	0.6059	8	162-267 (106)	12-21 (10)<br>54-65 (12)<br>73-78 (6)<br>142-159 (18)<br>228-236 (9)<br>263-270 (8)<br>286-299 (14)<br>337-344 (8)<br>394-403 (10)	32
TRIF/TICAM1	Toll-interleukin-1 receptor domain-containing adapter protein inducing interferon beta; TIR domain-containing adapter molecule 1	712	66.29	0.6621	9	124-393 (270)	96-104 (9)<br>185-194 (10)<br>201-223 (23)<br>228-267 (40)<br>273-341 (69)<br>374-384 (11)<br>392-403 (12)<br>567-578 (12)<br>588-595 (8)<br>649-666 (18)<br>673-703 (31)	34
RIP1/RIPK1 (Q13546)	Receptor-interacting protein 1; Receptor-interacting serine/threonine-protein kinase 1	671	47.84	0.4978	11	291-482 (192)	318-329 (12)<br>345-363 (19)<br>376-392 (17)<br>401-406 (6)<br>415-475 (61)<br>531-547 (17)	99
TRAM/TICAM2 (Q86XR7)	TRIF related adapter molecule; TIR domain-containing adapter molecule 2	235	50.21	0.5112	4	1-77 (77)	1-18 (18)<br>39-46 (8)<br>75-85 (11)	25
IFNA1 (P01562)	Interferon alpha	189	31.75	0.3759	4	22-56 (36)	N/P	74 (0.700)
IFNA4 (P05014)	Interferon alpha 4	189	16.40	0.3241	4	180-189 (10)	N/P	78 (0.400)

IFNB1 (P01574)	Interferon beta	187	11.76	0.2695	4	124-136 (13)	N/P	44

[a] Data on protein-protein interactions for HCV proteins are derived from [1]

[b] Interaction data are derived from STRING-based analysis using medium confidence (0.400) for a minimum required interaction score

[c] Interaction data are derived from STRING-based analysis using highest confidence (0.900) for a minimum required interaction score

[d] Interaction data are derived from STRING-based analysis using high confidence (0.700) for a minimum required interaction score

References

1. Fan, X.; Xue, B.; Dolan, P.T.; LaCount, D.J.; Kurgan, L.; Uversky, V.N. The intrinsic disorder status of the human hepatitis C virus proteome. *Molecular bioSystems* **2014**, *10*, 1345-1363, doi:10.1039/c4mb00027g.
